# Hepatic miR-149-5p upregulation fosters steatosis, inflammation and fibrosis development in mice and in human liver organoids

**DOI:** 10.1016/j.jhepr.2024.101126

**Published:** 2024-06-04

**Authors:** Marta Correia de Sousa, Etienne Delangre, Flavien Berthou, Sanae El Harane, Christine Maeder, Margot Fournier, Karl-Heinz Krause, Monika Gjorgjieva, Michelangelo Foti

**Affiliations:** 1 Department of Cell Physiology and Metabolism, Faculty of Medicine, University of Geneva, Geneva, Switzerland; 2 Department of Pathology and Immunology, Faculty of Medicine, University of Geneva, Geneva, Switzerland

**Keywords:** microRNAs, glucose metabolism, lipid metabolism, metabolic disruption, steatohepatitis, human liver organoids, MCD, FPC, miR-149-5p

## Abstract

**Background & Aims:**

The incidence of metabolic dysfunction-associated steatotic liver disease (MASLD) is increasing worldwide. Alterations of hepatic microRNA (miRNA) expression/activity significantly contribute to the development and progression of MASLD. Genetic polymorphisms of miR-149 are associated with an increased susceptibility to MASLD development in humans. Aberrant expression of miR-149 was also associated with metabolic alterations in several organs, but the impact of hepatic miR-149-5p deregulation in MASLD remains poorly characterized.

**Methods:**

MiR-149-5p was downregulated in the livers of mice by *in vivo* transduction with hepatotropic adeno-associated virus 8 harboring short-hairpin RNAs (shRNAs) specific for miR-149-5p (shmiR149) or scrambled shRNAs (shCTL). MASLD was then induced with a methionine/choline-deficient (MCD, n = 7 per group) diet or a fructose/palmitate/cholesterol-enriched (FPC, n = 8-12 per group, per protocol) diet. The impact of miR-149-5p modulation on MASLD development was assessed *in vivo* and *in vitro* using multi-lineage 3D human liver organoids (HLOs) and Huh7 cells.

**Results:**

MiR-149-5p expression was strongly upregulated in mouse livers from different models of MASLD (2-4-fold increase in *ob/ob*, *db/db* mice, high-fat and FPC-fed mice). *In vivo* downregulation of miR-149-5p led to an amelioration of diet-induced hepatic steatosis, inflammation/fibrosis, and to increased whole-body fatty acid consumption. In HLOs, miR-149-5p overexpression promoted lipid accumulation, inflammation and fibrosis. *In vitro* analyses of human Huh7 cells overexpressing miR-149-5p indicated that glycolysis and intracellular lipid accumulation was promoted, while mitochondrial respiration was impaired. Translatomic analyses highlighted deregulation of multiple potential miR-149-5p targets in hepatocytes involved in MASLD development.

**Conclusions:**

MiR-149-5p upregulation contributes to MASLD development by affecting multiple metabolic/inflammatory/fibrotic pathways in hepatocytes. Our results further demonstrate that HLOs are a relevant 3D *in vitro* model to investigate hepatic steatosis and inflammation/fibrosis development.

**Impact and implications:**

Our research shows compelling evidence that miR-149-5p plays a pivotal role in the development and progression of MASLD. By employing *in vivo* and innovative *in vitro* models using multi-lineage human liver organoids, we demonstrate that miR-149-5p upregulation significantly impacts hepatocyte energy metabolism, exacerbating hepatic steatosis and inflammation/fibrosis by modulating a wide network of target genes. These findings not only shed light on the intricate miR-149-5p-dependent molecular mechanisms underlying MASLD, but also underscore the importance of human liver organoids as valuable 3D *in vitro* models for studying the disease's pathogenesis.

## Highlights

• MicroRNAs contribute to the establishment and progression of metabolic liver diseases.
• Deregulation of miR-149-5p expression/activity fosters hepatic lipid accumulation, inflammation and fibrosis, promoting MASLD progression.
• Human liver organoids are relevant models that mimic hepatic metabolic pathologies from simple steatosis to fibrosis.

## Graphical abstract

**Figure:**
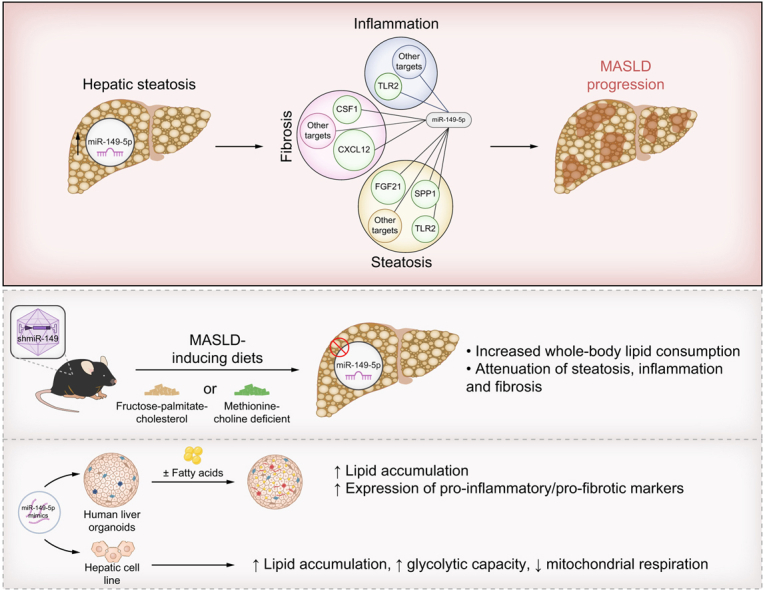

## Introduction

High-calorie diets and a sedentary lifestyle are key factors driving obesity. The lipotoxicity associated with excessive fat accumulation in the liver causes metabolic dysfunction-associated steatotic liver disease (MASLD), a progressive liver disease currently affecting >30% of the global population.^1^ MASLD represents a spectrum of hepatic disorders that starts with simple steatosis, where over 5% of hepatocytes accumulate lipids. The deregulated hepatic energetic metabolism and steatosis are tightly associated with insulin resistance (IR).^2^ If unresolved, hepatic steatosis/IR can progress to more severe pathologies like type 2 diabetes and metabolic dysfunction-associated steatohepatitis (MASH), characterized by extensive liver inflammation, hepatocyte ballooning and intra-sinusoidal fibrosis.^3^^,^^4^ The deregulation of microRNA (miRNA) expression and/or activity play a key role in the development and progression of multifactorial metabolic diseases.^5^^,^^6^ Many miRNAs and their function in hepatic metabolic diseases remain poorly characterized but have potential clinical applications as biomarkers or as addressable targets to prevent disease progression.

MiRNAs are small double-stranded molecules processed and incorporated in the RISC (RNA-induced silencing complex), where one strand (guide) directs RISC to inhibit protein translation through mRNA decay or translational blockage.^7^ Since one single miRNA can simultaneously target several mRNAs,^7^^,^^8^ multiple physiopathological mechanisms are fine-tuned through modulation of several target genes in parallel. Consistent with this feature, deregulation of specific hepatic miRNAs was reported to affect multiple gene networks involved in glucose homeostasis, cholesterol and lipid metabolism, energy expenditure, lipogenesis and inflammation, thus contributing to MASLD and IR/type 2 diabetes development.^6^^,^^9^^,^^10^ In this regard, upregulation of miR-149-5p was reported in a mouse model of diet-induced hepatic steatosis/IR^11^ and alterations of the two miR-149 strands were linked with metabolic dysfunction in different organs/cells, *i.e.* adipose tissue (AT),^12^^,^^13^ skeletal muscle,^14^ cultured hepatic cancer cells,[15], [16], [17] and adipocytes.^18^^,^^19^ Regarding the function of miR-149-5p in the liver, only fragmentary information is available. Depletion of miR-149 in mice increases sensitivity to acute liver injury induced by injection of lipopolysaccharides (LPS) or diethylnitrosamine, likely due to enhanced pro-inflammatory signaling pathways.^20^^,^^21^ Finally, *in vitro* studies using human hepatoblastoma HepG2 cells,^15^ mouse primary hepatocytes,^16^ or mouse AML12 cells^17^ suggested that miR-149-5p overexpression promoted intracellular lipid accumulation.

Herein, we investigated how miR-149-5p expression is modulated in the liver upon MASLD development in genetic and diet-induced mouse models, as well as the impact of deregulated expression of miR-149-5p on steatosis, inflammation and fibrosis development/progression in mice and in stem cell-derived multi-lineage 3D human liver organoids.

## Materials and methods

### Animals

Handling and experimental procedures were approved by the Geneva Health Head Office (authorization number GE/175) and performed following the Swiss guidelines for animal experimentation. Five-week-old males (C57BL6/J) were provided by Charles River Labs (France). Different mouse models of diet-induced MASLD/MASH were used: methionine/choline-deficient (MCD) and fructose/palmitate/cholesterol-enriched (FPC) diets. For downregulation of mir-149-5p, mice were injected retro-orbitally with adeno-associated virus 8 packed with short-hairpin (shRNA) specific for miR-149 (shmiR149) or scrambled shRNA (shCTL) and GFP reporter.

### *In vitro* models

Multi-lineage 3D human liver organoids (HLOs) and hepatic cell line Huh7 were transfected with synthetic oligonucleotides mimicking miR-149-5p. HLOs were used to investigate the impact of miR-149-5p overexpression on steatosis, inflammation and fibrosis development. Live metabolic characterization of Huh7 cells overexpressing miR-149-5p was performed with Seahorse MitoStress, GlycoRate and Substrate Oxidation Stress test (Agilent) and translatomic analyses were performed to identify potential miR-149-5p targets. Glucose uptake, lipid accumulation, mitochondrial function and insulin sensitivity was also assessed.

Detailed information on the materials and methods used are provided in the supplementary materials.

## Results

### MicroRNA-149-5p is upregulated in the liver of mice with steatosis

**Fig. 1:**
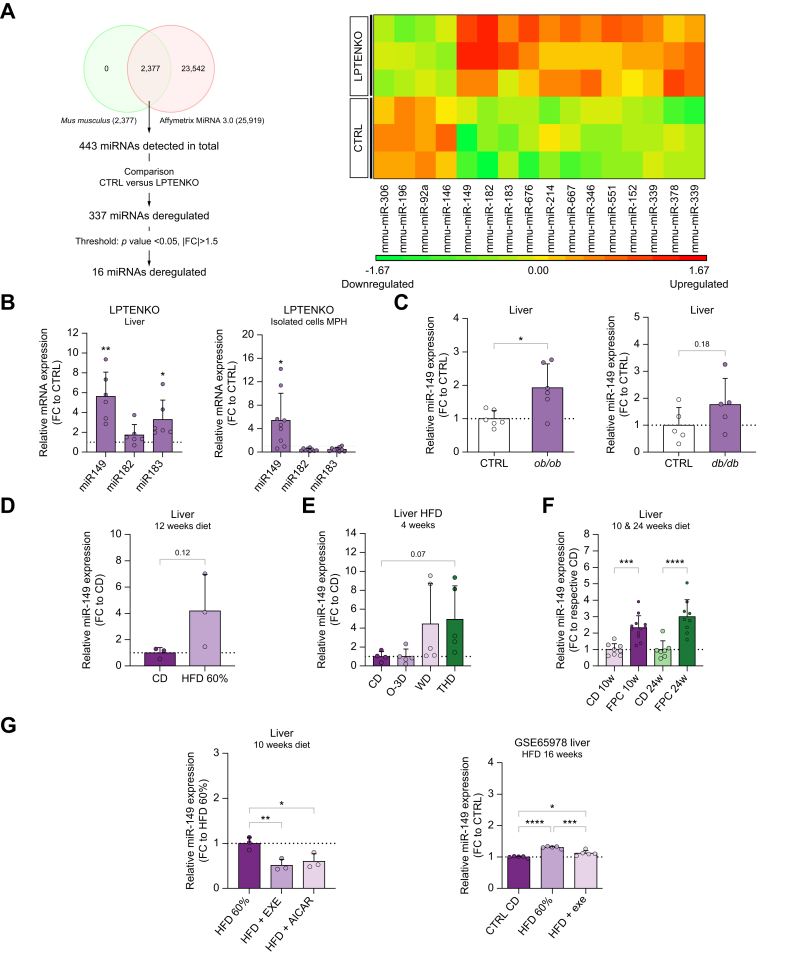
MiR-149-5p expression is consistently upregulated in several mouse models of MASLD and is modulated by AMPK activity. (A) Experimental microarray pipeline (left) and heatmap representation of differentially expressed hepatic miRNAs (right) and (B) relative miRNA expression in hepatic tissues and in MPHs from 4-month-old mice (CTRL) and LPTENKO mice. Relative hepatic miR-149-5p expression in (C) leptin signaling-deficient (*ob/ob* and *db/db*) and in diet-induced obesity/MASLD mouse models: (D) mice fed with CD or HFD, (E) mice fed with CD, HFD supplemented with O-3D or with THD or WD (FC to CD), (F) mice fed with CD or FPC diet. (G) Relative hepatic miR-149-5p expression in mice fed HFD submitted to exercise (HFD+EXE) or injected with AMPK-activators (HFD+AICAR) (left, FC to HFD) and in a gene expression omnibus dataset of mice fed CD or HFD 60%, forced to exercise or not (right). ∗*p <*0.05, ∗∗*p <*0.01, ∗∗∗*p <*0.001, ∗∗∗∗*p <*0.0001. Unpaired t-test with Welch’s correction or One-way ANOVA with Holm-Sidak’s correction. CD, chow diet; FPC, fructose/palmitate/cholesterol-enriched diet; HFD, high-fat diet; LPTENKO, liver-specific *Pten* knockout; MASLD, metabolic dysfunction-associated steatotic liver disease; MPHs, murine primary hepatocytes; O-3D, omega 3 fatty acids; THD, trans-unsaturated fatty acids; WD, western diet.

Microarray analysis of miRNA expression in liver tissues from 4-month-old liver-specific *Pten* knockout (LPTENKO) mice^22^ and corresponding wild-type littermates was performed. From a total of 443 miRNAs detected by the microarray, only 16 (4 downregulated miRNAs and 12 upregulated miRNAs) were within the established threshold (Fig. 1A). The top three most upregulated miRNAs identified were selected for further validation by quantitative reverse-transcription PCR in additional biological samples (miR-149, miR-182, miR-183, Fig. 1B). Only miR-149-5p was strongly upregulated in liver tissues of LPTENKO mice and in isolated hepatocytes of LPTENKO mice. The modest upregulation of miR-183 observed originated from non-parenchymal cells (Fig 1B and Fig. S1A). Of note, a trend for an increased circulating level of miR-149-5p in the plasma of LPTENKO was also observed (Fig. S1B).

To determine whether miR-149-5p upregulation is a general feature in MASLD, miR-149-5p expression was assessed in the liver of different genetic or diet-induced MASLD murine models. Leptin signaling-deficient model of steatosis, diabetes and obesity (*ob/ob* and *db/db* mice) displayed a similar increase in hepatic miR-149-5p expression (Fig. 1C). Mice with diet-induced steatosis (high-fat diet – HFD; 60% fat) also had increased hepatic miR-149-5p levels (Fig. 1D). The nature of fatty acids was also a determinant for steatosis development and miR-149-5p upregulation. In mice fed a diet containing 45% fat, miR-149-5p was upregulated in mice with steatosis (fed with saturated or with trans-fatty acids but not with omega-3, Figs 1E and S2). Upregulation of hepatic miR-149-5p was also observed in mice fed a FPC diet for 10 and 24 weeks (Fig. 1F). Of note, hepatic miR-149-5p expression tends also to be upregulated in mice fed a MCD diet (Fig. S3A), which was further confirmed by the analysis of a gene expression omnibus (GEO) dataset (GSE55593) (Fig. S3B). Since miR-149-5p expression was previously reported to be modulated by exercise in skeletal muscle cells,^23^ we examined hepatic miR-149-5p expression in mice fed a HFD and challenged with treadmill exercise.^24^ Physical activity in mice fed a HFD (HFD+EXE) decreased steatosis,^24^ as well as hepatic miR-149-5p expression (Fig. 1G, left panel). The same effect on miR-149-5p expression was observed in mice fed a HFD administered with AICAR (5-aminoimidazole-4-carboxamide ribonucleotide), a specific AMPK activator (HFD+AICAR; Fig. 1G, left panel). Similar observations were obtained with the analysis of a GEO dataset (GSE65978) of miRNA expression in mice fed a HFD and forced to exercise (HFD+exe) (Fig. 1G, right panel).

### Hepatocyte-specific microRNA-149-5p knock down decreases diet-induced steatosis, inflammation and fibrosis development

To assess the functional role of miR-149-5p on MASLD development/progression *in vivo*, miR-149-5p expression was downregulated specifically in hepatocytes of C57BL6/J mice. Post-transduction, mice were challenged with FPC diet (short and long FPC)^25^ or with MCD diet.^26^

**Fig. 2:**
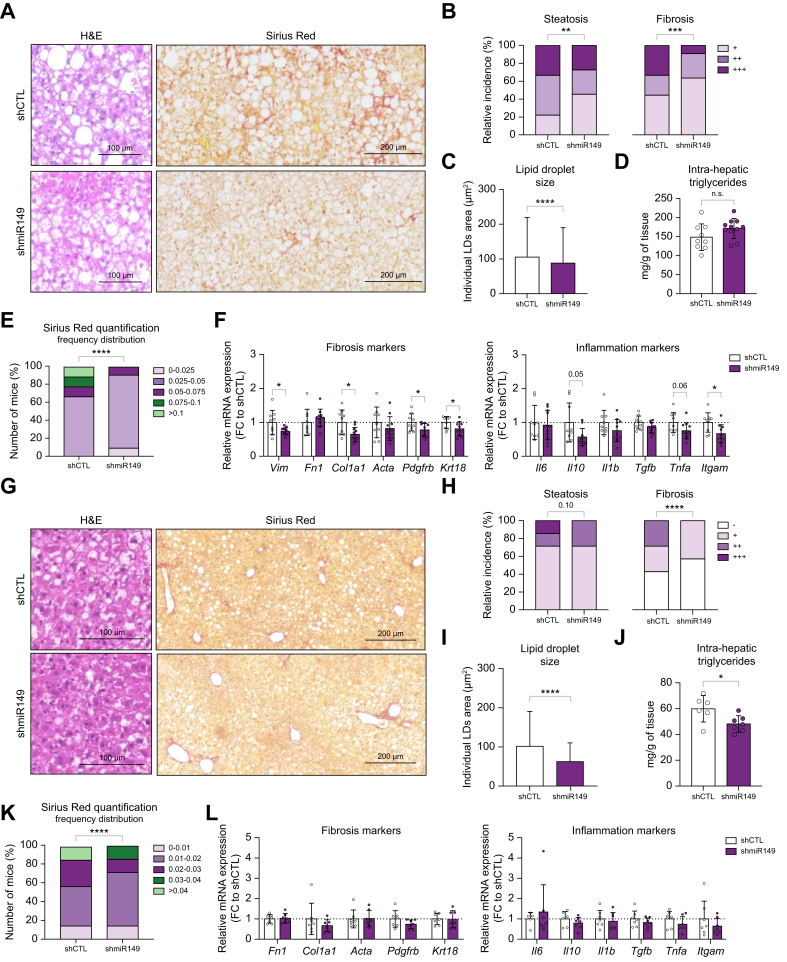
*In vivo* downregulation of miR-149-5p specifically in hepatocytes attenuates hepatic steatosis, inflammation and fibrosis induced by FPC or MCD diets. (A, G) Representative histopathological sections: H&E (left panel) and Sirius red (right panel) staining, magnification 20x, (B, H) blind scoring of steatosis and fibrosis with corresponding (C, I) quantification of individual LD size, (D, J) intrahepatic triglyceride content, (E, K) frequency distribution of the quantification of Sirius Red staining (positive area/total tissue area) and (F, L) relative mRNA expression of fibrosis and inflammation markers in explanted livers of FPC and MCD diet-fed mice. ∗*p <*0.05, ∗∗*p <*0.01, ∗∗∗*p <*0.001, ∗∗∗∗*p <*0.0001. Unpaired t-test with Welch’s correction, one-way ANOVA with Holm-Sidak’s correction or Fisher’s test. FPC, fructose/palmitate/cholesterol-enriched diet; LD, lipid droplet; MCD, methionine/choline-deficient diet.

Downregulation of miR-149-5p in hepatocytes from mice subjected to a FPC diet did not significantly affect general body parameters – body and liver weight, lean and fat mass body composition, adipose tissue size, plasma transaminases and triglycerides (TGs) levels (Fig. S4A-F). Blind scoring of steatosis and quantification of lipid droplet number and size on histological sections from liver tissues revealed a moderate attenuation of steatosis in mice following miR-149-5p downregulation, with a significant decrease in *Cd36, Cpt1a and Acox1* gene expression in the long FPC, despite no significant differences in intrahepatic TGs in either (Figs 2A-D and S5A-D). Scoring and quantification of Sirius Red staining indicated attenuation of fibrosis with downregulation of miR-149-5p (Fig. 2B,E). These histological observations were further supported by a significant decrease of molecular markers of fibrosis and inflammation (Figs 2F and S6). Activation of pro-inflammatory pathways previously linked with miR-149-5p expression^27^ were also assessed by western blot (NF-κB, p38, JNK and STAT3, Fig. S7A-D) in hepatic tissues of long FPC-fed mice, as well as macrophage infiltration (Iba1+ cells, Fig. S7E). We did not observe significant differences between mice with miR-149-5p downregulated and the control group.

Despite inducing weight loss, the MCD diet promotes severe steatosis, steatohepatitis and mild fibrosis in mice but through different mechanisms than obesogenic diets.^26^ miR-149-5p downregulation in mice fed a MCD diet attenuated weight loss but did not affect liver weight (Fig. S8A) nor plasma transaminase, TG or cholesterol levels (Fig. S8B-D). Histopathological analysis of H&E staining and measurement of intrahepatic TGs also indicated slightly attenuated steatosis (decreased lipid droplet size and TG content), despite no changes in the expression of genes involved in lipid uptake/oxidation (Figs 2G-J and S9A-B). Despite the mild steatosis, inflammation and fibrosis induced by MCD in our control cohort, blind assessment and quantification of Sirius Red staining also pointed to decreased fibrosis with miR-149-5p downregulation (Fig. 2H,K). A strong impact of miR-149-5p downregulation on fibrotic/inflammatory markers expression could not be confirmed (Fig. 2L).

### Hepatic miR-149-5p modulates whole-body metabolism and lipid utilization with MASLD

**Fig. 3:**
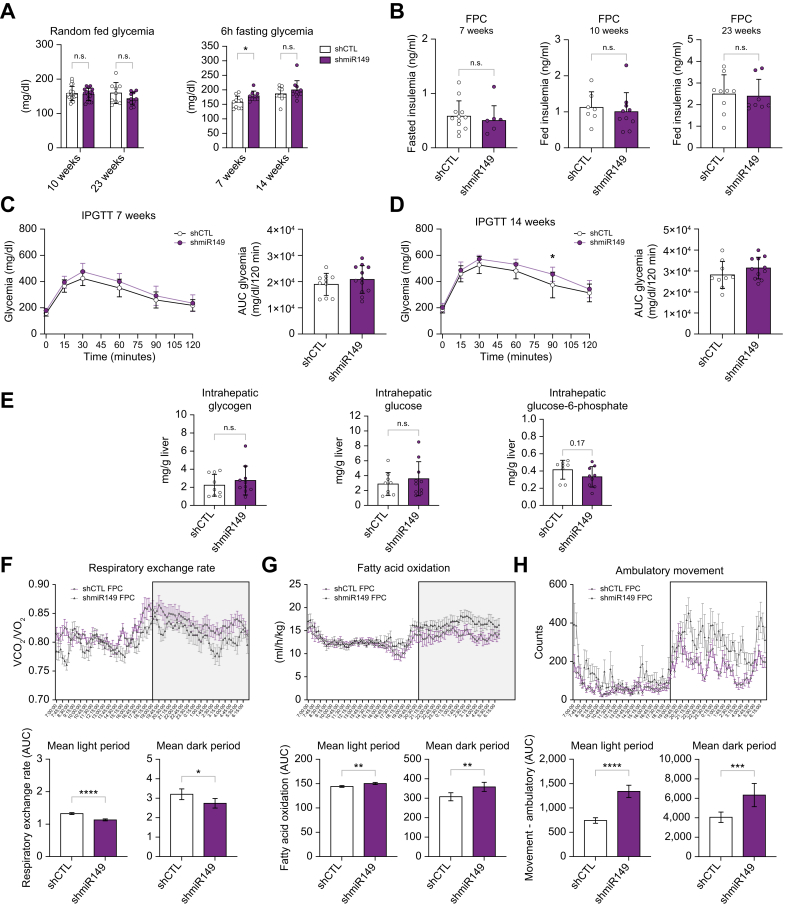
Hepatic miR-149 contributes to the regulation of fasting glycemia and modulates whole-body lipid utilization in FPC-fed mice. (A) Glycemia measurements in fed (left) and fasting states (right). (B) Plasma insulin levels in fasting and fed states. (C, D) IPGTT (left) and calculated AUC (right) at 7 and 14 weeks of diet. (E) Intrahepatic glycogen, glucose and glucose-6-phosphate in explanted tissues. (F) Respiratory exchange rate (carbon dioxide/oxygen consumption (VCO_2_/VO_2_), (G) fatty acid oxidation rate, and (H) ambulatory movement measured over 7 days (mean, left) and corresponding AUCs (light *vs*. dark period, right). ∗*p <*0.05, ∗∗*p <*0.01, ∗∗∗*p <*0.001, ∗∗∗∗*p <*0.0001. Unpaired t-test with Welch’s correction. FPC, fructose/palmitate/cholesterol-enriched diet; IPGTT, intraperitoneal glucose tolerance test; AUC, Area under curve.

Along with MASLD, the FPC diet also induces insulin resistance and glucose intolerance in mice.^25^ We investigated whether downregulation of hepatic miR-149 improved glucose tolerance in FPC-fed mice. Glycemia in fed state was not affected by miR-149-5p downregulation, but fasted glycemia was slightly increased after 7 weeks of diet (Fig. 3A). Insulinemia was unchanged between shCTL or shmiR149 mice (Fig. 3B), and glucose tolerance was not improved either (Fig. 3C,D). The increased glycemia in fasted states was not due to exacerbated hepatic glucose output as assessed by pyruvate tolerance tests (Fig. S10). Intrahepatic glycogen, glucose and glucose-6-phosphate content were also unchanged (Fig. 3E). However, whether hepatic miR-149-5p modulates liver-to-peripheral crosstalk mechanisms controlling glucose/lipid uptake and/or utilization remains unclear. To investigate hepatic miR-149-5p’s impact in whole-organism energy metabolism, we submitted FPC-fed mice to metabolic cages. Food and water intake was unchanged between groups (Fig. S11A,B). The respiratory exchange rate was significantly decreased indicating that fatty acid oxidation was upregulated, and carbohydrate utilization was decreased in mice with downregulated hepatic miR-149-5p (Fig. 3F,G). A significant increase in the ambulatory movement was observed in mice with hepatic miR-149-5p downregulation during both the light/dark period (Fig. 3H), while a mild decrease in energy expenditure was observed during the light period (Fig. S11C), likely associated with factors unrelated to food consumption or body size/composition.

### MiRNA-149-5p overexpression triggers lipid accumulation, inflammation and fibrosis in multi-lineage 3D HLOs

**Fig. 4:**
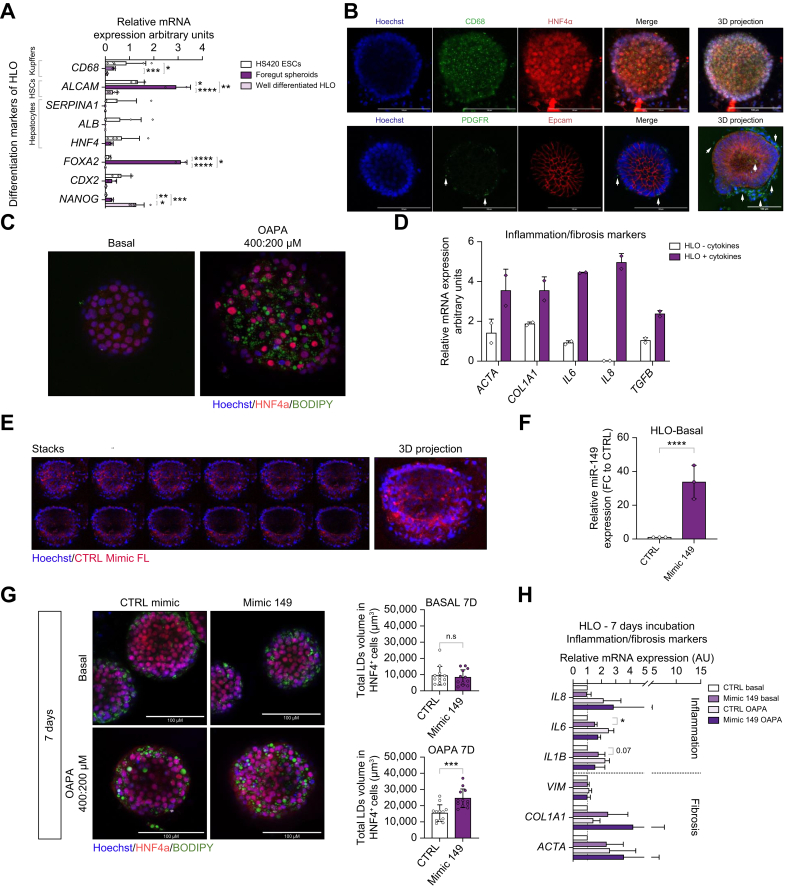
Overexpression of miR-149-5p in HLOs aggravates steatosis, inflammation and fibrosis development. (A) Differentiation markers in HS420 ESCs, in foregut spheroids and in HLOs. Representative confocal images of (B) HNF4+/Epcam+ (red, hepatocyte-like cells), PDGFR+ (green, stellate-like cells) and CD68+ (green, Kupffer-like cells) immunofluorescence in HLOs, and of (C) BODIPY staining (green), Hoechst staining (blue) and HNF4 immunofluorescence (red) in HLOs exposed to fatty acids. (D) Expression of inflammation/fibrosis markers in HLOs exposed to cytokines. (E) Representative stacks and 3D projection of confocal images of HLOs transfected with a fluorescent scrambled oligonucleotide (CTRL Mimic FL, red). Nuclei are stained by Hoechst (blue). (F) Relative miR-149-5p expression in HLOs transfected with scrambled oligonucleotides (CTRL) or miR-149-5p oligonucleotides (Mimic 149). (G) Representative images of BODIPY staining (green), Hoechst staining (blue) and HNF4 immunofluorescence (red) with corresponding quantification of BODIPY signal per individual organoid and (H) relative expression of inflammation/fibrosis markers in HLOs overexpressing or not miR-149-5p and challenged with pro-steatotic/pro-inflammatory conditions (n = 3-4 per group). ∗*p <*0.05, ∗∗*p <*0.01, ∗∗∗*p <*0.001, ∗∗∗∗*p <*0.0001. Unpaired t-test with Welch’s correction or One-way ANOVA with Holm-Sidak’s correction. ESCs, embryonic stem cells; HLOs, human liver organoids.

To evaluate the relevance of our *in vivo* findings in the context of the human pathology, we investigated the outcome of miR-149-5p overexpression in multi-lineage 3D HLOs. HLOs were generated following optimization of a previously described protocol.^28^^,^^29^ Differentiation of HLOs was validated by the loss of nanog homeobox (*NANOG*, stem cell marker), caudal type homeobox 2 (*CDX2*, mid-gut marker) and forkhead box A2 (*FOXA2*, foregut marker) expression, and increased levels of hepatocyte markers (hepatocyte nuclear factor 4 alpha (*HNF4α*), albumin (*ALB*) and α-antitrypsin (*SERPINA1*)), stellate cells marker (activated leukocyte cell adhesion molecule (*ALCAM*)) and Kupffer cell marker (*CD68,* Fig. 4A). The presence of hepatocyte-like (HNF4+/Epcam+), stellate-like (PDGFR+) and Kupffer-like cells (CD68+) was also validated by immunofluorescence (Fig. 4B). Exposure to fatty acids (400:200 μM oleate:palmitate [OAPA] for 2 days) generated steatosis in HLOs, as evidenced by BODIPY staining of neutral lipid-containing droplets in hepatocyte-like cells (HNF4+, Fig. 4C). Challenge with inflammatory cytokines induced inflammation/fibrosis in HLOs, as supported by the increase in pro-fibrotic/inflammatory gene expression (Fig. 4D).

Transfection efficiency of HLOs using small oligonucleotides was assessed using fluorescent microRNAs (Fig. 4E) and miR-149-5p overexpression was validated by quantitative reverse-transcription PCR (Fig. 4F). Quantification of lipid droplet volume in HNF4+ cells indicated that HLOs overexpressing miR-149-5p display significantly increased lipid accumulation in hepatocyte-like cells upon OAPA exposure for 7 days, but not in basal conditions (Fig. 4G). Despite the batch variability, in HLOs treated with OAPA for 7 days, a mild inflammation and fibrosis also develops (increased expression of markers, white *vs*. light pink bars, Fig. 4H), as previously reported.^28^^,^^30^ In HLOs overexpressing miR-149-5p, different inflammatory/fibrotic markers were increased both in basal and OAPA conditions (Fig. 4H).

### Aberrant lipid accumulation due to increased glycolysis and defective mitochondrial function in Huh7 cells overexpressing miR-149-5p

**Fig. 5:**
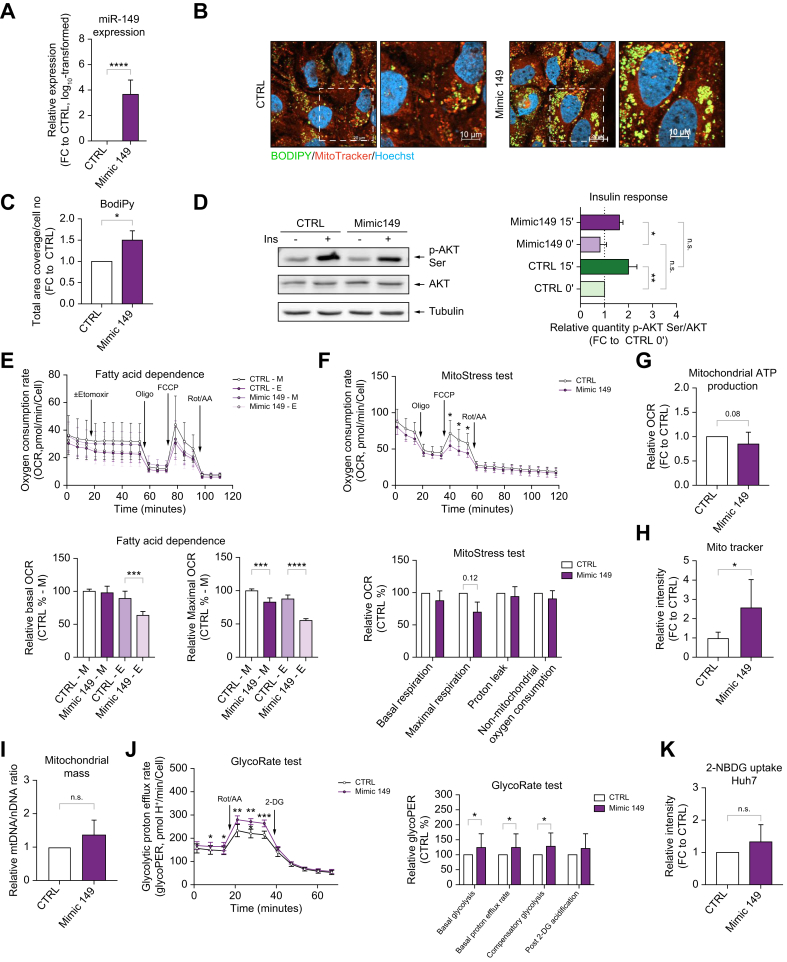
MiR-149-5p overexpression promotes intracellular lipid accumulation in Huh7 cells by promoting glucose metabolism and impairing lipid catabolism. (A) Relative miR-149-5p expression in transfected Huh7 cells. Data was log10 transformed. (B) Representative pictures of MitoTracker (red), Hoechst (blue) and BODIPY (green) staining in transfected Huh7 cells. Measurement of (C) total area coverage of lipid droplets normalized to cell number in transfected Huh7 cells. (D) Representative western blot analysis (left panel) and respective quantification (right panel) of the ratio of phosphorylated AKT (Serine 473)/total AKT in transfected Huh7 cells. (E) Fatty acid dependence test and (F) mitochondrial respiration rates through measurement of OCR (profile in the upper panel and relative parameters in the lower panel), (G) mitochondrial ATP production, (H) quantification of MitoTracker staining, (I) mitochondrial/nuclear DNA ratio, (J) glycolytic rate measurement (glycoPER) - profile in the left panel and relative parameters in the right panel and (K) 2-NBDG uptake in transfected Huh7 cells (n = 3-9 per group). Data is represented as mean±SD fold change to respective control when indicated. ∗*p <*0.05, ∗∗*p <*0.01, ∗∗∗*p <*0.001, ∗∗∗∗*p <*0.0001. Unpaired t-test with Welch’s correction or one-way ANOVA with Holm-Sidak’s correction. GlycoPER, glycolytic proton efflux rate; OCR, oxygen consumption rate.

To better delineate the molecular mechanisms through which miR-149-5p impacts lipid homeostasis in hepatic cells, we overexpressed miR-149-5p in Huh7 cells (Fig. 5A). As shown in Fig. 5B, C, BODIPY staining of lipid droplets indicated increased lipid accumulation in Huh7 cells overexpressing miR-149-5p in basal conditions. Western blot analyses of insulin-induced AKT-serine phosphorylation showed that miR-149-5p-induced steatosis did not impair insulin signaling (Fig. 5D).

SeaHorse analyses of Huh7 cells showed that miR-149-5p promotes an increased dependence of mitochondrial activity on fatty acids (Fig. 5E, etomoxir-treated cells), while decreasing mitochondrial oxidative phosphorylation (Fig. 5F) and mitochondrial ATP production (Fig. 5G). A quantitative increase in the mitochondrial mass was observed by both MitoTracker staining (Fig. 5H) and mitochondrial/nuclear DNA ratio (Fig. 5I) in Huh7 cells overexpressing miR-149-5p. Increased glycolytic rates in miR-149-5p-overexpressing Huh7 cells were also observed (Fig. 5J), despite no significant increase in glucose uptake (Fig. 5K) in these cells.

### MiR-149-5p alters the translation of several genes involved in metabolic and inflammatory pathways

**Fig. 6:**
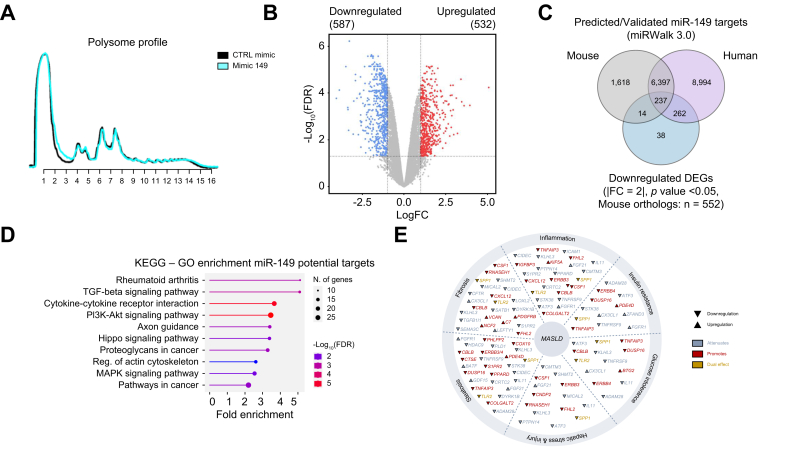
MiR-149-5p overexpression impairs a wide network of genes involved in metabolic/inflammatory/fibrotic processes. (A) Representative polysomes profiles and (B) deregulated genes identified in RNAseq of polysomal fractions of transfected Huh7 cells (n = 3 per group). (C) Venn diagram representing identified downregulated DEGs crossed with predicted/validated targets in both human and mouse genomes. ORAs by KEGG pathways of (D) shared targets (human and mouse) and of exclusively human targets. (E) Graphical representation of shared targets with reported implications in pathological mechanisms driving MASLD development/progression. Genes in light blue or red indicate an attenuation or promotion, respectively, while genes in yellow indicate a dual effect on the indicated process. ▲ upregulated; ▼ downregulated. DEGs, differentially expressed genes; MASLD, metabolic dysfunction-associated steatotic liver disease; ORAs, over-representation analyses.

As miRNAs modulate target gene expression by translational blockage or transcript degradation, we performed polysome fractionation and RNA sequencing of highly translated mRNAs in Huh7 cells overexpressing miR-149-5p or not, to identify miR-149-5p targets potentially contributing to MASLD. Despite no major alterations in polysome profile (Fig. 6A), we identified 587 downregulated and 532 upregulated genes in polysomal fractions of cells overexpressing miR-149-5p (Fig. 6B). Over-representation analyses based on KEGG pathways indicated that downregulated genes are part of key signaling pathways involved in cell metabolism, inflammatory processes and fibrosis (*e.g*. PI3K-Akt, calcium signaling, cytokine-cytokine receptor, TGF-Beta, *etc*. Fig. S12). Of note, upregulated genes were mostly regulating metabolic/inflammatory processes and detoxification mechanisms (*e.g.* retinol metabolism, complement and coagulation cascades, cell adhesion, drug metabolism, etc. Fig. S12).

The 587 potential targets found significantly downregulated were then cross-referenced with predicted/validated mRNA targets of miR-149-5p (human and mouse). Strikingly, 549 of the 587 downregulated transcripts were identified as potential targets of miR-149-5p in mice and/or humans (237 targets common between mice and humans). Gene enrichment analysis with the 237 common targets further confirmed that potential miR-149-5p target mRNAs were involved in metabolic/inflammatory/fibrotic processes (Fig. 6D). To highlight the relevance of these potential miR-149-5p targeted genes in MASLD development, we performed an extensive literature screening. Our screening indicated that 63 out of 237 were previously reported to play a significant role in different pathological processes associated with MASLD development/progression (Table S1, Fig. 6E). Among these 63 candidates, downregulation of 23 of them was previously reported to foster specific processes triggering MASLD (red and yellow genes in Fig. 6E). Of note, downregulation of 174 of the 237 predicted/validated miR-149-5p targets in mice/humans listed in Table S1 may have a significant impact on MASLD development and progression, but they have not yet been characterized in this pathological context.

## Discussion

Translational deregulation of gene expression by miRNAs is a key pathological mechanism contributing to chronic metabolic diseases such as MASLD.^6^ Herein, we identify miR-149-5p, which is strongly upregulated in hepatocytes with MASLD and fosters disease progression. Our data showed that miR-149-5p upregulation affects the expression of multiple cellular factors involved in the regulation of glucose and lipid metabolism, mitochondrial function, inflammation and tissue repair. Notably, the impact of miR-149-5p expression on steatosis, inflammation and fibrosis, that we observed in 2D cell culture and/or mouse models, was also confirmed in multi-lineage 3D HLOs, a newly established experimental model highly relevant for the human pathology.^28^^,^^30^ Based on publicly available GEO datasets of miRNA expression in humans with MASLD/MASH it is unclear if hepatic miR-149 is upregulated with MASLD/MASH (Fig. S13A).

The miR-149 genomic region contains 45 single nucleotide polymorphisms, among which two (rs2292832 and rs7142843, with 40% and 20% mean incidence, respectively, in the global population) were reported to have an effect on miR-149 maturation and to affect development and progression of several pathological conditions, including hepatic steatosis and hepatocellular carcinoma (HCC) (Fig. S13B-C, Table S2).^16^^,^^31^ Nevertheless, the prevalence of these polymorphisms in patients with MASLD and their correlation with MASLD severity needs to be further investigated. Despite our *in vitro/in vivo* results indicating that miR-149 modulates fuel utilization in hepatic energetic metabolism and promotes lipid accumulation in hepatocytes, experimental models such as immortalized/cancerous hepatic cell lines or genetic/diet-induced mouse models of MASLD have limitations, which must be considered when interpreting the data and extrapolating them to the human pathology.

Hepatic cell lines are highly proliferative, have multiple mutations, genomic and metabolic alterations, and are exempt from the influence of other cells in the hepatic microenvironment.^32^^,^^33^ Conversely, variability between *in vivo* models, differences between mouse and human pathophysiology (*e.g.* genetic, diets and metabolism, strain and sex, miRNA targets) must be considered when drawing conclusions about miRNA-associated pathological mechanisms.^34^ Considering these issues, the development of multi-lineage stem cell-derived HLOs has gained a lot of interest for the study of MASLD-associated pathological mechanisms.^35^^,^^36^ These HLOs exhibited transcriptomic profiles and metabolic activities similar to the human liver, and developed steatosis, inflammation and fibrosis upon pathological stimuli.^30^ In our study, *in vivo* and *in vitro* experiments were further supported by the results obtained with miR-149-5p upregulation in HLOs, thus highlighting miR-149-5p’s relevance for human pathology. Indeed, miR-149-5p overexpression in HLOs led to increased lipid accumulation in hepatocytes and upregulation of inflammatory/fibrotic markers, an inverted phenotype compared to what we observed in MASLD mouse models, in which miR-149-5p was silenced. Currently, HLOs’ application in translational research investigating MASLD development is limited by the high heterogeneity between different HLO batches, incomplete maturation of cells, as well as by the difficulties in genetic manipulation of HLOs. Further optimization and standardization of HLO production and genetic engineering in the future should provide an invaluable experimental model for human liver pathophysiology, as a complement to, or even partial replacement of animal experimentation.

miR-149-5p expression appears to be regulated differently in AT and muscles in mice with diet-induced obesity, as well as being sensitive to nutritional cues and physical activity. Consistent with what we and others observed in the liver,^11^^,^^37^ miR-149-5p regulated lipid metabolism in bovine adipocytes.^18^^,^^19^ Of note, miR-149-3p was also upregulated in the AT of mice fed a MASLD-inducing diet which promoted IR and adipogenesis in part by targeting PRDM16.^13^ In contrast, miR-149-5p was downregulated in skeletal muscle upon diet-induced obesity in mice, thus contributing to reduced mitochondrial biogenesis and IR partially due to PARP-2 upregulation.^14^ Several factors can explain the tissue-specific regulation of genes, the expression of different miR-149 strands and their impact on organs or whole-body homeostasis (*e.g.* diet type/duration, mouse strains, *etc*.).^6^^,^^7^^,^^38^^,^^39^ Regarding the PRDM16 and PARP-2 miR-149 targets previously identified in AT and muscles, respectively,^13^^,^^14^ we did not detect changes in their expression in our translatomic analysis, again suggesting a context-dependent regulation of miR-149 targets.^38^ However, given the numerous deregulated factors identified in our analyses as potential miR-149-5p targets, it is likely that the metabolic impact of miR-149-5p upregulation in hepatocytes is driven by fine modulation of multiple metabolic regulators rather than one single target.

Chen *et al.* recently reported hepatic miR-149 downregulation in diet-induced obesity in mice and that administration of miR-149 agomiRs led to an attenuation of hepatic steatosis and injury.^40^ These discrepancies likely stem from differences in experimental design and methods. First, the 4-week high fat-containing diet administered in mice by Chen and colleagues^40^ induced obesity, hepatic steatosis and IR but with no progression towards liver inflammation and fibrosis. In contrast, the FPC diet used in our study did not promote a significant weight gain in mice, but it led to massive steatosis and IR, as well as mild inflammation and fibrosis. The MCD diet induced significant weight loss concomitantly with steatosis, inflammation and fibrosis, but not insulin resistance. The systemic effects induced by the different diets may also have an impact on hepatic miR-149-5p expression/activity and associated metabolic disorders through inter-organ crosstalk mechanisms. While we injected hepatotropic adeno-associated virus 8 to specifically knockdown miR-149-5p in hepatocytes, Chen *et al.* injected miR-149 agomiRs systemically through the tail vein.^40^ Administration of synthetic nucleotides in mice was shown to trigger off-target effects due to the lack of cell/tissue specificity,^41^ as previously reported for miR-144-3p agomiRs injected in a model of diet-induced atherosclerosis^42^ or for miR-24 and miR-122 agomiRs that were detected in the liver, small intestine, aorta and muscle after tail injection.^43^ It is also well established that modulation of miRNA expression/activity by genetic engineering or administration of synthetic pharmacological nucleotides (*e.g.* agomiRs or antagomiRs) can yield different, or even opposite, biological outcomes in mice, as supported by previous studies investigating the role of miR-21 in cardiac diseases,^44^^,^^45^ or of miR-22 in diet-induced hepatic steatosis.^46^^,^^47^

Links between miR-149 expression/activity and inflammatory diseases were previously reported,^27^^,^[48], [49], [50], [51] but how miR-149 impacts hepatic inflammatory processes remain unclear. In patients with chronic hepatitis C virus infection, hepatic miR-149-3p was shown to be strongly upregulated,^52^ while its expression decreased in a mouse model of carbon tetrachloride-induced hepatic injury.^53^ miR-149 was further demonstrated to upregulate pro-inflammatory signaling mediated by STAT3, NF-kB, AMPK, MAPK and NFATc4,^27^ leading to changes in immune cell activity^50^ and expression of pro-inflammatory cytokines such as *Cxcl10, Il1b, Il6, Ccl2/5, Il8* and *Tnfa*.^27^^,^^51^ In contrast, genetic deficiency of both miR-149 strands in mice (MIR149KO mice) promoted LPS- and acute diethylnitrosamine-induced hepatic injury, by increasing STAT3 signaling, pro-inflammatory cytokine expression and hepatocyte death.^21^^,^^49^ Administration of miR-149-3p/5p agomiRs in MIR149KO mice partially alleviated LPS-induced hepatic inflammation.^49^ In our studies, inhibition of miR-149-5p *in vivo* triggered a mild reduction of inflammatory markers in FPC-fed mice, while this fine-tuned regulation was likely masked by the strong effect of the MCD on hepatic and whole-organism homeostasis. However, overexpression of miR-149-5p in HLOs significantly increased the expression of specific inflammatory cytokines (*i.e*. *IL6, IL1B*) and our translatomic analyses of Huh7 also showed a significant negative impact of miR-149-5p overexpression on factors governing inflammatory pathways. Again, the different experimental settings and the context-dependent function of the different miR-149 strands could be responsible for these divergent data in mice. Of note, MIR149KO mice have a deletion of both miR-149 strands in all cells. It is not possible to assess the role of different miR-149 strands in specific cell types (*e.g.* hepatic stellate cells, immune cells or hepatocytes). It is also unclear whether miR-149-3p and miR-149-5p target different sets of genes, and if the different strands have opposing or synergistic effects on inflammatory processes. Additionally, potential adaptive mechanisms occurring during embryogenesis and development to circumvent loss of miR-149 further complicate the interpretation of data obtained with total knockout mice.

Finally, regarding the impact of miR-149-5p in fibrosis development, what we observed in mice, HLOs and translatomic data is consistent with previous reports indicating that miR-149 overexpression affects TGF-β signaling,^50^ and activates LX2 human cultured stellate cells *in vitro*.^53^ The mild reduction in inflammation and fibrosis that we observed in FPC-fed mice following miR-149-5p downregulation could also derive from the improved steatosis. Indeed, pathological accumulation of specific lipid species (*e.g.* lipid peroxides, acylceramides, diacylglycerol, palmitate, *etc*.) can strongly promote lipotoxicity and inflammatory responses.^54^ Future investigations are required to experimentally validate if miR-149-5p drives inflammation and fibrosis indirectly through alterations of the glucose/lipid metabolism and/or also by targeting important cellular factors restraining inflammation and fibrosis in other cell types.

The impact of one specific miRNA in each cell is complex and does not depend solely on its expression. There is a delicate equilibrium established between miRNAs, and/or RNA-binding proteins, competing for overlapping seed sequences on the same mRNA 3’untranslated region.^7^^,^^55^ miRNA activities might also be tightly regulated independently of their expression as shown previously for miR-21 in hepatocytes.^56^^,^^57^ Finally, miRNAs can also be secreted or sequestered by other molecules such as pseudogenes or other non-coding RNAs.[58], [59], [60] All these processes are highly dependent on the cellular and organ context, as well as external pathophysiological stimuli.^53^^,^^56^ Based on our current knowledge of miRNA biology, it is inaccurate to attribute the pathophysiological outcomes of miRNA deregulation to the modulation of a single target gene. Since miRNAs can either block the translation of several transcripts, or trigger their degradation without necessarily affecting gene transcription, we performed translatomic analyses to identify potential miR-149-5p targets. Not surprisingly, miR-149-5p overexpression in Huh7 cells promotes the downregulation of several transcripts potentially containing miR-149-5p-specific seed sequences. Whether all the identified downregulated transcripts are directly linked with miR-149-5p activity remains to be experimentally validated, which surpasses the scope of this study. Nevertheless, more than 60 of these mRNAs were previously reported to play a significant role in MASLD development, leading us to conclude that miR-149-5p in hepatocytes likely influences the expression of numerous critical metabolic/inflammatory factors, collectively contributing to aggravation of MASLD, as further supported by our functional analyses.

While miR-149-5p is upregulated in MASLD, a switch in its expression occurs with hepatic carcinogenesis and miR-149-5p becomes strongly downregulated in HCC.[61], [62], [63], [64] Considering the impact of miR-149-5p on the hepatic metabolism it is likely that expression of miR-149-5p targets contributes to carcinogenesis either by favoring metabolic switches in cancer cells (*e.g.* Warburg effect) or by promoting other cancer-related features. Here again, the drastic shift in miR-149-5p expression is likely to affect multiple mRNAs involved in these processes and future studies are required to understand how miR-149 downregulation contributes to HCC development. In this regard, the well-established target of miR-149-5p, *Fgf21*^15^^,^^17^ (also validated in this study – Fig. S14 and in translatomic data) is an interesting example, which contributes to MASLD development when it is downregulated and to HCC development when it is upregulated. FGF-21 expression or analogs counteract metabolic disorders, inflammation and fibrosis in the liver,[65], [66], [67] while FGF-21 can have oncogenic activity in various cancers.[68], [69], [70]

Based on our data, and those of others,^16^^,^^37^ elevated miR-149-5p levels in hepatocytes foster MASLD development by affecting whole-body energy metabolism and hepatic glucose/lipid metabolism, mitochondrial function, and the expression/activity of pro-inflammatory/pro-fibrogenic molecules. Downregulation of miR-149-5p in hepatocytes appears to be a potential therapeutic strategy for MASLD. Synthetic pharmacological miRNA inhibitors are currently being clinically evaluated to treat liver pathologies, *e.g*. miR-34 antagomiRs for HCC^71^ and miR-122 antagomiRs for HCV infection.^72^^,^^73^ However, as previously discussed, prudence is needed before envisaging miR-149-5p inhibition as a therapeutic approach for MASLD. In particular, it is required to better understand the respective role of each miR-149 strand in the different hepatic cells. Further research is also needed to achieve cell-specific targeting of synthetic pharmacological miRNA inhibitors or mimics in order to prevent off-target effects of these molecules.

## Abbreviations

AT, adipose tissue; AAV8, adeno-associated virus serotype 8; BAT, brown adipose tissue; ESC, embryonic stem cells; eWAT, epididymal white adipose tissue; FPC, fructose/palmitate/cholesterol-enriched; GEO, gene expression omnibus; HCC, hepatocellular carcinoma; HFD, high-fat diet; HLOs, human liver organoids; IPGTT, intraperitoneal glucose tolerance test; IR, insulin resistance; LPS, lipopolysaccharide; LPTENKO, liver-specific *Pten* knockout; MASLD, metabolic dysfunction-associated steatotic liver disease; MCD, methionine/choline-deficient diet; miRNA, microRNA; mRNA, messenger RNA; mWAT, mesenteric white adipose tissue; NAFLD, non-alcoholic fatty liver disease; OAPA, oleic:palmitic acid; shRNA, short-hairpin RNA; T2D, type 2 diabetes; TG, triglyceride.

## Financial support

This study was supported by the Ernest Boninchi Foundation, the Bo and Kerstin Hjelt Diabetes Foundation and by the Swiss National Science Foundation (grant numbers 310030-172862 and 320030-200530).

## Conflict of interest

The authors declare no conflict of interest.

Please refer to the accompanying ICMJE disclosure forms for further details.

## Authors’ contributions

Study concept and design – MCS, MGj, FB, MFot; Acquisition of data – MCS, MGj, ED, CM, MFou, FB; analysis and interpretation of data – MCS, MGj, ED, CM, MFot; drafting of the manuscript – MCS, MGj, MFot; critical revision of the manuscript – MCS, MGj, ED, FB, MFot; statistical analysis – MCS; funding acquisition – MGj and MFot; Study supervision – MGj and MFot.

## Data availability statement

Data presented in this manuscript will be publicly available on https://doi.org/10.26037/yareta:lcl2chfvereufnbarebilj5yyq.

## Declaration of Generative AI and AI-assisted technologies in the writing process

During the preparation of this work, the authors used ChatGPT in order to improve language and readability. After using this tool/service, the authors reviewed and edited the content as needed and take full responsibility for the content of the publication.
