# Supplementary material for: Hepatic miR-149-5p upregulation fosters steatosis, inflammation and fibrosis development in mice and in human liver organoids
Source: JHEP Rep. 2024 Jun 4;6(9):101126. doi: 10.1016/j.jhepr.2024.101126 (PMC11388170; doi:10.1016/j.jhepr.2024.101126)
Supplement: Multimedia component 1 [file mmc1.pdf]

Supplementary material to:

Hepatic miR-149-Sp upregulation fosters steatosis, inflammation and fibrosis development in mice and in human liver organoids

Marta Correia de Sousa, Etienne Delangre, Flavien Berthou, Sanae El Harane, Christine Maeder, Margot Fournier, Karl-Heinz Krause, Monika Gjorgjieva, Michelangelo Foti

Table of contents

Supplementary methods 2

Supplementary figures 19

Supplementary tables 33

Supplementary references 41

Supplementary methods

1.1. In vivo

1.1.1. Animal housing

Mice were adapted to the animal facility of University of Geneva for three weeks and kept in ventilated cages with 2 to 5 animals per cage. During the period of adaptation, mice had access to standard chow diet (SAFE-150 diet, SAFE, Augy, France) and water ad libitum. Animals were maintained in cages with appropriate enrichment (disposable house and nesting material) and with a 12h light/dark cycle at 23°C.

1.1.2. Adeno-associated virus injection

Animals were randomly allocated to the different experimental groups. Mice were injected retro-orbitally with adeno-associated virus serotype 8 (AAV8) packed with either scrambled shRNA ($2x10^{11}$ GC/mouse of AAV8-U6-scrambled-shRNA-GFP diluted in 0.9% NaCl, group shCTL, VectorBiolabs, USA) or with shRNA specific for microRNA miR-149 ($2x10^{11}$ GC/mouse of AAV8-U6-shRNA-miR-149-5p-GFP in 0.9% NaCl, group shmiR-149 VectorBiolabs, USA).

1.1.3. Diets and experimental procedures

Mice fed with a high sugar/high fat diet (HFD)

Two month old C57BL/6J mice (Charles Rivers Laboratory) were submitted to four different isocaloric high sugar/high-fat diet (HFD: 45% kcal from fat, 17% kcal from sucrose) or a matched Control Diet (CD: 10% kcal from fat, 17% kcal from sucrose) for 16 weeks (n=5 per group). The different HFD were a Western Diet (WD: made with lard), an omega-3 enriched HFD (O-3D - similar to the WD but with 25% of the total fat mass replaced by omega-3 fish fatty acids) and a trans-hydrogenated fatty acid enriched HFD (THD - similar to the WD but with 23%-26% of the total fat mass replaced by trans-hydrogenated monounsaturated fatty acids). Detailed information on the different HFD diets is described on Table S1.

Mice fed with a methionine/choline-deficient diet (MCD)

Ten weeks old mice injected with AAV8-shCTL or AAV8-shmiR-149 (n=7 per group) were allowed to recover for 10 days and then were fed with a methionine/choline deficient diet (MCD; E15653-94, ssniff, Germany) for 19 days. During this feeding

period, mice were weighted every two days. At the end of the experiment, mice were decapitated following isoflurane anesthesia and liver and blood samples collected for further analyses. Detailed information on the MCD diets is described on Table S1.

Mice fed with a fructose/palmitate/cholesterol/trans-fat-enriched diet (FPC diet)

Ten weeks old mice injected with AAV8-shCTL or AAV8-shmiR-149 were allowed to recover for 10 days and then were fed with a Fructose/Palmitate/Cholesterol/Trans-Fat-enriched diet (FPC, TD.19142, Envigo, USA) for 10 weeks (short FPC, n=8-12 per group) or 24 weeks (Long FPC, n=10-12 per group). For the 24 weeks protocol, mice received a second injection of adenoviruses ($1x10^{11}$ GC/mouse) at 8 weeks of diet in order to ensure hepatic knockdown of miR-149. The FPC diet was previously reported to induce liver steatosis after 8-10 weeks and liver steatosis, fibrosis and inflammation after 16-24 weeks (1). During the feeding periods, mice were weighted each week and glycemia was measured at 9 a.m. using a Glucometer (AccuCheck - Roche) on blood collected from the tail vein after 10 (n=16-18 per group) and 23 (n=9-11 per group) weeks of diet. Glucose and pyruvate tolerance tests were performed on mice at 7/14 weeks of diet and 8/22 weeks of diet, respectively. The same blood samples (fasted: n=8-12 per group at 7 weeks; fed: n=7-10 per group at 10 weeks, n=8-9 per group at 22 weeks) were used to measure insulinemia by ELISA (Mercodia Ultrasensitive Mouse Insulin ELISA – 10-1249-01). Detailed information on the FPC diets is described on Table S1.

1.1.4. Glucose and Pyruvate Tolerance Tests

For the glucose tolerance test, mice were fasted 6 hours prior the intraperitoneal injection of 2 g/kg of glucose (n=10-12 per group at 7 weeks, n=9-11 per group at 14 weeks). For the pyruvate tolerance test, mice were fasted 18 hours prior the intraperitoneal injection of 2 g/kg of pyruvate (Sigma)(n=8-11 per group at 8 weeks, n=9-11 per group at 22 weeks). Blood glucose levels were measured at 0, 15, 30, 60, 90 and 120 minutes post-injection of glucose/pyruvate using a Glucometer (AccuCheck – Roche) in blood samples collected from the tail vein.

1.1.5. Metabolic cages and EchoMRI

Metabolic phenotyping of FPC fed mice (23 weeks, n=6-7 per group) was performed for 7 days using metabolic cages (LabMaster) after 2 days of adaptation prior to recording calorimetric parameters (O_2 consumption, CO_2 production, respiratory exchange ratio

and energy expenditure), food and water intake and locomotor activity. During this procedure, mice were isolated in metabolic cages. Body composition was measured using a positron emission tomographic whole-body composition analyzer (EchoMRI-700, Houston, Texas, USA).

1.1.6. Plasma analyses

Blood recovered during sacrifice following decapitation was centrifuged at 5000 rpm (Centrifuge) for 10 minutes. Plasma was collected and glucose, aspartate/alanin-aminotransferases (ASAT/ALAT), cholesterol and triglycerides levels were analyzed using Cobas 8000 system (Roche, Switzerland).

1.2. In vitro

1.2.1. Isolation of primary hepatocyte

Primary mouse hepatocytes were isolated from LPTENKO mice and control littermates as previously described (2, 3).

1.2.2. Cell lines

Huh7 cells were purchased from Sekisui Genotech (JCRB0403, Japan) and cultured in DMEM (1g/L glucose, Gibco) supplemented with 1% penicillin-streptomycin (PS, Gibco) and 10% fetal bovine serum (FBS, Gibco).

1.2.3. Human Liver Organoids

Human Liver Organoids (HLOs) were generated from human embryonic stem cells (ESC) (HS420, BAG-hES-IMP-0046, Karolinska Institute, Stockholm, Sweden) and cultured as previously described by Ouchi et al., 2019 with slight modifications (4). Briefly, HS420 were cultured in 35mm-dishes coated with 0.5µg/cm^2 laminin iMatrix-521 silk (A29248, Gibco) at 37°C in 5% CO_2. Upon 70%-80% confluency, cells were detached with Accutase (Gibco), washed with Wash medium (DMEM/F12 supplemented with 1% PS, Gibco) and centrifuged for 3 minutes at 300 x g at room temperature. The cell pellet was resuspended in mTeSR1 supplemented with 1% PS (Gibco) and 10 µM of Rock inhibitor (Y27632, Tocris) and 500’000 cells/mL were seeded in laminin coated 35mm-dishes following Trypan Blue Exclusion Test. Following 24 hours, the Rock inhibitor was removed from the medium. To induce definitive endoderm differentiation, when cells reached 85-90% confluence medium was changed to RPMI 1640 medium (61870010, Gibco) supplemented with 1% PS (Gibco), 50 ng/mL bone morphogenetic protein 4 (BMP4, 314-BP-010, R&D Biotechne) and 100

ng/mL Activin A (338-AC-050, R&D Biotechne). At day 2, RPMI 1640 was supplemented with 100 ng/mL Activin A (338-AC-050, R&D Biotechne) and 0.2% KSR (A3181502, Gibco) and at day 3 with 100 ng/mL Activin A (338-AC-050, R&D Biotechne) and 2% KSR (A3181502, Gibco). From day 4 to day 6, the medium was changed to Advanced DMEM/F12 (12634010, Gibco) supplemented with 0.1 mM Hepes, GlutaMax (35050038, Gibco), 1x B27 (17504044, Gibco), 1x N2 (17502048, Gibco), 1x gentamycin/amphotericin (R01510, Gibco), 500 ng/mL fibroblast growth factor 4 (FGF4, PeproTech) and 3µM CHIR99021 (130-106-539, Miltenyi), and replaced daily. At the end of 6th day, spheroids and associated cells were gently pipetted to release them from the dish. Subsequently, they were washed with washing medium and centrifuged for 3 minutes at room temperature and 300 x g to prepare them for embedding in 100% Matrigel. Matrigel drops containing spheroids and cells were plated in 35-mm dishes and Advanced DMEM/F12 with B27, N2 medium was further supplemented with 2µM retinoic acid (RA, Sigma). Following 4 days of RA treatment, medium was changed to Hepatocyte Culture Medium (HCM, Lonza) supplemented with 10 ng/mL hepatocyte growth factor (HGF, PeproTech), 100 nM Dexamethasone (Sigma) and 20 ng/mL Oncostatin M (300-10, Peprotech) and replaced every 3 days. At day 17, Matrigel drops were disrupted with gentle pipetting to release the HLOs and they were kept in suspension in complete HCM medium supplemented with 10% Matrigel (Gibco) until the end of the experiment (day 21, day 24 or day 28). Differentiation state of each preparation was assessed through quantitative expression (RT-qPCR) of cell markers: stem-cells (*NANOG*), mid-gut (*CDX2*), foregut (*FOXA2*), hepatocytes (*HNF4, ALB, SERPINA1*), stellate cell (*ALCAM*) and Kupffer cells (*CD68*). Ethical authorization to use ESC HS420 cells was provided by the Geneva Health Head Office (authorization number R-FP-S-2-0028) and performed following the Swiss guidelines on Research involving embryonic stem cells.

1.2.4. Synthetic Oligonucleotide transfections

Huh7 cells were transfected 24 hours after seeding using Interferin (Polyplus transfection, Illkirch, France) and Optimem (Gibco), following manufacturer's instructions. HLOs were transfected using Lipofectamine (ThermoFisher Scientific) and Optimem (Gibco), following manufacturer's instructions. HLOs were kept in DMEM (1g/L glucose, Gibco) supplemented with 1% PS for 24 hours and then the medium was changed to HCM supplemented as described in 2.2.2. until the end of the experiment. Huh7 cells and HLOs were transfected with miRIDIAN microRNA miR- 149-5p mimic

(Mimic 149, Horizon Discovery, UK) or miRIDIAN microRNA Mimic Negative Control #1 coupled to a fluorophore or not (CTRL Mimic/ CTRL Mimic FL, Horizon Discovery, UK) at concentrations of 10 nM and 25 nM for Huh7 cells and HLOs respectively.

1.2.5. SeaHorse analyses

Twenty-four hours after transfection of miRIDIAN microRNAs, Huh7 cells were re-seeded in a 96-well Seahorse Agilent Plate at 25'000 cells per well. Twenty-four hours post-reseeding, different Seahorse XF metabolic assays were performed in a Seahorse XFe96 Analyzer according to the manufacturer's recommendations – MitoStress, GlycoRate and Substrate Oxidation Test. At the end of each assay, cells were fixed with 4% paraformaldehyde for 15 minutes at room temperature, stained with Hoechst (1 µg/mL, 33342, ThermoFisher Scientific) for 10 minutes and scanned on Cytation 5. Following image acquisition, cell number was counted using the Gen5 software (BioTek) and used to normalize Seahorse assays results using Wave 2.4.0. software (Agilent Technologies).

1.2.5.1. MitoStress Test

Seahorse XF MitoStress Test (MitoStress 103015-100 kit) was performed as described by the manufacturer, using the following drug concentrations: Oligomycin (Oligo) – 1.5 µM, FCCP – 2 µM and Rotenone/Antimycin A (Rot/AA) – 0.5 µM.

1.2.5.2. GlycoRate Test

Seahorse XF Glycolytic Rate assay (GlycoRate 103344-100 kit, Agilent) was performed as described by the manufacturer, using the following drug concentrations: Rotenone/Antimycin A – 0.5 µM, 2-deoxy-D-glucose (2-DG) – 50 mM.

1.2.5.3. Substrate Oxidation Stress Test

Seahorse XF Substrate Oxidation Stress (Long Chain Fatty Acid 103672-100 kit) was performed as described by the manufacturer, using the following drug concentrations: Etomoxir (E) – 4 µM, Oligo – 1.5 µM, FCCP – 2 µM and Rot/AA – 0.5 µM. Conditions were etomoxir was not injected are identified as medium only (M).

1.2.6. Glucose uptake

Cells were seeded and transfected as described in 1.2.2 and in 1.2.4. Cells were then starved (glucose/FBS-free medium) for 3 hours and incubated with 2-(N-(7-Nitrobenz-2-oxa-1,3-diazol-4-yl)Amino)-2-Deoxyglucose (2-NBGD, N13195, ThermoFisher

Scientific) for 30 minutes. Following incubation, cells were fixed with 4% paraformaldehyde for 15 minutes at room temperature and counter-stained with Hoechst (1μg/mL, 33342, ThermoFisher Scientific) for 10 minutes. Coverslips were mounted using anti-fading agent (DAKO, S3023, Agilent) and imaged with Axiocam Fluo (Zeiss). Relative signal intensity of 2-NBDG was measured using CellProfiler v4.2.1. software and normalized to cell number.

1.2.7. Insulin stimulation

Cells were seeded and transfected as described in 1.2.2 and in 1.2.4. Cells were grown until 70% confluence, starved (FBS-free medium) for 6 hours and stimulated with insulin (Mixtard, 10^{-7}M) for 15 minutes or not. Following stimulation, cells were flash frozen using liquid nitrogen.

1.2.8. Morphological assessment of lipid droplets accumulation in hepatocytes (steatosis)

Huh7 cells and HLOs were cultured and transfected as described in 1.2.2 - 1.2.4. Following 24-hours post-transfection, Huh7 cells and HLOs were exposed to fatty acid (FA)-enriched medium for 48 hours/ 7 days (Oleate (OA) 100 μM, Oleate:Palmitate (OA:PA) 400:200 μM or OA:PA 100:50 μM) or not (basal conditions). Following incubation with FA-enriched medium, Huh7 cells and HLOs were fixed with 4% paraformaldehyde at room temperature for 15 minutes (Cells) or 2 hours (HLOs), stained for neutral lipids with BODIPY (1 μg/mL, D3922, Molecular probes), counter-stained with Hoechst (1μg/mL, 33342, ThermoFisher Scientific) for 10-20 minutes and imaged with Axiocam Fluo (Zeiss) or with Nikon A1r Spectral (Nikon).

1.2.9. Establishment of inflammation/ fibrosis in human liver organoids

HLOs were cultured as described in 1.2.2. At day 21 HLOs were exposed to a mixture of cytokines (TNFα, TGFβ, IL-6 and IL-1β, 10ng/mL each) for 3 days or to FA-enriched medium for 7 days (as described in 1.2.8.). Following incubation with cytokines/FAs, HLOs were collected and expression of inflammatory/fibrotic markers was assessed through RT-qPCR.

1.2.10. Determination of the cellular mitochondrial mass

Huh7 cells were cultured and transfected as described in 1.2.2 and in 1.2.4. To observe mitochondrial morphology and quantify mitochondrial mass, cells were stained at 72-

hours post-transfection with MitoTracker™ Red CMXRos (200 nM, M7512, ThermoScientific) for 20 minutes at 37°C. Subsequently, cells were fixed with 4% paraformaldehyde for 15 minutes at room temperature and counter-stained with Hoechst (1µg/mL, 33342, ThermoFisher Scientific) for 10 minutes. Coverslips were mounted using anti-fading agent (DAKO, S3023, Agilent) and imaged with Axiocam Fluo (Zeiss). In parallel, mitochondrial DNA was purified using the QiAmp ® DNA Micro kit (56304, Qiagen). Then, amplification of nuclear and mitochondrial DNA was performed and the mitochondrial/nuclear DNA ratio was calculated as previously described (5).

1.3. Polysome fractionation

Huh7 cells were cultured and transfected as described in 1.2.2 and in 1.2.4. and collected while in proliferative stage (70-80% confluency). Cycloheximide (CHX, 100 µg/ml) was added to the medium and cells were incubated at 37°C for 10 min in a humidified incubator. Plates were washed with 10 ml ice-cold 1xPBS (calcium and magnesium-free, Gibco) supplemented with CHX. Cells were collected using 0.25% trypsin supplemented with CHX for 10 min and centrifuged at 800 rpm, 4°C for 5 min. Cells were then lysed in 2 volumes of lysis buffer (20 mM Tris, pH 7.4, 140 mM KCl, 5 mM $MgCl2$, 1.0% Triton X-100, 1 mg/ml Heparine, 25 U/ml Turbo DNase I, 1mM DTT, 100 µg/ml CHX, Protease inhibitors (Roche), 0.25 U/ml SUPERaseIn RNase inhibitor (Ambion, #AM2694)) and by passing 10-12 times through 25G needle. Extracts were centrifuged at 20000 g, 4°C for 20 min. Cell lysates were loaded on linear 20-60% sucrose gradients prepared with gradient buffer (20 mM Tris, pH 7.4; 140 mM KCl; 5 mM MgCl2; 1 mM DTT; 0.1 mg/ml CHX). Ribosomes were fractionated at 247’600 g (38’000 rpm, rotor SW41 Ti (Beckman Coulter, #331362) for 3 h 30 min at 4°C. Fractionated ribosomes were monitored and collected using Density Gradient Fractionation System (ISCO).

1.4. RNA extraction and Real-time qPCR

RNA extraction from ribosomal fractions, flash frozen cells, HLOs and mouse tissues was performed using Trizol (Ambion, Thermo Scientific, USA) according to manufacter’s instructions. RNA concentration was measured using NanoDrop (Thermo Scientific). Prior to real-time qPCR, reverse transcription was performed using the High-Capacity cDNA Reverse Transcription kit (Applied Biosystems™). For miRNAs expression, the reverse transcription was performed as previously described (6). qPCR

was performed using the PowerUp™ SYBR™ Green Master Mix for Real-Time PCR and the QuantStudio 5 Real-time PCR System and data analysis software (Applied Biosystems™), according to manufacturer's specifications. Primer sequences used are described in *Table S2*. Gene expression was quantified using the ΔΔCT method.

1.5. Western Blot

Protein extraction from flash frozen cells and mouse tissues was performed using RIPA buffer (50 mM Tris-HCl, pH 6.8, 100 mM DTT, 2%SDS, 0.1% bromophenol blue, 10% glycerol). Tissues were homogenized using TissueLyser. Protein lysates were centrifuged at 12000 g for 10 minutes and the supernatant collected. Protein concentration was determined using BCA protein assay kit (Pierce Biotechnology). 5-10 µg of protein was charged in 5-20% gradient sodium dodecyl sulfate-polyacrylamide gel electrophoresis (SDS-PAGE) gels and transferred to nitrocellulose membranes (RPN303D, Amersham, Switzerland). Membranes were blocked with polyvinyl alcohol and incubated overnight with primary antibody at 4°C. Membranes were washed with 0.1% TBS-Tween and incubated with secondary antibodies at room temperature for 1 hour. Membranes were then incubated with ECL Prime Substrate (RPN22232, Amersham, Switzerland) for 1 minute and revealed using the PXI/PXI Touch system (Syngene, Synoptics group, UK) or Fusion instrument (Vilber, France). Between each step, membranes were washed with 0.1% TBS-Tween. Signal was quantified using GeneSys (Syngene, Synoptics group, UK) or ImageJ™ software. Antibodies used are described in *Table S2*.

1.6. Histology, Immunohistochemistry and immunofluorescence

Mouse tissues were fixed overnight with 4% paraformaldehyde (PFA) while HLOs were fixed for 2 hours in 4% PFA and then transferred to PBS for dehydration and paraffin-embedding. Tissue and HLOs specimens were cut into 5 µm sections prior to staining or immunohistochemistry/immunofluorescence. For morphological analysis, tissue sections from 3 samples collected from different lobes of the explanted liver were stained with hematoxylin and eosin while for fibrosis analysis, sections were stained with Sirius Red. For immunohistochemistry and immunofluorescence, tissue/HLOs sections were deparaffinized, rehydrated and heated in citrate buffer or treated with Proteinase K for antigen retrieval. Subsequently, sections were permeabilized with

0.3% Triton X-100 in TBS for 15 minutes, blocked with 10% goat serum (ab138478, Abcam) in 1% BSA-TBS for 2h at room temperature and incubated overnight at 4°C with primary antibody diluted in 1% BSA-TBS. Endogenous peroxidase was blocked by incubation with 0.3% H_2O_2 solution for 15 minutes at room temperature, prior to incubation with secondary antibody at room temperature for 1 hour. Between each step, slides were washed in 0.025% Triton X-100 in TBS. Signal was revealed following an incubation with DAB Substrate kit (ab64238, Abcam, UK) or with secondary antibodies conjugated with a fluorophore and counterstained with hematoxylin for 3 minutes or with Hoechst (1 µg/mL, 33342, ThermoFisher Scientific) for 10 minutes. Following staining or immunohistochemistry/immunofluorescence, slides were mounted and scanned in AxioScan Z.1 (Zeiss) or imaged in Axiocam Fluo (Zeiss) for subsequent analysis. The slides from the different experimental groups were then analyzed blindly by two different researchers. Antibodies and concentrations used are listed in *Table S2*.

1.7. Image analysis

Batched analysis and quantification of lipid droplet number/size per µm^2 of tissue were performed using QuPath software (version 0.4.3.) and CellPose package (version 2.0)(6) following pretrained automated tissue/lipid droplet detection. Sirius red positive areas per µm^2 of tissue were detected using QuPath's pixel classifier default settings following pretrained automated tissue detection.

Relative BODIPY and MitoTracker™ Red CMXRos signal in Huh7 cells was measured with CellProfiler v4.2.1. and normalized to cell number. Single lipid droplets morphological aspects in Huh7 cells were also analyzed with CellProfiler (number, area, diameter).

For HLO image analysis, 3D confocal stacks, acquired with multiple wavelengths, were automatically processed using a dedicated framework developed in Matlab R2023a (The MathWorks). Specifically, Nikon nd2 files were accessed through the Bio-formats package (7). Organoid localization was achieved by the maximum voxel-wise of all available channels following intensity normalization. Subsequently, alternating sequential filters and Otsu's method (8) for global image segmentation were employed. Nuclei, labeled with DAPI and/or HNF4, and lipid droplets were segmented following anisotropic diffusion filtering using Cellpose 2.0 (6) in 3D, with its pre-trained models for "nuclei" or "cyto". The resulting two sets of nuclei were combined to identify hepatocyte

nuclei. Lipid droplets were assigned to specific cell nuclei based on the shortest Euclidean distance centroid to centroid. Finally, the results were quantified in terms of organoid volume, hepatocyte cell-proportions in the organoids, total lipid droplet volume, and mean fluorescent intensities in HNF4-positive or -negative nuclei.

1.8. Glycogen, glucose and glucose-6-phosphate content assay

Frozen liver tissues (100 mg) from mice fed FPC diet for 24 weeks (n=9-10 per group) were lysed in 8 volumes of perchloric acid (6%). Lysates were centrifuged at 10'000 g for 15 min at 4°C. Supernatants were collected and neutralized with potassium carbonate (K_2CO_3, 3.2mM) until pH reached 6.5-8.5. Following another centrifugation at 10 000 g for 15 min at 4°C, glycogen content was measured with Keppler and Decker method, as previously described (7). Glycogen was partially hydrolyzed in NaOH (0.15 M) for 20 minutes at 100ºC and digested by α-amidoglucosidase for 1h at 45°C into glucose. Glucose was measured after the addition of hexokinase (0.7U/mL) and glucose-6-phosphate was measured after the addition of $NADP^+$(0.9 mM) and glucose-6-phosphate dehydrogenase (0.7U/mL). NADPH production was detected at 340 nm.

1.9. Triglyceride assay

Intra-hepatic triglycerides were measured using the Triglyceride-Glo™ assay (Promega) according to manufacturer's instructions. The results obtained for the FPC diet were further validated using the <Folch= method as described in (9).

1.10. Microarray analysis

Livers from 4 months old LPTENKO (*Pten*$^{lox/lox}$, AlbCre$^{+/-}$) mice and wild-type littermates (*Pten*$^{lox/lox}$, AlbCre$^{-/-}$; n=3 per group) were used for miRNAs microarray analysis. RNA was extracted as mentioned in section 1.4. and 500 ng of total RNA was used for subsequent analyses. Microarray miRNA expression profiles of were performed in the Genomics platform at University of Geneva. miRNA expression profiles were obtained using the Affymetrix GeneChip ® miRNA 3.0 Array (Affymetrix). After quality control, data was normalized and summarized using the robust multichip analysis (Affymetrix Microarray Suite). Partek was used to determine ANOVA p-values and fold-changes. Only miRNAs respecting the established threshold (p-value<0.05, !fold-change (FC)! > 1.5) were considered for analyses.

1.11. RNA sequencing

RNA extracted from ribosomal fractions (polysome fractions only) of Huh7 cells overexpressing miR-149 (Mimic 149) or scrambled synthetic oligonucleotides (Control Mimic) was used to perform high-throughput sequencing. Poly-A selected RNA was used for library preparation using TruSeq RNA Sample preparation kit (Illumina) andsubmitted to 100 nt single-end read TruSeq HT stranded mRNA sequencing protocol in an Illumina NovaSeq 6000 system, according to manufacturer's indications. Reads were processed with FastQC v.0.11.9, STAR v.2.7.4a, PicardTools v2.21.6, and HTSeq v0.9.1 for biological quality control, mapping, alignment and preparation of table of counts. Normalization and differential expression analysis were performed using the edgeR v3.38.4 R package. Data was normalized by sequencing depth and RNA composition and differentially expressed genes (DEGs) were estimated using the negative binomial general model. Subsequently, DEGs between conditions were identified after establishment of thresholds for statistical significance and magnitude of change (fold change (FC)=!2! and false discovery rate (FDR)<0.05).

1.12. In silico analysis

1.12.1. Over Representation Analysis

Over Representation Analysis (ORA) was performed using clusterProfiler v4.9.0 and edgeR package. Deregulated genes were identified using the following thresholds: p-value with a false discovery rate (FDR, Benjamini & Hochberg) <0.05, fold-change to Control Mimic condition = !1.5! and classified based on gene ontology by biological processes (GO:BP) and by KEGG pathways.

1.12.2. Identification of potential targets and related biological functions

Predicted and validated targets of miR-149 in human and mouse genomes were retrieved using the miRWalk database (http://mirwalk.um.uni-heidelberg.de/, accessed on 15/06/2023). This list was cross-referenced with DEGs identified in the RNA seq analysis after converting gene symbols to mouse orthologs using g:Orth on g:profiler (https://biit.cs.ut.ee/gprofiler/orth). Potential downregulated targets shared between human and mouse or exclusively human were considered for GO analysis by KEGG pathways using shinyGOv0.77 application (http://bioinformatics.sdstate.edu/go/, accessed on 10/08/2023). Shared targets were screened in PubMed to verify functional characterization of each gene in the context of MASLD development. The literature

screening was performed using the gene name and the following terms: <Obesity=, <Diabetes=, <Insulin Resistance=, <Steatosis=, <Inflammation=, <Fibrosis=, <Liver=, <NAFLD=, <MASLD=, <NASH= and <MASH=. Only studies using in vivo models were considered.

1.13. Statistical analysis

For animal experimentation we have performed power analysis (G*Power software, v. 3.1.9.7, p=0.1 (t-test), effect size 1.1, power: 0.9) to calculate the minimum number of animals necessary to observe an expected effect of 40% reduction in regards to steatosis between shCTL and shmiR149 mice. This analysis allowed to determine a minimum of 12 mice per group. Statistical analyses were performed using GraphPad Prism 8 Software (GraphPad Software, San Diego, CA, USA). Results are represented as mean ± standard deviation (SD). Outliers test was performed using the ROUT method (Q = 1%). Unpaired t-test with Welch's correction was performed to compare two groups. One-way ANOVA test with Holm-Sidak correction was applied to compare more than two groups. To evaluate the independence of categorical variables, chi-square test or Fisher's test was used. Results of statistical tests are represented in figure legends as follows: * p-value < 0.05, ** p-value < 0.01, *** p-value < 0.001, **** p-value < 0.0001.

Supplementary methods table 1 – Composition of the different diets used.

Diet	CD		WD		THD		0-3D		FPC		MCD	
Reference	D13012801		D13012802		D13012803		D13012804		TD.190142		E15653-94	
Company									Envigo		ssniff	
%	g	kcal	g	kcal	g	kcal	g	kcal	g	kcal	g	kcal
Protein	19.2	20	23.7	20	23.7	20	23.7	20	12.2	9.8	15	14
Carbohydrate	67.3	70	41.4	35	41.4	35	41.4	35	46.2	37.3	64.3	64
Fat	4.3	10	23.6	45	23.6	45	23.6	45	29.1	52.9	10	22
Total	100		100		100		100		100		100	
kcal/gm	3.85		4.73		4.73		4.73		5		4.73	

Supplementary methods table 2 – List of primers used and respective sequences.

Gene	Forward	Reverse	Species
miR-149	TCT GGC TCC GTG TCT TCA CT	-	
miR-182	TTT GGC AAT GGT AGA ACT CAC AC	-	
miR-183	TAT GGC ACT GGT AGA ATT CAC TAA	-	
miR-122	GGCTGTGGAGTGTGACAATG	-	
miR-16	ACAGCCTAGCAGCACGTAAAT	-	
univ-RT-polyT		GAGGTATTCGCACCAGAGGATTTTTTTTTTTTTTTVN	
Vim	CGG CTG CGA GAG AAA TTG C	CCA CTT TCC GTT CAA GGT CAA G	Mouse
Fn1	ATCTCGGAGCCATTTGTTCCT	CCAGGTCTACGGCAGTTGTCA	
Col1a1	GCT CCT CTT AGG GGC CAC T	CCA CGT CTC ACC ATT GGG G	
Acta	AAAAAAAACCACGAGTAACAAATCAA	TCAGCGCCTCCAGTTCCT	
Pdgfrb	GAGGCTTATCCGATGCCTTCT	AGACATGTTGCGAGTAGACAAAACTAA	
Krt18	CAG CCA GCG TCT ATG CAG G	CCT TCT CGG TCT GGA TTC CAC	
Il6	AGT TGC CTT CTT GGG ACT GAT	TCC ACG ATT TCC CAG AGA AC	
Il10	CTTTCAAACAAAGGACCAGC	CCAAGTAACCCTTAAAGTCCT	
Il1b	GACAACTGCACTACAGGC	CATGGAGAATATCACTTGTTGG	
Tgfb	CAACATGTGGAACTCTACCAG	TGTATTCCGTCTCCTTGGT	
Tnfa	AGGCTGCCCCGACTACGT	GACTTTCTCCTGGTATGAGATAGCAAA	
Itgam	ATG GAC GCT GAT GGC AAT ACC	TCC CCA TTC ACG TCT CCC A	
Fgf21	CAGTCCAGAAAGTCTCCTG	GATCAAAGTGAGGCGATCC	
Cd36	GTCTATCTACGCTGTGTTCG	ACAGGCTTTCCTTCTTTGC	
Acc1	GGACACCAGTTTTGCATTGA	AGTTTGGGAGGACATCGAAA	
Fasn	AAGTTGCCCGAGTCAGAGAACC	ATCCATAGAGCCCAGCCTTCCATC	
Cpt1a	ATGGCAGAGGCTCACCAAGC	GATGAACTTCTTCTTCCAGGAGTGC	
Acox1	CATGAATCCCGATCTGCG	TCAAGTTCTCGATTTCTCGAC	
Fabp4	CACCGAGATTTCCTTCAAACTG	TTTCATAACACATTCCACCACC	
Fatp5	TACAAGTTGGAGCCACCTG	TCACCCACATACAAGATCACTG	
Hmgcr	GTACATTCTGGGTATTGCTGG	GCACTCGCTCTAGAAAGG	
NANOG	TTT GTG GGC CTG AAG AAA ACT	AGG GCT GTC CTG AAT AAG CAG	Human
CDX2	GAC GTG AGC ATG TAC CCT AGC	GCG TAG CCA TTC CAG TCC T	
FOXA2	AGCGGTGAAGATGGAAGG	GTGTTCATGCCGTTCATCC	
HNF4A	CTCCTGCAGATTTAGCCG	CTGTCCTCATAGCTTGACC	
ALB	CTAGAGAAGTGCTGTGCC	CCACGGATAGATAGTCTTCTG	
SERPINA1	CTTCTTTAAAGGCAAATGGGAG	CTGGACAGCTTCTTACAGTG	
ALCAM	TCC TGC CGT CTG CTC TTC T	TTC TGA GGT ACG TCA AGT CGG	

CD68	GGA AAT GCC ACG GTT CAT CCA	TGG GGT TCA GTA CAG AGA TGC	
ACTA	TGATCACCATCGGAAATGAA	CGGCTTCATCGTATTCCTGT	
COL1A1	AACATGACCAAAAACCAAAAGTG	CATTGTTTCCTGTGTCTTCTGG	
IL6	AAATTCGGTACATCCTCGACGG	GGAAGGTTCAGGTTGTTTTCTGC	
IL8	CTGCGCCAACACAGAAATTA	ATTGCATCTGGCAACCCTAC	
IL1B	ATG GCT TAT TAC AGT GGC AA	GTC GGA GAT TCG TAG CTG GA	
TGFB	GTGACCTGGCCACCATTCAT	GTCAATGTACAGCTGCCGCA	
VIM	GCCCTAGACGAACTGGGTC	GGCTGCAACTGCCTAATGAG	

Supplementary methods table 3 – List of antibodies used and respective application.

Protein	Antibody production (Catalog number)	Dilution	Application
phospho AKT Ser	cell signaling (9271)	1/1000	WB
Total AKT	cell signaling (9272)	1/1000	WB
Tubulin	cell signaling (2128)	1/1000	WB
HNF4a	Santa-Cruz (SC-8987)	1/1000	WB
Phospho JNK	Cell signaling (9251)	1/1000	WB
Total JNK	Cell signaling (9252)	1/1000	WB
Phospho NFκB	Cell signaling (3037)	1/1000	WB
Total NFκB	Santa-Cruz (SC-372)	1/1000	WB
Phospo p38	Cell signaling (4511)	1/1000	WB
p38	Cell signaling (8690)	1/1000	WB
ERM	Cell signaling (3142)	1/1000	WB
PDGFR	abcam (ab32570)	1/1000	WB
HNF4a	Santa-Cruz (SC-8987)	1/100	IF
HNF4 - Alexa Fluor 555 (EPR16786)	abcam (ab217518)	1/100	IF
HNF4 - Alexa Fluor 647 (EPR3648)	abcam (ab217073)	1/100	IF
CD166	abcam (ab109215)	1/100	IF
CD166 - Alexa Fluor 488	abcam (ab197543)	1/100	IF
CD68 - Alexa Fluor 647	abcam (ab213363)	1/100	IF
CD68 - Alexa Fluor 647	abcam (ab224029)	1/100	IF
Iba1	abcam (ab178846)	1/2000	IF
GFP	Cell signalling (2956)	1/400	IF

Supplementary figures

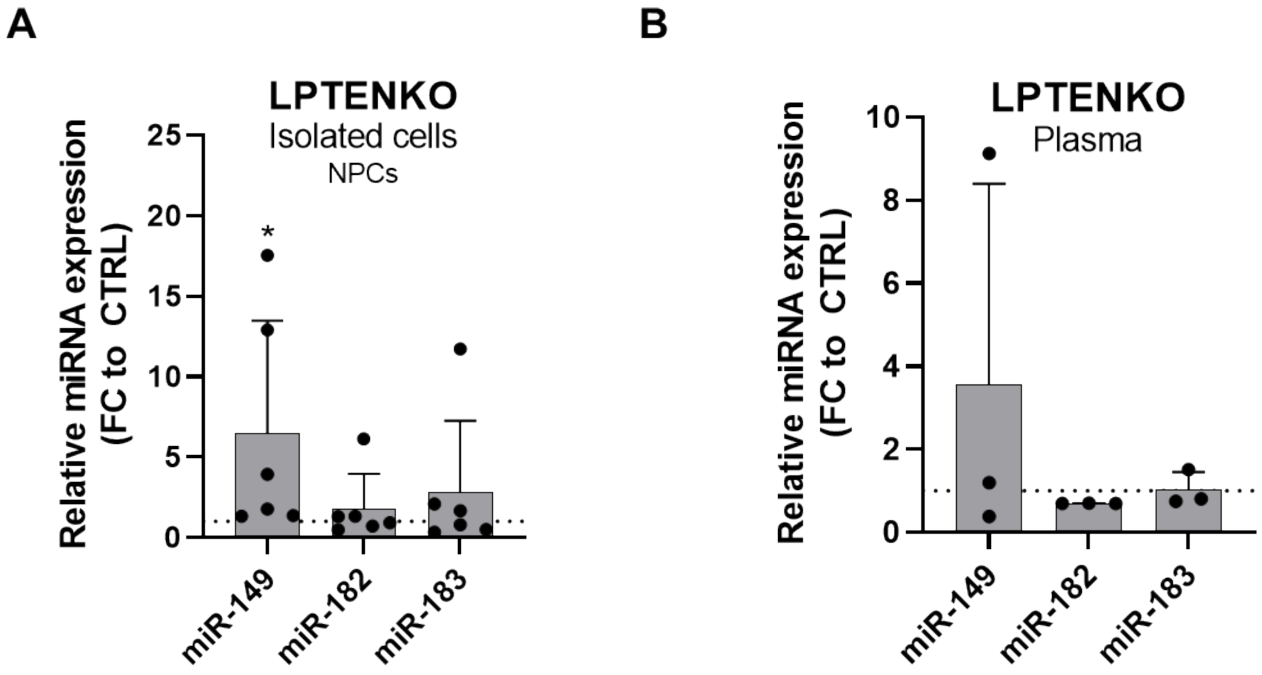

Fig. S1 – MiR-149-5p expression is upregulated isolated non-parenchymal cells (NPC) and in plasma from LPTENKO mice .
Relative miRNA expression of the top 3 most upregulated miRNAs identified in microarray analysis and validated through RT-qPCR in **(A)** non-parenchymal cells (NPC, n=4-6 per group) and **(B)** plasma (n=3 per group) of 4-months old CTRL and LPTENKO mice. Data is represented as mean ± SD. One-way ANOVA with Holm-Sidak's correction. *p-value < 0.05, **p-value < 0.01, ***p-value < 0.001, ****p-value < 0.0001.

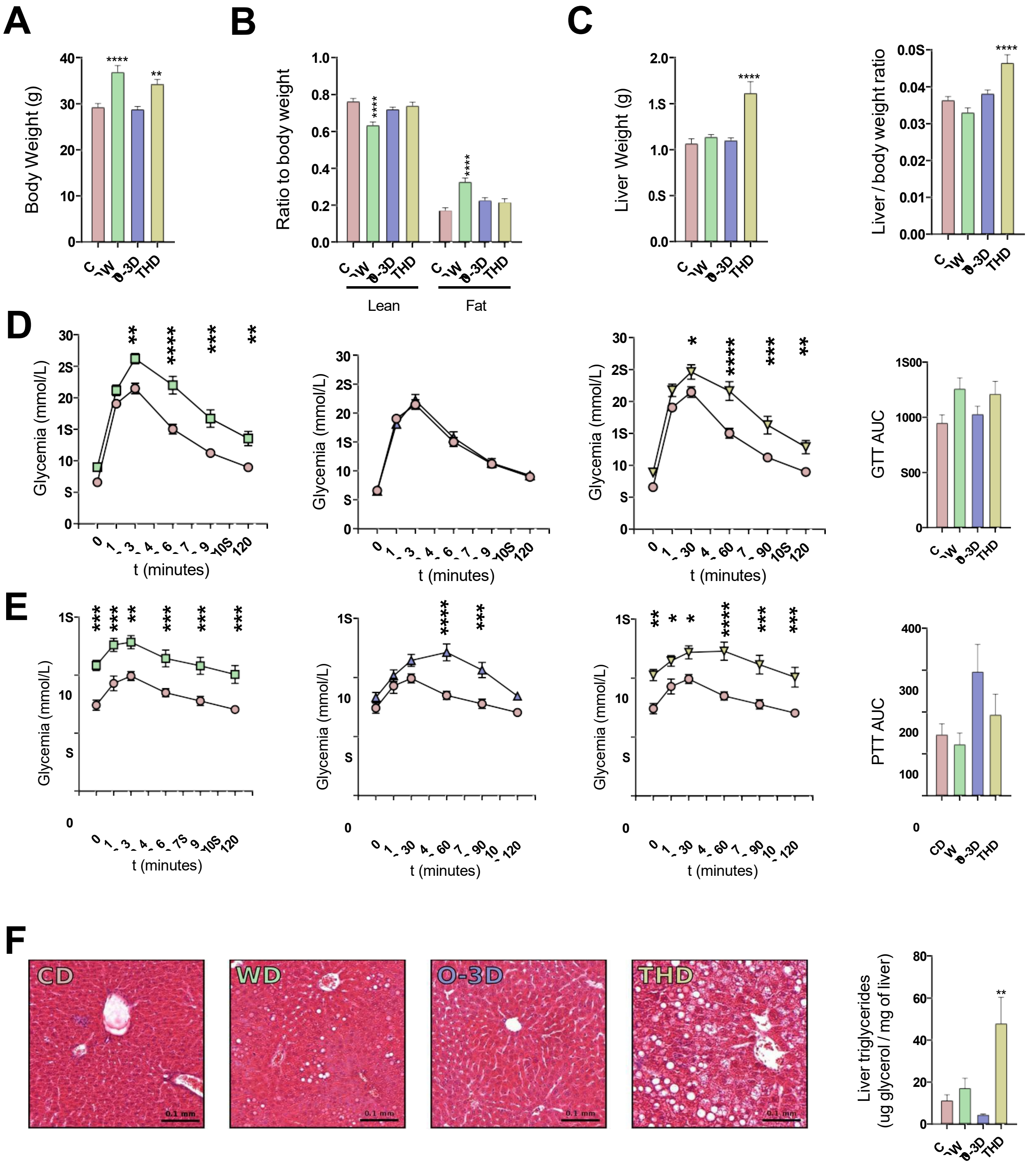

Fig. S2 – Impact of different high-sugar / high-fat diets on mouse metabolic phenotype. **(A)** Body weight, **(B)** body composition by echoMRI, **(C)** liver weight and liver/body weight ratio, **(D)** glucose tolerance test (GTT) and corresponding area under the curve (AUC), **(E)** pyruvate tolerance test (PTT) and corresponding area under the curve (AUC), **(F)** representative liver histology (hematoxylin and eosin staining) and **(G)** hepatic triglyceride content in 2-months old C57/BL6J mice fed for 16 weeks with control diet (CD, 10% cal from fat, 17% cal from sucrose) or with fatty acids-enriched diets (45% cal from fat, 17% cal from sucrose): either a mix of unsaturated/saturated fatty acids (Western diet, WD), omega-3 (O-3D) or trans-hydrogenated fatty acids (THD); n=6-12 per group. Data is represented as mean ± SD. One-way ANOVA with Holm-Sidak's correction. *p-value < 0.05, **p-value < 0.01, ***p-value < 0.001, ****p-value < 0.0001.

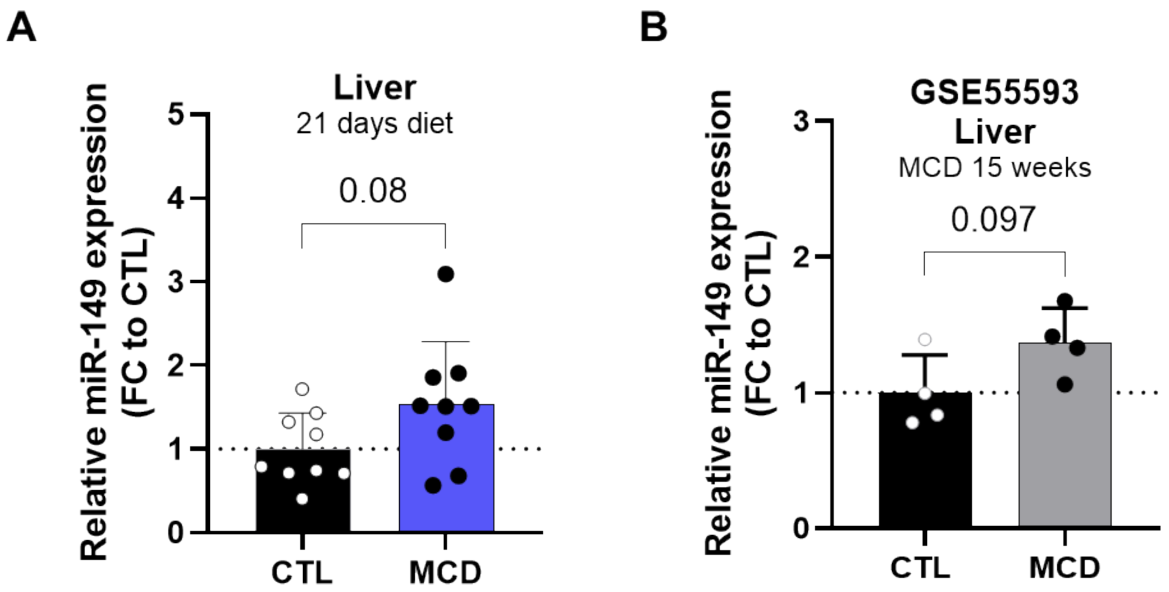

Fig. S3 – miR-149-5p expression is increased in hepatic tissues of mice fed a methionine/choline deficient diet.

(A) Relative miR-149-5p expression in hepatic tissue of diet-induced MASH model (21 days of methionine/choline deficient diet, left panel). **(B)** Gene expression omnibus (GEO) datasets analyses of miR-149-5p expression (FC to CTL) in hepatic tissues of mice fed MCD for 15 weeks (right panel). Unpaired t-test with Welch's correction. *p-value < 0.05, **p-value < 0.01, ***p-value < 0.001, ****p-value < 0.0001.

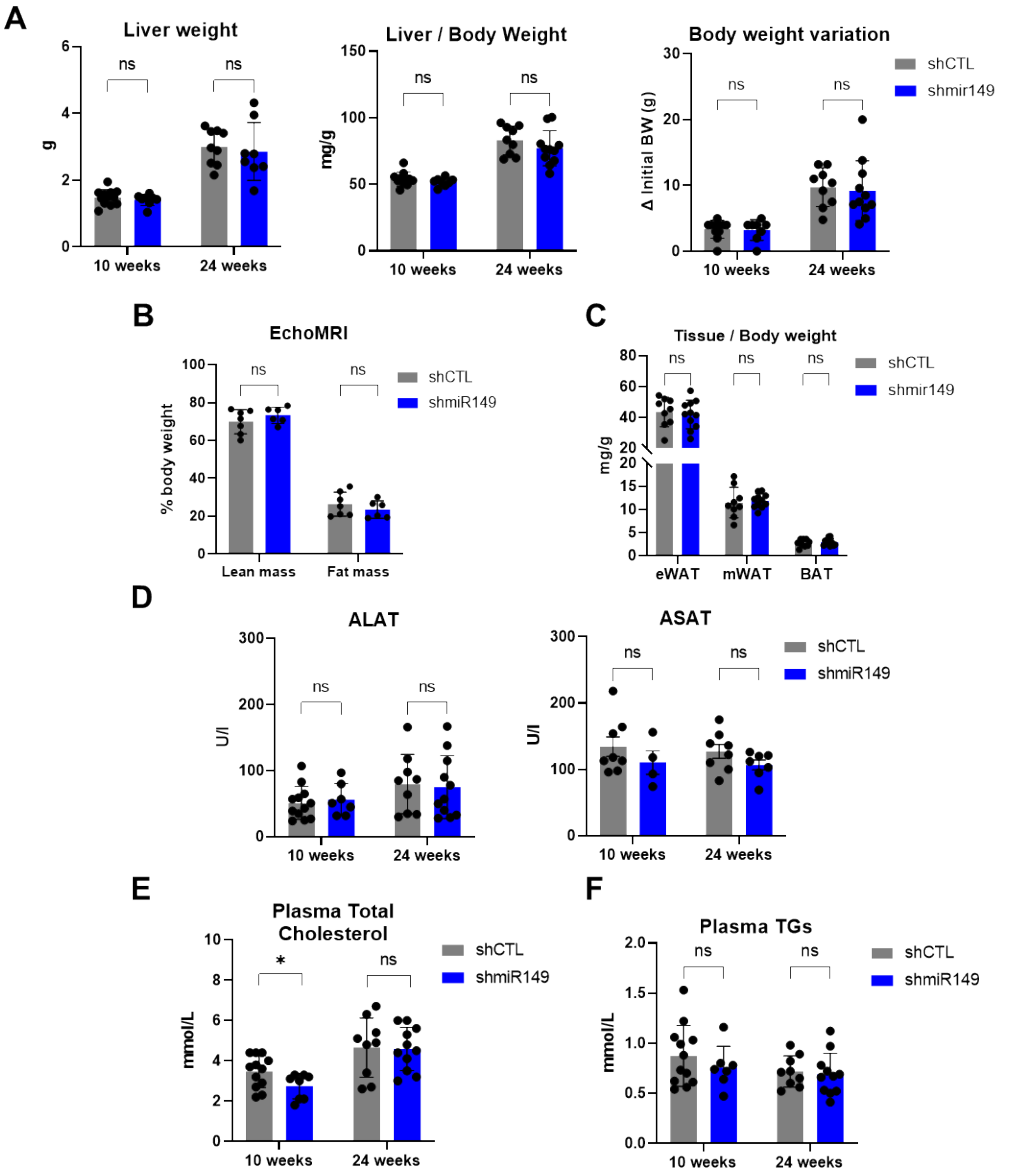

Fig. S4 – In vivo downregulation of miR-149-5p specifically in hepatocytes does not alter overall body parameters upon fructose/palmitate/cholesterol (FPC) diet.
(A) Liver weight, liver-to-body weight ratio and body weight gain of shCTL (n=9-12) and shmiR149 (n=8-11) mice fed FPC diet for 10 and 24 weeks. **(B)** EchoMRI analyses of lean and fat mass and **(C)** tissue-to-body weight ratio of explanted epidydimal (eWAT), mesenteric (mWAT) and brown (BAT) adipose tissue of shCTL (n=7-9) and shmiR149 (n=6-11) mice fed FPC diet for 24 weeks. Plasma levels of **(D)** transaminases (ALAT and ASAT), **(E)** triglycerides and **(F)** total cholesterol in shCTL (n=9-12) and shmiR149 (n=7-11) mice fed FPC diet for 10 and 24 weeks. Data is represented as mean ± SD. Unpaired t-test with Welch's correction or one-way ANOVA with Holm-Sidak's correction. *p-value < 0.05, **p-value < 0.01, ***p-value < 0.001, ****p-value < 0.0001.

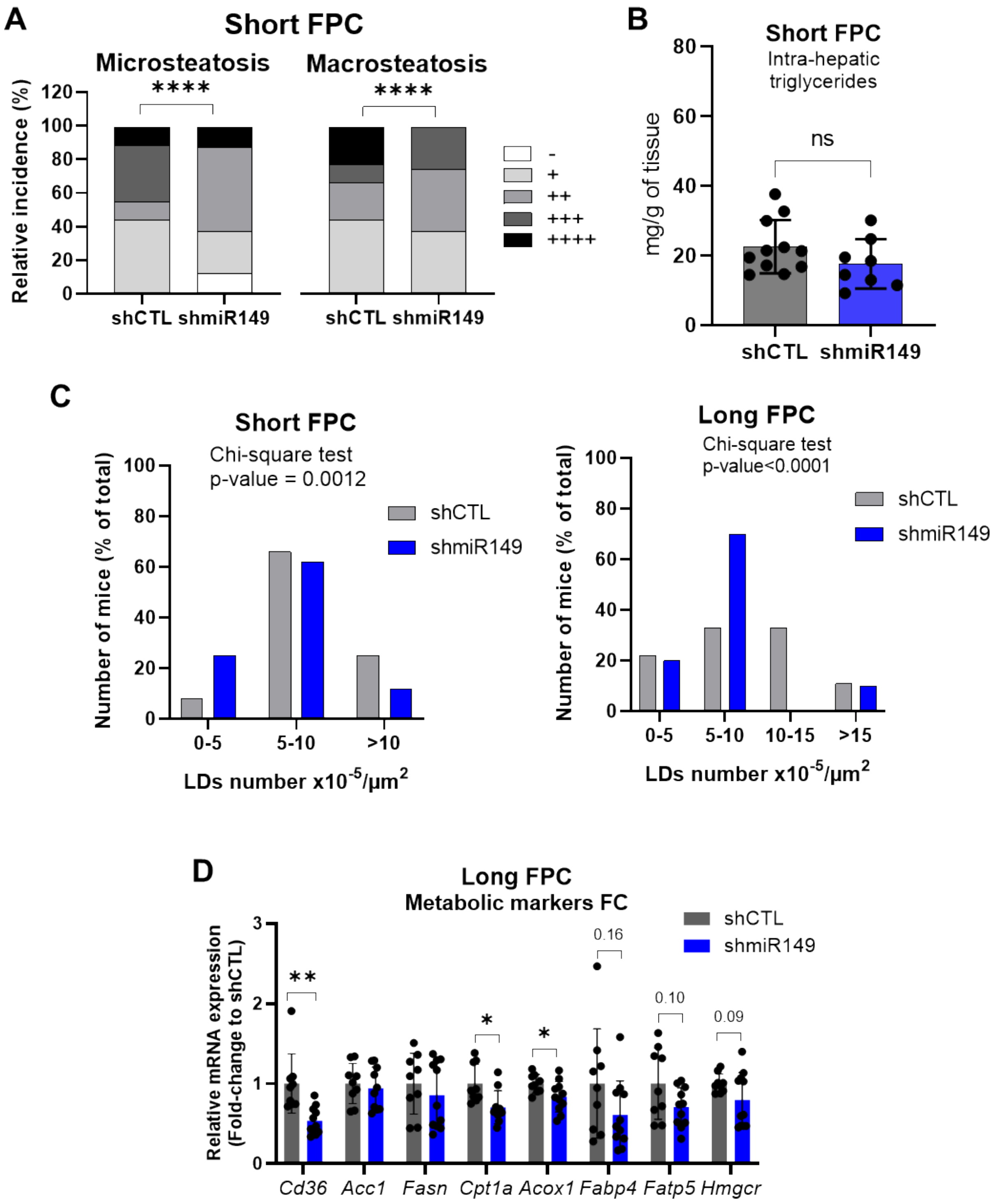

Fig. S5 – In vivo downregulation of miR-149-5p specifically in hepatocytes attenuates hepatic steatosis incidence and decreased markers of lipid uptake/ oxidation upon fructose/palmitate/cholesterol (FPC) diet.

(**A**)Blind scoring of micro-/macrosteatosis, **(B)** intra-hepatic triglycerides content, (**C)** Distribution of lipid droplet (LD) number per µm^2 of liver tissue area and **(D)** relative mRNA expression of metabolic markers in the explanted livers of FPC-fed mice after injection with hepatotropic adeno-associated virus (AAV8) harboring vectors encoding for scrambled shRNAs (shCTL, Long FPC - n=9; Short FPC – n=11) or for shRNAs specific for miR-149-5p (shmiR149, Long FPC - n=10-11; Short FPC n=8). Data is represented as mean Fold-change to shCTL ± SD. Unpaired t-test with Welch's correction, Fisher's test or Chi-square test. *p-value < 0.05, **p-value < 0.01, ***p-value < 0.001, ****p-value < 0.0001.

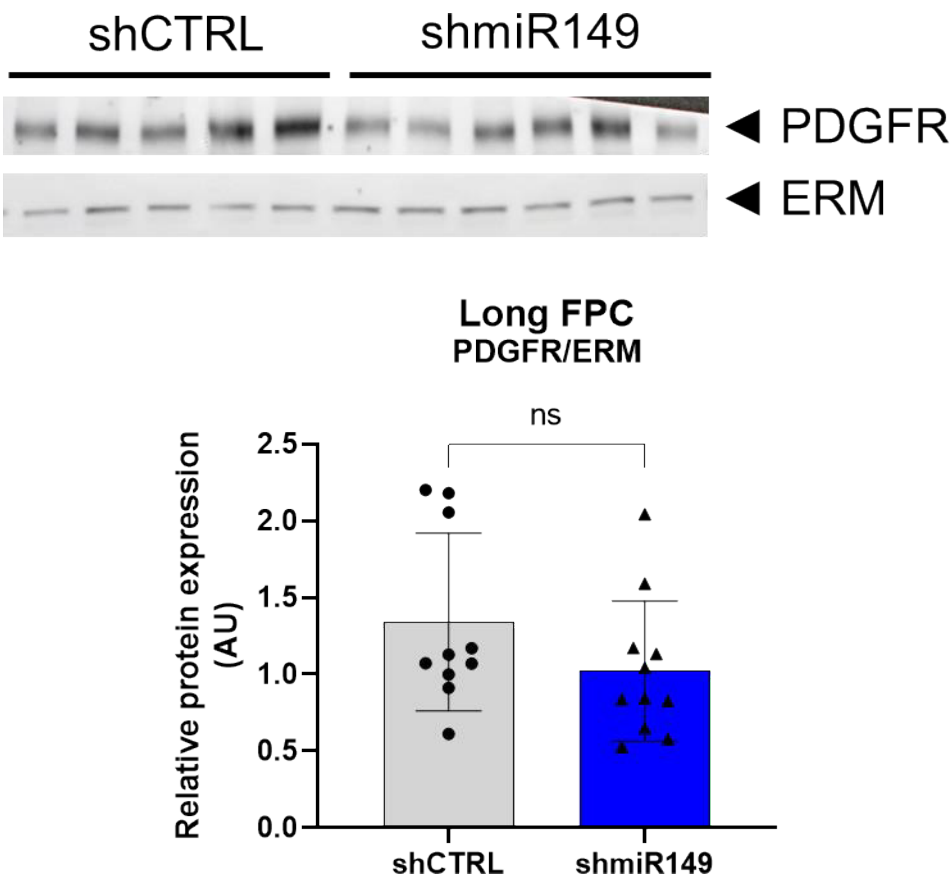

Fig. S6 – Inhibition of miR-149-5p in mice fed fructose-palmitate-cholesterol (FPC) diet tends to decrease PDGFR protein levels.
Western blot analysis (representative images on the left, n=5 shCTRL and n=6 shmiR149; quantifications on the right) of PDGFR protein expression in hepatic tissues of shCTL (n=10) and shmiR149 (n=11) mice after 24 weeks of FPC diet. ERM protein levels were used loading control. Data is represented as mean ± SD. Unpaired t-test with Welch's correction. *p-value < 0.05, **p-value < 0.01, ***p-value < 0.001, ****p-value < 0.0001.

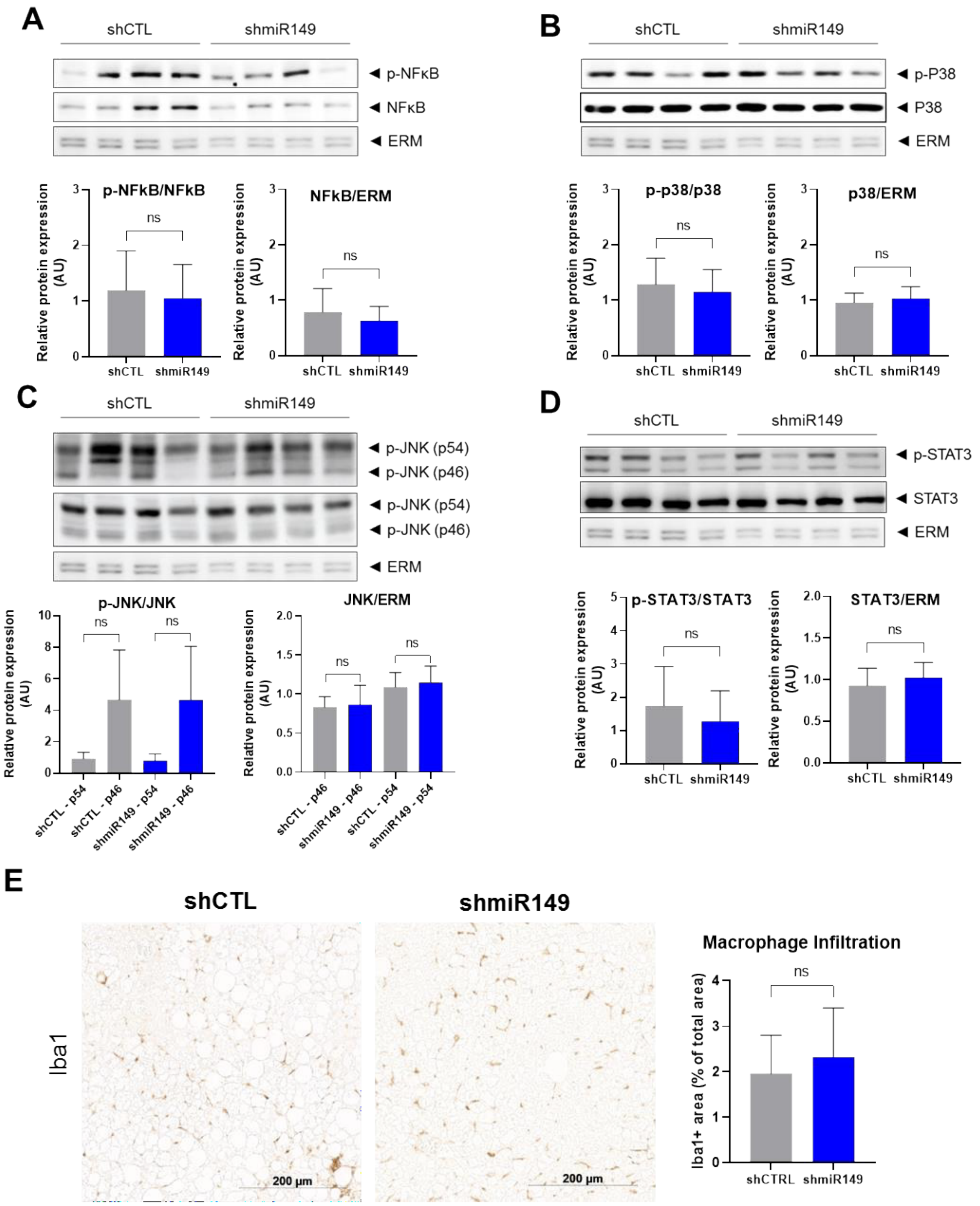

Fig. S7 – Inhibition of miR-149-5p in mice fed fructose-palmitate-cholesterol (FPC) diet does not impact inflammatory pathways nor macrophage infiltration.
Western blot analysis (representative images on the left, 4 mice per group; quantifications on the right) of phosphorylated over total expression of **(A)** NFκB, **(B)** p38, **(C)** JNK and **(D)** STAT3 signalling pathways in hepatic tissues of shCTL (n=9) and shmiR149 (n=11) mice after 24 weeks of FPC diet. ERM protein levels were used loading control. **(E)** Representative immunohistochemical staining of Iba1 in liver sections (left) and quantification of positive area over total tissue area (right) in shCTL (n=4) and shmiR149 (n=10) mice after 24 weeks of FPC diet. Data is represented as mean ± SD. Unpaired t-test with Welch's correction. *p-value < 0.05, **p-value < 0.01, ***p-value < 0.001, ****p-value < 0.0001.

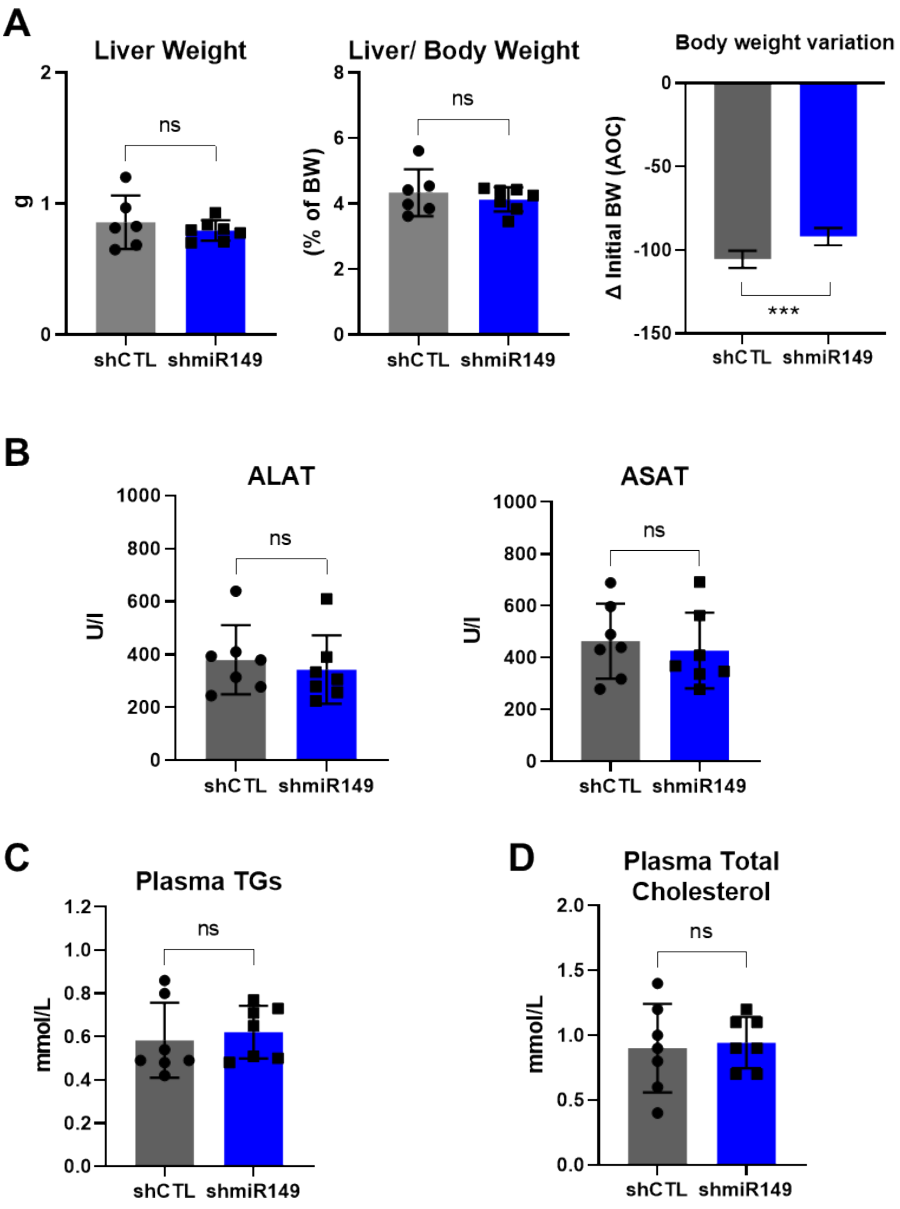

Fig. S8 – In vivo downregulation of miR-149-5p specifically in hepatocytes does not alter overall body parameters upon methionine-choline-deficient (MCD) diet.

(A) Liver weight, liver-to-body weight ratio (%) and body weight variation, **(B)** plasma levels of transaminases (ALAT and ASAT), **(C)** plasma triglycerides and **(D)** total cholesterol content in shCTL and shmiR149 fed MCD diet for 19 days after injection with hepatotropic adeno-associated virus (AAV8) harboring vectors encoding for scrambled shRNAs (shCTL) or for shRNAs specific for miR-149-5p (shmiR149, n=7 per group). Data is represented as mean ± SD. Unpaired t-test with Welch's correction. *p-value < 0.05, **p-value < 0.01, ***p-value < 0.001, ****p-value < 0.0001.

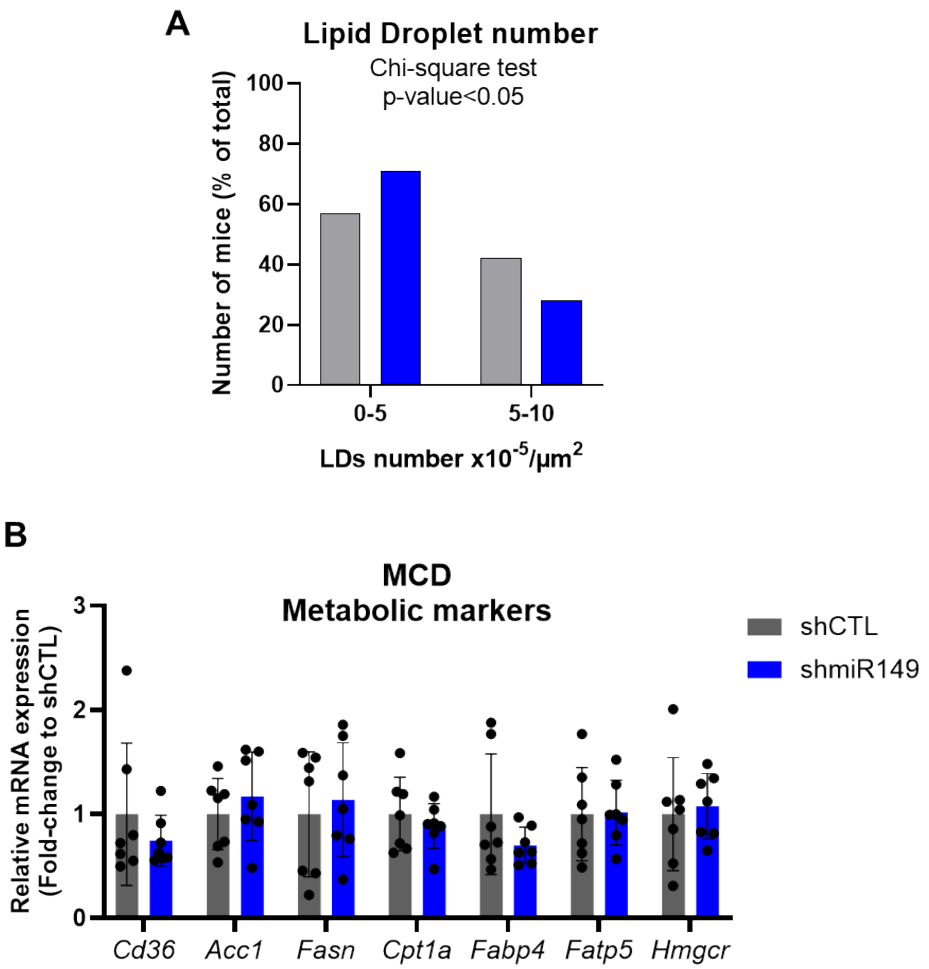

Fig. S9 – In vivo downregulation of miR-149-5p specifically in hepatocytes attenuates steatosis upon methionine-choline-deficient (MCD) diet.

(A) Distribution of lipid droplet (LD) number per μm^2 of liver tissue area and **(B)** relative mRNA expression of metabolic markers in the explanted livers of MCD-fed mice after injection with hepatotropic adeno-associated virus (AAV8) harboring vectors encoding for scrambled shRNAs (shCTL, n=7) or for shRNAs specific for miR-149-5p (shmiR149, n=7). Data is represented as mean Fold-change to shCTL ± SD. Chi-square test or Unpaired t-test with Welch's correction. *p-value < 0.05, **p-value < 0.01, ***p-value < 0.001, ****p-value < 0.0001.

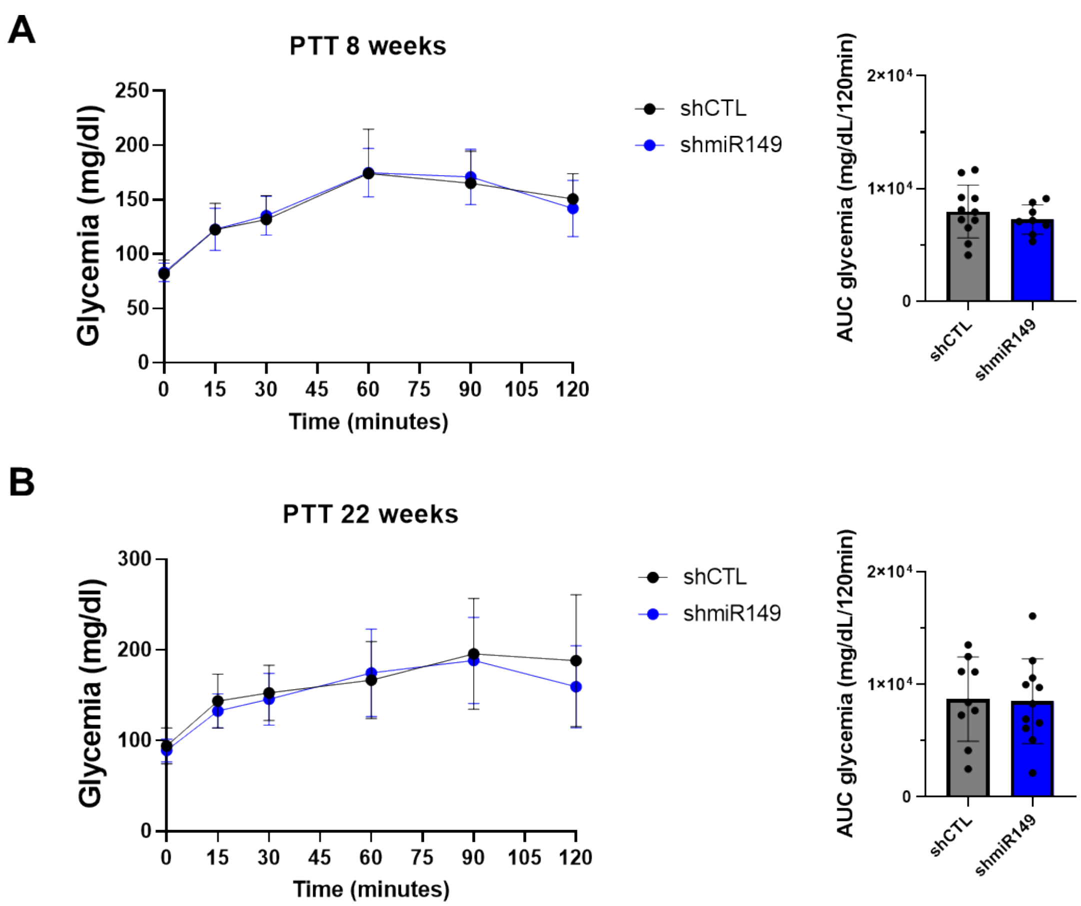

Fig. S10 – Inhibition of miR-149-5p in mice fed fructose-palmitate-cholesterol (FPC) diet does not affect hepatic glucose output.

Intraperitoneal pyruvate tolerance test (PTT, left panels) and calculated area under the curve (AUC, right panels) after 18 hours of fasting at **(A)** 8 and **(B)** 22 weeks of FPC-fed in shCTL and shmiR149 mice (n=8-11 per group at 8 weeks, n=9-11 per group at 22 weeks). Data is represented as mean ± SD. Unpaired t-test with Welch's correction. *p-value < 0.05, ** p-value < 0.01, ***p-value < 0.001, ****p-value < 0.0001.

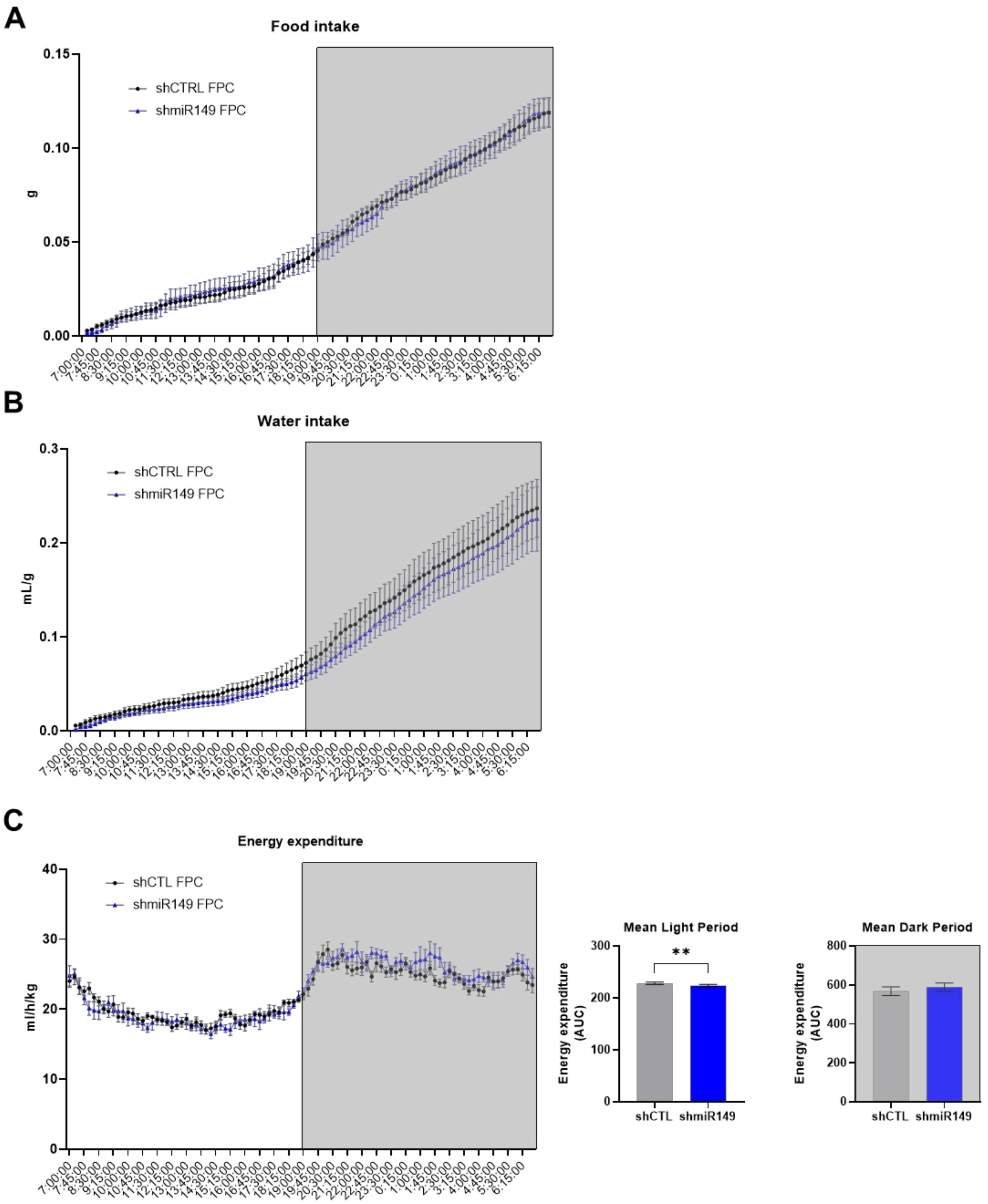

Fig. S11 – Inhibition of miR-149-5p in mice fed fructose-palmitate-cholesterol (FPC) diet does not impact energy expenditure, water and food intake.

(A) Food, **(B)** water intake and **(C)** energy expenditure rate and corresponding AUCs (light versus dark period, right panels) measured over 7 days (mean of the 7 days) in shCTL (n=7) and shmiR149 (n=6) mice after 23 weeks of FPC diet. Data is represented as mean ± SD. Unpaired t-test with Welch's correction. *p-value < 0.05, ** p-value < 0.01, ***p-value < 0.001, ****p-value < 0.0001.

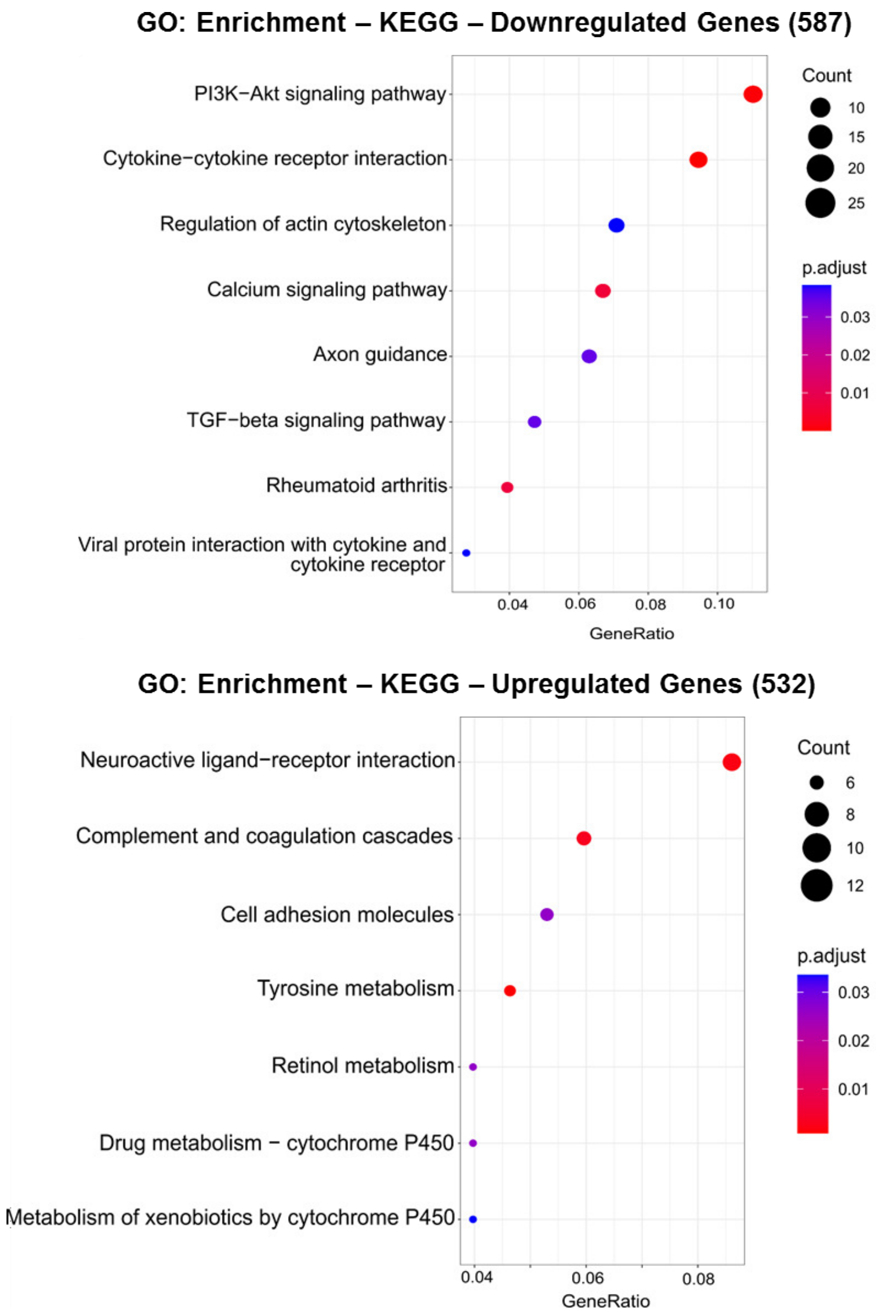

Fig. S12 – Overexpression of miR-149-5p in the hepatic cell line Huh7 leads to deregulation of genes involved in metabolic and inflammatory pathways.
Gene ontology enrichment analysis of KEGG pathways with **(A)** downregulated and **(B)** upregulated genes identified in transcriptomic analyses of polysomal fractions from Huh7 cells transfected with synthetic oligonucleotides mimicking miR-149-5p or with scrambled mimics. Significantly deregulated genes were identified using the following thresholds – fold-change to control mimic = |1.5| and false discovery rate (FDR) < 0.05.

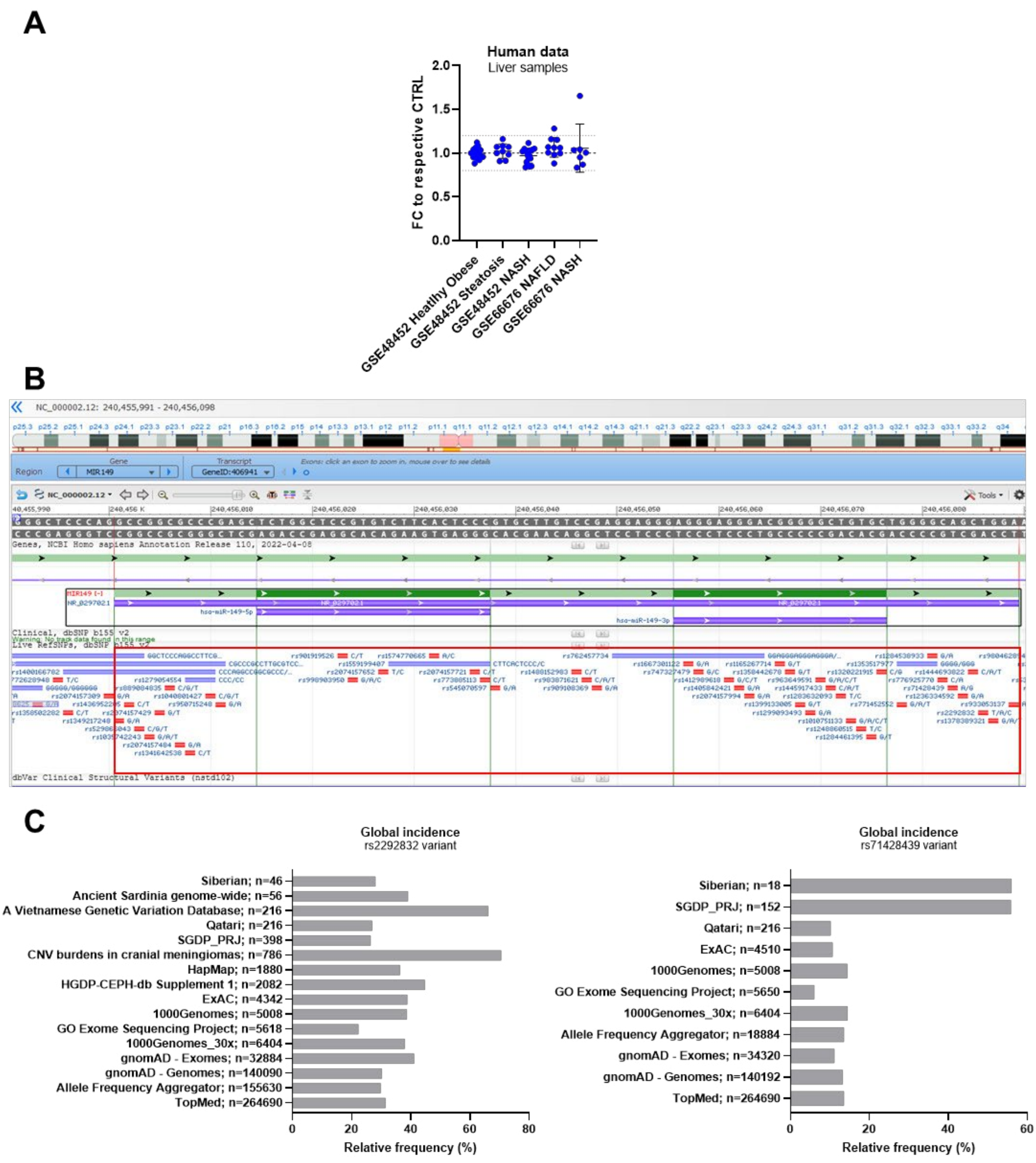

Fig. S13 – miR-149 expression in patients diagnosed with hepatic diseases and human polymorphisms identified in the genomic region encoding miR-149.

(A) Gene expression omnibus (GEO) datasets analyses of miR-149-5p expression (FC to CTRL) in hepatic tissues of healthy obese, steatotic or MASH patients. Data is represented as mean ± SD fold change (FC) to CTRL group. **(B)** Forty-five single nucleotide polymorphisms (SNP) annotated in miR-149 region (source: dbSNP-NCBI, accessed on 03/04/2024). **(C)** Global incidence of two of them (rs2292832 and rs71428439) with reported effects on miR-149 maturation and linked with susceptibility to develop several pathologies, including metabolic (dysfunction) – associated steatotic liver disease (MASLD, previously known as non-alcoholic fatty liver disease – NAFLD) and hepatocellular carcinoma (Table S2). Unpaired t-test with Welch's correction. *p-value < 0.05, ** p-value < 0.01, ***p-value < 0.001, ****p-value < 0.0001.

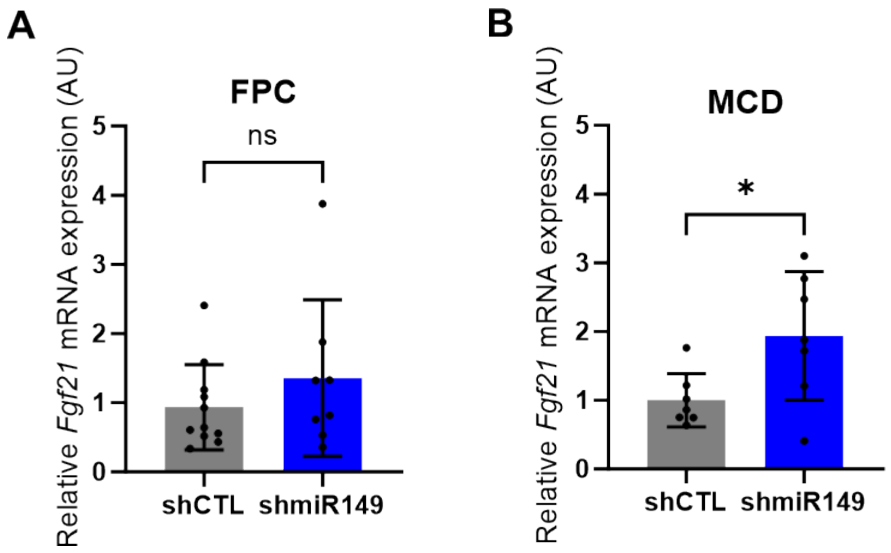

Fig. S14 – In vivo downregulation of miR-149-5p specifically in hepatocytes modulates *Fgf21* hepatic expression upon fructose/palmitate/cholesterol (FPC) diet or methionine/choline-deficient (MCD) diet. Relative *Fgf21* mRNA expression in livers of **(A)** fructose/palmitate/cholesterol (FPC) diet-fed mice or of **(B)** methionine/choline-deficient (MCD) diet-fed after injection with hepatotropic adeno-associated virus (AAV8) harboring vectors encoding for scrambled shRNAs (shCTL, n=7-12) or for shRNAs specific for miR-149-5p (shmiR149, n=7-11). Data is represented as mean ± SD fold change (FC) to CTRL group. Unpaired t-test with Welch's correction. *p-value < 0.05, ** p-value < 0.01, ***p-value < 0.001, ****p-value < 0.0001.

Supplementary tables

Supplementary tables

Table S1 – Literature screening performed using the 237 potential targets of miR-149 identified with translatomic analyses. Only studies related with MASLD/MASH or metabolic deregulations were considered.							
**Gene Name (Mouse)**	**Human Ortholog**	**Expression (FC to CTRL Mimic)**	**Adj p-value (FDR)**	**Investigated in liver disease7 Yes/No**	**Role in Steatosis/Fibrosis/Liver inflammation (NAFLD)7**	**PMID**	**▲ or ▼/ promotes which process)**
Cx3cl1	CX3CL1	-2.762777121	7.34E-06	Yes	Overexpression of Cx3cl1 attenuated glucose intolerance, hepatic IR and fibrosis (but not steatosis) after atherogenic diet for 16 weeks	35914622	▲/Restrains glucose intolerance, hepatic IR and fibrosis
Pdgfrb	PDGFRB	-4.257690608	7.33E-06	Yes	Used as a marker of liver fibrosis; Linked with stellate cell activation, hepatic collagen production and hepatic fibrosis. Regulated by PTEN. Increased in MCD and HFD fed mice with positive correlation with fibrosis scores.	31036530; 35834952, 30901579	▲ / promotes hepatic fibrosis
Vcan	VCAN	-11.12010812	7.53E-06	yes, but no mechanistic in vivo	Identified as potential marker of hepatitis-B related fibrosis and immune infiltration (increased with fibrosis). Identified also as a transcriptomic signature of MASH progression	36275632; 33837926; 30046009	▲ / promotes hepatic fibrosis and immune cell infiltration
Lefty1	LEFTY1	-2.92961806	0.007379222	yes	Delivery of Lefty1 mRNA through vesicles in HSC reduced fibrosis in rats injected with CCL4	36382306	▲/attenuates fibrosis
Zfand3	ZFAND3	-2.073016339	0.000111107	Yes	ZFAND3 overexpression in the mouse liver improves glucose tolerance and hepatic insulin resistance.	33782927	▲/attenuates hepatic IR
Batf	BATF	-2.345494153	0.017892142	Yes	AAV-induced overexpression of BATF alleviated HFD-induced steatosis	37712938	▲/attenuates hepatic steatosis
Fgfr1	FGFR1	-2.028466904	2.61E-05	Yes	Same litterature than FGF21. Nothing with a genetic alteration of FGFR1.	34654875	▲/attenuates hepatic steatosis, inflammation hyperglycemia and IR
Gdf15	GDF15	-2.72025072	0.001164787	Yes	Gene signature strongly associated with MASH. Treatment with recombinant Gdf15 ablated lipid accumulation in primary hepatocytes . Gdf15 Associated with weight loss; GDF15 correlates positively with better oxidative stress status.	33268509; 31857195; 35504134	▲/attenuates lipid accumulation
Fgf21	FGF21	-3.801489604	0.00563014	Yes	Fgf21 treatment reduced hepatic steatosis, lobular inflammation, hepatocyte ballooning and fibrosis in mice under highfat-highcholesterol diet for 23w. It also modulated immune cell population (decreased eosinophils, neutrophils and B cells), prevented activation of Kupffer cells and facilitated cholesterol removal. It also reduced MCD-induced hepatic and intestine injury - including hepatic steatosis and fibrosis. It also induced alterations in gut microbiota, short FA and BA metabolism	36648330, 36586452	▲/attenuation of hepatic steatosis, inflammation/injury and, immune infiltration
Tnip2	TNIP2	-2.487236906	1.46E-05	yes	Overexpression of Tnip2 led to inhibition of NFκB and delayed liver regeneration after partial hepatectomy.	16480954	▲/Inhibits NFκB (potential impact on chronic inflammation)
Fmnl1	FMNL1	-2.989480911	0.00536369	Yes	Transcriptome data provided a novel landscape of the Monocyte-Macrophage-Dendritic Cell (MMD) system that is involved in advanced MASH disease status. Fmnl1 expression in the MMD system was associated with the progression of MASH fibrosis.	36911682	▲/marker of fibrosis
Shh	SHH	-2.007896799	0.001719701	Yes	Marker of hepatocytes ballooning by IHC / MASH	35224757; 30744560	▲/marker of hepatic injury (ballooning)
Casr	CASR	-4.014213997	0.027757892	Yes (Liver not NAFLD)	In Wistar rats, the upregulation of Casr expression is involved in the occurrence of diabetic liver injury and fibrosis.	32476365	▲/marker of hepatic injury and fibrosis
Btg2	BTG2	-2.366617691	0.000152796	Yes	Overexpression of Btg2 in wild-type mice promoted the expression of gluconeogenic enzymes and hyperglycemia, while its downregulation in db/db mice attenuated glucose intolerance.	37488285	▲/promotes gluconeogenesis and hyperglycemia
C7	C7	-3.397567566	0.000128303	Yes	Functional characterization not available, but plasma levels of C7 correlated positively with fibrosis extent.	31860081	▲/promotes hepatic fibrosis
Ncf2	NCF2	-3.115587612	0.000145192	yes, but indirect mechanisms	HSC-specific SRF KO mice have attenuation of hepatic fibrosis induced by MCD (due to inhibition of NCF1/NCF2 transcription); NCF2 expression is increased in the livers of WD-induced MASH in female Ldlr KO mice	31442911; 31042760	▲/promotes hepatic fibrosis
Kif5a	KIF5A	-4.13575848	0.00031402	Yes	Increased expression linked with a specific subtype of HCC (increased CD8+ infiltration and anti-tumor responses)	36686769	▲/Promotes immune cell infiltration (inflammation)
Pde4d	PDE4D	-2.498215842	1.39E-05	Yes	Overexpression of PDE4D with AAV8 under chow diet impaired insulin signalling and promoted hepatic steatosis (due to increased expression of Cd36 - lipid uptake promoted). Treatment with Roflumilast (PDE4 inhibitor) under HFD conditions improved hepatic steatosis induced by the diet. Similarly, liver-specific PDE4D KO under HFD gained less weight, had attenuation of hepatic steatosis and other metabolic deregulations linked with the diet (i.e. hyperglycemia, insulin resistance)	34826603; 37072403	▲/Promotes Steatosis, hyperglycemia and IR
Rnaseh1	RNASEH1	-3.410011433	2.08E-05	Yes	RNaseH1 KO mice presented mitochondrial dysfunction and presented hepatic injury and fibrosis.	27131367	▼ / promoted hepatic injury and fibrosis

Phlpp2	PHLPP2	-3.446898415	1.02E-05	yes	Liver specific Phlpp2 KO mice showed increased liver weight and hepatic steatosis but normal glucose metabolism under chow diet. Overexpression decreased TG accumulation under HFD diet due to decreased lipogenesis	28859855, 26743335	▼ / promoted hepatic steatosis
Cidec	CIDEC	-2.266642423	0.004992412	Yes	Hepatic expression levels of Cidec/Fsp27 increases with MASLD progression to MASH. Adipose tissue specific Cidec (Fsp27) KO were protected against BW gain but had increased hepatic steatosis, dyslipidemia and IR in HFD fed mice. Silencing of Fsp27 with synthetic oligos systemically did not affect diet-induced hepatic steatosis/ steatohepatitis, but improved glucose tolerance and IR. Hepatic specific deletion of Fsp27 restrained hepatic steatosis and injury, inflamation and fibrosis induced by chronic ethanol feeding. In another study, KD led to decreased hepatic steatosis induced by fasting by promoting FAoxidaition.	25477509; 27884961; 28874443; 31097771; 26099526;	▼ in AT/promotes hepatic steatosis, glucose intolerance, IR and inflammation; ▼ in Liver/restrains hepatic steatosis, inflammation and fibrosis
Crtc2	CRTC2	-2.632009038	9.59E-06	Yes	Total CRTC2 KO total promoted HFD induced steatosis due to regulation of SREBP1c expression. Hepatocyte specific CRTC2 KO attenuated hepatic steatosis and macrophage infiltration	26147081; 34838715	▼ in hepatocytes/ attenuates hepatic steatosis and macrophage infiltration
S1pr2	S1PR2	-3.753428249	6.19E-06	yes	S1pr2 KO specifically in liver sinusoidal endothelial cells attenuated hepatic injury and fibrosis induced by CCl4 injection, while overexpression led to the inverse phenotype due to activation of YAP signalling and increased expression of TGF-³. Downregulation of S1pr2 in hepatocytes also attenuated hepatic injury and fibrosis induced by a DDC diet. Inhibition of S1pr2 with pharmacological agents attenuates fibrosis and HCC development in a model of congestion-induced fibrosis model and NLRP3 inflammasome priming/neutrophil infiltration/cytokine expression in a bile duct ligation model of hepatic injury, mice fed a HFD/MCD or CCl4 injection. Conversely, S1PR2 blockage increased diacylglycerol and TG levels in the liver of Mc4r-KO mice fed a western diet for 20 weeks	37039817; 36800698; 34855990; 33388881; 33197723; 32768187; 32695095	▼/ attenuates hepatic inflammation, fibrosis and neutrophil infiltration but promotes steatosis (DG/TG accumulation)
Cmtm3	CMTM3	-3.4186108	7.53E-06	yes, in HCC	Cmtm3 KO mice had attenuated liver damage induced by DEN injection (decreased ALT/AST and hepatic cytokine expression),	36284038	▼/ attenuates hepatic inflammation/injury
Cxcl12	CXCL12	-9.63069051	6.19E-07	yes, but no mechanistic in vivo	Inhibitor of CXCR4 (receptor of Cxcl12) decreased immune cell infiltration (CD4+ T cells) in the livers of db/db mice, but it promoted hepatic inflammation and fibrosis after CCl4-injection due to increased neutrophiles..	25074471; 25163538	▼/ promotes hepatic inflammation, fibrosis and neutrophil infiltration
Csf1	CSF1	-3.968651991	7.33E-06	yes	CSF1 circulating levels associated with a decline in MASLD severity. CSF1 inhibition with antibodies in a thioacetamide (TAA)-induced liver injury model promoted hepatic inflammation, but when TAA was removed Csf1-Ab promoted fibrosis resolution. In neonatal rats, Csf1-Ab promoted reversible hepatic steatosis and hepatic macrophage accumulation. Similar effects regarding macrophage accumulation in mice after partial hepatectomy and acetaminophen intoxication.	34006921; 35169835; 29351395; 26344055	▼/ promotes hepatic inflammation/fibrosis upon injury, but also resolution when damaging agent is removed
Fhl2	FHL2	-2.056473863	0.004105145	Yes	Fhl2 KO mice are more sensitive to fibrosis, liver injury and inflammation induced by bile duct ligation and CCL4 injection .	31963815; 28223370; 23311569	▼/aggravates hepatic injury, inflammation and fibrosis
Ptpn14	PTPN14	-2.480957533	4.97E-05	yes	Ptpn14 KO mice are less sensitive to acute liver injury (by interfering with SOCS7-dependent cytokine expression)	32978373	▼/attenuated hepatic injury and inflammation
Dyrk1b	DYRK1B	-2.320668358	6.38E-05	Yes	DYRK1B mutation associated with MASLD in humans and it is increased in MASH patients. KD of DYRK1B via AAV-shRNA reduced steatosis and fibrosis induced by high fat- high fructose diet due to increased lipogenesis and fatty acid uptake	34855620	▼/attenuated hepatic steatosis and fibrosis
Loxl2	LOXL2	-2.593775735	0.000530223	Yes	Injection of Loxl2/Loxl3 inhibitors ammeliorated hepatic fibrosis induced by CCL4 injection and sterptozotocin/HFD diet; Loxl2 expression is increased in experimental models of MASLD (MCD,MCD+HFD) and it is also increased in diabetic patients with hepatic fibrosis	30536539; 28468951	▼/attenuates fibrosis
Satb1	SATB1	-2.046876779	0.002052137	yes	KD of Satb1 in hepatocytes attenuated TAA- and CCl4-induced hepatic fibrosis in rats and in mice.	25896016; 27883059	▼/attenuates fibrosis

Tgfb1i1	TGFB1I1	-2.37163997	0.00611023	Yes	Tgfb1i1 (alias Hic-5) downregulation attenuated mouse liver fibrosis and HSC activation induced by bile duct ligation and CCl4 injection by inhibiting TGFB-Smad2 signalling and collagen/a-sma expression	26334580	▼/attenuates fibrosis
Sema3c	SEMA3C	-2.127413743	0.007366958	yes	Increased in human MASH and in MASH mouse models. Specific deletion of Sema3c in myofibroblasts and in HSCs attenuated fibrosis induced by CCl4 injection.	37055018; 36551769	▼/attenuates fibrosis
Cftr	CFTR	-2.096992615	0.003642721	Yes	Pharmacological inhibiton of CFTR --> decrease fibrosis induced by HFMCD	36754244	▼/attenuates fibrosis
Mical2	MICAL2	-2.205685168	8.42E-05	Yes	Mical2 silencing alleviated hepatic fibrosis and injury induced by CCl4 injection.	32659284	▼/attenuates hepatic injury and fibrosis
Shmt2	SHMT2	-2.394399467	0.002234562	Yes	In mice injected with CCl4 and fed WD, HSC overexpressed Shmt2.<br>KD of Shmt2 in these mice decreased fibrosis and hepatic injury (ALT/AST levels) after two weeks of treatment.	PMID: 37307917	▼/attenuates hepatic injury and fibrosis
Atf3	ATF3	-2.562711026	0.001109398	Yes	It was found that Atf3 switches cell death from apoptosis to necroptosis in hepatic steatosis. In severe hepatic steatosis, after partial hepatectomy, hepatic ATF3-deficient mice displayed decreased RIPK3 expression and necroptosis. Similarly, another study knockdown of ATF3 attenuated glucose intolerance, IR and inflammation in Zucked diabetic rats	36690638; 28365312	▼/attenuates hepatic injury, glucose intolerance, IR and inflammation
Klhl3	KLHL3	-2.542066047	0.003069474	yes	Klhl3 KO mice showed attenuated BW gain and IR, hepatic steatosis and glucose intolerance after aging or HFD due to increased exergy expenditure and O2 consumption. Upon MCD diet, the total KO showed attenuated hepatic steatosis and injury linked with decreased macrophage infiltration and fibrosis. Similar observations with KD in the hepatocytes specifically.	36028759	▼/attenuates hepatic injury, steatosis, inflammation, fibrosis and glucose intolerance
Il11	IL11	-4.51222769	0.002727796	yes	Hepatocyte specific deletion of Il11 attenuates hepatocyte death, hepatic steatosis, fibrosis and inflammation and reduces plasma levels of glucose, cholesterol and TG levels induced by High-fat MCD diet for 4 weeks or a WD for 16 weeks. Blockage of IL-11 attenuated hepatic steatosis, fibrosis, hepatocyte death, inflammation and hyperglycemia following HFMCD diet for 6/10 weeks and on db/db mice fed MCD diet.	33397952; 31078624	▼/attenuates hepatic injury, steatosis, inflammation, fibrosis and hyperglycemia
Pld1	PLD1	-2.181075369	8.67E-05	Yes	Hepatocyte-specific Pld1 deficiency ameliorates lipid accumulation induced by HFD by inhibiting the PPAR´/CD36 pathway. PLD1 may be a new target for the treatment of MASLD. Similarly, PLD1 was found to play an important role in hepatic steatosis via the regulation of autophagy in Pld1−/− mice.	37138676; 27976696	▼/attenuates hepatic steatosis
Tnfrsf9	TNFRSF9	-3.357319337	0.001410558	Yes	HFD-induced glucose intolerance/insulin resistance and hepatic steatosis and inflammation were attenuated in the 4-1BB-deficient mice.	21998397	▼/attenuates hepatic steatosis and inflammation, glucose intolerance and IR
Tlr2	TLR2	-2.476088408	0.012677243	Yes	Blockage of TLR2 in rats under HFD attenuated hepatic steatosis, fasting hyperglycemia, inflammation and fibrosis. But Tlr2 KO mice were more sensitive to MCD-induced hepatic steatohepatitis and fibrosis.	32151955; 20509914	▼/attenuates hepatic steatosis, fasting hyperglycemia, inflammation and fibrosis under HFD but aggravates steatohepatitis and fibrosis under MCD
Stk38	STK38	-2.093259298	8.01E-05	Yes	HFD induces the expression of hepatic STK38 promotes systemic inflammation and IR. Overexpression of STK38 in mouse liver leads to hepatic inflammation and steatosis, IR and hypertriglyceridemia in mice fed on a regular chow diet.KO of STK38 in HFD-fed mice attenuates inflammation, improves hepatic insulin sensitivity, and decreases hepatic fat accumulation. STK38 binds to Tank-Binding protein Kinase 1 and which promotes NF-κ³ nuclear translocation (expression of proinflammatory cytokines). Intrahepatic lipid accumulation due to enhanced de novo lipogenesis via reducing the AMPK-ACC signaling axis.	37028764	▼/attenuates inflammation, IR and steatosis
Bach2	BACH2	-2.237880831	0.000538247	Yes	Bach2 expression was increased in mice with hepatic steatosis. PCR, oil red O staining and triglyceride detection revealed that the silencing of BACH2 reduced lipid accumulation in hepatoblastoma cell line.	37362825	▼/attenuates lipid accumulation

Spp1	SPP1	-2.666216212	0.000392201	Yes	Increased in MASLD in humans. Spp1 impact in MASLD is inconclusive. Spp1 KO mice fed a HFD had decreased hepatic steatosis (due to decreased lipogenesis), glucose intolerance, insulin resistance and lobular inflammation/hepatocyte ballooning. However, Spp1KO mice treated with streptozotocin and challenged with HFD for 4 weeks had increased steatosis and hepatic injury (ALT levels increased). DNL was decreased but FA uptake as well as fibrosis was promoted in this context. In other studies, it was shown that Spp1KO mice fed MCD or HFD with 2% cholesterol had no effect regarding steatosis but they were protected from fibrosis. In WT mice under standard chow diet, treatment with recombinant osteopontin led to increase in liver PC/PE/Cholesterol and TG.	32730345, 21562757, 32281248, 32986864, 15044174, 31852298, 28754826	▼/Inconclusive. Depending on the stimuli it can promote/attenuate hepatic steatosis, inflammation/injury, fibrosis, glucose intolerance, IR
Inhbe	INHBE	-2.857728121	0.014191069	Yes	INHBE positively correlated with insulin resistance and body mass index in humans.Additionally, Inhbe gene expression increased in the livers of db/db mice. Downregulation of INHBE suppressed body weight gain due to decreased fat rather than lean mass. It also decreased the respiratory quotient and increased plasma total ketone bodies, suggesting enhanced whole-body fat utilization.	29596463	▼/increases fat utilization and decreases fat mass
Sema3d	SEMA3D	-2.249738309	0.000153715	Yes	Sema3d is downregulated in HCC, it's downregulation led to promoted tumor growth/EMT and metastasis in xenograft HCC models due to inactivation of Pi3k/Akt by FLNA (Sema3d interactor)	35957887	▼/Inhibition of PI3K/AKT
Cblb	CBLB	-2.019852724	0.00026572	yes, but no mechanistic in vivo	Aged total Cblb KO mice showed increased glucose intolerance, IR and hepatic steatosis. Dendritic cells-specific KO mice showed signs of hepatic fibrosis, cirrhosis and increased expression of cytokine expression upon aging.	17601987; 35354799; 34630435	▼/promotes glucose intolerance, hepatic steatosis, inflammation and fibrosis
Dusp16	DUSP16	-2.001454627	0.000251049	Yes	DUSP16 suppression accelerates dyslipidemia and inflammation in palmitate-treated hepatocytes. DUSP16 over-expression has the inverse phenotype, and DUSP16 knockout promotes glucose intolerance/IR, hepatic steatosis and inflammation in HFD-fed mice due to promoted lipogenesis and FA uptake and decreased oxidation.	31982140	▼/promotes glucose intolerance, IR, hepatic steatosis and inflammation
Cox10	COX10	-2.098539777	7.72E-05	Yes	Cox10 KO mice had severe liver dysfunction, increased mitochondrial proliferation and hepatic steatosis	17951359	▼/promotes hepatic steatosis
Ctse	CTSE	-2.260194175	0.001580279	Not directly	Ctse KO mice showed defective adipose tissue development which promoted hepatic steatosis and hypercholesterolemia under HFD for 24w.	24583126	▼/promotes hepatic steatosis
Colgalt2	COLGALT2	-3.267861364	7.83E-05	Yes	Total Colgalt2 KO has increased lipodystrophy and promotes hepatic steatosis and steatohepatitis induced by HFD or MCD	33865898	▼/promotes hepatic steatosis and inflammation
Ppard	PPARD	-2.136572473	7.83E-05	yes	Injection of Ppar· agonist and overexpression in vivo showed ammelioration of hepatic steatosis and plasma TGs. Reconstitution of Ppar· WT mice with bone marrow from Ppar· KO mice indicated decreased activated macrophages following HFD for 22weeks, but worsen hepatic steatosis due to decreased expression of FA oxidation and OXPHOS genes.	18024853; 18522831	▼/promotes hepatic steatosis but restrains macrophage activation
Erbb4	ERBB4	-3.17562217	0.00051304	Not directly	ErbB4 is activated by Nrg4 which leads to inhibits de novo lipogenesis, hepatic steatosis and ammeliorates IR; ErbB4 KO mice fed medium fat diet for 24w had aggravated obesity, dyslipidemia, hepatic steatos, hyperglycemia and hyperinsulinemia + IR.	25401691; 29944391	▼/promotes hepatic steatosis, glucose intolerance and IR
Tnfaip3	TNFAIP3	-2.078739395	0.000769763	Yes	Liver-specific Tnfaip3 KO had promoted BW gain, hepatic steatosis, glucose intolerance, insulin resistance and inflammation after 24w of HFD due to overactivation of ASK1/p38/JNK signalling	29227477	▼/promotes hepatic steatosis, glucose intolerance, IR and inflammation
Erbb3	ERBB3	-2.60601946	4.21E-05	Yes	Hepatocyte specific ERBB3 KO showed attenuated fibrosis in a CCl4-induced liver injury mouse model . It's silencing in vitro agravates steatosis and inflammation/oxidative stress due to increased TG levels, IL-6 and TNF-a by decreasing PI3K/AKT signalling .	27586651; 33541789	▼/promotes hepatic steatosis, inflammation/oxidative stress
Igfbp3	IGFBP3	-2.019231741	0.000125435	Yes, but not in vivo	Silencing IGFBP-3 in Huh7 cells enhanced JNK and NF-κB activity and increased palmitate-induced IL-8 secretion. Under lipotoxic conditions, palmitate inhibits hepatic macrophage secretion of IGFBP-3, thus enhancing palmitate-induced IL-8 synthesis and secretion.	22475139; 27553225	▼/promotes inflammation

Rbbp4	RBBP4	-2.196823559	4.68E-05	yes, but no mechanistic in vivo	Rbbp4 silencing inhibited fatty acid oxidation and promoted lipid accumulation in AML12 cells	35637971	▼/promotes lipid accumulation
Cndp2	CNDP2	-2.970564974	4.60E-05	Yes	CNDP2 KO are more prone to acetaminophed overdose-induced liver and renal injury due to potential increase of oxidative stress	34324979	▼/Promotes oxidative stress (inflammation)
Adam28	ADAM28	-5.133112725	0.000368588	Yes	Downregulation Adam28 in mice led to attenuation of BW gain, insulin resistance/Glucose intolerance and liver injury induced by HFD for 10weeks	28430139	▼/Restrains hepatic steatosis, hypeglycemia and IR
Hdac9	HDAC9	-2.73684791	0.000886702	Yes	Hdac9 KO mice are protected from BW gain, AT dysfunction and hepatic steatosis induced by HFD diet for 12 weeks. HDAC9 upregulation linked to increased hepatic gluconeogenesis induced by HCV infection due to regulation of CREB, PGC-1a, glucocorticoid receptor.	24101673; 28733598; 26420860	▼/Restrains hepatic steatosis; ▲/Promotes gluconeogenesis
Icam1	ICAM1	-2.230915347	0.008378443	yes	Icam KO mice challenged with ethanol had decreased graft injury upon transplantation into WT mice due to decreased leukocyte adherence and ROS production	22778492	▼/restrains inflammation and immune cell infiltration
Gpc4	GPC4	-2.008893903	0.004017722	yes, but no mechanistic in vivo	Linked with impaired glucose tolerance (increased expression) and T2D (decreased expression).	25240528	Inconclusive.
Hs6st2	HS6ST2	-3.948616021	2.73E-06	No			
Kctd20	KCTD20	-3.570798696	2.73E-06	No			
Tspan14	TSPAN14	-4.470437078	2.73E-06	No			
Plekhb1	PLEKHB1	-4.120678396	7.33E-06	No			
Myrf	MYRF	-3.013570954	7.34E-06	No			
Elp5	ELP5	-2.735917437	9.59E-06	No			
Map7d1	MAP7D1	-3.04932509	9.59E-06	No			
Rgs2	RGS2	-2.570315811	9.59E-06	No			
Tead2	TEAD2	-2.549061538	1.11E-05	No			
Zdhhc18	ZDHHC18	-2.33230941	1.16E-05	No			
Rnf2	RNF2	-2.612306594	1.66E-05	No			
Thyn1	THYN1	-2.331387512	1.75E-05	No			
Capn15	CAPN15	-2.284620987	1.78E-05	No			
Cxadr	CXADR	-2.224655086	1.80E-05	No			
Gpr37	GPR37	-2.760443859	1.80E-05	No			
Ap5m1	AP5M1	-2.401777747	1.96E-05	No			
Adam23	ADAM23	-2.417580688	2.04E-05	No			
Aak1	AAK1	-2.617391387	2.16E-05	No			
Pde3a	PDE3A	-2.492622412	2.48E-05	Not directly			
Cdk17	CDK17	-2.425987367	2.52E-05	No			
Flrt2	FLRT2	-3.071625083	2.61E-05	No			
Mrpl17	MRPL17	-2.346159029	2.61E-05	No			
Mxra8	MXRA8	-2.067606884	2.61E-05	No			
Ralbp1	RALBP1	-2.144718397	2.61E-05	No	It was shown to be involved in endocytosis of EGFR/Insulin Receptor in the liver by interacting with ARIP2	11882656	
Fhdc1	FHDC1	-3.229847224	2.61E-05	No			
Ranbp3l	RANBP3L	-3.600393248	2.67E-05	No			
Panx1	PANX1	-2.68137986	2.73E-05	No			
Tuba1a	TUBA1A	-3.487831395	2.75E-05	No			
Ngrn	NGRN	-2.082100668	2.90E-05	No			
Ttyh1	TTYH1	-7.475746216	2.98E-05	No			
Ebp	EBP	-2.5121591	3.36E-05	no			
Igdcc3	IGDCC3	-2.298801684	3.45E-05	No			
Gpr161	GPR161	-2.254752536	3.46E-05	No			
Glipr1	GLIPR1	-5.026245328	3.94E-05	No			
B3galt1	B3GALT1	-4.23968167	4.09E-05	no			
Hspb8	HSPB8	-3.081796277	4.09E-05	No			
Lmtk2	LMTK2	-2.391513509	4.21E-05	No			
Fam98a	FAM98A	-2.059328085	4.68E-05	No			
Mex3a	MEX3A	-2.448894354	5.04E-05	Yes	Nothing related to MASLD		
Rere	RERE	-2.045319459	5.57E-05	no			
Atp8b2	ATP8B2	-2.069426313	5.70E-05	Yes	Nothing related to MASLD		
Sema4g	SEMA4G	-2.00272875	5.92E-05	No			
Arhgef18	ARHGEF18	-2.491424675	6.01E-05	No			
Wsb1	WSB1	-2.075341478	6.42E-05	Yes	Nothing related to MASLD		
Rab3b	RAB3B	-2.408227029	7.21E-05	Yes	Identified as potential markers of oval cells (proilferating on the periportal region) in liver injury induced by CCL4 injection. No function described	16781709	
Prl3c1	PRL	-5.319486765	7.72E-05	No			
Cnnm1	CNNM1	-2.179426537	7.83E-05	No			
Dlg5	DLG5	-2.260874936	7.83E-05	No			
Tsc22d3	TSC22D3	-3.432804118	7.83E-05	Yes	Nothing related to MASLD		
Cnn1	CNN1	-3.651308593	8.00E-05	no			
Cdh6	CDH6	-3.658050741	8.24E-05	No			
Jade3	JADE3	-2.30523696	8.40E-05	No			
Itga2	ITGA2	-4.256480782	8.42E-05	No			
Rftn2	RFTN2	-3.902104345	8.59E-05	No			
Gp2	GP2	-4.172622075	8.61E-05	No			
Bmf	BMF	-2.060841067	8.70E-05	No			
Nexn	NEXN	-3.179458096	9.95E-05	No			
Scrn1	SCRN1	-2.19565101	9.95E-05	No			

Rnd1	RND1	-2.665841368	0.000100186	Yes	Nothing related to MASLD			
Osbp2	OSBP2	-3.18225983	0.000101773	No				
Dido1	DIDO1	-2.086626744	0.000105134	No				
Slc37a1	SLC37A1	-2.681815781	0.000107186	No				
Cdr2l	CDR2L	-2.207369067	0.000108352	No				
Rassf10	RASSF10	-6.290982745	0.000113662	Yes, but not in vivo				
Foxp4	FOXP4	-2.022171434	0.00012542	Not in vivo				
Rtn4rl1	RTN4RL1	-2.559756647	0.000139092	no				
Rnf122	RNF122	-2.188737062	0.000140611	No				
Ift81	IFT81	-2.173449113	0.000149415	No				
Scube3	SCUBE3	-5.940129265	0.000152796	No				
Mink1	MINK1	-2.063249122	0.000154002	no				
F2rl2	F2RL2	-4.503198881	0.000167976	No				
Vsig1	VSIG1	-16.48842458	0.000170858	No				
Hepacam2	HEPACAM2	-2.383656316	0.00017237	No				
Dennd2a	DENND2A	-2.731666058	0.000174548	Yes	Nothing related to MASLD			
Adamts12	ADAMTS12	-3.464673585	0.000175227	No				
Mmp11	MMP11	-2.115276424	0.00017956	Yes	Nothing related to MASLD			
Prtg	PRTG	-2.136180642	0.00019379	No				
Asb2	ASB2	-4.575918532	0.000217317	No				
Rab30	RAB30	-2.425223794	0.000250151	No	Investigation of the hepatocyte PPAR³ as a central regulator of gene expression during starvation in Ppar³hep−/− mice. Novel PPAR³-sensitive genes, including Rab30, were identified.	28774777		
Hcn4	HCN4	-2.978505426	0.000282511	No				
Ano3	ANO3	-2.262677592	0.000283771	No				
Brinp3	BRINP3	-2.023167663	0.000283771	No				
Galnt16	GALNT16	-2.11201783	0.000340639	Yes	Nothing related to MASLD			
Sdc3	SDC3	-2.032640063	0.000346589	Yes	Nothing related to MASLD			
Myo7a	MYO7A	-2.827044434	0.000348397	No				
Svil	SVIL	-2.017056422	0.000370508	Yes	Nothing related to MASLD			
Nostrin	NOSTRIN	-2.783890067	0.000377466	No				
Dpysl5	DPYSL5	-2.411555538	0.000382746	no				
Rcsd1	RCSD1	-10.25076473	0.000421213	No				
Slc7a1	SLC7A1	-2.285191524	0.000477855	No				
Spsb4	SPSB4	-2.411821351	0.000517391	No				
Emp3	EMP3	-2.131507992	0.000524806	Yes	Nothing related to MASLD			
Specc1	SPECC1	-2.019666796	0.000528435	No				
Brinp2	BRINP2	-4.121775372	0.000530223	No				
Pde5a	PDE5A	-2.267949365	0.000555725	No				
B4galnt3	B4GALNT3	-2.415823124	0.000557621	No				
Tceal6	TCEAL3	-3.770894484	0.000569501	No				
Bbc3	BBC3	-2.990696653	0.000621395	Yes	Nothing related to MASLD			
Tacc2	TACC2	-2.384695394	0.000699557	Yes	Nothing related to MASLD			
Kcnj16	KCNJ16	-2.986941414	0.000740193	No				
Ank3	ANK3	-2.031503902	0.000769763	Yes	Nothing related to MASLD			
Dnajc18	DNAJC18	-2.166756463	0.000840293	No				
Cgnl1	CGNL1	-2.149221203	0.000853627	No				
Rab27b	RAB27B	-2.578455871	0.000895995	No				
Map1a	MAP1A	-3.073838446	0.000909111	No				
Nptxr	NPTXR	-2.304663398	0.000923771	No				
Stox2	STOX2	-3.92072954	0.001013121	No				
Myof	MYOF	-2.005326485	0.001067256	No				
Gbp2b	GBP2	-4.510891331	0.001085303	Yes	Nothing related to MASLD			
Cpa4	CPA4	-2.867942514	0.001299648	Not in vivo				
Antxr2	ANTXR2	-2.181123712	0.001304411	No				
Lrrn3	LRRN3	-6.102535465	0.001564151	No				
Vil1	VIL1	-2.245690458	0.001600075	No				
Etv1	ETV1	-2.034243104	0.001601538	Yes	Nothing related to MASLD			
Ptges3l	PTGES3L	-2.299962138	0.001625432	No				
Muc6	MUC6	-5.789257104	0.001708099	Yes	Nothing related to MASLD			
Capn8	CAPN8	-5.883852037	0.001820243	No				
Pou2af1	POU2AF1	-2.124602303	0.001978897	No				
Plb1	PLB1	-4.101627122	0.002056047	No				
Styk1	STYK1	-2.199580183	0.002167025	No	Specific marker of NK cells and NK1.1 cells (like liver type 1 innate lymphoid cells)	30690705		
Gprc5a	GPRC5A	-2.761808847	0.002565399	Yes	Nothing related to MASLD			
Dgkg	DGKG	-2.098495135	0.002806547	yes, but no mechanistic in vivo	Missense in DGKG linked with hepatic fibrinogen disease in cattle	37681469		
Hey2	HEY2	-2.171307158	0.00281202	No				
Fibin	FIBIN	-2.352046492	0.002823929	No				
Mboat4	MBOAT4	-2.880122602	0.002831593	No				
Susd5	SUSD5	-4.828788396	0.002973325	No				
Arhgef2	ARHGEF2	-2.058949087	0.002995813	No				
Dpysl2	DPYSL2	-2.263930899	0.002995813	Yes	Nothing related to MASLD			
Krt15	KRT15	-4.315569652	0.003082229	Yes	Nothing related to MASLD			
Rassf6	RASSF6	-2.588169189	0.003482925	no				
Wfikkn1	WFIKKN1	-2.221117687	0.00349677	No				
Btbd9	BTBD9	-2.073276609	0.003693959	No				
Cdh16	CDH16	-5.497554657	0.00391567	No				
Rab37	RAB37	-2.031687521	0.003994348	No				
Dchs1	DCHS1	-2.01837919	0.00399677	No				

S100a3	S100A3	-4.917760738	0.004142494	No			
Otogl	OTOGL	-2.625802408	0.004418841	No			
Enam	ENAM	-2.292468599	0.004854335	No			
Camk2a	CAMK2A	-4.663686164	0.005218051	No			
Mgat3	MGAT3	-4.399752935	0.005358924	Yes	Nothing related to MASLD		
Dll4	DLL4	-2.22407864	0.005360889	Yes	Myeloid DLL4 Does Not Contribute to the Pathogenesis of Non-Alcoholic Steatohepatitis in Ldlr-/- Mice	2789869	
Crispld1	CRISPLD1	-2.036710374	0.005539268	No			
Fut1	FUT1	-3.130940075	0.005902644	Yes	Nothing related to MASLD		
C6	C6	-2.203409329	0.006105007	No			
Plch1	PLCH1	-2.105365656	0.006164872	No			
Dusp13	DUSP13	-3.661537322	0.007553091	No			
Camkv	CAMKV	-2.245136356	0.008646565	No			
P2rx6	P2RX6	-2.796269858	0.008676974	no			
Ina	INA	-2.290729239	0.008893037	No			
Pcdhgb6	PCDHGB6	-3.180381561	0.009065125	No			
Lrp1b	LRP1B	-2.227509788	0.010096001	Yes, but not in vivo	Nothing related to MASLD		
Paqr8	PAQR8	-4.008813239	0.010132316	No			
Arhgap31	ARHGAP31	-3.794313033	0.010833389	No			
Tbc1d32	TBC1D32	-2.009129395	0.01096634	No			
Cga	CGA	-2.567377945	0.011237754	No			
Filip1	FILIP1	-3.43847481	0.011832999	no			
Dnah11	DNAH11	-2.724339649	0.01414795	no			
Ank1	ANK1	-3.352095178	0.019125194	No			
Birc3	BIRC3	-5.965854685	0.020570248	Yes	They found significant overexpression of Birc3 in alcoholic steatohepatitis and steatosis but not in MASH but no in vivo results or mechanistic explanation.	29307797	
Tmem154	TMEM154	-2.235002544	0.023107388	No			
Itgb6	ITGB6	-2.852535817	0.023857372	No			
Unc13d	UNC13D	-2.099334225	0.02995786	No			
Pcdhgb1	PCDHGB1	-2.328204141	0.033411352	No			
Chrdl2	CHRDL2	-2.120646749	0.034793546	No			
Mlf1	MLF1	-2.402139369	0.036688503	no			
Dnah17	DNAH17	-2.108037011	0.037914769	Yes	Nothing related to MASLD		
Srcin1	SRCIN1	-2.215911773	0.039688971	Not in vivo	Srcin1 was shown to inhibit NFkB activity and expression of PTN (Target gene), which promotes EMT, mestastasis and angiogenesis In HBV-related HCC.	29928866	
Mrap2	MRAP2	-2.662592918	0.042310818	Yes	Nothing related to MASLD		
Gng2	GNG2	-3.139187407	0.043329519	No			
Pkhd1l1	PKHD1L1	-2.632161724	0.044501998	no			
C1qtnf1	C1QTNF1	-2.463377434	0.044654249	No			
Srpx	SRPX	-2.480055729	0.045782422	No			

Table S2 – List of studies reporting association between different pathologies and miR-149 single nucleotide polymorphisms (rs71428439 and rs2292832) affecting miR-149 expression/maturation. In bold it is highlighted reports where variants were linked with non-alcoholic fatty liver disease and hepatocellular carcinoma.

Variant ID	Effect	Disease	PMID
rs71428439	AA carriers: Increased maturation of miR-149 linked with decreased apoptosis of cardiomyocytes	Myocardial infarction	23873935
	GG carriers: increased risk to develop renal carcinoma due to decreased miR-149 expression	Clear Renal Cell Carcinoma	25213695
	GG carriers: decreased risk do develop ischemic stroke	Ischemic Stroke	25867405
	GG carriers: increased risk to develop hepatocellular carcinoma due to downregulation of miR-149 and increased expression of AKT1	**Hepatocellular Carcinoma**	26550305
	associated with lung cancer prevalence	Lung cancer	30069329
	AA carriers: decreased risk to develop extrapulmonary tuberculosis	Extrapulmonary tuberculosis	31219360
rs2292832	TT carriers: increased risk to develop breast cancer and linked with decreased maturation of miR-149	Breast Cancer	36371555
	TT carriers: decreased risk to develop acute lymphoblastic leukemia	Acute Lymphoblastic Leukemia	35690283
	CC carriers: improved survival rate in patients with hepatocellular carcinoma with bone metastasis	**Hepatocellular Carcinoma**	35672274
	CC carriers: decreased risk to develop cervical cancer	Cervical Cancer	35495171
	CC carriers: increased risk to develop coronary artery aneurysm	Kawasaki disease	34969167
	CC carriers: increased risk to develop thyroid cancer	Thyroid Cancer	34643920; 25405731
	CC carriers: increased risk to develop endometriosis	Endometriosis	33583336
	associated with earlier onset of stroke	Ischemic Stroke	31811586
	CC carriers: increased risk to develop extrapulmonary tuberculosis	Extrapulmonary tuberculosis	31219360
	possible link with risk to develop gastroinstestinal cancers	Gastrointestinal cancers	30930933
	CC carriers: increased risk to develop cervical cancer and linked with increased maturation of miR-149	Cervical Cancer	30852614
	TT carriers: increased risk to develop colorectal cancer	Colorectal Cancer	30447914 27706637 24568449
	TT carriers: increased risk to develop gastric cancer	Gastric cancer	30274913 23001871
	CC carriers: increased risk to develop ischemic stroke	Ischemic Stroke	30254431 26690224
	CC carriers: increased risk to develop hepatocellular carcinoma in women	**Hepatocellular Carcinoma**	30215231
	decreased risk to develop HBV-related hepatocellular carcinoma	**Hepatocellular Carcinoma**	29976775
	CC carriers: later onset and milder symptoms of Charcot-Marie-Tooth disease type 1A	Charcot-Marie-Tooth disease type 1A	29729827
	CC carriers: increased risk to develop allergic rhinits and asthma	Allergic Rhinitis and Asthma	28181414
	CC carriers: improved survival rate in patients with non-small cell lung cancer	Non-small cell Lung Cancer	27825117 23470291
	TT carriers: increased risk to develop coronary heart disease	Coronary Heart Disease	27430349
	TT carriers: increased risk to develop inflammatory bowel disease	Inflammatory Bowel Disease	27109937
	CC carriers: increased disease-free survival in squamous cell carcinoma of the nonoropharynx	Squamous Cell Carcinoma	27050146
	TT carriers: increased risk to develop HBV-associated hepatocellular carcinoma	**Hepatocellular Carcinoma**	25190221
	CC carriers: increased risk for disease progression in nasopharyngeal carcinoma	Nasopharyngeal Carcinoma	24648993
	TT carriers: increased risk for disease progression and worse prognosis in head and neck squamous cell carcinoma	Head and Neck Squamous Cell Carcinoma	23272122
	TT carriers: primary hepatocytes increased miR-149 expression in response to fatty acid treatment, possibly link with NAFLD risk	**Non-Alcoholic Fatty Liver Disease**	28507283

Supplementary references

1. **Hansen HH**, Feigh M, Veidal SS, Rigbolt KT, Vrang N, Fosgerau K. Mouse models of nonalcoholic steatohepatitis in preclinical drug development. Drug Discov Today 2017;22:1707-1718.
2. **Berthou F**, Sobolewski C, Abegg D, Fournier M, Maeder C, Dolicka D, Correia de Sousa M, et al. Hepatic PTEN Signaling Regulates Systemic Metabolic Homeostasis through Hepatokines-Mediated Liver-to-Peripheral Organs Crosstalk. Int J Mol Sci 2022;23.
3. **Sobolewski C**, Abegg D, Berthou F, Dolicka D, Calo N, Sempoux C, Fournier M, et al. S100A11/ANXA2 belongs to a tumour suppressor/oncogene network deregulated early with steatosis and involved in inflammation and hepatocellular carcinoma development. Gut 2020;69:1841-1854.
4. **0uchi R**, Togo S, Kimura M, Shinozawa T, Koido M, Koike H, Thompson W, et al. Modeling Steatohepatitis in Humans with Pluripotent Stem Cell-Derived Organoids. Cell Metab 2019;30:374-384 e376.
5. **Li Q**, Dong Z, Lian W, Cui J, Wang J, Shen H, Liu W, et al. Ochratoxin A causes mitochondrial dysfunction, apoptotic and autophagic cell death and also induces mitochondrial biogenesis in human gastric epithelium cells. Arch Toxicol 2019;93:1141-1155.
6. **Calo N**, Ramadori P, Sobolewski C, Romero Y, Maeder C, Fournier M, Rantakari P, et al. Stress-activated miR-21/miR-21* in hepatocytes promotes lipid and glucose metabolic disorders associated with high-fat diet consumption. Gut 2016;65:1871-1881.
7. **Mithieux G**, Guignot L, Bordet JC, Wiernsperger N. Intrahepatic mechanisms underlying the effect of metformin in decreasing basal glucose production in rats fed a high-fat diet. Diabetes 2002;51:139-143.
