# Supplementary material for: Hepatic miR-149-5p upregulation fosters steatosis, inflammation and fibrosis development in mice and in human liver organoids
Source: JHEP Rep. 2024 Jun 4;6(9):101126. doi: 10.1016/j.jhepr.2024.101126 (PMC11388170; doi:10.1016/j.jhepr.2024.101126)
Supplement: Multimedia component 2 [file mmc2.docx]

**JHEP Reports**

**CTAT methods**

Tables for a “Complete, Transparent, Accurate and Timely account” (CTAT) are now mandatory for all revised submissions. The aim is to enhance the reproducibility of methods.

- Only include the parts relevant to your study
- Refer to the CTAT in the main text as ‘Supplementary CTAT Table’
- Do not add subheadings
- Add as many rows as needed to include all information
- Only include one item per row

**If the CTAT form is not relevant to your study, please outline the reasons why:**

|  |
| --- |

- 1. **Antibodies**

| **Name** | **Citation** | **Supplier** | **Cat no.** | **Clone no.** |
| --- | --- | --- | --- | --- |
| phospho AKT Ser | PMID: 37038247 | Cell signaling | #9271 | Polyclonal |
| Total AKT | PMID: 37038247 | Cell signaling | #9272 | Polyclonal |
| Tubulin | PMID: 38001062 | Cell signaling | #2128 | Polyclonal |
| HNF4a | PMID: 17924446 | Santa-Cruz | SC-8987 | Polyclonal |
| Phospho JNK | PMID: 37598316 | Cell signaling | #9251 | Polyclonal |
| Total JNK | PMID: 37598316 | Cell signaling | #9252 | Polyclonal |
| Phospho NFκB | PMID: 36523164 | Cell signaling | #3037 | Polyclonal |
| Total NFκB | PMID: 26538602 | Santa-Cruz | SC-372 | Polyclonal |
| Phospo p38 | PMID: 36999351 | Cell signaling | #4511 | Polyclonal |
| p38 | PMID: 36999351 | Cell signaling | #8690 | Polyclonal |
| ERM | PMID: 36572741 | Cell signaling | #3142 | Polyclonal |
| PDGFR | PMID: 32805235 | abcam | ab32570 | Polyclonal |
| HNF4 - Alexa Fluor 555 |  | abcam | ab217518 | EPR16786 |
| HNF4 - Alexa Fluor 647 |  | abcam | ab217073 | EPR3648 |
| CD166 | PMID: 36499099 | abcam | ab109215 | EPR2759(2) |
| CD166 - Alexa Fluor 488 |  | abcam | ab197543 | EPR2759(2) |
| CD68 - Alexa Fluor 647 | PMID: 36198439 | abcam | ab213363 | EPR20545 |
| CD68 - Alexa Fluor 647 | PMID: 33778787 | abcam | ab224029 | EPR20545 |
| Iba1 | PMID: 36410420 | abcam | ab178846 | EPR16588 |
| GFP | PMID: 38039321 | Cell signaling | #2956 | D5.1 |

- 1. **Cell lines**

| **Name** | **Citation** | **Supplier** | **Cat no.** | **Passage no.** | **Authentication test method** |
| --- | --- | --- | --- | --- | --- |
| Huh7 | Nakabayashi H, et  al. Cancer Res.  1982;42:3858 | JCBR Cell Bank and Sekisui XenoTech | JCRB0403 | 9-25 |  |
| Human embryonic stem cells (HS420) | Main H, et al. Karolinska Institutet Human Embryonic Stem Cell Bank. Stem Cell Res. 2020 | Karolinska Institute, Stockholm, Sweden | BAG-hES-IMP-0046 | 20-45 |  |

- 1. **Organisms**

| **Name** | **Citation** | **Supplier** | **Strain** | **Sex** | **Age** | **Overall n number** |
| --- | --- | --- | --- | --- | --- | --- |
| Mus musculus |  | Charles River | C57BL/6J | Males | Bellow 35 weeks | 76 |
| Mus musculus |  | Foti Lab | C57BL/6J  *Pten*^lox/lox^, AlbCre^+/-^ | Males | Bellow 16 weeks | 9 |
| Mus musculus |  | Foti Lab | C57BL/6J  *Pten*^lox/lox^, AlbCre^-/-^ | Males | Bellow 16 weeks | 9 |

- 1. **Sequence based reagents**

| **Name** | **Sequence** | **Supplier** |
| --- | --- | --- |
| miR-149 | TCT GGC TCC GTG TCT TCA CT | Microsynth Switzerland |
| miR-182 | TTT GGC AAT GGT AGA ACT CAC AC | Microsynth Switzerland |
| miR-183 | TAT GGC ACT GGT AGA ATT CAC TAA | Microsynth Switzerland |
| miR-122 | GGCTGTGGAGTGTGACAATG | Microsynth Switzerland |
| miR-16 | ACAGCCTAGCAGCACGTAAAT | Microsynth Switzerland |
| univ-RT-polyT | GAGGTATTCGCACCAGAGGATTTTTTTTTTTTTTTVN | Microsynth Switzerland |
| *Vim FW* | CGG CTG CGA GAG AAA TTG C | Microsynth Switzerland |
| *Vim REV* | CCA CTT TCC GTT CAA GGT CAA G | Microsynth Switzerland |
| *Fn1 FW* | ATCTCGGAGCCATTTGTTCCT | Microsynth Switzerland |
| *Fn1 REV* | CCAGGTCTACGGCAGTTGTCA | Microsynth Switzerland |
| *Col1a1 FW* | GCT CCT CTT AGG GGC CAC T | Microsynth Switzerland |
| *Col1a1 REV* | CCA CGT CTC ACC ATT GGG G | Microsynth Switzerland |
| *Acta FW* | AAAAAAAACCACGAGTAACAAATCAA | Microsynth Switzerland |
| *Acta REV* | TCAGCGCCTCCAGTTCCT | Microsynth Switzerland |
| *Pdgfrb FW* | GAGGCTTATCCGATGCCTTCT | Microsynth Switzerland |
| *Pdgfrb REV* | AGACATGTTGCGAGTAGACAAAACTAA | Microsynth Switzerland |
| *Krt18 FW* | CAG CCA GCG TCT ATG CAG G | Microsynth Switzerland |
| *Krt18 REV* | CCT TCT CGG TCT GGA TTC CAC | Microsynth Switzerland |
| *Il6 FW* | AGT TGC CTT CTT GGG ACT GAT | Microsynth Switzerland |
| *Il6 REV* | TCC ACG ATT TCC CAG AGA AC | Microsynth Switzerland |
| *Il10 FW* | CTTTCAAACAAAGGACCAGC | Microsynth Switzerland |
| *Il10 REV* | CCAAGTAACCCTTAAAGTCCT | Microsynth Switzerland |
| *Il1b FW* | GACAACTGCACTACAGGC | Microsynth Switzerland |
| *Il1b REV* | CATGGAGAATATCACTTGTTGG | Microsynth Switzerland |
| *Tgfb FW* | CAACATGTGGAACTCTACCAG | Microsynth Switzerland |
| *Tgfb REV* | TGTATTCCGTCTCCTTGGT | Microsynth Switzerland |
| *Tnfa FW* | AGGCTGCCCCGACTACGT | Microsynth Switzerland |
| *Tnfa REV* | GACTTTCTCCTGGTATGAGATAGCAAA | Microsynth Switzerland |
| *Itgam FW* | ATG GAC GCT GAT GGC AAT ACC | Microsynth Switzerland |
| *Itgam REV* | TCC CCA TTC ACG TCT CCC A | Microsynth Switzerland |
| *Fgf21 FW* | CAGTCCAGAAAGTCTCCTG | Microsynth Switzerland |
| *Fgf21 REV* | GATCAAAGTGAGGCGATCC | Microsynth Switzerland |
| *Cd36 FW* | GTCTATCTACGCTGTGTTCG | Microsynth Switzerland |
| *Cd36 REV* | ACAGGCTTTCCTTCTTTGC | Microsynth Switzerland |
| *Acc1 FW* | GGACACCAGTTTTGCATTGA | Microsynth Switzerland |
| *Acc1 REV* | AGTTTGGGAGGACATCGAAA | Microsynth Switzerland |
| *Fasn FW* | AAGTTGCCCGAGTCAGAGAACC | Microsynth Switzerland |
| *Fasn REV* | ATCCATAGAGCCCAGCCTTCCATC | Microsynth Switzerland |
| *Cpt1a FW* | ATGGCAGAGGCTCACCAAGC | Microsynth Switzerland |
| *Cpt1a REV* | GATGAACTTCTTCTTCCAGGAGTGC | Microsynth Switzerland |
| *Acox1 FW* | CATGAATCCCGATCTGCG | Microsynth Switzerland |
| *Acox1 REV* | TCAAGTTCTCGATTTCTCGAC | Microsynth Switzerland |
| *Fabp4 FW* | CACCGAGATTTCCTTCAAACTG | Microsynth Switzerland |
| *Fabp4 REV* | TTTCATAACACATTCCACCACC | Microsynth Switzerland |
| *Fatp5 FW* | TACAAGTTGGAGCCACCTG | Microsynth Switzerland |
| *Fatp5 REV* | TCACCCACATACAAGATCACTG | Microsynth Switzerland |
| *Hmgcr FW* | GTACATTCTGGGTATTGCTGG | Microsynth Switzerland |
| *Hmgcr REV* | GCACTCGCTCTAGAAAGG | Microsynth Switzerland |
| *NANOG FW* | TTT GTG GGC CTG AAG AAA ACT | Microsynth Switzerland |
| *NANOG REV* | AGG GCT GTC CTG AAT AAG CAG | Microsynth Switzerland |
| *CDX2 FW* | GAC GTG AGC ATG TAC CCT AGC | Microsynth Switzerland |
| *CDX2 REV* | GCG TAG CCA TTC CAG TCC T | Microsynth Switzerland |
| *FOXA2 FW* | AGCGGTGAAGATGGAAGG | Microsynth Switzerland |
| *FOXA2 REV* | GTGTTCATGCCGTTCATCC | Microsynth Switzerland |
| *HNF4A FW* | CTCCTGCAGATTTAGCCG | Microsynth Switzerland |
| *HNF4A REV* | CTGTCCTCATAGCTTGACC | Microsynth Switzerland |
| *ALB FW* | CTAGAGAAGTGCTGTGCC | Microsynth Switzerland |
| *ALB REV* | CCACGGATAGATAGTCTTCTG | Microsynth Switzerland |
| *SERPINA1 FW* | CTTCTTTAAAGGCAAATGGGAG | Microsynth Switzerland |
| *SERPINA1 REV* | CTGGACAGCTTCTTACAGTG | Microsynth Switzerland |
| *ALCAM FW* | TCC TGC CGT CTG CTC TTC T | Microsynth Switzerland |
| *ALCAM REV* | TTC TGA GGT ACG TCA AGT CGG | Microsynth Switzerland |
| *CD68 FW* | GGA AAT GCC ACG GTT CAT CCA | Microsynth Switzerland |
| *CD68 REV* | TGG GGT TCA GTA CAG AGA TGC | Microsynth Switzerland |
| *ACTA FW* | TGATCACCATCGGAAATGAA | Microsynth Switzerland |
| *ACTA REV* | CGGCTTCATCGTATTCCTGT | Microsynth Switzerland |
| *COL1A1 FW* | AACATGACCAAAAACCAAAAGTG | Microsynth Switzerland |
| *COL1A1 REV* | CATTGTTTCCTGTGTCTTCTGG | Microsynth Switzerland |
| *IL6 FW* | AAATTCGGTACATCCTCGACGG | Microsynth Switzerland |
| *IL6 REV* | GGAAGGTTCAGGTTGTTTTCTGC | Microsynth Switzerland |
| *IL8 FW* | CTGCGCCAACACAGAAATTA | Microsynth Switzerland |
| *IL8 REV* | ATTGCATCTGGCAACCCTAC | Microsynth Switzerland |
| *IL1B FW* | ATG GCT TAT TAC AGT GGC AA | Microsynth Switzerland |
| *IL1B REV* | GTC GGA GAT TCG TAG CTG GA | Microsynth Switzerland |
| *TGFB FW* | GTGACCTGGCCACCATTCAT | Microsynth Switzerland |
| *TGFB REV* | GTCAATGTACAGCTGCCGCA | Microsynth Switzerland |
| *VIM FW* | GCCCTAGACGAACTGGGTC | Microsynth Switzerland |
| *VIM REV* | GGCTGCAACTGCCTAATGAG | Microsynth Switzerland |

- 1. **Deposited data**

| **Name of repository** | **Identifier** | **Link** |
| --- | --- | --- |
| Yareta | 5897a11c-b524-4942-b420-890285a7b8c4 | doi:10.26037/yareta:lcl2chfvereufnbarebilj5yyq |

- 1. **Software**

| **Software name** | **Manufacturer** | **Version** |
| --- | --- | --- |
| Wave | Agilent Technologies | 2.4.0 |
| Gen5 | BioTek |  |
| CellProfiler | Broad Institute of MIT and Harvard | 4.2.1. |
| ImageJ | National Institutes of Health | 1.52u |
| GeneSys | Syngene, Synoptics group |  |
| QuPath |  | 0.4.3. |
| Matlab | Mathworks | R2023a |
| GraphPad Prism | GraphPad | 8 |
| InkScape |  | 0.92.3 |

- 1. **Other (*e.g*. drugs, proteins, vectors etc.)**

| AAV8-U6-scrambled-shRNA-GFP | VectorBiolabs, USA |  |
| --- | --- | --- |
| AAV8-U6-shRNA-miR-149-5p-GFP | VectorBiolabs, USA |  |
| miRIDIAN microRNA Mimic Negative Control #1 | Horizon Discovery, UK | CN-001000-01 |
| miRIDIAN microRNA miR-149-5p mimic | Horizon Discovery, UK | C-300631-07 |
| Seahorse XF Cell Mito Stress Test Kit | Agilent Technologies | 103015-100 |
| Seahorse XF Glycolytic Rate Assay Kit | Agilent Technologies | 103344-100 |
| Seahorse XF Long Chain Fatty Acid Oxidation Stress Test Kit | Agilent Technologies | 103672-100 |
| Hoechst | ThermoFisher Scientific | 33342 |
| 2-NBDG | ThermoFisher Scientific | N13195 |
| BODIPY | Molecular probes | D3922 |
| MitoTracker™ Red CMXRos | ThermoFisher Scientific | M7512 |
| QiAmp ® DNA Micro kit | Qiagen | 56304 |
| High-Capacity cDNA Reverse Transcription kit | ThermoFisher Scientific | 4368814 |
| PowerUp™ 5 Real-Time PCR System | ThermoFisher Scientific | A25742 |
| ECL Prime Substrate | Amersham | RPN22232 |
| DAB Substrate kit | Abcam | ab64238 |

- 1. **Please provide the details of the corresponding methods author for the manuscript:**

| Marta Correia de Sousa  Department of Cell Physiology and Metabolism  Faculty of Medicine, University of Geneva  Rue Michel-Servet 1  1211 Geneva, Switzerland  e-mail: marta.sousa@unige.ch |
| --- |
