# Supplementary material for: Hepatic miR-149-5p upregulation fosters steatosis, inflammation and fibrosis development in mice and in human liver organoids
Source: JHEP Rep. 2024 Jun 4;6(9):101126. doi: 10.1016/j.jhepr.2024.101126 (PMC11388170; doi:10.1016/j.jhepr.2024.101126)
Supplement: Multimedia component 4 [file mmc4.pdf]

# Hepatic miR-149-5p upregulation fosters steatosis, inflammation and fibrosis development in mice and in human liver organoids

Marta Correia de Sousa[1,*], Etienne Delangre[1], Flavien Berthou[1], Sanae El Harane[2], Christine Maeder[1], Margot Fournier[1], Karl-Heinz Krause[2], Monika Gjorgjieva[1], Michelangelo Foti[1,†]

JHEP Reports **2024**. vol. 6 | 1–14

Check for updates

**Background & Aims:** The incidence of metabolic dysfunction-associated steatotic liver disease (MASLD) is increasing worldwide. Alterations of hepatic microRNA (miRNA) expression/activity significantly contribute to the development and progression of MASLD. Genetic polymorphisms of miR-149 are associated with an increased susceptibility to MASLD development in humans. Aberrant expression of miR-149 was also associated with metabolic alterations in several organs, but the impact of hepatic miR-149-5p deregulation in MASLD remains poorly characterized.

**Methods:** MiR-149-5p was downregulated in the livers of mice by *in vivo* transduction with hepatotropic adeno-associated virus 8 harboring short-hairpin RNAs (shRNAs) specific for miR-149-5p (shmiR149) or scrambled shRNAs (shCTL). MASLD was then induced with a methionine/choline-deficient (MCD, n = 7 per group) diet or a fructose/palmitate/cholesterol-enriched (FPC, n = 8-12 per group, per protocol) diet. The impact of miR-149-5p modulation on MASLD development was assessed *in vivo* and *in vitro* using multi-lineage 3D human liver organoids (HLOs) and Huh7 cells.

**Results:** MiR-149-5p expression was strongly upregulated in mouse livers from different models of MASLD (2-4-fold increase in *ob/ob*, *db/db* mice, high-fat and FPC-fed mice). *In vivo* downregulation of miR-149-5p led to an amelioration of diet-induced hepatic steatosis, inflammation/fibrosis, and to increased whole-body fatty acid consumption. In HLOs, miR-149-5p overexpression promoted lipid accumulation, inflammation and fibrosis. *In vitro* analyses of human Huh7 cells overexpressing miR-149-5p indicated that glycolysis and intracellular lipid accumulation was promoted, while mitochondrial respiration was impaired. Translatomic analyses highlighted deregulation of multiple potential miR-149-5p targets in hepatocytes involved in MASLD development.

**Conclusions:** MiR-149-5p upregulation contributes to MASLD development by affecting multiple metabolic/inflammatory/fibrotic pathways in hepatocytes. Our results further demonstrate that HLOs are a relevant 3D *in vitro* model to investigate hepatic steatosis and inflammation/fibrosis development.

© 2024 The Author(s). Published by Elsevier B.V. on behalf of European Association for the Study of the Liver (EASL). This is an open access article under the CC BY license (http://creativecommons.org/licenses/by/4.0/).

## Introduction

High-calorie diets and a sedentary lifestyle are key factors driving obesity. The lipotoxicity associated with excessive fat accumulation in the liver causes metabolic dysfunction-associated steatotic liver disease (MASLD), a progressive liver disease currently affecting >30% of the global population.[1] MASLD represents a spectrum of hepatic disorders that starts with simple steatosis, where over 5% of hepatocytes accumulate lipids. The deregulated hepatic energetic metabolism and steatosis are tightly associated with insulin resistance (IR).[2] If unresolved, hepatic steatosis/IR can progress to more severe pathologies like type 2 diabetes and metabolic dysfunction-associated steatohepatitis (MASH), characterized by extensive liver inflammation, hepatocyte ballooning and intra-sinusoidal fibrosis.[3,4] The deregulation of microRNA (miRNA) expression and/or activity play a key role in the development and progression of multifactorial metabolic diseases.[5,6] Many miRNAs and their function in hepatic metabolic diseases remain poorly characterized but have potential clinical applications as biomarkers or as addressable targets to prevent disease progression.

MiRNAs are small double-stranded molecules processed and incorporated in the RISC (RNA-induced silencing complex), where one strand (guide) directs RISC to inhibit protein translation through mRNA decay or translational blockage.[7] Since one single miRNA can simultaneously target several mRNAs,[7,8] multiple physiopathological mechanisms are fine-tuned through modulation of several target genes in parallel. Consistent with this feature, deregulation of specific hepatic miRNAs was reported to

* Corresponding author. Address: Department of Cell Physiology and Metabolism, Faculty of Medicine, University of Geneva, Geneva, Switzerland.
*E-mail address:* marta.sousa@unige.ch (M. Correia de Sousa).
† Deceased.
https://doi.org/10.1016/j.jhepr.2024.101126

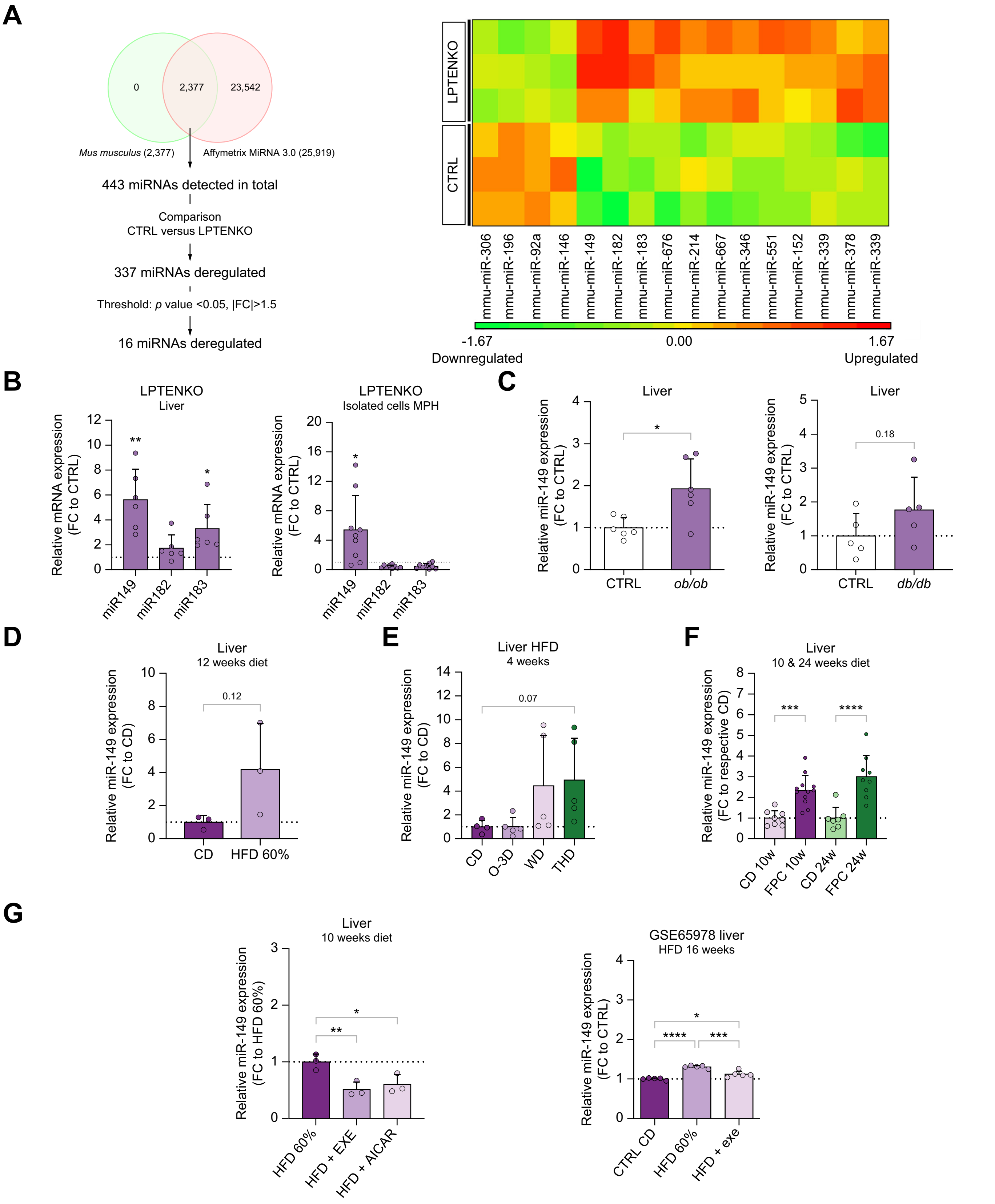


**Fig. 1. MiR-149-5p expression is consistently upregulated in several mouse models of MASLD and is modulated by AMPK activity.** (A) Experimental microarray pipeline (left) and heatmap representation of differentially expressed hepatic miRNAs (right) and (B) relative miRNA expression in hepatic tissues and in MPHs from 4-month-old mice (CTRL) and LPTENKO mice. Relative hepatic miR-149-5p expression in (C) leptin signaling-deficient (*ob/ob* and *db/db*) and in diet-induced obesity/ MASLD mouse models: (D) mice fed with CD or HFD, (E) mice fed with CD, HFD supplemented with O-3D or with THD or WD (FC to CD), (F) mice fed with CD or FPC diet. (G) Relative hepatic miR-149-5p expression in mice fed HFD submitted to exercise (HFD+EXE) or injected with AMPK-activators (HFD+AICAR) (left, FC to HFD)

affect multiple gene networks involved in glucose homeostasis, cholesterol and lipid metabolism, energy expenditure, lipogenesis and inflammation, thus contributing to MASLD and IR/type 2 diabetes development.[6,9,10] In this regard, upregulation of miR-149-5p was reported in a mouse model of diet-induced hepatic steatosis/IR[11] and alterations of the two miR-149 strands were linked with metabolic dysfunction in different organs/cells, *i.e.* adipose tissue (AT),[12,13] skeletal muscle,[14] cultured hepatic cancer cells,[15–17] and adipocytes.[18,19] Regarding the function of miR-149-5p in the liver, only fragmentary information is available. Depletion of miR-149 in mice increases sensitivity to acute liver injury induced by injection of lipopolysaccharides (LPS) or diethylnitrosamine, likely due to enhanced pro-inflammatory signaling pathways.[20,21] Finally, *in vitro* studies using human hepatoblastoma HepG2 cells,[15] mouse primary hepatocytes,[16] or mouse AML12 cells[17] suggested that miR-149-5p overexpression promoted intracellular lipid accumulation.

Herein, we investigated how miR-149-5p expression is modulated in the liver upon MASLD development in genetic and diet-induced mouse models, as well as the impact of deregulated expression of miR-149-5p on steatosis, inflammation and fibrosis development/progression in mice and in stem cell-derived multi-lineage 3D human liver organoids.

## Materials and methods

### Animals

Handling and experimental procedures were approved by the Geneva Health Head Office (authorization number GE/175) and performed following the Swiss guidelines for animal experimentation. Five-week-old males (C57BL6/J) were provided by Charles River Labs (France). Different mouse models of diet-induced MASLD/MASH were used: methionine/choline-deficient (MCD) and fructose/palmitate/cholesterol-enriched (FPC) diets. For downregulation of mir-149-5p, mice were injected retro-orbitally with adeno-associated virus 8 packed with short-hairpin (shRNA) specific for miR-149 (shmiR149) or scrambled shRNA (shCTL) and GFP reporter.

### *In vitro* models

Multi-lineage 3D human liver organoids (HLOs) and hepatic cell line Huh7 were transfected with synthetic oligonucleotides mimicking miR-149-5p. HLOs were used to investigate the impact of miR-149-5p overexpression on steatosis, inflammation and fibrosis development. Live metabolic characterization of Huh7 cells overexpressing miR-149-5p was performed with Seahorse MitoStress, GlycoRate and Substrate Oxidation Stress test (Agilent) and translatomic analyses were performed to identify potential miR-149-5p targets. Glucose uptake, lipid accumulation, mitochondrial function and insulin sensitivity was also assessed.

Detailed information on the materials and methods used are provided in the supplementary materials.

## Results

### MicroRNA-149-5p is upregulated in the liver of mice with steatosis

Microarray analysis of miRNA expression in liver tissues from 4-month-old liver-specific *Pten* knockout (LPTENKO) mice[22] and corresponding wild-type littermates was performed. From a total of 443 miRNAs detected by the microarray, only 16 (4 downregulated miRNAs and 12 upregulated miRNAs) were within the established threshold (Fig. 1A). The top three most upregulated miRNAs identified were selected for further validation by quantitative reverse-transcription PCR in additional biological samples (miR-149, miR-182, miR-183, Fig. 1B). Only miR-149-5p was strongly upregulated in liver tissues of LPTENKO mice and in isolated hepatocytes of LPTENKO mice. The modest upregulation of miR-183 observed originated from non-parenchymal cells (Fig 1B and Fig. S1A). Of note, a trend for an increased circulating level of miR-149-5p in the plasma of LPTENKO was also observed (Fig. S1B).

To determine whether miR-149-5p upregulation is a general feature in MASLD, miR-149-5p expression was assessed in the liver of different genetic or diet-induced MASLD murine models. Leptin signaling-deficient model of steatosis, diabetes and obesity (*ob/ob* and *db/db* mice) displayed a similar increase in hepatic miR-149-5p expression (Fig. 1C). Mice with diet-induced steatosis (high-fat diet – HFD; 60% fat) also had increased hepatic miR-149-5p levels (Fig. 1D). The nature of fatty acids was also a determinant for steatosis development and miR-149-5p upregulation. In mice fed a diet containing 45% fat, miR-149-5p was upregulated in mice with steatosis (fed with saturated or with trans-fatty acids but not with omega-3, Figs 1E and S2). Upregulation of hepatic miR-149-5p was also observed in mice fed a FPC diet for 10 and 24 weeks (Fig. 1F). Of note, hepatic miR-149-5p expression tends also to be upregulated in mice fed a MCD diet (Fig. S3A), which was further confirmed by the analysis of a gene expression omnibus (GEO) dataset (GSE55593) (Fig. S3B). Since miR-149-5p expression was previously reported to be modulated by exercise in skeletal muscle cells,[23] we examined hepatic miR-149-5p expression in mice fed a HFD and challenged with treadmill exercise.[24] Physical activity in mice fed a HFD (HFD+EXE) decreased steatosis,[24] as well as hepatic miR-149-5p expression (Fig. 1G, left panel). The same effect on miR-149-5p expression was observed in mice fed a HFD administered with AICAR (5-aminoimidazole-4-carboxamide ribonucleotide), a specific AMPK activator (HFD+AICAR; Fig. 1G, left panel). Similar observations were obtained with the analysis of a GEO dataset (GSE65978) of miRNA expression in mice fed a HFD and forced to exercise (HFD+exe) (Fig. 1G, right panel).

### Hepatocyte-specific microRNA-149-5p knock down decreases diet-induced steatosis, inflammation and fibrosis development

To assess the functional role of miR-149-5p on MASLD development/progression *in vivo*, miR-149-5p expression was

and in a gene expression omnibus dataset of mice fed CD or HFD 60%, forced to exercise or not (right). **p* <0.05, ***p* <0.01, ****p* <0.001, *****p* <0.0001. Unpaired t-test with Welch's correction or One-way ANOVA with Holm-Sidak's correction. CD, chow diet; FPC, fructose/palmitate/cholesterol-enriched diet; HFD, high-fat diet; LPTENKO, liver-specific *Pten* knockout; MASLD, metabolic dysfunction-associated steatotic liver disease; MPHs, murine primary hepatocytes; O-3D, omega 3 fatty acids; THD, trans-unsaturated fatty acids; WD, western diet.

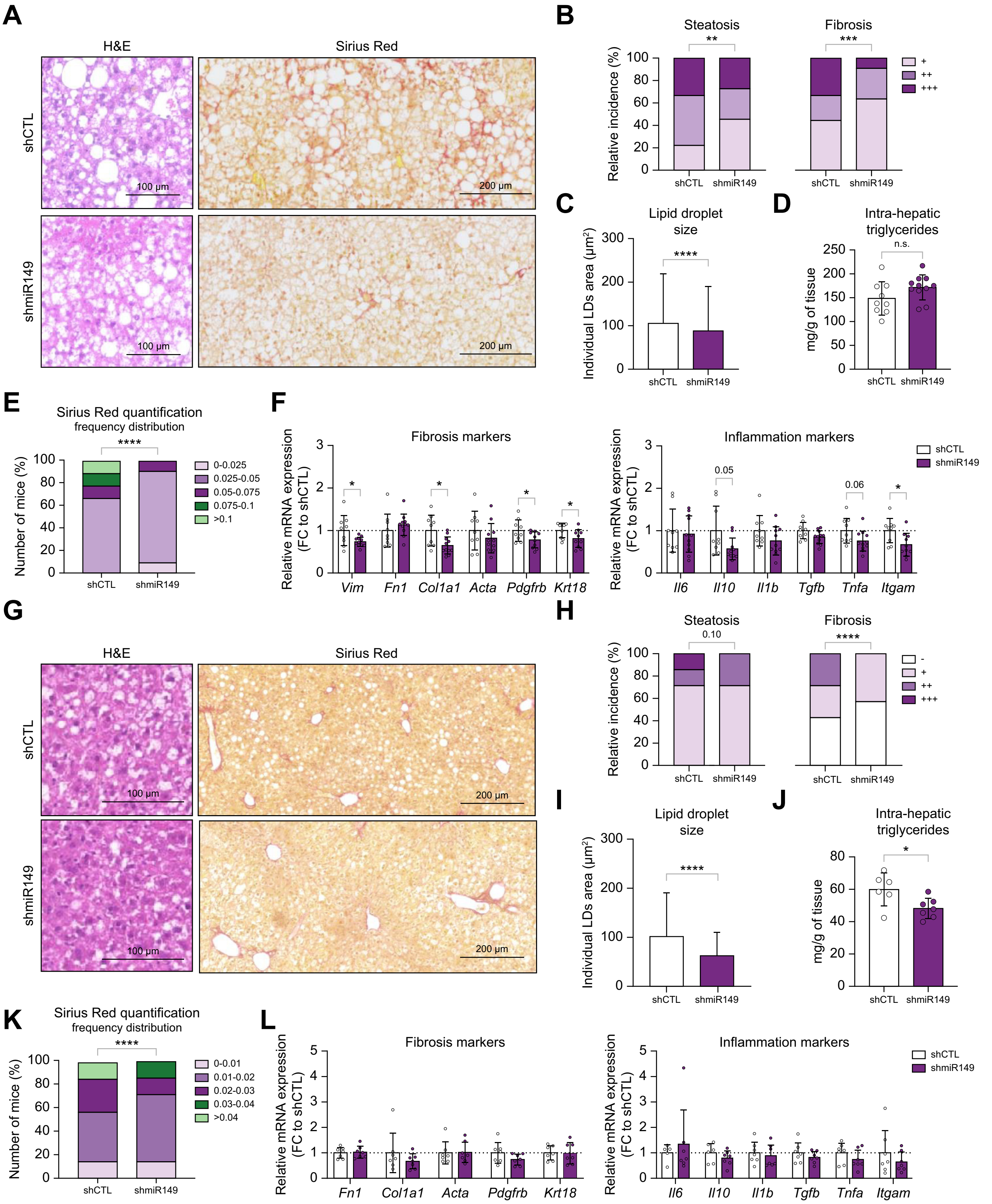


**Fig. 2. *In vivo* downregulation of miR-149-5p specifically in hepatocytes attenuates hepatic steatosis, inflammation and fibrosis induced by FPC or MCD diets.** (A, G) Representative histopathological sections: H&E (left panel) and Sirius red (right panel) staining, magnification 20x, (B, H) blind scoring of steatosis and fibrosis with corresponding (C, I) quantification of individual LD size, (D, J) intrahepatic triglyceride content, (E, K) frequency distribution of the quantification of Sirius

downregulated specifically in hepatocytes of C57BL6/J mice. Post-transduction, mice were challenged with FPC diet (short and long FPC)[25] or with MCD diet.[26]

Downregulation of miR-149-5p in hepatocytes from mice subjected to a FPC diet did not significantly affect general body parameters – body and liver weight, lean and fat mass body composition, adipose tissue size, plasma transaminases and triglycerides (TGs) levels (Fig. S4A-F). Blind scoring of steatosis and quantification of lipid droplet number and size on histological sections from liver tissues revealed a moderate attenuation of steatosis in mice following miR-149-5p downregulation, with a significant decrease in *Cd36, Cpt1a and Acox1* gene expression in the long FPC, despite no significant differences in intrahepatic TGs in either (Figs 2A-D and S5A-D). Scoring and quantification of Sirius Red staining indicated attenuation of fibrosis with downregulation of miR-149-5p (Fig. 2B,E). These histological observations were further supported by a significant decrease of molecular markers of fibrosis and inflammation (Figs 2F and S6). Activation of pro-inflammatory pathways previously linked with miR-149-5p expression[27] were also assessed by western blot (NF-κB, p38, JNK and STAT3, Fig. S7A-D) in hepatic tissues of long FPC-fed mice, as well as macrophage infiltration (Iba1+ cells, Fig. S7E). We did not observe significant differences between mice with miR-149-5p downregulated and the control group.

Despite inducing weight loss, the MCD diet promotes severe steatosis, steatohepatitis and mild fibrosis in mice but through different mechanisms than obesogenic diets.[26] miR-149-5p downregulation in mice fed a MCD diet attenuated weight loss but did not affect liver weight (Fig. S8A) nor plasma transaminase, TG or cholesterol levels (Fig. S8B-D). Histopathological analysis of H&E staining and measurement of intrahepatic TGs also indicated slightly attenuated steatosis (decreased lipid droplet size and TG content), despite no changes in the expression of genes involved in lipid uptake/oxidation (Figs 2G-J and S9A-B). Despite the mild steatosis, inflammation and fibrosis induced by MCD in our control cohort, blind assessment and quantification of Sirius Red staining also pointed to decreased fibrosis with miR-149-5p downregulation (Fig. 2H,K). A strong impact of miR-149-5p downregulation on fibrotic/inflammatory markers expression could not be confirmed (Fig. 2L).

### Hepatic miR-149-5p modulates whole-body metabolism and lipid utilization with MASLD

Along with MASLD, the FPC diet also induces insulin resistance and glucose intolerance in mice.[25] We investigated whether downregulation of hepatic miR-149 improved glucose tolerance in FPC-fed mice. Glycemia in fed state was not affected by miR-149-5p downregulation, but fasted glycemia was slightly increased after 7 weeks of diet (Fig. 3A). Insulinemia was unchanged between shCTL or shmiR149 mice (Fig. 3B), and glucose tolerance was not improved either (Fig. 3C,D). The increased glycemia in fasted states was not due to exacerbated hepatic glucose output as assessed by pyruvate tolerance tests (Fig. S10). Intrahepatic glycogen, glucose and glucose-6-phosphate content were also unchanged (Fig. 3E). However, whether hepatic miR-149-5p modulates liver-to-peripheral crosstalk mechanisms controlling glucose/lipid uptake and/or utilization remains unclear. To investigate hepatic miR-149-5p's impact in whole-organism energy metabolism, we submitted FPC-fed mice to metabolic cages. Food and water intake was unchanged between groups (Fig. S11A,B). The respiratory exchange rate was significantly decreased indicating that fatty acid oxidation was upregulated, and carbohydrate utilization was decreased in mice with downregulated hepatic miR-149-5p (Fig. 3F,G). A significant increase in the ambulatory movement was observed in mice with hepatic miR-149-5p downregulation during both the light/dark period (Fig. 3H), while a mild decrease in energy expenditure was observed during the light period (Fig. S11C), likely associated with factors unrelated to food consumption or body size/composition.

### MiRNA-149-5p overexpression triggers lipid accumulation, inflammation and fibrosis in multi-lineage 3D HLOs

To evaluate the relevance of our *in vivo* findings in the context of the human pathology, we investigated the outcome of miR-149-5p overexpression in multi-lineage 3D HLOs. HLOs were generated following optimization of a previously described protocol.[28,29] Differentiation of HLOs was validated by the loss of nanog homeobox (*NANOG*, stem cell marker), caudal type homeobox 2 (*CDX2*, mid-gut marker) and forkhead box A2 (*FOXA2*, foregut marker) expression, and increased levels of hepatocyte markers (hepatocyte nuclear factor 4 alpha (*HNF4α*), albumin (*ALB*) and α-antitrypsin (*SERPINA1*)), stellate cells marker (activated leukocyte cell adhesion molecule (*ALCAM*)) and Kupffer cell marker (*CD68,* Fig. 4A). The presence of hepatocyte-like (HNF4+/Epcam+), stellate-like (PDGFR+) and Kupffer-like cells (CD68+) was also validated by immunofluorescence (Fig. 4B). Exposure to fatty acids (400:200 μM oleate:palmitate [OAPA] for 2 days) generated steatosis in HLOs, as evidenced by BODIPY staining of neutral lipid-containing droplets in hepatocyte-like cells (HNF4+, Fig. 4C). Challenge with inflammatory cytokines induced inflammation/fibrosis in HLOs, as supported by the increase in pro-fibrotic/inflammatory gene expression (Fig. 4D).

Transfection efficiency of HLOs using small oligonucleotides was assessed using fluorescent microRNAs (Fig. 4E) and miR-149-5p overexpression was validated by quantitative reverse-transcription PCR (Fig. 4F). Quantification of lipid droplet volume in HNF4+ cells indicated that HLOs overexpressing miR-149-5p display significantly increased lipid accumulation in hepatocyte-like cells upon OAPA exposure for 7 days, but not in basal conditions (Fig. 4G). Despite the batch variability, in HLOs treated with OAPA for 7 days, a mild inflammation and fibrosis also develops (increased expression of markers, white *vs*. light pink bars, Fig. 4H), as previously reported.[28,30] In HLOs overexpressing miR-149-5p, different inflammatory/fibrotic

Red staining (positive area/total tissue area) and (F, L) relative mRNA expression of fibrosis and inflammation markers in explanted livers of FPC and MCD diet-fed mice. **p* <0.05, ***p* <0.01, ****p* <0.001, *****p* <0.0001. Unpaired t-test with Welch's correction, one-way ANOVA with Holm-Sidak's correction or Fisher's test. FPC, fructose/palmitate/cholesterol-enriched diet; LD, lipid droplet; MCD, methionine/choline-deficient diet.

markers were increased both in basal and OAPA conditions (Fig. 4H).

### Aberrant lipid accumulation due to increased glycolysis and defective mitochondrial function in Huh7 cells overexpressing miR-149-5p

To better delineate the molecular mechanisms through which miR-149-5p impacts lipid homeostasis in hepatic cells, we overexpressed miR-149-5p in Huh7 cells (Fig. 5A). As shown in Fig. 5B, C, BODIPY staining of lipid droplets indicated increased lipid accumulation in Huh7 cells overexpressing miR-149-5p in basal conditions. Western blot analyses of insulin-induced AKT-serine phosphorylation showed that miR-149-5p-induced steatosis did not impair insulin signaling (Fig. 5D).

SeaHorse analyses of Huh7 cells showed that miR-149-5p promotes an increased dependence of mitochondrial activity on fatty acids (Fig. 5E, etomoxir-treated cells), while decreasing mitochondrial oxidative phosphorylation (Fig. 5F) and mitochondrial ATP production (Fig. 5G). A quantitative increase in the mitochondrial mass was observed by both MitoTracker staining (Fig. 5H) and mitochondrial/nuclear DNA ratio (Fig. 5I) in Huh7 cells overexpressing miR-149-5p. Increased glycolytic rates in miR-149-5p-overexpressing Huh7 cells were also observed (Fig. 5J), despite no significant increase in glucose uptake (Fig. 5K) in these cells.

### MiR-149-5p alters the translation of several genes involved in metabolic and inflammatory pathways

As miRNAs modulate target gene expression by translational blockage or transcript degradation, we performed polysome fractionation and RNA sequencing of highly translated mRNAs in Huh7 cells overexpressing miR-149-5p or not, to identify miR-149-5p targets potentially contributing to MASLD. Despite no major alterations in polysome profile (Fig. 6A), we identified 587 downregulated and 532 upregulated genes in polysomal fractions of cells overexpressing miR-149-5p (Fig. 6B). Overrepresentation analyses based on KEGG pathways indicated that downregulated genes are part of key signaling pathways involved in cell metabolism, inflammatory processes and fibrosis (*e.g*. PI3K-Akt, calcium signaling, cytokine-cytokine receptor, TGF-Beta, *etc*. Fig. S12). Of note, upregulated genes were mostly regulating metabolic/inflammatory processes and detoxification mechanisms (*e.g.* retinol metabolism, complement and coagulation cascades, cell adhesion, drug metabolism, etc. Fig. S12).

The 587 potential targets found significantly downregulated were then cross-referenced with predicted/validated mRNA targets of miR-149-5p (human and mouse). Strikingly, 549 of the 587 downregulated transcripts were identified as potential targets of miR-149-5p in mice and/or humans (237 targets common between mice and humans). Gene enrichment analysis with the 237 common targets further confirmed that potential miR-149-5p target mRNAs were involved in metabolic/inflammatory/fibrotic processes (Fig. 6D). To highlight the relevance of these potential miR-149-5p targeted genes in MASLD development, we performed an extensive literature screening. Our screening indicated that 63 out of 237 were previously reported to play a significant role in different pathological processes associated with MASLD development/progression (Table S1, Fig. 6E). Among these 63 candidates, downregulation of 23 of them was previously reported to foster specific processes triggering MASLD (red and yellow genes in Fig. 6E). Of note, downregulation of 174 of the 237 predicted/validated miR-149-5p targets in mice/humans listed in Table S1 may have a significant impact on MASLD development and progression, but they have not yet been characterized in this pathological context.

## Discussion

Translational deregulation of gene expression by miRNAs is a key pathological mechanism contributing to chronic metabolic diseases such as MASLD.[6] Herein, we identify miR-149-5p, which is strongly upregulated in hepatocytes with MASLD and fosters disease progression. Our data showed that miR-149-5p upregulation affects the expression of multiple cellular factors involved in the regulation of glucose and lipid metabolism, mitochondrial function, inflammation and tissue repair. Notably, the impact of miR-149-5p expression on steatosis, inflammation and fibrosis, that we observed in 2D cell culture and/or mouse models, was also confirmed in multi-lineage 3D HLOs, a newly established experimental model highly relevant for the human pathology.[28,30] Based on publicly available GEO datasets of miRNA expression in humans with MASLD/MASH it is unclear if hepatic miR-149 is upregulated with MASLD/MASH (Fig. S13A).

The miR-149 genomic region contains 45 single nucleotide polymorphisms, among which two (rs2292832 and rs7142843, with 40% and 20% mean incidence, respectively, in the global population) were reported to have an effect on miR-149 maturation and to affect development and progression of several pathological conditions, including hepatic steatosis and hepatocellular carcinoma (HCC) (Fig. S13B-C, Table S2).[16,31] Nevertheless, the prevalence of these polymorphisms in patients with MASLD and their correlation with MASLD severity needs to be further investigated. Despite our *in vitro*/*in vivo* results indicating that miR-149 modulates fuel utilization in hepatic energetic metabolism and promotes lipid accumulation in hepatocytes, experimental models such as immortalized/cancerous hepatic cell lines or genetic/diet-induced mouse models of MASLD have limitations, which must be considered when interpreting the data and extrapolating them to the human pathology.

Hepatic cell lines are highly proliferative, have multiple mutations, genomic and metabolic alterations, and are exempt from the influence of other cells in the hepatic microenvironment.[32,33] Conversely, variability between *in vivo* models, differences between mouse and human pathophysiology (*e.g.* genetic, diets and metabolism, strain and sex, miRNA targets) must be considered when drawing conclusions about miRNA-associated pathological mechanisms.[34] Considering these issues, the development of multi-lineage stem cell-derived HLOs has gained a lot of interest for the study of MASLD-associated pathological mechanisms.[35,36] These HLOs exhibited transcriptomic profiles and metabolic activities similar to the human liver, and developed steatosis, inflammation and fibrosis upon pathological stimuli.[30] In our study, *in vivo* and *in vitro* experiments were further supported by the results obtained with miR-149-5p upregulation in HLOs, thus highlighting miR-149-5p's relevance for human

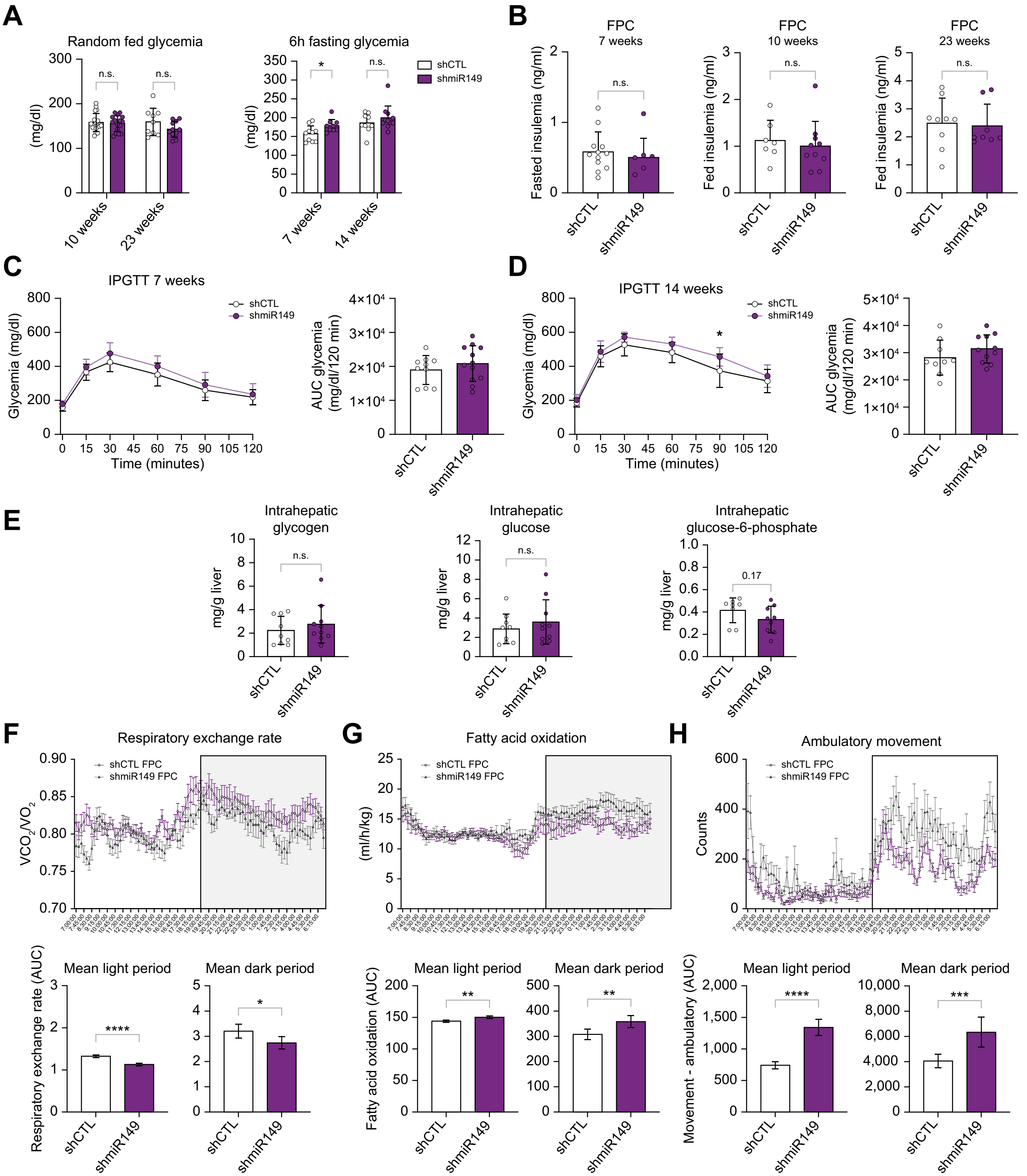


**Fig. 3. Hepatic miR-149 contributes to the regulation of fasting glycemia and modulates whole-body lipid utilization in FPC-fed mice.** (A) Glycemia measurements in fed (left) and fasting states (right). (B) Plasma insulin levels in fasting and fed states. (C, D) IPGTT (left) and calculated AUC (right) at 7 and 14 weeks of diet. (E) Intrahepatic glycogen, glucose and glucose-6-phosphate in explanted tissues. (F) Respiratory exchange rate (carbon dioxide/oxygen consumption ($VCO_2/VO_2$), (G) fatty acid oxidation rate, and (H) ambulatory movement measured over 7 days (mean, left) and corresponding AUCs (light *vs*. dark period, right). **p* <0.05, ***p* <0.01, ****p* <0.001, *****p* <0.0001. Unpaired t-test with Welch's correction. FPC, fructose/palmitate/cholesterol-enriched diet; IPGTT, intraperitoneal glucose tolerance test; AUC, Area under curve.

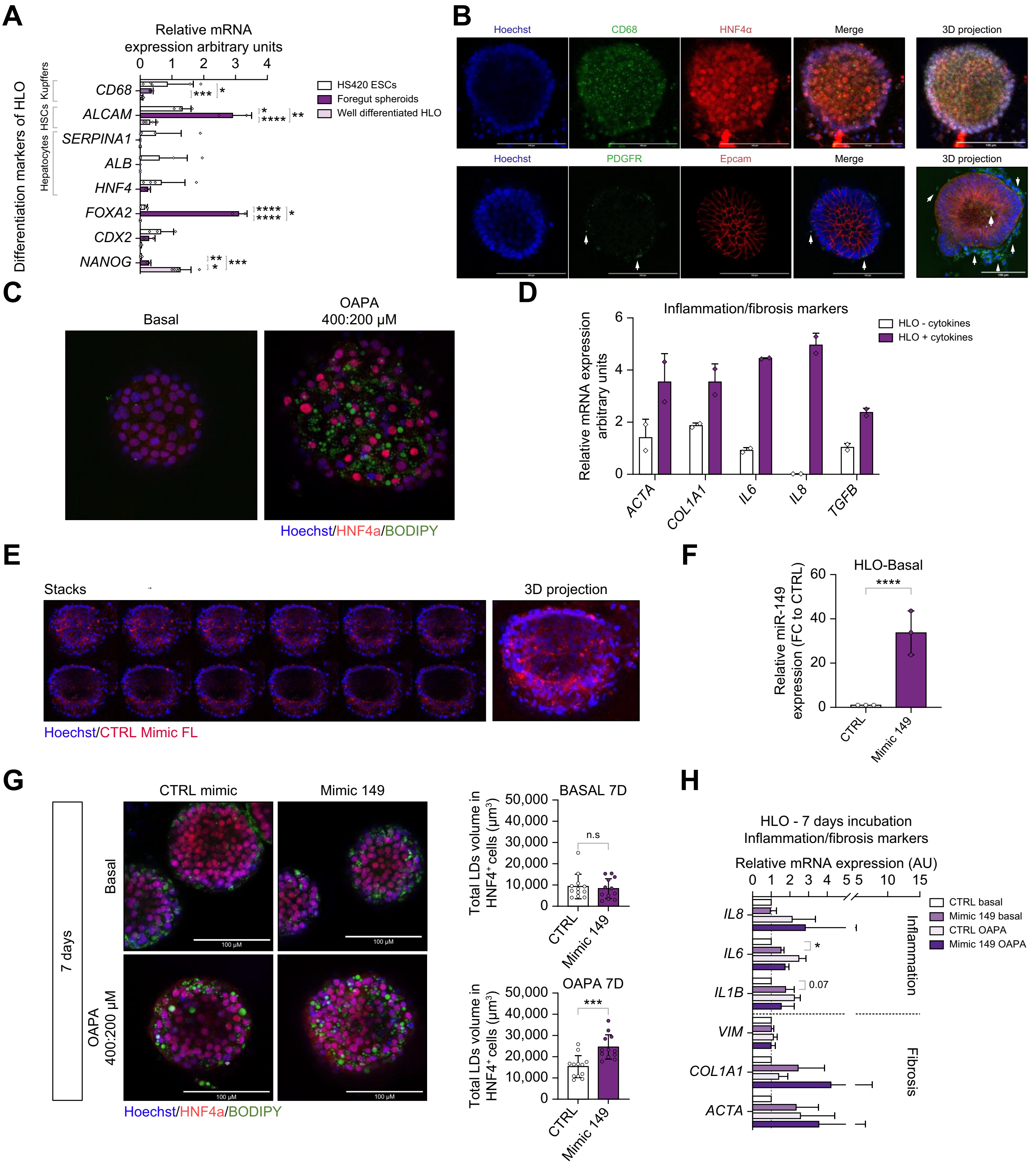


**Fig. 4. Overexpression of miR-149-5p in HLOs aggravates steatosis, inflammation and fibrosis development.** (A) Differentiation markers in HS420 ESCs, in foregut spheroids and in HLOs. Representative confocal images of (B) HNF4+/Epcam+ (red, hepatocyte-like cells), PDGFR+ (green, stellate-like cells) and CD68+ (green, Kupffer-like cells) immunofluorescence in HLOs, and of (C) BODIPY staining (green), Hoechst staining (blue) and HNF4 immunofluorescence (red) in HLOs exposed to fatty acids. (D) Expression of inflammation/fibrosis markers in HLOs exposed to cytokines. (E) Representative stacks and 3D projection of confocal images of HLOs transfected with a fluorescent scrambled oligonucleotide (CTRL Mimic FL, red). Nuclei are stained by Hoechst (blue). (F) Relative miR-149-5p expression in HLOs transfected with scrambled oligonucleotides (CTRL) or miR-149-5p oligonucleotides (Mimic 149). (G) Representative images of BODIPY staining (green), Hoechst staining (blue) and HNF4 immunofluorescence (red) with corresponding quantification of BODIPY signal per individual organoid and (H) relative expression of

pathology. Indeed, miR-149-5p overexpression in HLOs led to increased lipid accumulation in hepatocytes and upregulation of inflammatory/fibrotic markers, an inverted phenotype compared to what we observed in MASLD mouse models, in which miR-149-5p was silenced. Currently, HLOs' application in translational research investigating MASLD development is limited by the high heterogeneity between different HLO batches, incomplete maturation of cells, as well as by the difficulties in genetic manipulation of HLOs. Further optimization and standardization of HLO production and genetic engineering in the future should provide an invaluable experimental model for human liver pathophysiology, as a complement to, or even partial replacement of animal experimentation.

miR-149-5p expression appears to be regulated differently in AT and muscles in mice with diet-induced obesity, as well as being sensitive to nutritional cues and physical activity. Consistent with what we and others observed in the liver,[11,37] miR-149-5p regulated lipid metabolism in bovine adipocytes.[18,19] Of note, miR-149-3p was also upregulated in the AT of mice fed a MASLD-inducing diet which promoted IR and adipogenesis in part by targeting PRDM16.[13] In contrast, miR-149-5p was downregulated in skeletal muscle upon diet-induced obesity in mice, thus contributing to reduced mitochondrial biogenesis and IR partially due to PARP-2 upregulation.[14] Several factors can explain the tissue-specific regulation of genes, the expression of different miR-149 strands and their impact on organs or whole-body homeostasis (*e.g.* diet type/duration, mouse strains, *etc*.).[6,7,38,39] Regarding the PRDM16 and PARP-2 miR-149 targets previously identified in AT and muscles, respectively,[13,14] we did not detect changes in their expression in our translatomic analysis, again suggesting a context-dependent regulation of miR-149 targets.[38] However, given the numerous deregulated factors identified in our analyses as potential miR-149-5p targets, it is likely that the metabolic impact of miR-149-5p upregulation in hepatocytes is driven by fine modulation of multiple metabolic regulators rather than one single target.

Chen *et al.* recently reported hepatic miR-149 downregulation in diet-induced obesity in mice and that administration of miR-149 agomiRs led to an attenuation of hepatic steatosis and injury.[40] These discrepancies likely stem from differences in experimental design and methods. First, the 4-week high fat-containing diet administered in mice by Chen and colleagues[40] induced obesity, hepatic steatosis and IR but with no progression towards liver inflammation and fibrosis. In contrast, the FPC diet used in our study did not promote a significant weight gain in mice, but it led to massive steatosis and IR, as well as mild inflammation and fibrosis. The MCD diet induced significant weight loss concomitantly with steatosis, inflammation and fibrosis, but not insulin resistance. The systemic effects induced by the different diets may also have an impact on hepatic miR-149-5p expression/activity and associated metabolic disorders through inter-organ crosstalk mechanisms. While we injected hepatotropic adeno-associated virus 8 to specifically knockdown miR-149-5p in hepatocytes, Chen *et al.* injected miR-149 agomiRs systemically through the tail vein.[40] Administration of synthetic nucleotides in mice was shown to trigger off-target effects due to the lack of cell/tissue specificity,[41] as previously reported for miR-144-3p agomiRs injected in a model of diet-induced atherosclerosis[42] or for miR-24 and miR-122 agomiRs that were detected in the liver, small intestine, aorta and muscle after tail injection.[43] It is also well established that modulation of miRNA expression/activity by genetic engineering or administration of synthetic pharmacological nucleotides (*e.g.* agomiRs or antagomiRs) can yield different, or even opposite, biological outcomes in mice, as supported by previous studies investigating the role of miR-21 in cardiac diseases,[44,45] or of miR-22 in diet-induced hepatic steatosis.[46,47]

Links between miR-149 expression/activity and inflammatory diseases were previously reported,[27,48–51] but how miR-149 impacts hepatic inflammatory processes remain unclear. In patients with chronic hepatitis C virus infection, hepatic miR-149-3p was shown to be strongly upregulated,[52] while its expression decreased in a mouse model of carbon tetrachloride-induced hepatic injury.[53] miR-149 was further demonstrated to upregulate pro-inflammatory signaling mediated by STAT3, NF-kB, AMPK, MAPK and NFATc4,[27] leading to changes in immune cell activity[50] and expression of pro-inflammatory cytokines such as *Cxcl10, Il1b, Il6, Ccl2/5, Il8* and *Tnfa*.[27,51] In contrast, genetic deficiency of both miR-149 strands in mice (MIR149KO mice) promoted LPS- and acute diethylnitrosamine-induced hepatic injury, by increasing STAT3 signaling, pro-inflammatory cytokine expression and hepatocyte death.[21,49] Administration of miR-149-3p/5p agomiRs in MIR149KO mice partially alleviated LPS-induced hepatic inflammation.[49] In our studies, inhibition of miR-149-5p *in vivo* triggered a mild reduction of inflammatory markers in FPC-fed mice, while this fine-tuned regulation was likely masked by the strong effect of the MCD on hepatic and whole-organism homeostasis. However, overexpression of miR-149-5p in HLOs significantly increased the expression of specific inflammatory cytokines (*i.e. IL6, IL1B*) and our translatomic analyses of Huh7 also showed a significant negative impact of miR-149-5p overexpression on factors governing inflammatory pathways. Again, the different experimental settings and the context-dependent function of the different miR-149 strands could be responsible for these divergent data in mice. Of note, MIR149KO mice have a deletion of both miR-149 strands in all cells. It is not possible to assess the role of different miR-149 strands in specific cell types (*e.g.* hepatic stellate cells, immune cells or hepatocytes). It is also unclear whether miR-149-3p and miR-149-5p target different sets of genes, and if the different strands have opposing or synergistic effects on inflammatory processes. Additionally, potential adaptive mechanisms occurring during embryogenesis and development to circumvent loss of miR-149 further complicate the interpretation of data obtained with total knockout mice.

Finally, regarding the impact of miR-149-5p in fibrosis development, what we observed in mice, HLOs and translatomic data is consistent with previous reports indicating that

inflammation/fibrosis markers in HLOs overexpressing or not miR-149-5p and challenged with pro-steatotic/pro-inflammatory conditions (n = 3-4 per group). **p* <0.05, ***p* <0.01, ****p* <0.001, *****p* <0.0001. Unpaired t-test with Welch's correction or One-way ANOVA with Holm-Sidak's correction. ESCs, embryonic stem cells; HLOs, human liver organoids.

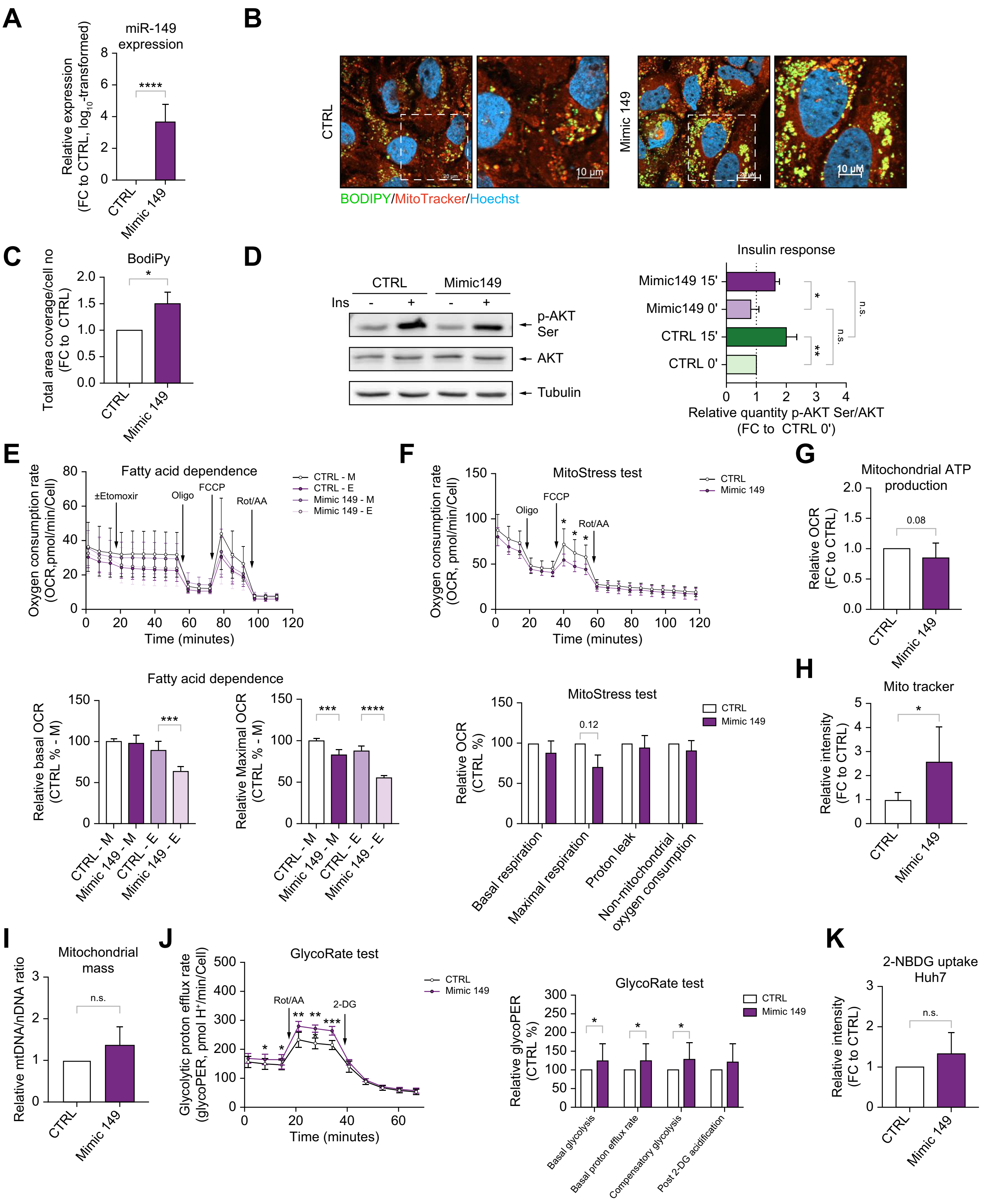

**Fig. 5. MiR-149-5p overexpression promotes intracellular lipid accumulation in Huh7 cells by promoting glucose metabolism and impairing lipid catabolism.** (A) Relative miR-149-5p expression in transfected Huh7 cells. Data was log10 transformed. (B) Representative pictures of MitoTracker (red), Hoechst (blue) and BODIPY (green) staining in transfected Huh7 cells. Measurement of (C) total area coverage of lipid droplets normalized to cell number in transfected Huh7 cells. (D) Representative western blot analysis (left panel) and respective quantification (right panel) of the ratio of phosphorylated AKT (Serine 473)/total AKT in transfected Huh7

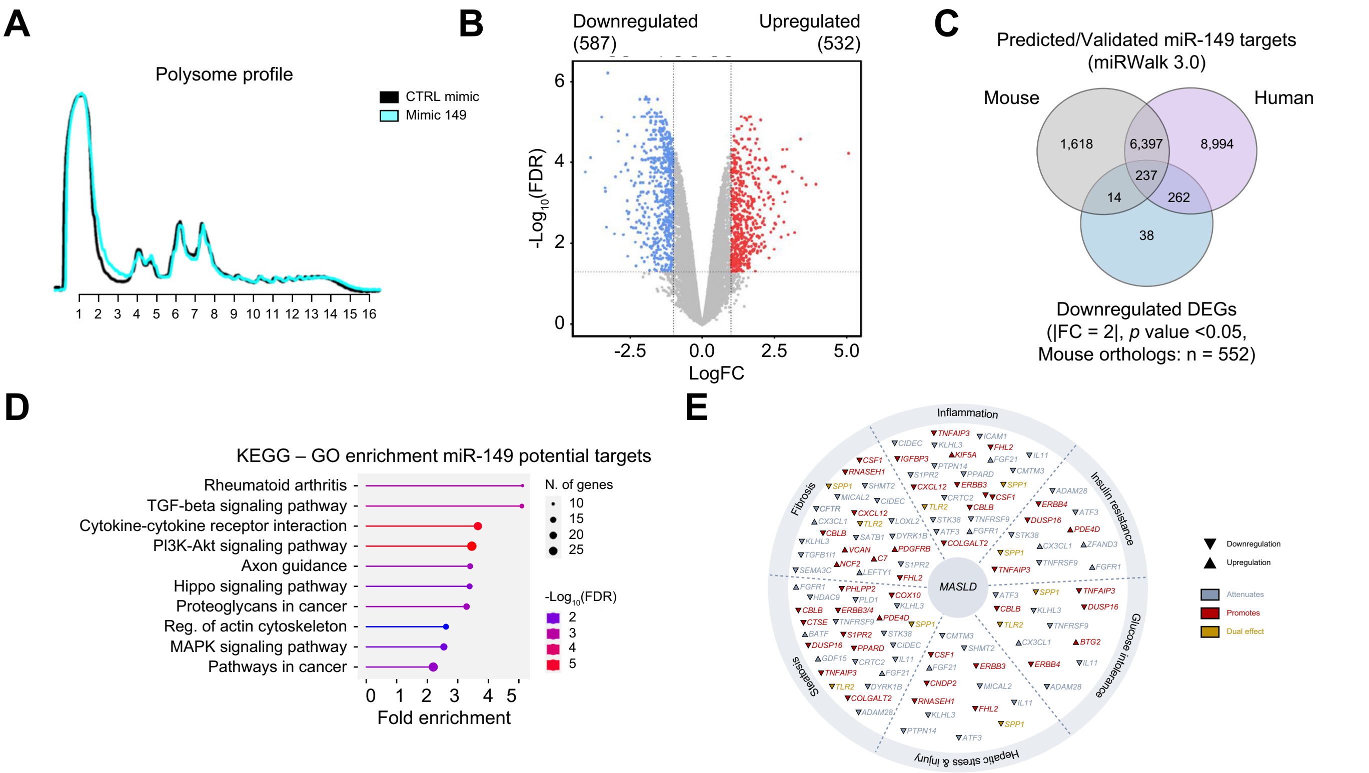


**Fig. 6. MiR-149-5p overexpression impairs a wide network of genes involved in metabolic/inflammatory/fibrotic processes.** (A) Representative polysomes profiles and (B) deregulated genes identified in RNAseq of polysomal fractions of transfected Huh7 cells (n = 3 per group). (C) Venn diagram representing identified downregulated DEGs crossed with predicted/validated targets in both human and mouse genomes. ORAs by KEGG pathways of (D) shared targets (human and mouse) and of exclusively human targets. (E) Graphical representation of shared targets with reported implications in pathological mechanisms driving MASLD development/progression. Genes in light blue or red indicate an attenuation or promotion, respectively, while genes in yellow indicate a dual effect on the indicated process. ▲ upregulated; ▼ downregulated. DEGs, differentially expressed genes; MASLD, metabolic dysfunction-associated steatotic liver disease; ORAs, over-representation analyses.

miR-149 overexpression affects TGF-β signaling,[50] and activates LX2 human cultured stellate cells *in vitro*.[53] The mild reduction in inflammation and fibrosis that we observed in FPC-fed mice following miR-149-5p downregulation could also derive from the improved steatosis. Indeed, pathological accumulation of specific lipid species (*e.g.* lipid peroxides, acylceramides, diacylglycerol, palmitate, *etc.*) can strongly promote lipotoxicity and inflammatory responses.[54] Future investigations are required to experimentally validate if miR-149-5p drives inflammation and fibrosis indirectly through alterations of the glucose/lipid metabolism and/or also by targeting important cellular factors restraining inflammation and fibrosis in other cell types.

The impact of one specific miRNA in each cell is complex and does not depend solely on its expression. There is a delicate equilibrium established between miRNAs, and/or RNA-binding proteins, competing for overlapping seed sequences on the same mRNA 3'untranslated region.[7,55] miRNA activities might also be tightly regulated independently of their expression as shown previously for miR-21 in hepatocytes.[56,57] Finally, miRNAs can also be secreted or sequestered by other molecules such as pseudogenes or other non-coding RNAs.[58–60] All these processes are highly dependent on the cellular and organ context, as well as external pathophysiological stimuli.[53,56] Based on our current knowledge of miRNA biology, it is inaccurate to attribute the pathophysiological outcomes of miRNA deregulation to the modulation of a single target gene. Since miRNAs can either block the translation of several transcripts, or trigger their degradation without necessarily affecting gene transcription, we performed translatomic analyses to identify potential miR-149-5p targets. Not surprisingly, miR-149-5p overexpression in Huh7 cells promotes the downregulation of several transcripts potentially containing miR-149-5p-specific seed sequences. Whether all the identified downregulated transcripts are directly linked with miR-149-5p activity remains to be experimentally validated, which surpasses the scope of this study. Nevertheless, more than 60 of these mRNAs were previously reported to play a significant role

cells. (E) Fatty acid dependence test and (F) mitochondrial respiration rates through measurement of OCR (profile in the upper panel and relative parameters in the lower panel), (G) mitochondrial ATP production, (H) quantification of MitoTracker staining, (I) mitochondrial/nuclear DNA ratio, (J) glycolytic rate measurement (glycoPER) - profile in the left panel and relative parameters in the right panel and (K) 2-NBDG uptake in transfected Huh7 cells (n = 3-9 per group). Data is represented as mean±SD fold change to respective control when indicated. *$p$ <0.05, **$p$ <0.01, ***$p$ <0.001, ****$p$ <0.0001. Unpaired t-test with Welch's correction or one-way ANOVA with Holm-Sidak's correction. GlycoPER, glycolytic proton efflux rate; OCR, oxygen consumption rate.

in MASLD development, leading us to conclude that miR-149-5p in hepatocytes likely influences the expression of numerous critical metabolic/inflammatory factors, collectively contributing to aggravation of MASLD, as further supported by our functional analyses.

While miR-149-5p is upregulated in MASLD, a switch in its expression occurs with hepatic carcinogenesis and miR-149-5p becomes strongly downregulated in HCC.[61–64] Considering the impact of miR-149-5p on the hepatic metabolism it is likely that expression of miR-149-5p targets contributes to carcinogenesis either by favoring metabolic switches in cancer cells (*e.g.* Warburg effect) or by promoting other cancer-related features. Here again, the drastic shift in miR-149-5p expression is likely to affect multiple mRNAs involved in these processes and future studies are required to understand how miR-149 downregulation contributes to HCC development. In this regard, the well-established target of miR-149-5p, *Fgf21*[15,17] (also validated in this study – Fig. S14 and in translatomic data) is an interesting example, which contributes to MASLD development when it is downregulated and to HCC development when it is upregulated. FGF-21 expression or analogs counteract metabolic disorders, inflammation and fibrosis in the liver,[65–67] while FGF-21 can have oncogenic activity in various cancers.[68–70]

Based on our data, and those of others,[16,37] elevated miR-149-5p levels in hepatocytes foster MASLD development by affecting whole-body energy metabolism and hepatic glucose/lipid metabolism, mitochondrial function, and the expression/activity of pro-inflammatory/pro-fibrogenic molecules. Downregulation of miR-149-5p in hepatocytes appears to be a potential therapeutic strategy for MASLD. Synthetic pharmacological miRNA inhibitors are currently being clinically evaluated to treat liver pathologies, *e.g.* miR-34 antagomiRs for HCC[71] and miR-122 antagomiRs for HCV infection.[72,73] However, as previously discussed, prudence is needed before envisaging miR-149-5p inhibition as a therapeutic approach for MASLD. In particular, it is required to better understand the respective role of each miR-149 strand in the different hepatic cells. Further research is also needed to achieve cell-specific targeting of synthetic pharmacological miRNA inhibitors or mimics in order to prevent off-target effects of these molecules.


## Affiliations

[1]*Department of Cell Physiology and Metabolism, Faculty of Medicine, University of Geneva, Geneva, Switzerland;* [2]*Department of Pathology and Immunology, Faculty of Medicine, University of Geneva, Geneva, Switzerland*


## Abbreviations

AT, adipose tissue; AAV8, adeno-associated virus serotype 8; BAT, brown adipose tissue; ESC, embryonic stem cells; eWAT, epididymal white adipose tissue; FPC, fructose/palmitate/cholesterol-enriched; GEO, gene expression omnibus; HCC, hepatocellular carcinoma; HFD, high-fat diet; HLOs, human liver organoids; IPGTT, intraperitoneal glucose tolerance test; IR, insulin resistance; LPS, lipopolysaccharide; LPTENKO, liver-specific *Pten* knockout; MASLD, metabolic dysfunction-associated steatotic liver disease; MCD, methionine/choline-deficient diet; miRNA, microRNA; mRNA, messenger RNA; mWAT, mesenteric white adipose tissue; NAFLD, non-alcoholic fatty liver disease; OAPA, oleic:palmitic acid; shRNA, short-hairpin RNA; T2D, type 2 diabetes; TG, triglyceride.


## Financial support

This study was supported by the Ernest Boninchi Foundation, the Bo and Kerstin Hjelt Diabetes Foundation and by the Swiss National Science Foundation (grant numbers 310030-172862 and 320030-200530).


## Conflict of interest

The authors declare no conflict of interest.

Please refer to the accompanying ICMJE disclosure forms for further details.

## Authors' contributions

Study concept and design – MCS, MGj, FB, MFot; Acquisition of data – MCS, MGj, ED, CM, MFou, FB; analysis and interpretation of data – MCS, MGj, ED, CM, MFot; drafting of the manuscript – MCS, MGj, MFot; critical revision of the manuscript – MCS, MGj, ED, FB, MFot; statistical analysis – MCS; funding acquisition – MGj and MFot; Study supervision – MGj and MFot.

## Data availability statement

Data presented in this manuscript will be publicly available on https://doi.org/10.26037/yareta:lcl2chfvereufnbarebilj5yyq.

## Declaration of Generative AI and AI-assisted technologies in the writing process

During the preparation of this work, the authors used ChatGPT in order to improve language and readability. After using this tool/service, the authors reviewed and edited the content as needed and take full responsibility for the content of the publication.


## Acknowledgements

The authors thank Yves Cambet for the help with the Seahorse experiments, Dr. Mylène Docquier and her team as well as Olesya Panasenko for the assistance in the translatomic experiment and Nicolas Liaudet for the image analysis assistance. The authors also thank Prof. Pierre Maechler for the support provided during the completion of this study, following Prof. Michelangelo Foti's death. The authors deeply acknowledge the invaluable guidance and support provided by Prof. Michelangelo Foti, whose unwavering commitment to this research, substantial scientific contributions, and instrumental role in securing funding have left an indelible mark on this work. Sadly, Prof. Michelangelo Foti is no longer with us, but his legacy endures in the completion of this study.


## Supplementary data

Supplementary data to this article can be found online at https://doi.org/10.1016/j.jhepr.2024.101126.

## References

*Author names in bold designate shared co-first authorship*


[1] Younossi ZM, Golabi P, Paik JM, et al. The global epidemiology of nonalcoholic fatty liver disease (NAFLD) and nonalcoholic steatohepatitis (NASH): a systematic review. Hepatology 2023;77:1335–1347.

[2] Saltiel AR, Kahn CR. Insulin signalling and the regulation of glucose and lipid metabolism. Nature 2001;414:799–806.

[3] Gastaldelli A, Cusi K. From NASH to diabetes and from diabetes to NASH: mechanisms and treatment options. JHEP Rep 2019;1:312–328.

[4] Pierantonelli I, Svegliati-Baroni G. Nonalcoholic fatty liver disease: basic pathogenetic mechanisms in the progression from NAFLD to NASH. Transplantation 2019;103:e1–e13.

[5] Chen XM. MicroRNA signatures in liver diseases. World J Gastroenterol 2009;15:1665–1672.

[6] Gjorgjieva M, Sobolewski C, Dolicka D, et al. miRNAs and NAFLD: from pathophysiology to therapy. Gut 2019;68:2065.

[7] Correia de Sousa M, Gjorgjieva M, Dolicka D, et al. Deciphering miRNAs' action through miRNA editing. Int J Mol Sci 2019;20.

[8] Bhaskaran M, Mohan M. MicroRNAs: history, biogenesis, and their evolving role in animal development and disease. Vet Pathol 2014;51:759–774.

[9] Fernandez-Hernando C, Ramirez CM, Goedeke L, et al. MicroRNAs in metabolic disease. Arterioscler Thromb Vasc Biol 2013;33:178–185.

[10] Deiuliis JA. MicroRNAs as regulators of metabolic disease: pathophysiologic significance and emerging role as biomarkers and therapeutics. Int J Obes (Lond) 2016;40:88–101.

[11] **Zhao X**, **Chen Z**, Zhou Z, et al. High-throughput sequencing of small RNAs and analysis of differentially expressed microRNAs associated with high-fat diet-induced hepatic insulin resistance in mice. Genes Nutr 2019;14:6.
[12] Ding H, Zheng S, Garcia-Ruiz D, et al. Fasting induces a subcutaneous-to-visceral fat switch mediated by microRNA-149-3p and suppression of PRDM16. Nat Commun 2016;7:11533.
[13] Zheng S, Guo S, Sun G, et al. Gain of metabolic benefit with ablation of miR-149-3p from subcutaneous adipose tissue in diet-induced obese mice. Mol Ther Nucleic Acids 2019;18:194–203.
[14] Mohamed JS, Hajira A, Pardo PS, et al. MicroRNA-149 inhibits PARP-2 and promotes mitochondrial biogenesis via SIRT-1/PGC-1alpha network in skeletal muscle. Diabetes 2014;63:1546–1559.
[15] Xiao J, Lv D, Zhao Y, et al. miR-149 controls non-alcoholic fatty liver by targeting FGF-21. J Cel Mol Med 2016;20:1603–1608.
[16] An X, Yang Z, An Z. MiR-149 compromises the reactions of liver cells to fatty acid via its polymorphism and increases non-alcoholic fatty liver disease (NAFLD) risk by targeting methylene tetrahydrofolate reductase (MTHFR). Med Sci Monitor 2017;23:2299–2307.
[17] Chen S, Chen D, Yang H, et al. Uric acid induced hepatocytes lipid accumulation through regulation of miR-149-5p/FGF21 axis. BMC Gastroenterol 2020;20:39.
[18] Khan R, Raza SHA, Junjvlieke Z, et al. Bta-miR-149-5p inhibits proliferation and differentiation of bovine adipocytes through targeting CRTCs at both transcriptional and posttranscriptional levels. J Cel Physiol 2020;235:5796–5810.
[19] Guo H, Khan R, Abbas Raza SH, et al. RNA-seq reveals function of bta-miR-149-5p in the regulation of bovine adipocyte differentiation. Animals (Basel) 2021;11.
[20] Zhang Q, Su J, Wang Z, et al. MicroRNA-149* suppresses hepatic inflammatory response through antagonizing STAT3 signaling pathway. Oncotarget 2017;8:65397–65406.
[21] Feng Q, Zhang H, Nie X, et al. miR-149* suppresses liver cancer progression by down-regulating tumor necrosis factor receptor 1-associated death domain protein expression. Am J Pathol 2020;190:469–483.
[22] Peyrou M, Bourgoin L, Poher A-L, et al. Hepatic PTEN deficiency improves muscle insulin sensitivity and decreases adiposity in mice. J Hepatol 2015;62:421–429.
[23] D'Souza RF, Markworth JF, Aasen KMM, et al. Acute resistance exercise modulates microRNA expression profiles: combined tissue and circulatory targeted analyses. PloS one 2017;12.
[24] Linecker M, Frick L, Kron P, et al. Exercise improves outcomes of surgery on fatty liver in mice: a novel effect mediated by the AMPK pathway. Ann Surg 2020;271:347–355.
[25] Hansen HH, Feigh M, Veidal SS, et al. Mouse models of nonalcoholic steatohepatitis in preclinical drug development. Drug Discov Today 2017;22:1707–1718.
[26] Machado MV, Michelotti GA, Xie G, et al. Mouse models of diet-induced nonalcoholic steatohepatitis reproduce the heterogeneity of the human disease. PLoS One 2015;10:e0127991.
[27] Feng Q, Li Y, Zhang H, et al. Deficiency of miRNA-149-3p shaped gut microbiota and enhanced dextran sulfate sodium-induced colitis. Mol Ther Nucleic Acids 2022;30:208–225.
[28] Ouchi R, Togo S, Kimura M, et al. Modeling steatohepatitis in humans with pluripotent stem cell-derived organoids. Cell Metab 2019;30:374–384 e376.
[29] Thompson WL, Takebe T. Generation of multi-cellular human liver organoids from pluripotent stem cells. Methods Cel Biol 2020;159:47–68.
[30] Kimura M, Iguchi T, Iwasawa K, et al. En masse organoid phenotyping informs metabolic-associated genetic susceptibility to NASH. Cell 2022;185:4216–4232 e4216.
[31] Ding SL, Wang JX, Jiao JQ, et al. A pre-microRNA-149 (miR-149) genetic variation affects miR-149 maturation and its ability to regulate the Puma protein in apoptosis. J Biol Chem 2013;288:26865–26877.
[32] Green CJ, Parry SA, Gunn PJ, et al. Studying non-alcoholic fatty liver disease: the ins and outs of in vivo, ex vivo and in vitro human models. Horm Mol Biol Clin Investig 2018;41.
[33] Ramos MJ, Bandiera L, Menolascina F, et al. In vitro models for non-alcoholic fatty liver disease: emerging platforms and their applications. iScience 2022;25:103549.
[34] Takahashi Y, Soejima Y, Fukusato T. Animal models of nonalcoholic fatty liver disease/nonalcoholic steatohepatitis. World J Gastroenterol 2012;18:2300–2308.
[35] Harrison SP, Baumgarten SF, Verma R, et al. Liver organoids: recent developments, limitations and potential. Front Med (Lausanne) 2021;8:574047.
[36] Ramli MNB, Lim YS, Koe CT, et al. Human pluripotent stem cell-derived organoids as models of liver disease. Gastroenterology 2020;159:1471–1486 e1412.
[37] Lopez-Pastor AR, Infante-Menendez J, Gonzalez-Illanes T, et al. Concerted regulation of non-alcoholic fatty liver disease progression by microRNAs in apolipoprotein E-deficient mice. Dis Model Mech 2021;14.
[38] Erhard F, Haas J, Lieber D, et al. Widespread context dependency of microRNA-mediated regulation. Genome Res 2014;24.
[39] Olive V, Minella AC, He L. Outside the coding genome, mammalian microRNAs confer structural and functional complexity. Sci Signal 2015;8:re2.
[40] Chen Z, Liu Y, Yang L, et al. MiR-149 attenuates endoplasmic reticulum stress-induced inflammation and apoptosis in nonalcoholic fatty liver disease by negatively targeting ATF6 pathway. Immunol Lett 2020;222:40–48.
[41] Lee SWL, Paoletti C, Campisi M, et al. MicroRNA delivery through nanoparticles. J Control Release 2019;313:80–95.
[42] Hu YW, Hu YR, Zhao JY, et al. An agomir of miR-144-3p accelerates plaque formation through impairing reverse cholesterol transport and promoting pro-inflammatory cytokine production. PLoS One 2014;9:e94997.
[43] Sun Y, Wang H, Li Y, et al. miR-24 and miR-122 negatively regulate the transforming growth factor-beta/smad signaling pathway in skeletal muscle fibrosis. Mol Ther Nucleic Acids 2018;11:528–537.
[44] Dong S, Ma W, Hao B, et al. microRNA-21 promotes cardiac fibrosis and development of heart failure with preserved left ventricular ejection fraction by up-regulating Bcl-2. Int J Clin Exp Pathol 2014;7:565–574.
[45] Patrick DM, Montgomery RL, Qi X, et al. Stress-dependent cardiac remodeling occurs in the absence of microRNA-21 in mice. J Clin Invest 2010;120:3912–3916.
[46] Hu Y, Liu HX, Jena PK, et al. miR-22 inhibition reduces hepatic steatosis via FGF21 and FGFR1 induction. JHEP Rep 2020;2:100093.
[47] Gjorgjieva M, Sobolewski C, Ay AS, et al. Genetic ablation of MiR-22 fosters diet-induced obesity and NAFLD development. J Pers Med 2020;10.
[48] Ma L, Wang L, Chang L, et al. A role of microRNA-149 in the prefrontal cortex for prophylactic actions of (R)-ketamine in inflammation model. Neuropharmacology 2022;219:109250.
[49] Zhang QQ, Su J, Wang ZW, et al. MicroRNA-149*suppresses hepatic inflammatory response through antagonizing STAT3 signaling pathway. Oncotarget 2017;8:65397–65406.
[50] Cao Y, Wang Z, Yan Y, et al. Enterotoxigenic bacteroidesfragilis promotes intestinal inflammation and malignancy by inhibiting exosome-packaged miR-149-3p. Gastroenterology 2021;161:1552–1566 e1512.
[51] Li Q, Li S, Xu C, et al. microRNA-149-5p mediates the PM(2.5)-induced inflammatory response by targeting TAB2 via MAPK and NF-kappaB signaling pathways in vivo and in vitro. Cell Biol Toxicol 2021;39:703–717.
[52] El-Guendy NM, Helwa R, El-Halawany MS, et al. The liver MicroRNA expression profiles associated with chronic hepatitis C virus (HCV) genotype-4 infection: a preliminary study. Hepat Mon 2016;16:e33881.
[53] Zhu S, Chen X, Wang JN, et al. Circular RNA circUbe2k promotes hepatic fibrosis via sponging miR-149-5p/TGF-beta2 axis. FASEB J 2021;35:e21622.
[54] Geltinger F, Schartel L, Wiederstein M, et al. Friend or foe: lipid droplets as organelles for protein and lipid storage in cellular stress response, aging and disease. Molecules 2020;25.
[55] Dolicka D, Sobolewski C, Correia de Sousa M, et al. mRNA post-transcriptional regulation by AU-rich element-binding proteins in liver inflammation and cancer. Int J Mol Sci 2020;21.
[56] Calo N, Ramadori P, Sobolewski C, et al. Stress-activated miR-21/miR-21* in hepatocytes promotes lipid and glucose metabolic disorders associated with high-fat diet consumption. Gut 2016;65:1871–1881.
[57] Androsavich JR, Chau BN, Bhat B, et al. Disease-linked microRNA-21 exhibits drastically reduced mRNA binding and silencing activity in healthy mouse liver. RNA 2012;18:1510–1526.
[58] O'Brien J, Hayder H, Zayed Y, et al. Overview of MicroRNA biogenesis, mechanisms of actions, and circulation. Front Endocrinol 2018;9.
[59] Mori MA, Ludwig RG, Garcia-Martin R, et al. Extracellular miRNAs: from biomarkers to mediators of physiology and disease. Cel Metab 2019;30:656–673.
[60] Ren FJ, Yao Y, Cai XY, et al. MiR-149-5p: an important miRNA regulated by competing endogenous RNAs in diverse human cancers. Front Oncol 2021;11:743077.
[61] Nagy A, Lanczky A, Menyhart O, et al. Author Correction: validation of miRNA prognostic power in hepatocellular carcinoma using expression data of independent datasets. Sci Rep 2018;8:11515.
[62] Wu J, Lv S, An J, et al. Pre-miR-149 rs71428439 polymorphism is associated with increased cancer risk and AKT1/cyclinD1 signaling in hepatocellular carcinoma. Int J Clin Exp Med 2015;8:13628–13633.

[63] Wang R, Zhang J, Ma Y, et al. Association study of miR-149 rs2292832 and miR-608 rs4919510 and the risk of hepatocellular carcinoma in a large-scale population. Mol Med Rep 2014;10:2736–2744.

[64] Jia H, Yu H, Liu Q. Single nucleotide polymorphisms of MIR-149 gene rs2292832 contributes to the risk of hepatocellular carcinoma, but not overall cancer: a meta-analysis. Minerva Med 2016;107:259–269.

[65] Lee JH, Kang YE, Chang JY, et al. An engineered FGF21 variant, LY2405319, can prevent non-alcoholic steatohepatitis by enhancing hepatic mitochondrial function. Am J Transl Res 2016;8:4750–4763.

[66] Liang Q, Zhong L, Zhang J, et al. FGF21 maintains glucose homeostasis by mediating the cross talk between liver and brain during prolonged fasting. Diabetes 2014;63:4064–4075.

[67] Markan KR, Naber MC, Ameka MK, et al. Circulating FGF21 is liver derived and enhances glucose uptake during refeeding and overfeeding. Diabetes 2014;63:4057–4063.

[68] Kang YE, Kim JT, Lim MA, et al. Association between circulating fibroblast growth factor 21 and aggressiveness in thyroid cancer. Cancers (Basel) 2019;11.

[69] Yu X, Li Y, Jiang G, et al. FGF21 promotes non-small cell lung cancer progression by SIRT1/PI3K/AKT signaling. Life Sci 2021;269:118875.

[70] Florea A, Harris RB, Klimentidis YC, et al. Circulating fibroblast growth factor-21 and risk of metachronous colorectal adenoma. J Gastrointest Cancer 2021;52:940–946.

[71] Beg MS, Brenner AJ, Sachdev J, et al. Phase I study of MRX34, a liposomal miR-34a mimic, administered twice weekly in patients with advanced solid tumors. Invest New Drugs 2017;35:180–188.

[72] Lanford RE, Hildebrandt-Eriksen ES, Petri A, et al. Therapeutic silencing of microRNA-122 in primates with chronic hepatitis C virus infection. Science 2010;327:198–201.

[73] van der Ree MH, de Vree JM, Stelma F, et al. Safety, tolerability, and antiviral effect of RG-101 in patients with chronic hepatitis C: a phase 1B, double-blind, randomised controlled trial. Lancet 2017;389:709–717.


Keywords: microRNAs; glucose metabolism; lipid metabolism; metabolic disruption; steatohepatitis; human liver organoids; MCD; FPC; miR-149-5p.

*Received 6 March 2024; received in revised form 23 May 2024; accepted 27 May 2024; Available online 4 June 2024*

Journal of Hepatology, Volume *6*

## Supplemental information

# Hepatic miR-149-5p upregulation fosters steatosis, inflammation and fibrosis development in mice and in human liver organoids

**Marta Correia de Sousa, Etienne Delangre, Flavien Berthou, Sanae El Harane, Christine Maeder, Margot Fournier, Karl-Heinz Krause, Monika Gjorgjieva, and Michelangelo Foti**

**Supplementary material to:**

# Hepatic miR-149-Sp upregulation fosters steatosis, inflammation and fibrosis development in mice and in human liver organoids

Marta Correia de Sousa, Etienne Delangre, Flavien Berthou, Sanae El Harane, Christine Maeder, Margot Fournier, Karl-Heinz Krause, Monika Gjorgjieva, Michelangelo Foti

## Table of contents

Supplementary methods ........ 2

Supplementary figures ........ 19

Supplementary tables ........ 33

Supplementary references ........ 41

**Supplementary methods**

## *1.1. In vivo*

### *1.1.1. Animal housing*

Mice were adapted to the animal facility of University of Geneva for three weeks and kept in ventilated cages with 2 to 5 animals per cage. During the period of adaptation, mice had access to standard chow diet (SAFE-150 diet, SAFE, Augy, France) and water ad libitum. Animals were maintained in cages with appropriate enrichment (disposable house and nesting material) and with a 12h light/dark cycle at 23°C.

### *1.1.2. Adeno-associated virus injection*

Animals were randomly allocated to the different experimental groups. Mice were injected retro-orbitally with adeno-associated virus serotype 8 (AAV8) packed with either scrambled shRNA ($2x10^{11}$ GC/mouse of AAV8-U6-scrambled-shRNA-GFP diluted in 0.9% NaCl, group shCTL, VectorBiolabs, USA) or with shRNA specific for microRNA miR-149 ($2x10^{11}$ GC/mouse of AAV8-U6-shRNA-miR-149-5p-GFP in 0.9% NaCl, group shmiR-149 VectorBiolabs, USA).

### *1.1.3. Diets and experimental procedures*

*Mice fed with a high sugar/high fat diet (HFD)*

Two month old C57BL/6J mice (Charles Rivers Laboratory) were submitted to four different isocaloric high sugar/high-fat diet (HFD: 45% kcal from fat, 17% kcal from sucrose) or a matched Control Diet (CD: 10% kcal from fat, 17% kcal from sucrose) for 16 weeks (n=5 per group). The different HFD were a Western Diet (WD: made with lard), an omega-3 enriched HFD (O-3D - similar to the WD but with 25% of the total fat mass replaced by omega-3 fish fatty acids) and a trans-hydrogenated fatty acid enriched HFD (THD - similar to the WD but with 23%-26% of the total fat mass replaced by trans-hydrogenated monounsaturated fatty acids). Detailed information on the different HFD diets is described on Table S1.

*Mice fed with a methionine/choline-deficient diet (MCD)*

Ten weeks old mice injected with AAV8-shCTL or AAV8-shmiR-149 (n=7 per group) were allowed to recover for 10 days and then were fed with a methionine/choline deficient diet (MCD; E15653-94, ssniff, Germany) for 19 days. During this feeding

period, mice were weighted every two days. At the end of the experiment, mice were decapitated following isoflurane anesthesia and liver and blood samples collected for further analyses. Detailed information on the MCD diets is described on Table S1.

*Mice fed with a fructose/palmitate/cholesterol/trans-fat-enriched diet (FPC diet)*

Ten weeks old mice injected with AAV8-shCTL or AAV8-shmiR-149 were allowed to recover for 10 days and then were fed with a Fructose/Palmitate/Cholesterol/Trans-Fat-enriched diet (FPC, TD.19142, Envigo, USA) for 10 weeks (short FPC, n=8-12 per group) or 24 weeks (Long FPC, n=10-12 per group). For the 24 weeks protocol, mice received a second injection of adenoviruses ($1x10^{11}$ GC/mouse) at 8 weeks of diet in order to ensure hepatic knockdown of miR-149. The FPC diet was previously reported to induce liver steatosis after 8-10 weeks and liver steatosis, fibrosis and inflammation after 16-24 weeks (1). During the feeding periods, mice were weighted each week and glycemia was measured at 9 a.m. using a Glucometer (AccuCheck - Roche) on blood collected from the tail vein after 10 (n=16-18 per group) and 23 (n=9-11 per group) weeks of diet. Glucose and pyruvate tolerance tests were performed on mice at 7/14 weeks of diet and 8/22 weeks of diet, respectively. The same blood samples (fasted: n=8-12 per group at 7 weeks; fed: n=7-10 per group at 10 weeks, n=8-9 per group at 22 weeks) were used to measure insulinemia by ELISA (Mercodia Ultrasensitive Mouse Insulin ELISA – 10-1249-01). Detailed information on the FPC diets is described on Table S1.

### *1.1.4. Glucose and Pyruvate Tolerance Tests*

For the glucose tolerance test, mice were fasted 6 hours prior the intraperitoneal injection of 2 g/kg of glucose (n=10-12 per group at 7 weeks, n=9-11 per group at 14 weeks). For the pyruvate tolerance test, mice were fasted 18 hours prior the intraperitoneal injection of 2 g/kg of pyruvate (Sigma)(n=8-11 per group at 8 weeks, n=9-11 per group at 22 weeks). Blood glucose levels were measured at 0, 15, 30, 60, 90 and 120 minutes post-injection of glucose/pyruvate using a Glucometer (AccuCheck – Roche) in blood samples collected from the tail vein.

### *1.1.5. Metabolic cages and EchoMRI*

Metabolic phenotyping of FPC fed mice (23 weeks, n=6-7 per group) was performed for 7 days using metabolic cages (LabMaster) after 2 days of adaptation prior to recording calorimetric parameters ($O_2$ consumption, $CO_2$ production, respiratory exchange ratio

and energy expenditure), food and water intake and locomotor activity. During this procedure, mice were isolated in metabolic cages. Body composition was measured using a positron emission tomographic whole-body composition analyzer (EchoMRI-700, Houston, Texas, USA).

*1.1.6. Plasma analyses*

Blood recovered during sacrifice following decapitation was centrifuged at 5000 rpm (Centrifuge) for 10 minutes. Plasma was collected and glucose, aspartate/alanin-aminotransferases (ASAT/ALAT), cholesterol and triglycerides levels were analyzed using Cobas 8000 system (Roche, Switzerland).

### *1.2. In vitro*

*1.2.1. Isolation of primary hepatocyte*

Primary mouse hepatocytes were isolated from LPTENKO mice and control littermates as previously described (2, 3).

*1.2.2. Cell lines*

Huh7 cells were purchased from Sekisui Genotech (JCRB0403, Japan) and cultured in DMEM (1g/L glucose, Gibco) supplemented with 1% penicillin-streptomycin (PS, Gibco) and 10% fetal bovine serum (FBS, Gibco).

*1.2.3. Human Liver Organoids*

Human Liver Organoids (HLOs) were generated from human embryonic stem cells (ESC) (HS420, BAG-hES-IMP-0046, Karolinska Institute, Stockholm, Sweden) and cultured as previously described by Ouchi et al., 2019 with slight modifications (4). Briefly, HS420 were cultured in 35mm-dishes coated with 0.5µg/cm$^2$ laminin iMatrix-521 silk (A29248, Gibco) at 37°C in 5% $CO_2$. Upon 70%-80% confluency, cells were detached with Accutase (Gibco), washed with Wash medium (DMEM/F12 supplemented with 1% PS, Gibco) and centrifuged for 3 minutes at 300 x g at room temperature. The cell pellet was resuspended in mTeSR1 supplemented with 1% PS (Gibco) and 10 µM of Rock inhibitor (Y27632, Tocris) and 500'000 cells/mL were seeded in laminin coated 35mm-dishes following Trypan Blue Exclusion Test. Following 24 hours, the Rock inhibitor was removed from the medium. To induce definitive endoderm differentiation, when cells reached 85-90% confluence medium was changed to RPMI 1640 medium (61870010, Gibco) supplemented with 1% PS (Gibco), 50 ng/mL bone morphogenetic protein 4 (BMP4, 314-BP-010, R&D Biotechne) and 100

ng/mL Activin A (338-AC-050, R&D Biotechne). At day 2, RPMI 1640 was supplemented with 100 ng/mL Activin A (338-AC-050, R&D Biotechne) and 0.2% KSR (A3181502, Gibco) and at day 3 with 100 ng/mL Activin A (338-AC-050, R&D Biotechne) and 2% KSR (A3181502, Gibco). From day 4 to day 6, the medium was changed to Advanced DMEM/F12 (12634010, Gibco) supplemented with 0.1 mM Hepes, GlutaMax (35050038, Gibco), 1x B27 (17504044, Gibco), 1x N2 (17502048, Gibco), 1x gentamycin/amphotericin (R01510, Gibco), 500 ng/mL fibroblast growth factor 4 (FGF4, PeproTech) and 3μM CHIR99021 (130-106-539, Miltenyi), and replaced daily. At the end of 6$^{th}$ day, spheroids and associated cells were gently pipetted to release them from the dish. Subsequently, they were washed with washing medium and centrifuged for 3 minutes at room temperature and 300 x g to prepare them for embedding in 100% Matrigel. Matrigel drops containing spheroids and cells were plated in 35-mm dishes and Advanced DMEM/F12 with B27, N2 medium was further supplemented with 2μM retinoic acid (RA, Sigma). Following 4 days of RA treatment, medium was changed to Hepatocyte Culture Medium (HCM, Lonza) supplemented with 10 ng/mL hepatocyte growth factor (HGF, PeproTech), 100 nM Dexamethasone (Sigma) and 20 ng/mL Oncostatin M (300-10, Peprotech) and replaced every 3 days. At day 17, Matrigel drops were disrupted with gentle pipetting to release the HLOs and they were kept in suspension in complete HCM medium supplemented with 10% Matrigel (Gibco) until the end of the experiment (day 21, day 24 or day 28). Differentiation state of each preparation was assessed through quantitative expression (RT-qPCR) of cell markers: stem-cells (*NANOG*), mid-gut (*CDX2*), foregut (*FOXA2*), hepatocytes (*HNF4, ALB, SERPINA1*), stellate cell (*ALCAM*) and Kupffer cells (*CD68*). Ethical authorization to use ESC HS420 cells was provided by the Geneva Health Head Office (authorization number R-FP-S-2-0028) and performed following the Swiss guidelines on Research involving embryonic stem cells.

*1.2.4. Synthetic Oligonucleotide transfections*

Huh7 cells were transfected 24 hours after seeding using Interferin (Polyplus transfection, Illkirch, France) and Optimem (Gibco), following manufacturer’s instructions. HLOs were transfected using Lipofectamine (ThermoFisher Scientific) and Optimem (Gibco), following manufacturer’s instructions. HLOs were kept in DMEM (1g/L glucose, Gibco) supplemented with 1% PS for 24 hours and then the medium was changed to HCM supplemented as described in 2.2.2. until the end of the experiment. Huh7 cells and HLOs were transfected with miRIDIAN microRNA miR- 149-5p mimic

(Mimic 149, Horizon Discovery, UK) or miRIDIAN microRNA Mimic Negative Control #1 coupled to a fluorophore or not (CTRL Mimic/ CTRL Mimic FL, Horizon Discovery, UK) at concentrations of 10 nM and 25 nM for Huh7 cells and HLOs respectively.

### *1.2.5. SeaHorse analyses*

Twenty-four hours after transfection of miRIDIAN microRNAs, Huh7 cells were re-seeded in a 96-well Seahorse Agilent Plate at 25'000 cells per well. Twenty-four hours post-reseeding, different Seahorse XF metabolic assays were performed in a Seahorse XFe96 Analyzer according to the manufacturer's recommendations – MitoStress, GlycoRate and Substrate Oxidation Test. At the end of each assay, cells were fixed with 4% paraformaldehyde for 15 minutes at room temperature, stained with Hoechst (1 µg/mL, 33342, ThermoFisher Scientific) for 10 minutes and scanned on Cytation 5. Following image acquisition, cell number was counted using the Gen5 software (BioTek) and used to normalize Seahorse assays results using Wave 2.4.0. software (Agilent Technologies).

#### *1.2.5.1. MitoStress Test*

Seahorse XF MitoStress Test (MitoStress 103015-100 kit) was performed as described by the manufacturer, using the following drug concentrations: Oligomycin (Oligo) – 1.5 µM, FCCP – 2 µM and Rotenone/Antimycin A (Rot/AA) – 0.5 µM.

#### *1.2.5.2. GlycoRate Test*

Seahorse XF Glycolytic Rate assay (GlycoRate 103344-100 kit, Agilent) was performed as described by the manufacturer, using the following drug concentrations: Rotenone/Antimycin A – 0.5 µM, 2-deoxy-D-glucose (2-DG) – 50 mM.

#### *1.2.5.3. Substrate Oxidation Stress Test*

Seahorse XF Substrate Oxidation Stress (Long Chain Fatty Acid 103672-100 kit) was performed as described by the manufacturer, using the following drug concentrations: Etomoxir (E) – 4 µM, Oligo – 1.5 µM, FCCP – 2 µM and Rot/AA – 0.5 µM. Conditions were etomoxir was not injected are identified as medium only (M).

### *1.2.6. Glucose uptake*

Cells were seeded and transfected as described in 1.2.2 and in 1.2.4. Cells were then starved (glucose/FBS-free medium) for 3 hours and incubated with 2-(N-(7-Nitrobenz-2-oxa-1,3-diazol-4-yl)Amino)-2-Deoxyglucose (2-NBGD, N13195, ThermoFisher

Scientific) for 30 minutes. Following incubation, cells were fixed with 4% paraformaldehyde for 15 minutes at room temperature and counter-stained with Hoechst (1µg/mL, 33342, ThermoFisher Scientific) for 10 minutes. Coverslips were mounted using anti-fading agent (DAKO, S3023, Agilent) and imaged with Axiocam Fluo (Zeiss). Relative signal intensity of 2-NBDG was measured using CellProfiler v4.2.1. software and normalized to cell number.

### *1.2.7. Insulin stimulation*

Cells were seeded and transfected as described in 1.2.2 and in 1.2.4. Cells were grown until 70% confluence, starved (FBS-free medium) for 6 hours and stimulated with insulin (Mixtard, $10^{-7}$M) for 15 minutes or not. Following stimulation, cells were flash frozen using liquid nitrogen.

### *1.2.8. Morphological assessment of lipid droplets accumulation in hepatocytes (steatosis)*

Huh7 cells and HLOs were cultured and transfected as described in 1.2.2 - 1.2.4. Following 24-hours post-transfection, Huh7 cells and HLOs were exposed to fatty acid (FA)-enriched medium for 48 hours/ 7 days (Oleate (OA) 100 µM, Oleate:Palmitate (OA:PA) 400:200 µM or OA:PA 100:50 µM) or not (basal conditions). Following incubation with FA-enriched medium, Huh7 cells and HLOs were fixed with 4% paraformaldehyde at room temperature for 15 minutes (Cells) or 2 hours (HLOs), stained for neutral lipids with BODIPY (1 µg/mL, D3922, Molecular probes), counter-stained with Hoechst (1µg/mL, 33342, ThermoFisher Scientific) for 10-20 minutes and imaged with Axiocam Fluo (Zeiss) or with Nikon A1r Spectral (Nikon).

### *1.2.9. Establishment of inflammation/ fibrosis in human liver organoids*

HLOs were cultured as described in 1.2.2. At day 21 HLOs were exposed to a mixture of cytokines (TNFα, TGFβ, IL-6 and IL-1β, 10ng/mL each) for 3 days or to FA-enriched medium for 7 days (as described in 1.2.8.). Following incubation with cytokines/FAs, HLOs were collected and expression of inflammatory/fibrotic markers was assessed through RT-qPCR.

### *1.2.10. Determination of the cellular mitochondrial mass*

Huh7 cells were cultured and transfected as described in 1.2.2 and in 1.2.4. To observe mitochondrial morphology and quantify mitochondrial mass, cells were stained at 72-

hours post-transfection with MitoTracker™ Red CMXRos (200 nM, M7512, ThermoScientific) for 20 minutes at 37°C. Subsequently, cells were fixed with 4% paraformaldehyde for 15 minutes at room temperature and counter-stained with Hoechst (1µg/mL, 33342, ThermoFisher Scientific) for 10 minutes. Coverslips were mounted using anti-fading agent (DAKO, S3023, Agilent) and imaged with Axiocam Fluo (Zeiss). In parallel, mitochondrial DNA was purified using the QiAmp ® DNA Micro kit (56304, Qiagen). Then, amplification of nuclear and mitochondrial DNA was performed and the mitochondrial/nuclear DNA ratio was calculated as previously described (5).

### *1.3. Polysome fractionation*

Huh7 cells were cultured and transfected as described in 1.2.2 and in 1.2.4. and collected while in proliferative stage (70-80% confluency). Cycloheximide (CHX, 100 µg/ml) was added to the medium and cells were incubated at 37°C for 10 min in a humidified incubator. Plates were washed with 10 ml ice-cold 1xPBS (calcium and magnesium-free, Gibco) supplemented with CHX. Cells were collected using 0.25% trypsin supplemented with CHX for 10 min and centrifuged at 800 rpm, 4°C for 5 min. Cells were then lysed in 2 volumes of lysis buffer (20 mM Tris, pH 7.4, 140 mM KCl, 5 mM MgCl2, 1.0% Triton X-100, 1 mg/ml Heparine, 25 U/ml Turbo DNase I, 1mM DTT, 100 µg/ml CHX, Protease inhibitors (Roche), 0.25 U/ml SUPERaseIn RNase inhibitor (Ambion, #AM2694)) and by passing 10-12 times through 25G needle. Extracts were centrifuged at 20000 g, 4°C for 20 min. Cell lysates were loaded on linear 20-60% sucrose gradients prepared with gradient buffer (20 mM Tris, pH 7.4; 140 mM KCl; 5 mM MgCl2; 1 mM DTT; 0.1 mg/ml CHX). Ribosomes were fractionated at 247’600 g (38’000 rpm, rotor SW41 Ti (Beckman Coulter, #331362) for 3 h 30 min at 4°C. Fractionated ribosomes were monitored and collected using Density Gradient Fractionation System (ISCO).

### *1.4. RNA extraction and Real-time qPCR*

RNA extraction from ribosomal fractions, flash frozen cells, HLOs and mouse tissues was performed using Trizol (Ambion, Thermo Scientific, USA) according to manufacter’s instructions. RNA concentration was measured using NanoDrop (Thermo Scientific). Prior to real-time qPCR, reverse transcription was performed using the High-Capacity cDNA Reverse Transcription kit (Applied Biosystems™). For miRNAs expression, the reverse transcription was performed as previously described (6). qPCR

was performed using the PowerUp™ SYBR™ Green Master Mix for Real-Time PCR and the QuantStudio 5 Real-time PCR System and data analysis software (Applied Biosystems™), according to manufacturer's specifications. Primer sequences used are described in *Table S2*. Gene expression was quantified using the ΔΔCT method.

### *1.5. Western Blot*

Protein extraction from flash frozen cells and mouse tissues was performed using RIPA buffer (50 mM Tris-HCl, pH 6.8, 100 mM DTT, 2%SDS, 0.1% bromophenol blue, 10% glycerol). Tissues were homogenized using TissueLyser. Protein lysates were centrifuged at 12000 g for 10 minutes and the supernatant collected. Protein concentration was determined using BCA protein assay kit (Pierce Biotechnology). 5-10 μg of protein was charged in 5-20% gradient sodium dodecyl sulfate-polyacrylamide gel electrophoresis (SDS-PAGE) gels and transferred to nitrocellulose membranes (RPN303D, Amersham, Switzerland). Membranes were blocked with polyvinyl alcohol and incubated overnight with primary antibody at 4°C. Membranes were washed with 0.1% TBS-Tween and incubated with secondary antibodies at room temperature for 1 hour. Membranes were then incubated with ECL Prime Substrate (RPN22232, Amersham, Switzerland) for 1 minute and revealed using the PXI/PXI Touch system (Syngene, Synoptics group, UK) or Fusion instrument (Vilber, France). Between each step, membranes were washed with 0.1% TBS-Tween. Signal was quantified using GeneSys (Syngene, Synoptics group, UK) or ImageJ™ software. Antibodies used are described in *Table S2*.

### *1.6. Histology, Immunohistochemistry and immunofluorescence*

Mouse tissues were fixed overnight with 4% paraformaldehyde (PFA) while HLOs were fixed for 2 hours in 4% PFA and then transferred to PBS for dehydration and paraffin-embedding. Tissue and HLOs specimens were cut into 5 μm sections prior to staining or immunohistochemistry/immunofluorescence. For morphological analysis, tissue sections from 3 samples collected from different lobes of the explanted liver were stained with hematoxylin and eosin while for fibrosis analysis, sections were stained with Sirius Red. For immunohistochemistry and immunofluorescence, tissue/HLOs sections were deparaffinized, rehydrated and heated in citrate buffer or treated with Proteinase K for antigen retrieval. Subsequently, sections were permeabilized with

0.3% Triton X-100 in TBS for 15 minutes, blocked with 10% goat serum (ab138478, Abcam) in 1% BSA-TBS for 2h at room temperature and incubated overnight at 4°C with primary antibody diluted in 1% BSA-TBS. Endogenous peroxidase was blocked by incubation with 0.3% $H_2O_2$ solution for 15 minutes at room temperature, prior to incubation with secondary antibody at room temperature for 1 hour. Between each step, slides were washed in 0.025% Triton X-100 in TBS. Signal was revealed following an incubation with DAB Substrate kit (ab64238, Abcam, UK) or with secondary antibodies conjugated with a fluorophore and counterstained with hematoxylin for 3 minutes or with Hoechst (1 µg/mL, 33342, ThermoFisher Scientific) for 10 minutes. Following staining or immunohistochemistry/immunofluorescence, slides were mounted and scanned in AxioScan Z.1 (Zeiss) or imaged in Axiocam Fluo (Zeiss) for subsequent analysis. The slides from the different experimental groups were then analyzed blindly by two different researchers. Antibodies and concentrations used are listed in *Table S2*.

### *1.7. Image analysis*

Batched analysis and quantification of lipid droplet number/size per $µm^2$ of tissue were performed using QuPath software (version 0.4.3.) and CellPose package (version 2.0)(6) following pretrained automated tissue/lipid droplet detection. Sirius red positive areas per $µm^2$ of tissue were detected using QuPath's pixel classifier default settings following pretrained automated tissue detection.

Relative BODIPY and MitoTracker™ Red CMXRos signal in Huh7 cells was measured with CellProfiler v4.2.1. and normalized to cell number. Single lipid droplets morphological aspects in Huh7 cells were also analyzed with CellProfiler (number, area, diameter).

For HLO image analysis, 3D confocal stacks, acquired with multiple wavelengths, were automatically processed using a dedicated framework developed in Matlab R2023a (The MathWorks). Specifically, Nikon nd2 files were accessed through the Bio-formats package (7). Organoid localization was achieved by the maximum voxel-wise of all available channels following intensity normalization. Subsequently, alternating sequential filters and Otsu's method (8) for global image segmentation were employed. Nuclei, labeled with DAPI and/or HNF4, and lipid droplets were segmented following anisotropic diffusion filtering using Cellpose 2.0 (6) in 3D, with its pre-trained models for "nuclei" or "cyto". The resulting two sets of nuclei were combined to identify hepatocyte

nuclei. Lipid droplets were assigned to specific cell nuclei based on the shortest Euclidean distance centroid to centroid. Finally, the results were quantified in terms of organoid volume, hepatocyte cell-proportions in the organoids, total lipid droplet volume, and mean fluorescent intensities in HNF4-positive or -negative nuclei.

### *1.8. Glycogen, glucose and glucose-6-phosphate content assay*

Frozen liver tissues (100 mg) from mice fed FPC diet for 24 weeks (n=9-10 per group) were lysed in 8 volumes of perchloric acid (6%). Lysates were centrifuged at 10'000 g for 15 min at 4°C. Supernatants were collected and neutralized with potassium carbonate ($K_2CO_3$, 3.2mM) until pH reached 6.5-8.5. Following another centrifugation at 10 000 g for 15 min at 4°C, glycogen content was measured with Keppler and Decker method, as previously described (7). Glycogen was partially hydrolyzed in NaOH (0.15 M) for 20 minutes at 100ºC and digested by α-amidoglucosidase for 1h at 45°C into glucose. Glucose was measured after the addition of hexokinase (0.7U/mL) and glucose-6-phosphate was measured after the addition of $NADP^+$(0.9 mM) and glucose-6-phosphate dehydrogenase (0.7U/mL). NADPH production was detected at 340 nm.

### *1.9. Triglyceride assay*

Intra-hepatic triglycerides were measured using the Triglyceride-Glo™ assay (Promega) according to manufacturer's instructions. The results obtained for the FPC diet were further validated using the <Folch= method as described in (9).

### *1.10. Microarray analysis*

Livers from 4 months old LPTENKO (*Pten*$^{lox/lox}$, AlbCre$^{+/-}$) mice and wild-type littermates (*Pten*$^{lox/lox}$, AlbCre$^{-/-}$; n=3 per group) were used for miRNAs microarray analysis. RNA was extracted as mentioned in section 1.4. and 500 ng of total RNA was used for subsequent analyses. Microarray miRNA expression profiles of were performed in the Genomics platform at University of Geneva. miRNA expression profiles were obtained using the Affymetrix GeneChip ® miRNA 3.0 Array (Affymetrix). After quality control, data was normalized and summarized using the robust multichip analysis (Affymetrix Microarray Suite). Partek was used to determine ANOVA p-values and fold-changes. Only miRNAs respecting the established threshold (p-value<0.05,
!fold-change (FC)! > 1.5) were considered for analyses.

### *1.11. RNA sequencing*

RNA extracted from ribosomal fractions (polysome fractions only) of Huh7 cells overexpressing miR-149 (Mimic 149) or scrambled synthetic oligonucleotides (Control Mimic) was used to perform high-throughput sequencing. Poly-A selected RNA was used for library preparation using TruSeq RNA Sample preparation kit (Illumina) andsubmitted to 100 nt single-end read TruSeq HT stranded mRNA sequencing protocol in an Illumina NovaSeq 6000 system, according to manufacturer's indications. Reads were processed with FastQC v.0.11.9, STAR v.2.7.4a, PicardTools v2.21.6, and HTSeq v0.9.1 for biological quality control, mapping, alignment and preparation of table of counts. Normalization and differential expression analysis were performed using the edgeR v3.38.4 R package. Data was normalized by sequencing depth and RNA composition and differentially expressed genes (DEGs) were estimated using the negative binomial general model. Subsequently, DEGs between conditions were identified after establishment of thresholds for statistical significance and magnitude of change (fold change (FC)=!2! and false discovery rate (FDR)<0.05).

### *1.12. In silico analysis*

#### *1.12.1. Over Representation Analysis*

Over Representation Analysis (ORA) was performed using clusterProfiler v4.9.0 and edgeR package. Deregulated genes were identified using the following thresholds: p-value with a false discovery rate (FDR, Benjamini & Hochberg) <0.05, fold-change to Control Mimic condition = !1.5! and classified based on gene ontology by biological processes (GO:BP) and by KEGG pathways.

#### *1.12.2. Identification of potential targets and related biological functions*

Predicted and validated targets of miR-149 in human and mouse genomes were retrieved using the miRWalk database (http://mirwalk.um.uni-heidelberg.de/, accessed on 15/06/2023). This list was cross-referenced with DEGs identified in the RNA seq analysis after converting gene symbols to mouse orthologs using g:Orth on g:profiler (https://biit.cs.ut.ee/gprofiler/orth). Potential downregulated targets shared between human and mouse or exclusively human were considered for GO analysis by KEGG pathways using shinyGOv0.77 application (http://bioinformatics.sdstate.edu/go/, accessed on 10/08/2023). Shared targets were screened in PubMed to verify functional characterization of each gene in the context of MASLD development. The literature

screening was performed using the gene name and the following terms: <Obesity=, <Diabetes=, <Insulin Resistance=, <Steatosis=, <Inflammation=, <Fibrosis=, <Liver=, <NAFLD=, <MASLD=, <NASH= and <MASH=. Only studies using in vivo models were considered.

### *1.13. Statistical analysis*

For animal experimentation we have performed power analysis (G*Power software, v. 3.1.9.7, p=0.1 (t-test), effect size 1.1, power: 0.9) to calculate the minimum number of animals necessary to observe an expected effect of 40% reduction in regards to steatosis between shCTL and shmiR149 mice. This analysis allowed to determine a minimum of 12 mice per group. Statistical analyses were performed using GraphPad Prism 8 Software (GraphPad Software, San Diego, CA, USA). Results are represented as mean ± standard deviation (SD). Outliers test was performed using the ROUT method (Q = 1%). Unpaired t-test with Welch's correction was performed to compare two groups. One-way ANOVA test with Holm-Sidak correction was applied to compare more than two groups. To evaluate the independence of categorical variables, chi-square test or Fisher's test was used. Results of statistical tests are represented in figure legends as follows: * p-value < 0.05, ** p-value < 0.01, *** p-value < 0.001, **** p-value < 0.0001.

**Supplementary methods table 1** – Composition of the different diets used.

| Diet | CD | | WD | | THD | | 0-3D | | FPC | | MCD | |
|---|---|---|---|---|---|---|---|---|---|---|---|---|
| Reference | D13012801 | | D13012802 | | D13012803 | | D13012804 | | TD.190142 | | E15653-94 | |
| Company | | | | | | | | | Envigo | | ssniff | |
| % | g | kcal | g | kcal | g | kcal | g | kcal | g | kcal | g | kcal |
| Protein | 19.2 | 20 | 23.7 | 20 | 23.7 | 20 | 23.7 | 20 | 12.2 | 9.8 | 15 | 14 |
| Carbohydrate | 67.3 | 70 | 41.4 | 35 | 41.4 | 35 | 41.4 | 35 | 46.2 | 37.3 | 64.3 | 64 |
| Fat | 4.3 | 10 | 23.6 | 45 | 23.6 | 45 | 23.6 | 45 | 29.1 | 52.9 | 10 | 22 |
| Total | 100 | | 100 | | 100 | | 100 | | 100 | | 100 | |
| kcal/gm | 3.85 | | 4.73 | | 4.73 | | 4.73 | | 5 | | 4.73 | |

**Supplementary methods table 2 –** List of primers used and respective sequences.

| Gene | Forward | Reverse | Species |
|---|---|---|---|
| miR-149 | TCT GGC TCC GTG TCT TCA CT | - | |
| miR-182 | TTT GGC AAT GGT AGA ACT CAC AC | - | |
| miR-183 | TAT GGC ACT GGT AGA ATT CAC TAA | - | |
| miR-122 | GGCTGTGGAGTGTGACAATG | - | |
| miR-16 | ACAGCCTAGCAGCACGTAAAT | - | |
| univ-RT-polyT | | GAGGTATTCGCACCAGAGGATTTTTTTTTTTTTTTVN | |
| *Vim* | CGG CTG CGA GAG AAA TTG C | CCA CTT TCC GTT CAA GGT CAA G | Mouse |
| *Fn1* | ATCTCGGAGCCATTTGTTCCT | CCAGGTCTACGGCAGTTGTCA | |
| *Col1a1* | GCT CCT CTT AGG GGC CAC T | CCA CGT CTC ACC ATT GGG G | |
| *Acta* | AAAAAAAACCACGAGTAACAAATCAA | TCAGCGCCTCCAGTTCCT | |
| *Pdgfrb* | GAGGCTTATCCGATGCCTTCT | AGACATGTTGCGAGTAGACAAAACTAA | |
| *Krt18* | CAG CCA GCG TCT ATG CAG G | CCT TCT CGG TCT GGA TTC CAC | |
| *Il6* | AGT TGC CTT CTT GGG ACT GAT | TCC ACG ATT TCC CAG AGA AC | |
| *Il10* | CTTTCAAACAAAGGACCAGC | CCAAGTAACCCTTAAAGTCCT | |
| *Il1b* | GACAACTGCACTACAGGC | CATGGAGAATATCACTTGTTGG | |
| *Tgfb* | CAACATGTGGAACTCTACCAG | TGTATTCCGTCTCCTTGGT | |
| *Tnfa* | AGGCTGCCCCGACTACGT | GACTTTCTCCTGGTATGAGATAGCAAA | |
| *Itgam* | ATG GAC GCT GAT GGC AAT ACC | TCC CCA TTC ACG TCT CCC A | |
| *Fgf21* | CAGTCCAGAAAGTCTCCTG | GATCAAAGTGAGGCGATCC | |
| *Cd36* | GTCTATCTACGCTGTGTTCG | ACAGGCTTTCCTTCTTTGC | |
| *Acc1* | GGACACCAGTTTTGCATTGA | AGTTTGGGAGGACATCGAAA | |
| *Fasn* | AAGTTGCCCGAGTCAGAGAACC | ATCCATAGAGCCCAGCCTTCCATC | |
| *Cpt1a* | ATGGCAGAGGCTCACCAAGC | GATGAACTTCTTCTTCCAGGAGTGC | |
| *Acox1* | CATGAATCCCGATCTGCG | TCAAGTTCTCGATTTCTCGAC | |
| *Fabp4* | CACCGAGATTTCCTTCAAACTG | TTTCATAACACATTCCACCACC | |
| *Fatp5* | TACAAGTTGGAGCCACCTG | TCACCCACATACAAGATCACTG | |
| *Hmgcr* | GTACATTCTGGGTATTGCTGG | GCACTCGCTCTAGAAAGG | |
| *NANOG* | TTT GTG GGC CTG AAG AAA ACT | AGG GCT GTC CTG AAT AAG CAG | Human |
| *CDX2* | GAC GTG AGC ATG TAC CCT AGC | GCG TAG CCA TTC CAG TCC T | |
| *FOXA2* | AGCGGTGAAGATGGAAGG | GTGTTCATGCCGTTCATCC | |
| *HNF4A* | CTCCTGCAGATTTAGCCG | CTGTCCTCATAGCTTGACC | |
| *ALB* | CTAGAGAAGTGCTGTGCC | CCACGGATAGATAGTCTTCTG | |
| *SERPINA1* | CTTCTTTAAAGGCAAATGGGAG | CTGGACAGCTTCTTACAGTG | |
| *ALCAM* | TCC TGC CGT CTG CTC TTC T | TTC TGA GGT ACG TCA AGT CGG | |

| | | | |
|---|---|---|---|
| *CD68* | GGA AAT GCC ACG GTT CAT CCA | TGG GGT TCA GTA CAG AGA TGC | |
| *ACTA* | TGATCACCATCGGAAATGAA | CGGCTTCATCGTATTCCTGT | |
| *COL1A1* | AACATGACCAAAAACCAAAAGTG | CATTGTTTCCTGTGTCTTCTGG | |
| *IL6* | AAATTCGGTACATCCTCGACGG | GGAAGGTTCAGGTTGTTTTCTGC | |
| *IL8* | CTGCGCCAACACAGAAATTA | ATTGCATCTGGCAACCCTAC | |
| *IL1B* | ATG GCT TAT TAC AGT GGC AA | GTC GGA GAT TCG TAG CTG GA | |
| *TGFB* | GTGACCTGGCCACCATTCAT | GTCAATGTACAGCTGCCGCA | |
| *VIM* | GCCCTAGACGAACTGGGTC | GGCTGCAACTGCCTAATGAG | |

**Supplementary methods table 3 –** List of antibodies used and respective application.

| Protein | Antibody production (Catalog number) | Dilution | Application |
|---|---|---|---|
| phospho AKT Ser | cell signaling (9271) | 1/1000 | WB |
| Total AKT | cell signaling (9272) | 1/1000 | WB |
| Tubulin | cell signaling (2128) | 1/1000 | WB |
| HNF4a | Santa-Cruz (SC-8987) | 1/1000 | WB |
| Phospho JNK | Cell signaling (9251) | 1/1000 | WB |
| Total JNK | Cell signaling (9252) | 1/1000 | WB |
| Phospho NFκB | Cell signaling (3037) | 1/1000 | WB |
| Total NFκB | Santa-Cruz (SC-372) | 1/1000 | WB |
| Phospo p38 | Cell signaling (4511) | 1/1000 | WB |
| p38 | Cell signaling (8690) | 1/1000 | WB |
| ERM | Cell signaling (3142) | 1/1000 | WB |
| PDGFR | abcam (ab32570) | 1/1000 | WB |
| HNF4a | Santa-Cruz (SC-8987) | 1/100 | IF |
| HNF4 - Alexa Fluor 555 (EPR16786) | abcam (ab217518) | 1/100 | IF |
| HNF4 - Alexa Fluor 647 (EPR3648) | abcam (ab217073) | 1/100 | IF |
| CD166 | abcam (ab109215) | 1/100 | IF |
| CD166 - Alexa Fluor 488 | abcam (ab197543) | 1/100 | IF |
| CD68 - Alexa Fluor 647 | abcam (ab213363) | 1/100 | IF |
| CD68 - Alexa Fluor 647 | abcam (ab224029) | 1/100 | IF |
| Iba1 | abcam (ab178846) | 1/2000 | IF |
| GFP | Cell signalling (2956) | 1/400 | IF |

**Supplementary figures**

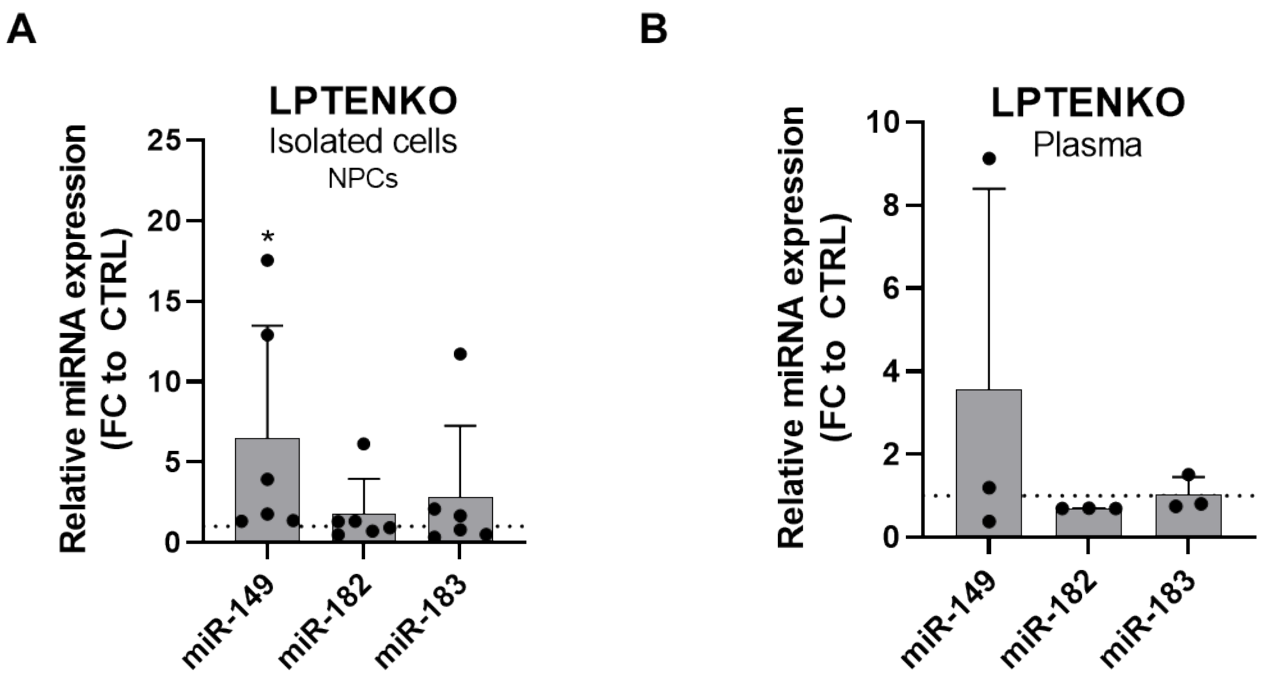


**Fig. S1 – MiR-149-5p expression is upregulated isolated non-parenchymal cells (NPC) and in plasma from LPTENKO mice .**
Relative miRNA expression of the top 3 most upregulated miRNAs identified in microarray analysis and validated through RT-qPCR in **(A)** non-parenchymal cells (NPC, n=4-6 per group) and **(B)** plasma (n=3 per group) of 4-months old CTRL and LPTENKO mice. Data is represented as mean ± SD. One-way ANOVA with Holm-Sidak's correction. *p-value < 0.05, **p-value < 0.01, ***p-value < 0.001, ****p-value < 0.0001.

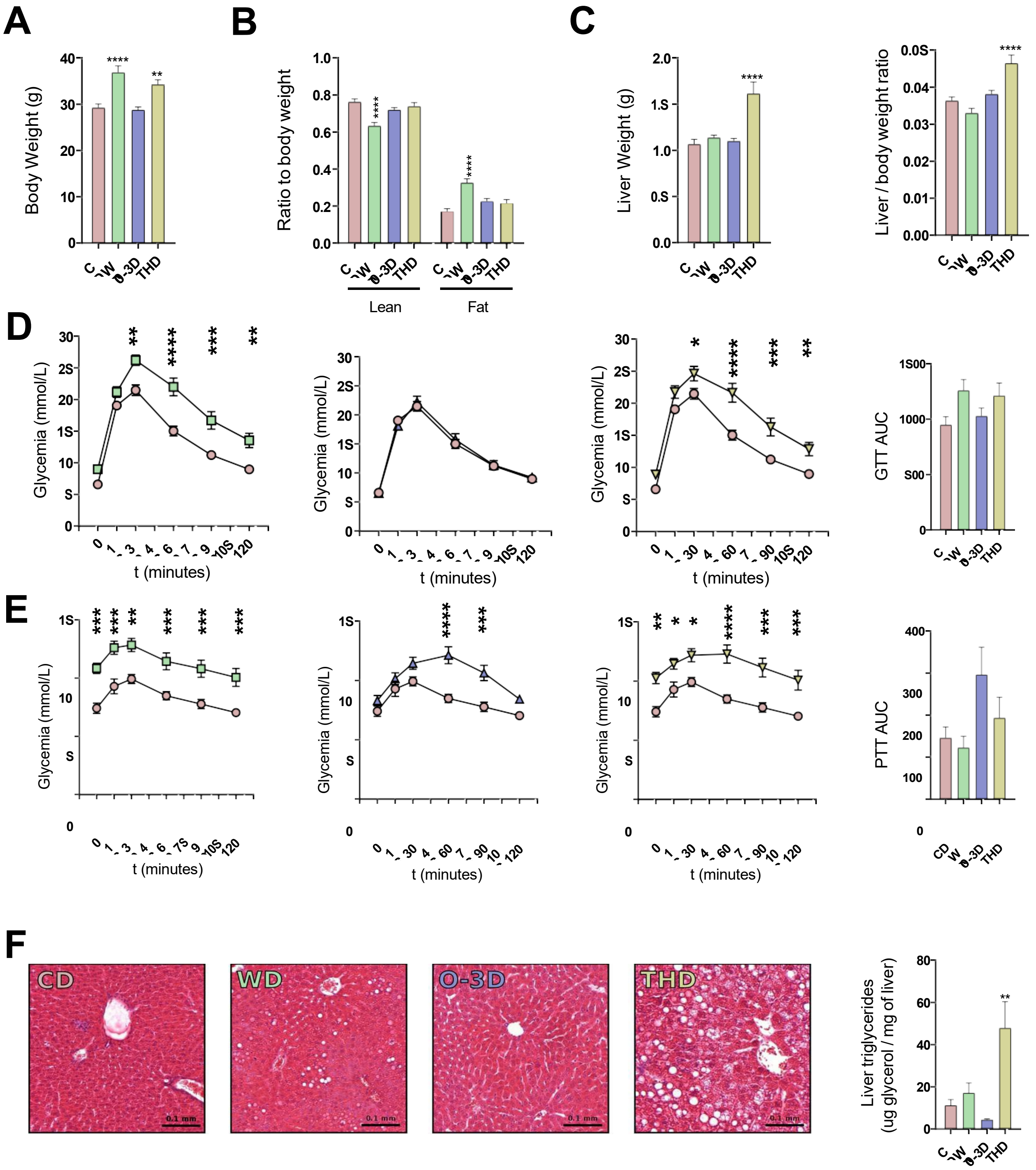


**Fig. S2 – Impact of different high-sugar / high-fat diets on mouse metabolic phenotype.** **(A)** Body weight, **(B)** body composition by echoMRI, **(C)** liver weight and liver/body weight ratio, **(D)** glucose tolerance test (GTT) and corresponding area under the curve (AUC), **(E)** pyruvate tolerance test (PTT) and corresponding area under the curve (AUC), **(F)** representative liver histology (hematoxylin and eosin staining) and **(G)** hepatic triglyceride content in 2-months old C57/BL6J mice fed for 16 weeks with control diet (CD, 10% cal from fat, 17% cal from sucrose) or with fatty acids-enriched diets (45% cal from fat, 17% cal from sucrose): either a mix of unsaturated/saturated fatty acids (Western diet, WD), omega-3 (O-3D) or trans-hydrogenated fatty acids (THD); n=6-12 per group. Data is represented as mean ± SD. One-way ANOVA with Holm-Sidak's correction. *p-value < 0.05, **p-value < 0.01, ***p-value < 0.001, ****p-value < 0.0001.

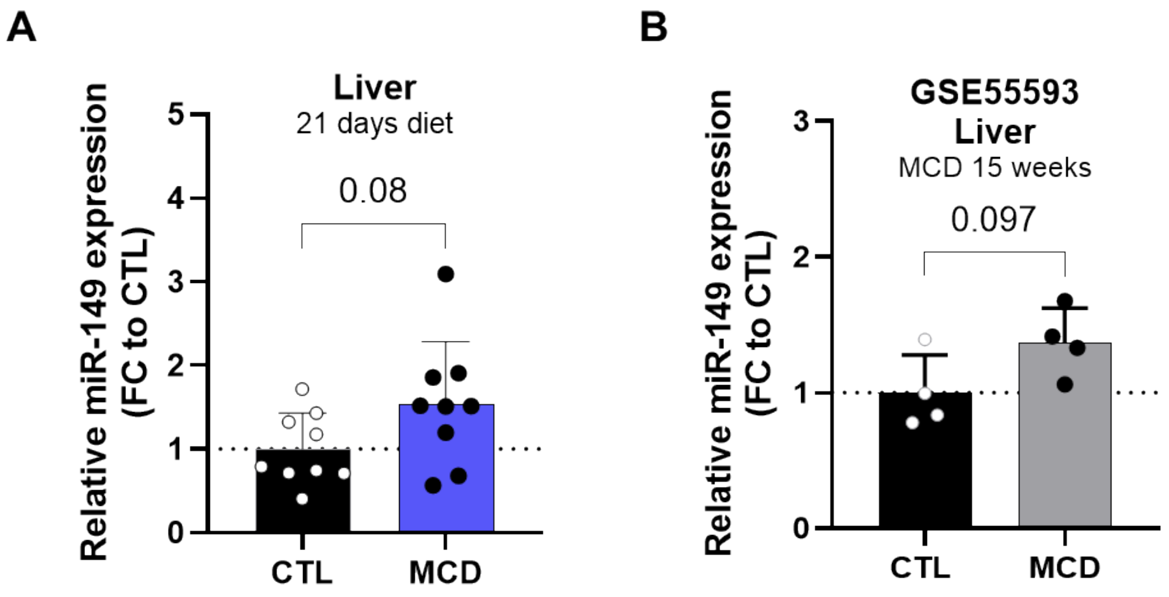


**Fig. S3 – miR-149-5p expression is increased in hepatic tissues of mice fed a methionine/choline deficient diet.**
**(A)** Relative miR-149-5p expression in hepatic tissue of diet-induced MASH model (21 days of methionine/choline deficient diet, left panel). **(B)** Gene expression omnibus (GEO) datasets analyses of miR-149-5p expression (FC to CTL) in hepatic tissues of mice fed MCD for 15 weeks (right panel). Unpaired t-test with Welch's correction. *p-value < 0.05, **p-value < 0.01, ***p-value < 0.001, ****p-value < 0.0001.

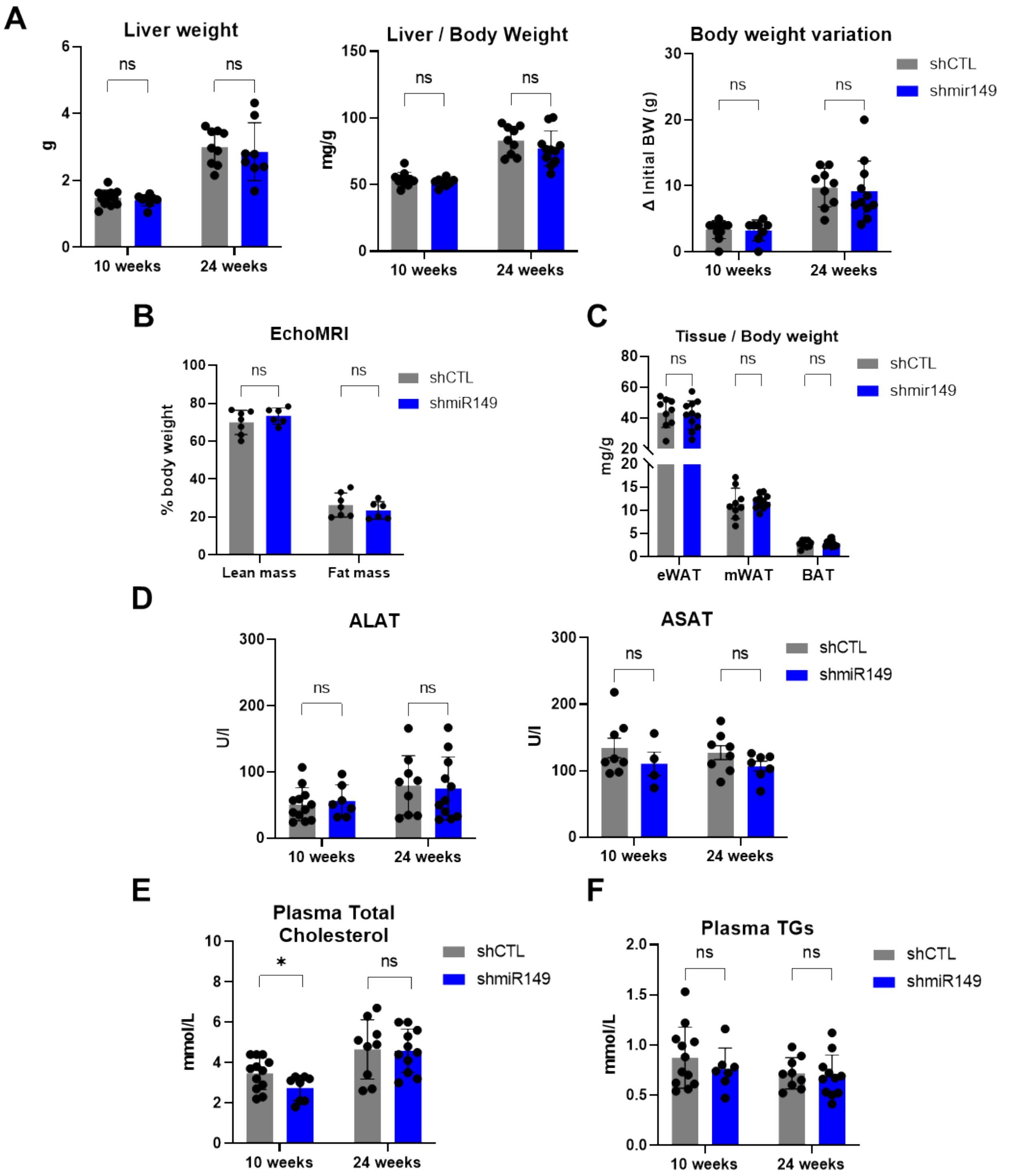


**Fig. S4 – In vivo downregulation of miR-149-5p specifically in hepatocytes does not alter overall body parameters upon fructose/palmitate/cholesterol (FPC) diet.**
**(A)** Liver weight, liver-to-body weight ratio and body weight gain of shCTL (n=9-12) and shmiR149 (n=8-11) mice fed FPC diet for 10 and 24 weeks. **(B)** EchoMRI analyses of lean and fat mass and **(C)** tissue-to-body weight ratio of explanted epidydimal (eWAT), mesenteric (mWAT) and brown (BAT) adipose tissue of shCTL (n=7-9) and shmiR149 (n=6-11) mice fed FPC diet for 24 weeks. Plasma levels of **(D)** transaminases (ALAT and ASAT), **(E)** triglycerides and **(F)** total cholesterol in shCTL (n=9-12) and shmiR149 (n=7-11) mice fed FPC diet for 10 and 24 weeks. Data is represented as mean ± SD. Unpaired t-test with Welch's correction or one-way ANOVA with Holm-Sidak's correction. *p-value < 0.05, **p-value < 0.01, ***p-value < 0.001, ****p-value < 0.0001.

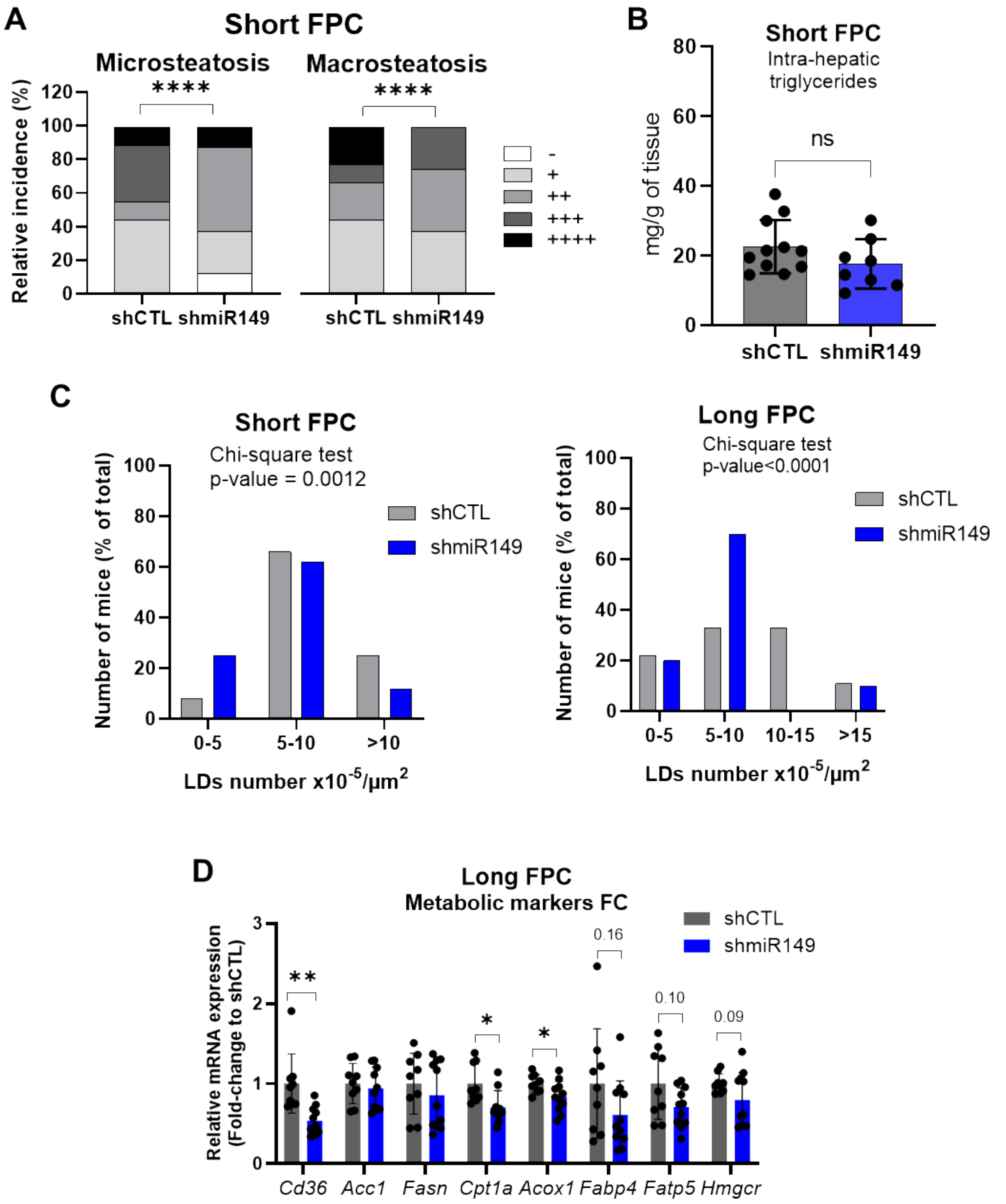


**Fig. S5 – In vivo downregulation of miR-149-5p specifically in hepatocytes attenuates hepatic steatosis incidence and decreased markers of lipid uptake/ oxidation upon fructose/palmitate/cholesterol (FPC) diet.**
(**A**)Blind scoring of micro-/macrosteatosis, **(B)** intra-hepatic triglycerides content, (**C)** Distribution of lipid droplet (LD) number per μm$^2$ of liver tissue area and **(D)** relative mRNA expression of metabolic markers in the explanted livers of FPC-fed mice after injection with hepatotropic adeno-associated virus (AAV8) harboring vectors encoding for scrambled shRNAs (shCTL, Long FPC - n=9; Short FPC – n=11) or for shRNAs specific for miR-149-5p (shmiR149, Long FPC - n=10-11; Short FPC n=8). Data is represented as mean Fold-change to shCTL ± SD. Unpaired t-test with Welch's correction, Fisher's test or Chi-square test. *p-value < 0.05, **p-value < 0.01, ***p-value < 0.001, ****p-value < 0.0001.

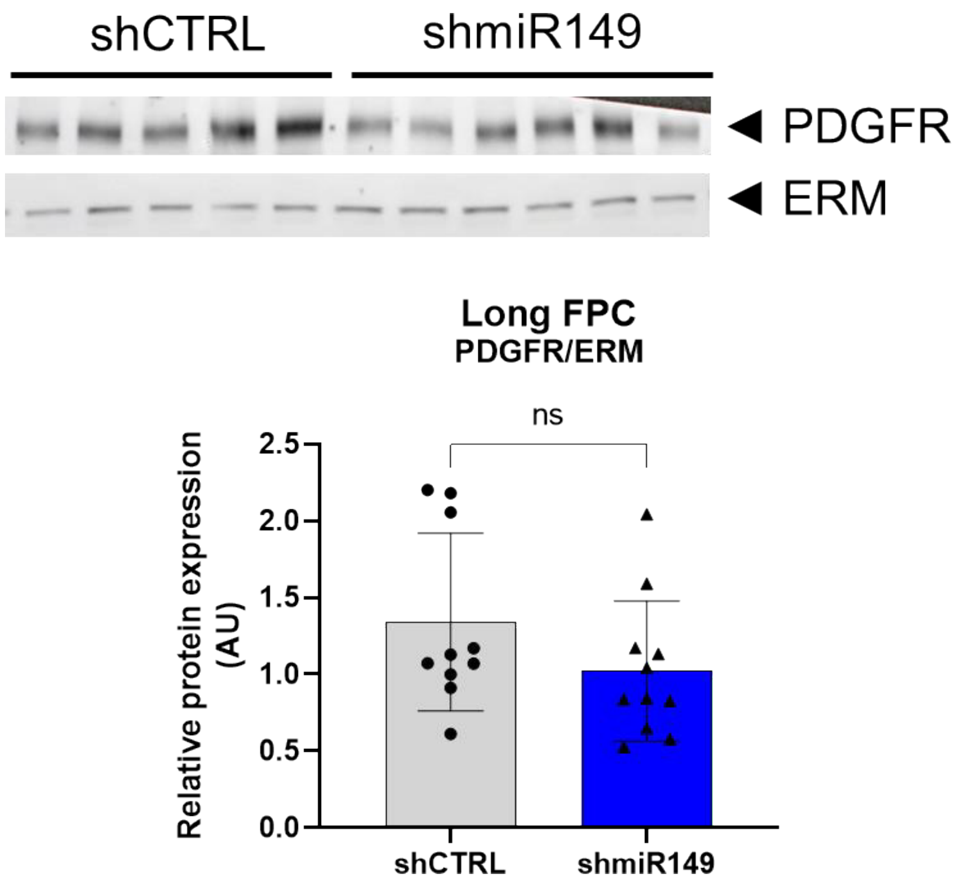


**Fig. S6 – Inhibition of miR-149-5p in mice fed fructose-palmitate-cholesterol (FPC) diet tends to decrease PDGFR protein levels.**
Western blot analysis (representative images on the left, n=5 shCTRL and n=6 shmiR149; quantifications on the right) of PDGFR protein expression in hepatic tissues of shCTL (n=10) and shmiR149 (n=11) mice after 24 weeks of FPC diet. ERM protein levels were used loading control. Data is represented as mean ± SD. Unpaired t-test with Welch's correction. *p-value < 0.05, **p-value < 0.01, ***p-value < 0.001, ****p-value < 0.0001.

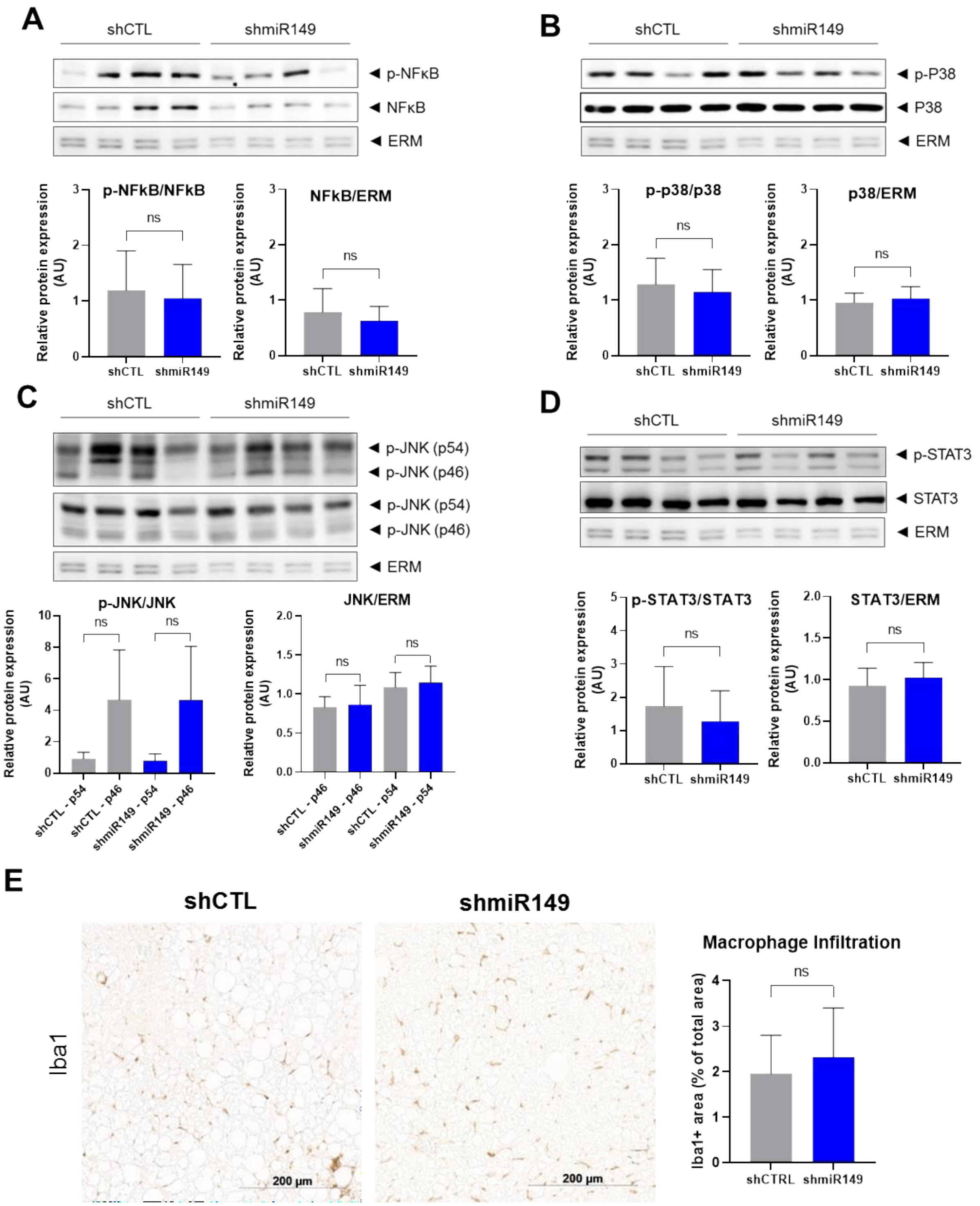


**Fig. S7 – Inhibition of miR-149-5p in mice fed fructose-palmitate-cholesterol (FPC) diet does not impact inflammatory pathways nor macrophage infiltration.**

Western blot analysis (representative images on the left, 4 mice per group; quantifications on the right) of phosphorylated over total expression of **(A)** NFκB, **(B)** p38, **(C)** JNK and **(D)** STAT3 signalling pathways in hepatic tissues of shCTL (n=9) and shmiR149 (n=11) mice after 24 weeks of FPC diet. ERM protein levels were used loading control. **(E)** Representative immunohistochemical staining of Iba1 in liver sections (left) and quantification of positive area over total tissue area (right) in shCTL (n=4) and shmiR149 (n=10) mice after 24 weeks of FPC diet. Data is represented as mean ± SD. Unpaired t-test with Welch's correction. *p-value < 0.05, **p-value < 0.01, ***p-value < 0.001, ****p-value < 0.0001.

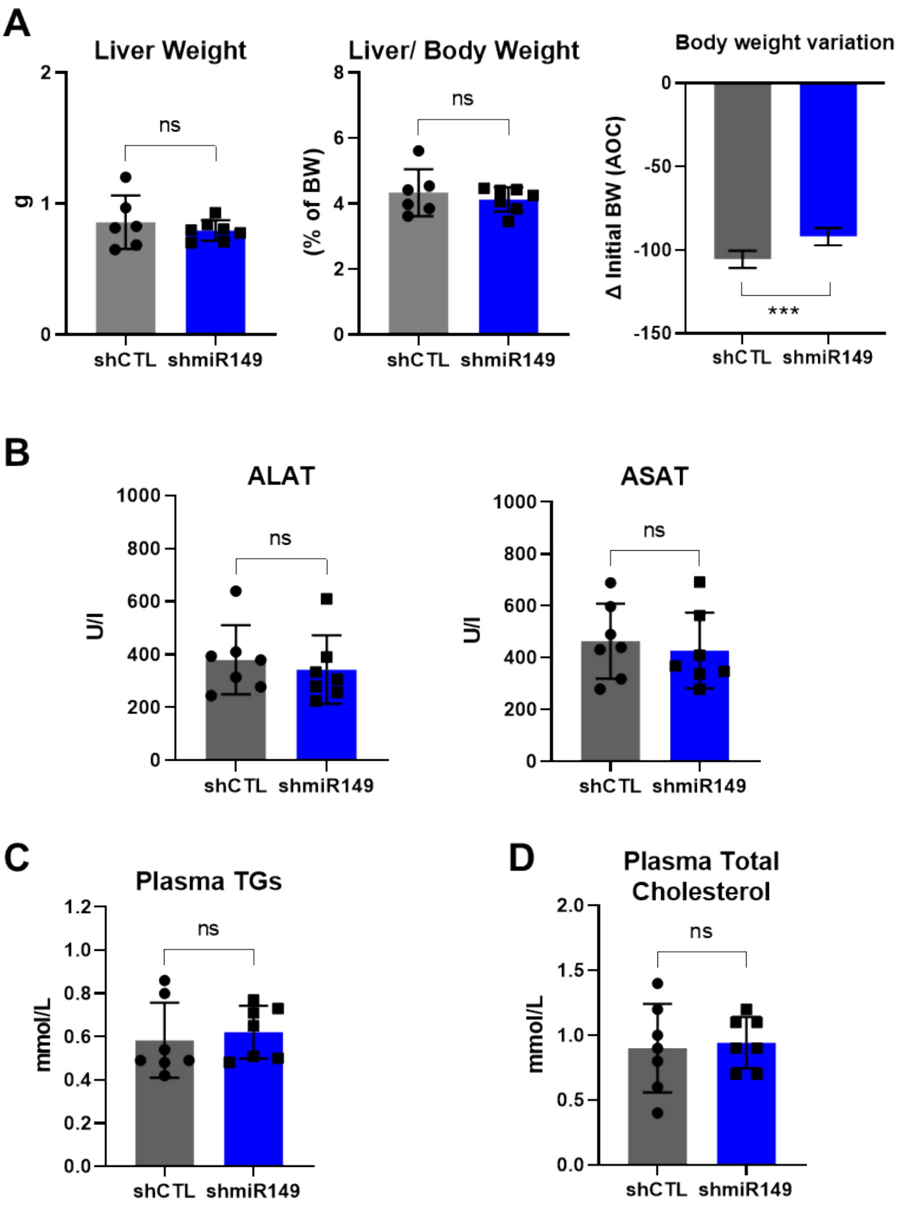


**Fig. S8 – In vivo downregulation of miR-149-5p specifically in hepatocytes does not alter overall body parameters upon methionine-choline-deficient (MCD) diet.**

**(A)** Liver weight, liver-to-body weight ratio (%) and body weight variation, **(B)** plasma levels of transaminases (ALAT and ASAT), **(C)** plasma triglycerides and **(D)** total cholesterol content in shCTL and shmiR149 fed MCD diet for 19 days after injection with hepatotropic adeno-associated virus (AAV8) harboring vectors encoding for scrambled shRNAs (shCTL) or for shRNAs specific for miR-149-5p (shmiR149, n=7 per group). Data is represented as mean ± SD. Unpaired t-test with Welch's correction. *p-value < 0.05, **p-value < 0.01, ***p-value < 0.001, ****p-value < 0.0001.

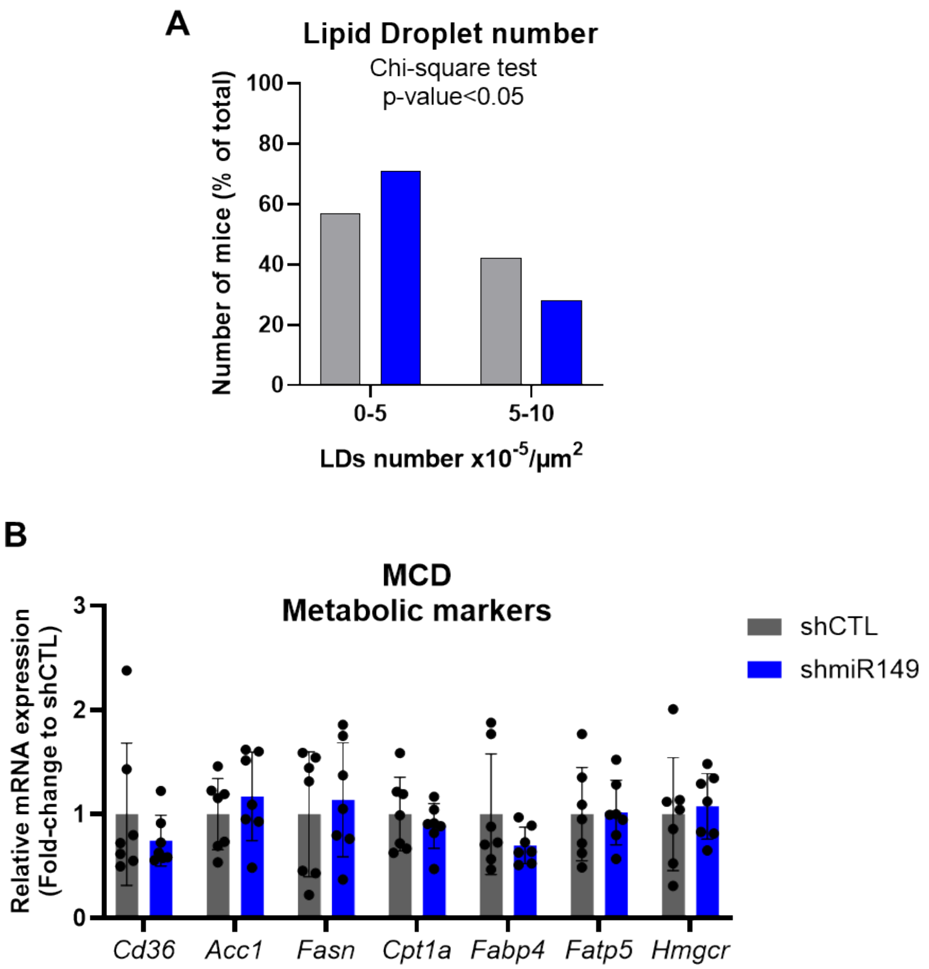


**Fig. S9 – In vivo downregulation of miR-149-5p specifically in hepatocytes attenuates steatosis upon methionine-choline-deficient (MCD) diet.**

**(A)** Distribution of lipid droplet (LD) number per µm$^2$ of liver tissue area and **(B)** relative mRNA expression of metabolic markers in the explanted livers of MCD-fed mice after injection with hepatotropic adeno-associated virus (AAV8) harboring vectors encoding for scrambled shRNAs (shCTL, n=7) or for shRNAs specific for miR-149-5p (shmiR149, n=7). Data is represented as mean Fold-change to shCTL ± SD. Chi-square test or Unpaired t-test with Welch's correction. *p-value < 0.05, **p-value < 0.01, ***p-value < 0.001, ****p-value < 0.0001.

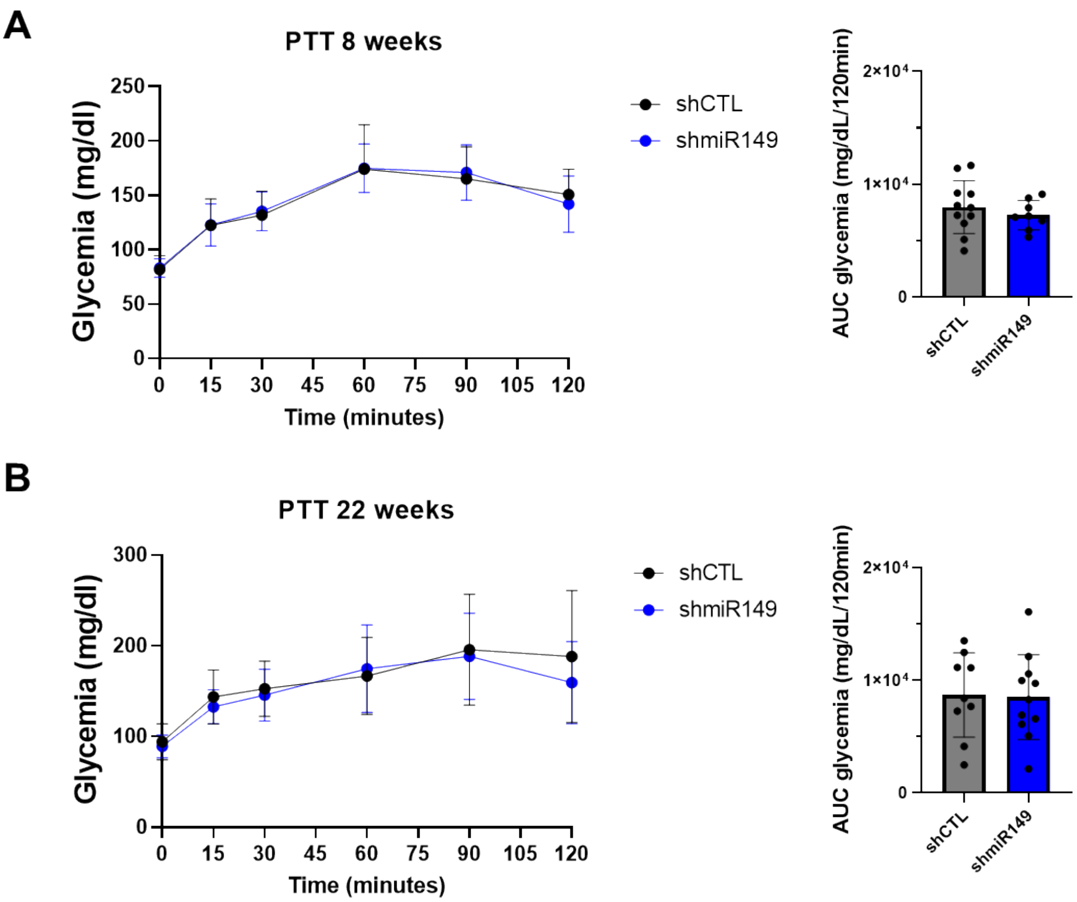


**Fig. S10 – Inhibition of miR-149-5p in mice fed fructose-palmitate-cholesterol (FPC) diet does not affect hepatic glucose output**.

Intraperitoneal pyruvate tolerance test (PTT, left panels) and calculated area under the curve (AUC, right panels) after 18 hours of fasting at **(A)** 8 and **(B)** 22 weeks of FPC-fed in shCTL and shmiR149 mice (n=8-11 per group at 8 weeks, n=9-11 per group at 22 weeks). Data is represented as mean ± SD. Unpaired t-test with Welch's correction. *p-value < 0.05, ** p-value < 0.01, ***p-value < 0.001, ****p-value < 0.0001.

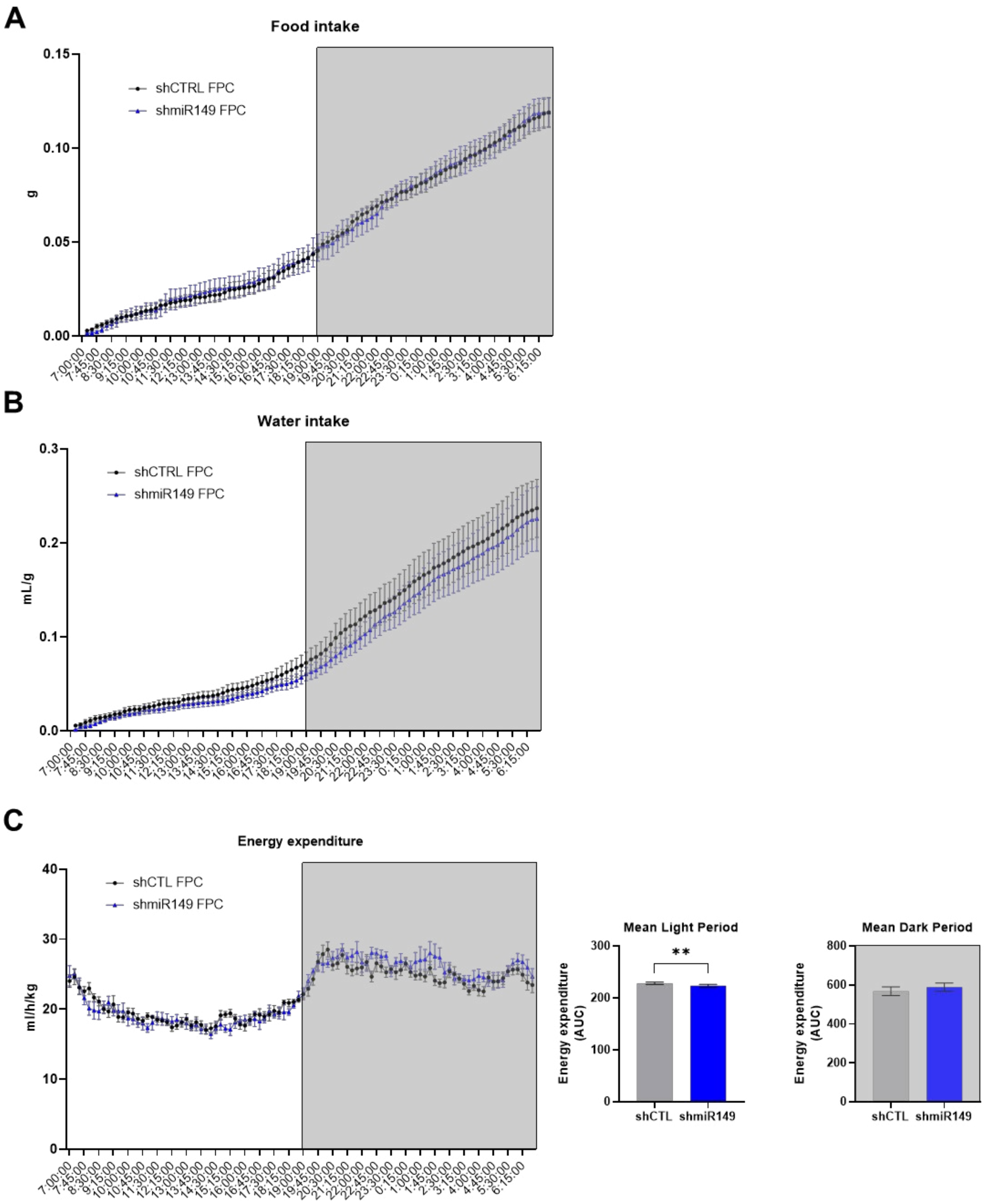


**Fig. S11 – Inhibition of miR-149-5p in mice fed fructose-palmitate-cholesterol (FPC) diet does not impact energy expenditure, water and food intake.**

**(A)** Food, **(B)** water intake and **(C)** energy expenditure rate and corresponding AUCs (light versus dark period, right panels) measured over 7 days (mean of the 7 days) in shCTL (n=7) and shmiR149 (n=6) mice after 23 weeks of FPC diet. Data is represented as mean ± SD. Unpaired t-test with Welch's correction. *p-value < 0.05, ** p-value < 0.01, ***p-value < 0.001, ****p-value < 0.0001.

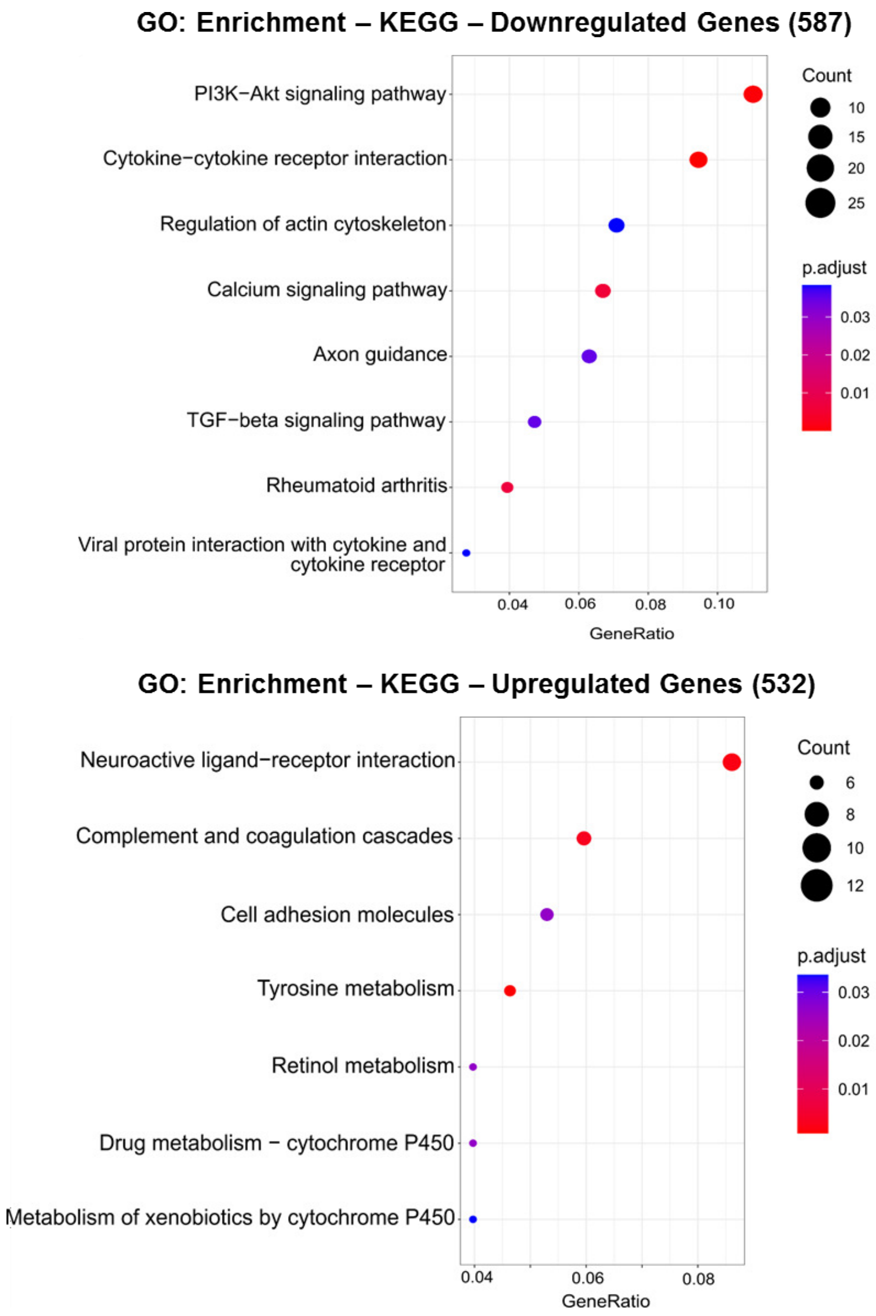


**Fig. S12 – Overexpression of miR-149-5p in the hepatic cell line Huh7 leads to deregulation of genes involved in metabolic and inflammatory pathways.**
Gene ontology enrichment analysis of KEGG pathways with **(A)** downregulated and **(B)** upregulated genes identified in transcriptomic analyses of polysomal fractions from Huh7 cells transfected with synthetic oligonucleotides mimicking miR-149-5p or with scrambled mimics. Significantly deregulated genes were identified using the following thresholds – fold-change to control mimic = |1.5| and false discovery rate (FDR) < 0.05.

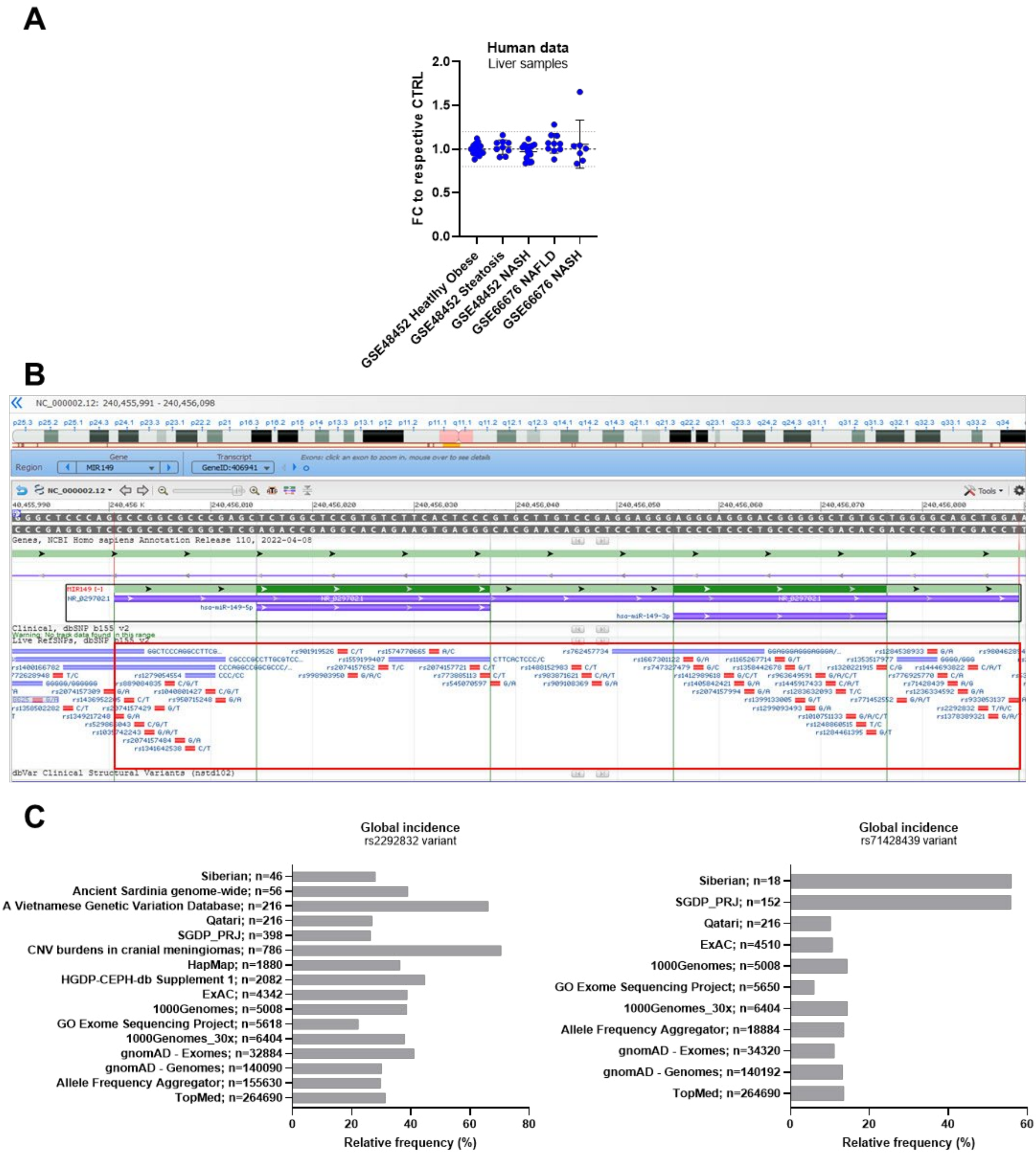


**Fig. S13 – miR-149 expression in patients diagnosed with hepatic diseases and human polymorphisms identified in the genomic region encoding miR-149.**
**(A)** Gene expression omnibus (GEO) datasets analyses of miR-149-5p expression (FC to CTRL) in hepatic tissues of healthy obese, steatotic or MASH patients. Data is represented as mean ± SD fold change (FC) to CTRL group. **(B)** Forty-five single nucleotide polymorphisms (SNP) annotated in miR-149 region (source: dbSNP-NCBI, accessed on 03/04/2024). **(C)** Global incidence of two of them (rs2292832 and rs71428439) with reported effects on miR-149 maturation and linked with susceptibility to develop several pathologies, including metabolic (dysfunction) – associated steatotic liver disease (MASLD, previously known as non-alcoholic fatty liver disease – NAFLD) and hepatocellular carcinoma (Table S2). Unpaired t-test with Welch's correction. *p-value < 0.05, ** p-value < 0.01, ***p-value < 0.001, ****p-value < 0.0001.

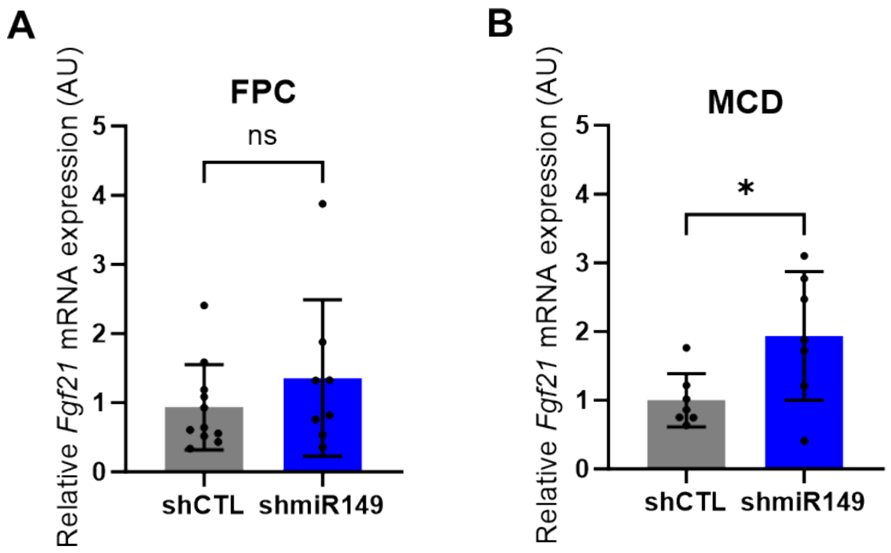


**Fig. S14 – In vivo downregulation of miR-149-5p specifically in hepatocytes modulates *Fgf21* hepatic expression upon fructose/palmitate/cholesterol (FPC) diet or methionine/choline-deficient (MCD) diet.** Relative *Fgf21* mRNA expression in livers of **(A)** fructose/palmitate/cholesterol (FPC) diet-fed mice or of **(B)** methionine/choline-deficient (MCD) diet-fed after injection with hepatotropic adeno-associated virus (AAV8) harboring vectors encoding for scrambled shRNAs (shCTL, n=7-12) or for shRNAs specific for miR-149-5p (shmiR149, n=7-11). Data is represented as mean ± SD fold change (FC) to CTRL group. Unpaired t-test with Welch's correction. *p-value < 0.05, ** p-value < 0.01, ***p-value < 0.001, ****p-value < 0.0001.

## Supplementary tables

**Supplementary tables**

| Table S1 – Literature screening performed using the 237 potential targets of miR-149 identified with translatomic analyses. Only studies related with MASLD/MASH or metabolic deregulations were considered. | | | | | | | |
|---|---|---|---|---|---|---|---|
| **Gene Name (Mouse)** | **Human Ortholog** | **Expression (FC to CTRL Mimic)** | **Adj p-value (FDR)** | **Investigated in liver disease7 Yes/No** | **Role in Steatosis/Fibrosis/Liver inflammation (NAFLD)7** | **PMID** | **▲ or ▼/ promotes which process)** |
| Cx3cl1 | CX3CL1 | -2.762777121 | 7.34E-06 | Yes | Overexpression of Cx3cl1 attenuated glucose intolerance, hepatic IR and fibrosis (but not steatosis) after atherogenic diet for 16 weeks | 35914622 | ▲/Restrains glucose intolerance, hepatic IR and fibrosis |
| Pdgfrb | PDGFRB | -4.257690608 | 7.33E-06 | Yes | Used as a marker of liver fibrosis; Linked with stellate cell activation, hepatic collagen production and hepatic fibrosis. Regulated by PTEN. Increased in MCD and HFD fed mice with positive correlation with fibrosis scores. | 31036530; 35834952, 30901579 | ▲ / promotes hepatic fibrosis |
| Vcan | VCAN | -11.12010812 | 7.53E-06 | yes, but no mechanistic in vivo | Identified as potential marker of hepatitis-B related fibrosis and immune infiltration (increased with fibrosis). Identified also as a transcriptomic signature of MASH progression | 36275632; 33837926; 30046009 | ▲ / promotes hepatic fibrosis and immune cell infiltration |
| Lefty1 | LEFTY1 | -2.92961806 | 0.007379222 | yes | Delivery of Lefty1 mRNA through vesicles in HSC reduced fibrosis in rats injected with CCL4 | 36382306 | ▲/attenuates fibrosis |
| Zfand3 | ZFAND3 | -2.073016339 | 0.000111107 | Yes | ZFAND3 overexpression in the mouse liver improves glucose tolerance and hepatic insulin resistance. | 33782927 | ▲/attenuates hepatic IR |
| Batf | BATF | -2.345494153 | 0.017892142 | Yes | AAV-induced overexpression of BATF alleviated HFD-induced steatosis | 37712938 | ▲/attenuates hepatic steatosis |
| Fgfr1 | FGFR1 | -2.028466904 | 2.61E-05 | Yes | Same litterature than FGF21. Nothing with a genetic alteration of FGFR1. | 34654875 | ▲/attenuates hepatic steatosis, inflammation hyperglycemia and IR |
| Gdf15 | GDF15 | -2.72025072 | 0.001164787 | Yes | Gene signature strongly associated with MASH. Treatment with recombinant Gdf15 ablated lipid accumulation in primary hepatocytes . Gdf15 Associated with weight loss; GDF15 correlates positively with better oxidative stress status. | 33268509; 31857195; 35504134 | ▲/attenuates lipid accumulation |
| Fgf21 | FGF21 | -3.801489604 | 0.00563014 | Yes | Fgf21 treatment reduced hepatic steatosis, lobular inflammation, hepatocyte ballooning and fibrosis in mice under highfat-highcholesterol diet for 23w. It also modulated immune cell population (decreased eosinophils, neutrophils and B cells), prevented activation of Kupffer cells and facilitated cholesterol removal. It also reduced MCD-induced hepatic and intestine injury - including hepatic steatosis and fibrosis. It also induced alterations in gut microbiota, short FA and BA metabolism | 36648330, 36586452 | ▲/attenuation of hepatic steatosis, inflammation/injury and, immune infiltration |
| Tnip2 | TNIP2 | -2.487236906 | 1.46E-05 | yes | Overexpression of Tnip2 led to inhibition of NFκB and delayed liver regeneration after partial hepatectomy. | 16480954 | ▲/Inhibits NFκB (potential impact on chronic inflammation) |
| Fmnl1 | FMNL1 | -2.989480911 | 0.00536369 | Yes | Transcriptome data provided a novel landscape of the Monocyte-Macrophage-Dendritic Cell (MMD) system that is involved in advanced MASH disease status. Fmnl1 expression in the MMD system was associated with the progression of MASH fibrosis. | 36911682 | ▲/marker of fibrosis |
| Shh | SHH | -2.007896799 | 0.001719701 | Yes | Marker of hepatocytes ballooning by IHC / MASH | 35224757; 30744560 | ▲/marker of hepatic injury (ballooning) |
| Casr | CASR | -4.014213997 | 0.027757892 | Yes (Liver not NAFLD) | In Wistar rats, the upregulation of Casr expression is involved in the occurrence of diabetic liver injury and fibrosis. | 32476365 | ▲/marker of hepatic injury and fibrosis |
| Btg2 | BTG2 | -2.366617691 | 0.000152796 | Yes | Overexpression of Btg2 in wild-type mice promoted the expression of gluconeogenic enzymes and hyperglycemia, while its downregulation in db/db mice attenuated glucose intolerance. | 37488285 | ▲/promotes gluconeogenesis and hyperglycemia |
| C7 | C7 | -3.397567566 | 0.000128303 | Yes | Functional characterization not available, but plasma levels of C7 correlated positively with fibrosis extent. | 31860081 | ▲/promotes hepatic fibrosis |
| Ncf2 | NCF2 | -3.115587612 | 0.000145192 | yes, but indirect mechanisms | HSC-specific SRF KO mice have attenuation of hepatic fibrosis induced by MCD (due to inhibition of NCF1/NCF2 transcription); NCF2 expression is increased in the livers of WD-induced MASH in female Ldlr KO mice | 31442911; 31042760 | ▲/promotes hepatic fibrosis |
| Kif5a | KIF5A | -4.13575848 | 0.00031402 | Yes | Increased expression linked with a specific subtype of HCC (increased CD8+ infiltration and anti-tumor responses) | 36686769 | ▲/Promotes immune cell infiltration (inflammation) |
| Pde4d | PDE4D | -2.498215842 | 1.39E-05 | Yes | Overexpression of PDE4D with AAV8 under chow diet impaired insulin signalling and promoted hepatic steatosis (due to increased expression of Cd36 - lipid uptake promoted). Treatment with Roflumilast (PDE4 inhibitor) under HFD conditions improved hepatic steatosis induced by the diet. Similarly, liver-specific PDE4D KO under HFD gained less weight, had attenuation of hepatic steatosis and other metabolic deregulations linked with the diet (i.e. hyperglycemia, insulin resistance) | 34826603; 37072403 | ▲/Promotes Steatosis, hyperglycemia and IR |
| Rnaseh1 | RNASEH1 | -3.410011433 | 2.08E-05 | Yes | RNaseH1 KO mice presented mitochondrial dysfunction and presented hepatic injury and fibrosis. | 27131367 | ▼ / promoted hepatic injury and fibrosis |

| | | | | | | | |
|---|---|---|---|---|---|---|---|
| Phlpp2 | PHLPP2 | -3.446898415 | 1.02E-05 | yes | Liver specific Phlpp2 KO mice showed increased liver weight and hepatic steatosis but normal glucose metabolism under chow diet. Overexpression decreased TG accumulation under HFD diet due to decreased lipogenesis | 28859855, 26743335 | ▼ / promoted hepatic steatosis |
| Cidec | CIDEC | -2.266642423 | 0.004992412 | Yes | Hepatic expression levels of Cidec/Fsp27 increases with MASLD progression to MASH. Adipose tissue specific Cidec (Fsp27) KO were protected against BW gain but had increased hepatic steatosis, dyslipidemia and IR in HFD fed mice. Silencing of Fsp27 with synthetic oligos systemically did not affect diet-induced hepatic steatosis/ steatohepatitis, but improved glucose tolerance and IR. Hepatic specific deletion of Fsp27 restrained hepatic steatosis and injury, inflamation and fibrosis induced by chronic ethanol feeding. In another study, KD led to decreased hepatic steatosis induced by fasting by promoting FAoxidaition. | 25477509; 27884961; 28874443; 31097771; 26099526; | ▼ in AT/promotes hepatic steatosis, glucose intolerance, IR and inflammation; ▼ in Liver/restrains hepatic steatosis, inflammation and fibrosis |
| Crtc2 | CRTC2 | -2.632009038 | 9.59E-06 | Yes | Total CRTC2 KO total promoted HFD induced steatosis due to regulation of SREBP1c expression. Hepatocyte specific CRTC2 KO attenuated hepatic steatosis and macrophage infiltration | 26147081; 34838715 | ▼ in hepatocytes/ attenuates hepatic steatosis and macrophage infiltration |
| S1pr2 | S1PR2 | -3.753428249 | 6.19E-06 | yes | S1pr2 KO specifically in liver sinusoidal endothelial cells attenuated hepatic injury and fibrosis induced by CCl4 injection, while overexpression led to the inverse phenotype due to activation of YAP signalling and increased expression of TGF-³. Downregulation of S1pr2 in hepatocytes also attenuated hepatic injury and fibrosis induced by a DDC diet. Inhibition of S1pr2 with pharmacological agents attenuates fibrosis and HCC development in a model of congestion-induced fibrosis model and NLRP3 inflammasome priming/neutrophil infiltration/cytokine expression in a bile duct ligation model of hepatic injury, mice fed a HFD/MCD or CCl4 injection. Conversely, S1PR2 blockage increased diacylglycerol and TG levels in the liver of Mc4r-KO mice fed a western diet for 20 weeks | 37039817; 36800698; 34855990; 33388881; 33197723; 32768187; 32695095 | ▼/ attenuates hepatic inflammation, fibrosis and neutrophil infiltration but promotes steatosis (DG/TG accumulation) |
| Cmtm3 | CMTM3 | -3.4186108 | 7.53E-06 | yes, in HCC | Cmtm3 KO mice had attenuated liver damage induced by DEN injection (decreased ALT/AST and hepatic cytokine expression), | 36284038 | ▼/ attenuates hepatic inflammation/injury |
| Cxcl12 | CXCL12 | -9.63069051 | 6.19E-07 | yes, but no mechanistic in vivo | Inhibitor of CXCR4 (receptor of Cxcl12) decreased immune cell infiltration (CD4+ T cells) in the livers of db/db mice, but it promoted hepatic inflammation and fibrosis after CCl4-injection due to increased neutrophiles.. | 25074471; 25163538 | ▼/ promotes hepatic inflammation, fibrosis and neutrophil infiltration |
| Csf1 | CSF1 | -3.968651991 | 7.33E-06 | yes | CSF1 circulating levels associated with a decline in MASLD severity. CSF1 inhibition with antibodies in a thioacetamide (TAA)-induced liver injury model promoted hepatic inflammation, but when TAA was removed Csf1-Ab promoted fibrosis resolution. In neonatal rats, Csf1-Ab promoted reversible hepatic steatosis and hepatic macrophage accumulation. Similar effects regarding macrophage accumulation in mice after partial hepatectomy and acetaminophen intoxication. | 34006921; 35169835; 29351395; 26344055 | ▼/ promotes hepatic inflammation/fibrosis upon injury, but also resolution when damaging agent is removed |
| Fhl2 | FHL2 | -2.056473863 | 0.004105145 | Yes | Fhl2 KO mice are more sensitive to fibrosis, liver injury and inflammation induced by bile duct ligation and CCL4 injection . | 31963815; 28223370; 23311569 | ▼/aggravates hepatic injury, inflammation and fibrosis |
| Ptpn14 | PTPN14 | -2.480957533 | 4.97E-05 | yes | Ptpn14 KO mice are less sensitive to acute liver injury (by interfering with SOCS7-dependent cytokine expression) | 32978373 | ▼/attenuated hepatic injury and inflammation |
| Dyrk1b | DYRK1B | -2.320668358 | 6.38E-05 | Yes | DYRK1B mutation associated with MASLD in humans and it is increased in MASH patients. KD of DYRK1B via AAV-shRNA reduced steatosis and fibrosis induced by high fat- high fructose diet due to increased lipogenesis and fatty acid uptake | 34855620 | ▼/attenuated hepatic steatosis and fibrosis |
| Loxl2 | LOXL2 | -2.593775735 | 0.000530223 | Yes | Injection of Loxl2/Loxl3 inhibitors ammeliorated hepatic fibrosis induced by CCL4 injection and sterptozotocin/HFD diet; Loxl2 expression is increased in experimental models of MASLD (MCD,MCD+HFD) and it is also increased in diabetic patients with hepatic fibrosis | 30536539; 28468951 | ▼/attenuates fibrosis |
| Satb1 | SATB1 | -2.046876779 | 0.002052137 | yes | KD of Satb1 in hepatocytes attenuated TAA- and CCl4-induced hepatic fibrosis in rats and in mice. | 25896016; 27883059 | ▼/attenuates fibrosis |

| | | | | | | | |
|---|---|---|---|---|---|---|---|
| Tgfb1i1 | TGFB1I1 | -2.37163997 | 0.00611023 | Yes | Tgfb1i1 (alias Hic-5) downregulation attenuated mouse liver fibrosis and HSC activation induced by bile duct ligation and CCl4 injection by inhibiting TGFB-Smad2 signalling and collagen/a-sma expression | 26334580 | ▼/attenuates fibrosis |
| Sema3c | SEMA3C | -2.127413743 | 0.007366958 | yes | Increased in human MASH and in MASH mouse models. Specific deletion of Sema3c in myofibroblasts and in HSCs attenuated fibrosis induced by CCl4 injection. | 37055018; 36551769 | ▼/attenuates fibrosis |
| Cftr | CFTR | -2.096992615 | 0.003642721 | Yes | Pharmacological inhibiton of CFTR --> decrease fibrosis induced by HFMCD | 36754244 | ▼/attenuates fibrosis |
| Mical2 | MICAL2 | -2.205685168 | 8.42E-05 | Yes | Mical2 silencing alleviated hepatic fibrosis and injury induced by CCl4 injection. | 32659284 | ▼/attenuates hepatic injury and fibrosis |
| Shmt2 | SHMT2 | -2.394399467 | 0.002234562 | Yes | In mice injected with CCl4 and fed WD, HSC overexpressed Shmt2.<br>KD of Shmt2 in these mice decreased fibrosis and hepatic injury (ALT/AST levels) after two weeks of treatment. | PMID: 37307917 | ▼/attenuates hepatic injury and fibrosis |
| Atf3 | ATF3 | -2.562711026 | 0.001109398 | Yes | It was found that Atf3 switches cell death from apoptosis to necroptosis in hepatic steatosis. In severe hepatic steatosis, after partial hepatectomy, hepatic ATF3-deficient mice displayed decreased RIPK3 expression and necroptosis. Similarly, another study knockdown of ATF3 attenuated glucose intolerance, IR and inflammation in Zucked diabetic rats | 36690638; 28365312 | ▼/attenuates hepatic injury, glucose intolerance, IR and inflammation |
| Klhl3 | KLHL3 | -2.542066047 | 0.003069474 | yes | Klhl3 KO mice showed attenuated BW gain and IR, hepatic steatosis and glucose intolerance after aging or HFD due to increased exergy expenditure and O2 consumption. Upon MCD diet, the total KO showed attenuated hepatic steatosis and injury linked with decreased macrophage infiltration and fibrosis. Similar observations with KD in the hepatocytes specifically. | 36028759 | ▼/attenuates hepatic injury, steatosis, inflammation, fibrosis and glucose intolerance |
| Il11 | IL11 | -4.51222769 | 0.002727796 | yes | Hepatocyte specific deletion of Il11 attenuates hepatocyte death, hepatic steatosis, fibrosis and inflammation and reduces plasma levels of glucose, cholesterol and TG levels induced by High-fat MCD diet for 4 weeks or a WD for 16 weeks. Blockage of IL-11 attenuated hepatic steatosis, fibrosis, hepatocyte death, inflammation and hyperglycemia following HFMCD diet for 6/10 weeks and on db/db mice fed MCD diet. | 33397952; 31078624 | ▼/attenuates hepatic injury, steatosis, inflammation, fibrosis and hyperglycemia |
| Pld1 | PLD1 | -2.181075369 | 8.67E-05 | Yes | Hepatocyte-specific Pld1 deficiency ameliorates lipid accumulation induced by HFD by inhibiting the PPAR´/CD36 pathway. PLD1 may be a new target for the treatment of MASLD. Similarly, PLD1 was found to play an important role in hepatic steatosis via the regulation of autophagy in Pld1−/− mice. | 37138676; 27976696 | ▼/attenuates hepatic steatosis |
| Tnfrsf9 | TNFRSF9 | -3.357319337 | 0.001410558 | Yes | HFD-induced glucose intolerance/insulin resistance and hepatic steatosis and inflammation were attenuated in the 4-1BB-deficient mice. | 21998397 | ▼/attenuates hepatic steatosis and inflammation, glucose intolerance and IR |
| Tlr2 | TLR2 | -2.476088408 | 0.012677243 | Yes | Blockage of TLR2 in rats under HFD attenuated hepatic steatosis, fasting hyperglycemia, inflammation and fibrosis. But Tlr2 KO mice were more sensitive to MCD-induced hepatic steatohepatitis and fibrosis. | 32151955; 20509914 | ▼/attenuates hepatic steatosis, fasting hyperglycemia, inflammation and fibrosis under HFD but aggravates steatohepatitis and fibrosis under MCD |
| Stk38 | STK38 | -2.093259298 | 8.01E-05 | Yes | HFD induces the expression of hepatic STK38 promotes systemic inflammation and IR. Overexpression of STK38 in mouse liver leads to hepatic inflammation and steatosis, IR and hypertriglyceridemia in mice fed on a regular chow diet.KO of STK38 in HFD-fed mice attenuates inflammation, improves hepatic insulin sensitivity, and decreases hepatic fat accumulation. STK38 binds to Tank-Binding protein Kinase 1 and which promotes NF-κ³ nuclear translocation (expression of proinflammatory cytokines). Intrahepatic lipid accumulation due to enhanced de novo lipogenesis via reducing the AMPK-ACC signaling axis. | 37028764 | ▼/attenuates inflammation, IR and steatosis |
| Bach2 | BACH2 | -2.237880831 | 0.000538247 | Yes | Bach2 expression was increased in mice with hepatic steatosis. PCR, oil red O staining and triglyceride detection revealed that the silencing of BACH2 reduced lipid accumulation in hepatoblastoma cell line. | 37362825 | ▼/attenuates lipid accumulation |

| | | | | | | | |
|---|---|---|---|---|---|---|---|
| Spp1 | SPP1 | -2.666216212 | 0.000392201 | Yes | Increased in MASLD in humans. Spp1 impact in MASLD is inconclusive. Spp1 KO mice fed a HFD had decreased hepatic steatosis (due to decreased lipogenesis), glucose intolerance, insulin resistance and lobular inflammation/hepatocyte ballooning. However, Spp1KO mice treated with streptozotocin and challenged with HFD for 4 weeks had increased steatosis and hepatic injury (ALT levels increased). DNL was decreased but FA uptake as well as fibrosis was promoted in this context. In other studies, it was shown that Spp1KO mice fed MCD or HFD with 2% cholesterol had no effect regarding steatosis but they were protected from fibrosis. In WT mice under standard chow diet, treatment with recombinant osteopontin led to increase in liver PC/PE/Cholesterol and TG. | 32730345, 21562757, 32281248, 32986864, 15044174, 31852298, 28754826 | ▼/Inconclusive. Depending on the stimuli it can promote/attenuate hepatic steatosis, inflammation/injury, fibrosis, glucose intolerance, IR |
| Inhbe | INHBE | -2.857728121 | 0.014191069 | Yes | INHBE positively correlated with insulin resistance and body mass index in humans.Additionally, Inhbe gene expression increased in the livers of db/db mice. Downregulation of INHBE suppressed body weight gain due to decreased fat rather than lean mass. It also decreased the respiratory quotient and increased plasma total ketone bodies, suggesting enhanced whole-body fat utilization. | 29596463 | ▼/increases fat utilization and decreases fat mass |
| Sema3d | SEMA3D | -2.249738309 | 0.000153715 | Yes | Sema3d is downregulated in HCC, it's downregulation led to promoted tumor growth/EMT and metastasis in xenograft HCC models due to inactivation of Pi3k/Akt by FLNA (Sema3d interactor) | 35957887 | ▼/Inhibition of PI3K/AKT |
| Cblb | CBLB | -2.019852724 | 0.00026572 | yes, but no mechanistic in vivo | Aged total Cblb KO mice showed increased glucose intolerance, IR and hepatic steatosis. Dendritic cells-specific KO mice showed signs of hepatic fibrosis, cirrhosis and increased expression of cytokine expression upon aging. | 17601987; 35354799; 34630435 | ▼/promotes glucose intolerance, hepatic steatosis, inflammation and fibrosis |
| Dusp16 | DUSP16 | -2.001454627 | 0.000251049 | Yes | DUSP16 suppression accelerates dyslipidemia and inflammation in palmitate-treated hepatocytes. DUSP16 over-expression has the inverse phenotype, and DUSP16 knockout promotes glucose intolerance/IR, hepatic steatosis and inflammation in HFD-fed mice due to promoted lipogenesis and FA uptake and decreased oxidation. | 31982140 | ▼/promotes glucose intolerance, IR, hepatic steatosis and inflammation |
| Cox10 | COX10 | -2.098539777 | 7.72E-05 | Yes | Cox10 KO mice had severe liver dysfunction, increased mitochondrial proliferation and hepatic steatosis | 17951359 | ▼/promotes hepatic steatosis |
| Ctse | CTSE | -2.260194175 | 0.001580279 | Not directly | Ctse KO mice showed defective adipose tissue development which promoted hepatic steatosis and hypercholesterolemia under HFD for 24w. | 24583126 | ▼/promotes hepatic steatosis |
| Colgalt2 | COLGALT2 | -3.267861364 | 7.83E-05 | Yes | Total Colgalt2 KO has increased lipodystrophy and promotes hepatic steatosis and steatohepatitis induced by HFD or MCD | 33865898 | ▼/promotes hepatic steatosis and inflammation |
| Ppard | PPARD | -2.136572473 | 7.83E-05 | yes | Injection of Ppar· agonist and overexpression in vivo showed ammelioration of hepatic steatosis and plasma TGs. Reconstitution of Ppar· WT mice with bone marrow from Ppar· KO mice indicated decreased activated macrophages following HFD for 22weeks, but worsen hepatic steatosis due to decreased expression of FA oxidation and OXPHOS genes. | 18024853; 18522831 | ▼/promotes hepatic steatosis but restrains macrophage activation |
| Erbb4 | ERBB4 | -3.17562217 | 0.00051304 | Not directly | ErbB4 is activated by Nrg4 which leads to inhibits de novo lipogenesis, hepatic steatosis and ammeliorates IR; ErbB4 KO mice fed medium fat diet for 24w had aggravated obesity, dyslipidemia, hepatic steatos, hyperglycemia and hyperinsulinemia + IR. | 25401691; 29944391 | ▼/promotes hepatic steatosis, glucose intolerance and IR |
| Tnfaip3 | TNFAIP3 | -2.078739395 | 0.000769763 | Yes | Liver-specific Tnfaip3 KO had promoted BW gain, hepatic steatosis, glucose intolerance, insulin resistance and inflammation after 24w of HFD due to overactivation of ASK1/p38/JNK signalling | 29227477 | ▼/promotes hepatic steatosis, glucose intolerance, IR and inflammation |
| Erbb3 | ERBB3 | -2.60601946 | 4.21E-05 | Yes | Hepatocyte specific ERBB3 KO showed attenuated fibrosis in a CCl4-induced liver injury mouse model . It's silencing in vitro agravates steatosis and inflammation/oxidative stress due to increased TG levels, IL-6 and TNF-a by decreasing PI3K/AKT signalling . | 27586651; 33541789 | ▼/promotes hepatic steatosis, inflammation/oxidative stress |
| Igfbp3 | IGFBP3 | -2.019231741 | 0.000125435 | Yes, but not in vivo | Silencing IGFBP-3 in Huh7 cells enhanced JNK and NF-κB activity and increased palmitate-induced IL-8 secretion. Under lipotoxic conditions, palmitate inhibits hepatic macrophage secretion of IGFBP-3, thus enhancing palmitate-induced IL-8 synthesis and secretion. | 22475139; 27553225 | ▼/promotes inflammation |

| | | | | | | | | |
|---|---|---|---|---|---|---|---|---|
| Rbbp4 | RBBP4 | -2.196823559 | 4.68E-05 | yes, but no mechanistic in vivo | Rbbp4 silencing inhibited fatty acid oxidation and promoted lipid accumulation in AML12 cells | 35637971 | ▼/promotes lipid accumulation |
| Cndp2 | CNDP2 | -2.970564974 | 4.60E-05 | Yes | CNDP2 KO are more prone to acetaminophed overdose-induced liver and renal injury due to potential increase of oxidative stress | 34324979 | ▼/Promotes oxidative stress (inflammation) |
| Adam28 | ADAM28 | -5.133112725 | 0.000368588 | Yes | Downregulation Adam28 in mice led to attenuation of BW gain, insulin resistance/Glucose intolerance and liver injury induced by HFD for 10weeks | 28430139 | ▼/Restrains hepatic steatosis, hypeglycemia and IR |
| Hdac9 | HDAC9 | -2.73684791 | 0.000886702 | Yes | Hdac9 KO mice are protected from BW gain, AT dysfunction and hepatic steatosis induced by HFD diet for 12 weeks. HDAC9 upregulation linked to increased hepatic gluconeogenesis induced by HCV infection due to regulation of CREB, PGC-1a, glucocorticoid receptor. | 24101673; 28733598; 26420860 | ▼/Restrains hepatic steatosis; ▲/Promotes gluconeogenesis |
| Icam1 | ICAM1 | -2.230915347 | 0.008378443 | yes | Icam KO mice challenged with ethanol had decreased graft injury upon transplantation into WT mice due to decreased leukocyte adherence and ROS production | 22778492 | ▼/restrains inflammation and immune cell infiltration |
| Gpc4 | GPC4 | -2.008893903 | 0.004017722 | yes, but no mechanistic in vivo | Linked with impaired glucose tolerance (increased expression) and T2D (decreased expression). | 25240528 | Inconclusive. |
| Hs6st2 | HS6ST2 | -3.948616021 | 2.73E-06 | No | | | |
| Kctd20 | KCTD20 | -3.570798696 | 2.73E-06 | No | | | |
| Tspan14 | TSPAN14 | -4.470437078 | 2.73E-06 | No | | | |
| Plekhb1 | PLEKHB1 | -4.120678396 | 7.33E-06 | No | | | |
| Myrf | MYRF | -3.013570954 | 7.34E-06 | No | | | |
| Elp5 | ELP5 | -2.735917437 | 9.59E-06 | No | | | |
| Map7d1 | MAP7D1 | -3.04932509 | 9.59E-06 | No | | | |
| Rgs2 | RGS2 | -2.570315811 | 9.59E-06 | No | | | |
| Tead2 | TEAD2 | -2.549061538 | 1.11E-05 | No | | | |
| Zdhhc18 | ZDHHC18 | -2.33230941 | 1.16E-05 | No | | | |
| Rnf2 | RNF2 | -2.612306594 | 1.66E-05 | No | | | |
| Thyn1 | THYN1 | -2.331387512 | 1.75E-05 | No | | | |
| Capn15 | CAPN15 | -2.284620987 | 1.78E-05 | No | | | |
| Cxadr | CXADR | -2.224655086 | 1.80E-05 | No | | | |
| Gpr37 | GPR37 | -2.760443859 | 1.80E-05 | No | | | |
| Ap5m1 | AP5M1 | -2.401777747 | 1.96E-05 | No | | | |
| Adam23 | ADAM23 | -2.417580688 | 2.04E-05 | No | | | |
| Aak1 | AAK1 | -2.617391387 | 2.16E-05 | No | | | |
| Pde3a | PDE3A | -2.492622412 | 2.48E-05 | Not directly | | | |
| Cdk17 | CDK17 | -2.425987367 | 2.52E-05 | No | | | |
| Flrt2 | FLRT2 | -3.071625083 | 2.61E-05 | No | | | |
| Mrpl17 | MRPL17 | -2.346159029 | 2.61E-05 | No | | | |
| Mxra8 | MXRA8 | -2.067606884 | 2.61E-05 | No | | | |
| Ralbp1 | RALBP1 | -2.144718397 | 2.61E-05 | No | It was shown to be involved in endocytosis of EGFR/Insulin Receptor in the liver by interacting with ARIP2 | 11882656 | |
| Fhdc1 | FHDC1 | -3.229847224 | 2.61E-05 | No | | | |
| Ranbp3l | RANBP3L | -3.600393248 | 2.67E-05 | No | | | |
| Panx1 | PANX1 | -2.68137986 | 2.73E-05 | No | | | |
| Tuba1a | TUBA1A | -3.487831395 | 2.75E-05 | No | | | |
| Ngrn | NGRN | -2.082100668 | 2.90E-05 | No | | | |
| Ttyh1 | TTYH1 | -7.475746216 | 2.98E-05 | No | | | |
| Ebp | EBP | -2.5121591 | 3.36E-05 | no | | | |
| Igdcc3 | IGDCC3 | -2.298801684 | 3.45E-05 | No | | | |
| Gpr161 | GPR161 | -2.254752536 | 3.46E-05 | No | | | |
| Glipr1 | GLIPR1 | -5.026245328 | 3.94E-05 | No | | | |
| B3galt1 | B3GALT1 | -4.23968167 | 4.09E-05 | no | | | |
| Hspb8 | HSPB8 | -3.081796277 | 4.09E-05 | No | | | |
| Lmtk2 | LMTK2 | -2.391513509 | 4.21E-05 | No | | | |
| Fam98a | FAM98A | -2.059328085 | 4.68E-05 | No | | | |
| Mex3a | MEX3A | -2.448894354 | 5.04E-05 | Yes | Nothing related to MASLD | | |
| Rere | RERE | -2.045319459 | 5.57E-05 | no | | | |
| Atp8b2 | ATP8B2 | -2.069426313 | 5.70E-05 | Yes | Nothing related to MASLD | | |
| Sema4g | SEMA4G | -2.00272875 | 5.92E-05 | No | | | |
| Arhgef18 | ARHGEF18 | -2.491424675 | 6.01E-05 | No | | | |
| Wsb1 | WSB1 | -2.075341478 | 6.42E-05 | Yes | Nothing related to MASLD | | |
| Rab3b | RAB3B | -2.408227029 | 7.21E-05 | Yes | Identified as potential markers of oval cells (proilferating on the periportal region) in liver injury induced by CCL4 injection. No function described | 16781709 | |
| Prl3c1 | PRL | -5.319486765 | 7.72E-05 | No | | | |
| Cnnm1 | CNNM1 | -2.179426537 | 7.83E-05 | No | | | |
| Dlg5 | DLG5 | -2.260874936 | 7.83E-05 | No | | | |
| Tsc22d3 | TSC22D3 | -3.432804118 | 7.83E-05 | Yes | Nothing related to MASLD | | |
| Cnn1 | CNN1 | -3.651308593 | 8.00E-05 | no | | | |
| Cdh6 | CDH6 | -3.658050741 | 8.24E-05 | No | | | |
| Jade3 | JADE3 | -2.30523696 | 8.40E-05 | No | | | |
| Itga2 | ITGA2 | -4.256480782 | 8.42E-05 | No | | | |
| Rftn2 | RFTN2 | -3.902104345 | 8.59E-05 | No | | | |
| Gp2 | GP2 | -4.172622075 | 8.61E-05 | No | | | |
| Bmf | BMF | -2.060841067 | 8.70E-05 | No | | | |
| Nexn | NEXN | -3.179458096 | 9.95E-05 | No | | | |
| Scrn1 | SCRN1 | -2.19565101 | 9.95E-05 | No | | | |

| | | | | | | | |
|---|---|---|---|---|---|---|---|
| Rnd1 | RND1 | -2.665841368 | 0.000100186 | Yes | Nothing related to MASLD | | |
| Osbp2 | OSBP2 | -3.18225983 | 0.000101773 | No | | | |
| Dido1 | DIDO1 | -2.086626744 | 0.000105134 | No | | | |
| Slc37a1 | SLC37A1 | -2.681815781 | 0.000107186 | No | | | |
| Cdr2l | CDR2L | -2.207369067 | 0.000108352 | No | | | |
| Rassf10 | RASSF10 | -6.290982745 | 0.000113662 | Yes, but not in vivo | | | |
| Foxp4 | FOXP4 | -2.022171434 | 0.00012542 | Not in vivo | | | |
| Rtn4rl1 | RTN4RL1 | -2.559756647 | 0.000139092 | no | | | |
| Rnf122 | RNF122 | -2.188737062 | 0.000140611 | No | | | |
| Ift81 | IFT81 | -2.173449113 | 0.000149415 | No | | | |
| Scube3 | SCUBE3 | -5.940129265 | 0.000152796 | No | | | |
| Mink1 | MINK1 | -2.063249122 | 0.000154002 | no | | | |
| F2rl2 | F2RL2 | -4.503198881 | 0.000167976 | No | | | |
| Vsig1 | VSIG1 | -16.48842458 | 0.000170858 | No | | | |
| Hepacam2 | HEPACAM2 | -2.383656316 | 0.00017237 | No | | | |
| Dennd2a | DENND2A | -2.731666058 | 0.000174548 | Yes | Nothing related to MASLD | | |
| Adamts12 | ADAMTS12 | -3.464673585 | 0.000175227 | No | | | |
| Mmp11 | MMP11 | -2.115276424 | 0.00017956 | Yes | Nothing related to MASLD | | |
| Prtg | PRTG | -2.136180642 | 0.00019379 | No | | | |
| Asb2 | ASB2 | -4.575918532 | 0.000217317 | No | | | |
| Rab30 | RAB30 | -2.425223794 | 0.000250151 | No | Investigation of the hepatocyte PPAR³ as a central regulator of gene expression during starvation in Ppar³hep−/− mice. Novel PPAR³-sensitive genes, including Rab30, were identified. | 28774777 | |
| Hcn4 | HCN4 | -2.978505426 | 0.000282511 | No | | | |
| Ano3 | ANO3 | -2.262677592 | 0.000283771 | No | | | |
| Brinp3 | BRINP3 | -2.023167663 | 0.000283771 | No | | | |
| Galnt16 | GALNT16 | -2.11201783 | 0.000340639 | Yes | Nothing related to MASLD | | |
| Sdc3 | SDC3 | -2.032640063 | 0.000346589 | Yes | Nothing related to MASLD | | |
| Myo7a | MYO7A | -2.827044434 | 0.000348397 | No | | | |
| Svil | SVIL | -2.017056422 | 0.000370508 | Yes | Nothing related to MASLD | | |
| Nostrin | NOSTRIN | -2.783890067 | 0.000377466 | No | | | |
| Dpysl5 | DPYSL5 | -2.411555538 | 0.000382746 | no | | | |
| Rcsd1 | RCSD1 | -10.25076473 | 0.000421213 | No | | | |
| Slc7a1 | SLC7A1 | -2.285191524 | 0.000477855 | No | | | |
| Spsb4 | SPSB4 | -2.411821351 | 0.000517391 | No | | | |
| Emp3 | EMP3 | -2.131507992 | 0.000524806 | Yes | Nothing related to MASLD | | |
| Specc1 | SPECC1 | -2.019666796 | 0.000528435 | No | | | |
| Brinp2 | BRINP2 | -4.121775372 | 0.000530223 | No | | | |
| Pde5a | PDE5A | -2.267949365 | 0.000555725 | No | | | |
| B4galnt3 | B4GALNT3 | -2.415823124 | 0.000557621 | No | | | |
| Tceal6 | TCEAL3 | -3.770894484 | 0.000569501 | No | | | |
| Bbc3 | BBC3 | -2.990696653 | 0.000621395 | Yes | Nothing related to MASLD | | |
| Tacc2 | TACC2 | -2.384695394 | 0.000699557 | Yes | Nothing related to MASLD | | |
| Kcnj16 | KCNJ16 | -2.986941414 | 0.000740193 | No | | | |
| Ank3 | ANK3 | -2.031503902 | 0.000769763 | Yes | Nothing related to MASLD | | |
| Dnajc18 | DNAJC18 | -2.166756463 | 0.000840293 | No | | | |
| Cgnl1 | CGNL1 | -2.149221203 | 0.000853627 | No | | | |
| Rab27b | RAB27B | -2.578455871 | 0.000895995 | No | | | |
| Map1a | MAP1A | -3.073838446 | 0.000909111 | No | | | |
| Nptxr | NPTXR | -2.304663398 | 0.000923771 | No | | | |
| Stox2 | STOX2 | -3.92072954 | 0.001013121 | No | | | |
| Myof | MYOF | -2.005326485 | 0.001067256 | No | | | |
| Gbp2b | GBP2 | -4.510891331 | 0.001085303 | Yes | Nothing related to MASLD | | |
| Cpa4 | CPA4 | -2.867942514 | 0.001299648 | Not in vivo | | | |
| Antxr2 | ANTXR2 | -2.181123712 | 0.001304411 | No | | | |
| Lrrn3 | LRRN3 | -6.102535465 | 0.001564151 | No | | | |
| Vil1 | VIL1 | -2.245690458 | 0.001600075 | No | | | |
| Etv1 | ETV1 | -2.034243104 | 0.001601538 | Yes | Nothing related to MASLD | | |
| Ptges3l | PTGES3L | -2.299962138 | 0.001625432 | No | | | |
| Muc6 | MUC6 | -5.789257104 | 0.001708099 | Yes | Nothing related to MASLD | | |
| Capn8 | CAPN8 | -5.883852037 | 0.001820243 | No | | | |
| Pou2af1 | POU2AF1 | -2.124602303 | 0.001978897 | No | | | |
| Plb1 | PLB1 | -4.101627122 | 0.002056047 | No | | | |
| Styk1 | STYK1 | -2.199580183 | 0.002167025 | No | Specific marker of NK cells and NK1.1 cells (like liver type 1 innate lymphoid cells) | 30690705 | |
| Gprc5a | GPRC5A | -2.761808847 | 0.002565399 | Yes | Nothing related to MASLD | | |
| Dgkg | DGKG | -2.098495135 | 0.002806547 | yes, but no mechanistic in vivo | Missense in DGKG linked with hepatic fibrinogen disease in cattle | 37681469 | |
| Hey2 | HEY2 | -2.171307158 | 0.00281202 | No | | | |
| Fibin | FIBIN | -2.352046492 | 0.002823929 | No | | | |
| Mboat4 | MBOAT4 | -2.880122602 | 0.002831593 | No | | | |
| Susd5 | SUSD5 | -4.828788396 | 0.002973325 | No | | | |
| Arhgef2 | ARHGEF2 | -2.058949087 | 0.002995813 | No | | | |
| Dpysl2 | DPYSL2 | -2.263930899 | 0.002995813 | Yes | Nothing related to MASLD | | |
| Krt15 | KRT15 | -4.315569652 | 0.003082229 | Yes | Nothing related to MASLD | | |
| Rassf6 | RASSF6 | -2.588169189 | 0.003482925 | no | | | |
| Wfikkn1 | WFIKKN1 | -2.221117687 | 0.00349677 | No | | | |
| Btbd9 | BTBD9 | -2.073276609 | 0.003693959 | No | | | |
| Cdh16 | CDH16 | -5.497554657 | 0.00391567 | No | | | |
| Rab37 | RAB37 | -2.031687521 | 0.003994348 | No | | | |
| Dchs1 | DCHS1 | -2.01837919 | 0.00399677 | No | | | |

| | | | | | | | |
|---|---|---|---|---|---|---|---|
| S100a3 | S100A3 | -4.917760738 | 0.004142494 | No | | | |
| Otogl | OTOGL | -2.625802408 | 0.004418841 | No | | | |
| Enam | ENAM | -2.292468599 | 0.004854335 | No | | | |
| Camk2a | CAMK2A | -4.663686164 | 0.005218051 | No | | | |
| Mgat3 | MGAT3 | -4.399752935 | 0.005358924 | Yes | Nothing related to MASLD | | |
| Dll4 | DLL4 | -2.22407864 | 0.005360889 | Yes | Myeloid DLL4 Does Not Contribute to the Pathogenesis of Non-Alcoholic Steatohepatitis in Ldlr-/- Mice | 2789869 | |
| Crispld1 | CRISPLD1 | -2.036710374 | 0.005539268 | No | | | |
| Fut1 | FUT1 | -3.130940075 | 0.005902644 | Yes | Nothing related to MASLD | | |
| C6 | C6 | -2.203409329 | 0.006105007 | No | | | |
| Plch1 | PLCH1 | -2.105365656 | 0.006164872 | No | | | |
| Dusp13 | DUSP13 | -3.661537322 | 0.007553091 | No | | | |
| Camkv | CAMKV | -2.245136356 | 0.008646565 | No | | | |
| P2rx6 | P2RX6 | -2.796269858 | 0.008676974 | no | | | |
| Ina | INA | -2.290729239 | 0.008893037 | No | | | |
| Pcdhgb6 | PCDHGB6 | -3.180381561 | 0.009065125 | No | | | |
| Lrp1b | LRP1B | -2.227509788 | 0.010096001 | Yes, but not in vivo | Nothing related to MASLD | | |
| Paqr8 | PAQR8 | -4.008813239 | 0.010132316 | No | | | |
| Arhgap31 | ARHGAP31 | -3.794313033 | 0.010833389 | No | | | |
| Tbc1d32 | TBC1D32 | -2.009129395 | 0.01096634 | No | | | |
| Cga | CGA | -2.567377945 | 0.011237754 | No | | | |
| Filip1 | FILIP1 | -3.43847481 | 0.011832999 | no | | | |
| Dnah11 | DNAH11 | -2.724339649 | 0.01414795 | no | | | |
| Ank1 | ANK1 | -3.352095178 | 0.019125194 | No | | | |
| Birc3 | BIRC3 | -5.965854685 | 0.020570248 | Yes | They found significant overexpression of Birc3 in alcoholic steatohepatitis and steatosis but not in MASH but no in vivo results or mechanistic explanation. | 29307797 | |
| Tmem154 | TMEM154 | -2.235002544 | 0.023107388 | No | | | |
| Itgb6 | ITGB6 | -2.852535817 | 0.023857372 | No | | | |
| Unc13d | UNC13D | -2.099334225 | 0.02995786 | No | | | |
| Pcdhgb1 | PCDHGB1 | -2.328204141 | 0.033411352 | No | | | |
| Chrdl2 | CHRDL2 | -2.120646749 | 0.034793546 | No | | | |
| Mlf1 | MLF1 | -2.402139369 | 0.036688503 | no | | | |
| Dnah17 | DNAH17 | -2.108037011 | 0.037914769 | Yes | Nothing related to MASLD | | |
| Srcin1 | SRCIN1 | -2.215911773 | 0.039688971 | Not in vivo | Srcin1 was shown to inhibit NFkB activity and expression of PTN (Target gene), which promotes EMT, mestastasis and angiogenesis In HBV-related HCC. | 29928866 | |
| Mrap2 | MRAP2 | -2.662592918 | 0.042310818 | Yes | Nothing related to MASLD | | |
| Gng2 | GNG2 | -3.139187407 | 0.043329519 | No | | | |
| Pkhd1l1 | PKHD1L1 | -2.632161724 | 0.044501998 | no | | | |
| C1qtnf1 | C1QTNF1 | -2.463377434 | 0.044654249 | No | | | |
| Srpx | SRPX | -2.480055729 | 0.045782422 | No | | | |

**Table S2 –** List of studies reporting association between different pathologies and miR-149 single nucleotide polymorphisms (rs71428439 and rs2292832) affecting miR-149 expression/maturation. In bold it is highlighted reports where variants were linked with non-alcoholic fatty liver disease and hepatocellular carcinoma.

| Variant ID | Effect | Disease | PMID |
|---|---|---|---|
| rs71428439 | AA carriers: Increased maturation of miR-149 linked with decreased apoptosis of cardiomyocytes | Myocardial infarction | 23873935 |
| | GG carriers: increased risk to develop renal carcinoma due to decreased miR-149 expression | Clear Renal Cell Carcinoma | 25213695 |
| | GG carriers: decreased risk do develop ischemic stroke | Ischemic Stroke | 25867405 |
| | GG carriers: increased risk to develop hepatocellular carcinoma due to downregulation of miR-149 and increased expression of AKT1 | **Hepatocellular Carcinoma** | 26550305 |
| | associated with lung cancer prevalence | Lung cancer | 30069329 |
| | AA carriers: decreased risk to develop extrapulmonary tuberculosis | Extrapulmonary tuberculosis | 31219360 |
| rs2292832 | TT carriers: increased risk to develop breast cancer and linked with decreased maturation of miR-149 | Breast Cancer | 36371555 |
| | TT carriers: decreased risk to develop acute lymphoblastic leukemia | Acute Lymphoblastic Leukemia | 35690283 |
| | CC carriers: improved survival rate in patients with hepatocellular carcinoma with bone metastasis | **Hepatocellular Carcinoma** | 35672274 |
| | CC carriers: decreased risk to develop cervical cancer | Cervical Cancer | 35495171 |
| | CC carriers: increased risk to develop coronary artery aneurysm | Kawasaki disease | 34969167 |
| | CC carriers: increased risk to develop thyroid cancer | Thyroid Cancer | 34643920; 25405731 |
| | CC carriers: increased risk to develop endometriosis | Endometriosis | 33583336 |
| | associated with earlier onset of stroke | Ischemic Stroke | 31811586 |
| | CC carriers: increased risk to develop extrapulmonary tuberculosis | Extrapulmonary tuberculosis | 31219360 |
| | possible link with risk to develop gastroinstestinal cancers | Gastrointestinal cancers | 30930933 |
| | CC carriers: increased risk to develop cervical cancer and linked with increased maturation of miR-149 | Cervical Cancer | 30852614 |
| | TT carriers: increased risk to develop colorectal cancer | Colorectal Cancer | 30447914 27706637 24568449 |
| | TT carriers: increased risk to develop gastric cancer | Gastric cancer | 30274913 23001871 |
| | CC carriers: increased risk to develop ischemic stroke | Ischemic Stroke | 30254431 26690224 |
| | CC carriers: increased risk to develop hepatocellular carcinoma in women | **Hepatocellular Carcinoma** | 30215231 |
| | decreased risk to develop HBV-related hepatocellular carcinoma | **Hepatocellular Carcinoma** | 29976775 |
| | CC carriers: later onset and milder symptoms of Charcot-Marie-Tooth disease type 1A | Charcot-Marie-Tooth disease type 1A | 29729827 |
| | CC carriers: increased risk to develop allergic rhinits and asthma | Allergic Rhinitis and Asthma | 28181414 |
| | CC carriers: improved survival rate in patients with non-small cell lung cancer | Non-small cell Lung Cancer | 27825117 23470291 |
| | TT carriers: increased risk to develop coronary heart disease | Coronary Heart Disease | 27430349 |
| | TT carriers: increased risk to develop inflammatory bowel disease | Inflammatory Bowel Disease | 27109937 |
| | CC carriers: increased disease-free survival in squamous cell carcinoma of the nonoropharynx | Squamous Cell Carcinoma | 27050146 |
| | TT carriers: increased risk to develop HBV-associated hepatocellular carcinoma | **Hepatocellular Carcinoma** | 25190221 |
| | CC carriers: increased risk for disease progression in nasopharyngeal carcinoma | Nasopharyngeal Carcinoma | 24648993 |
| | TT carriers: increased risk for disease progression and worse prognosis in head and neck squamous cell carcinoma | Head and Neck Squamous Cell Carcinoma | 23272122 |
| | TT carriers: primary hepatocytes increased miR-149 expression in response to fatty acid treatment, possibly link with NAFLD risk | **Non-Alcoholic Fatty Liver Disease** | 28507283 |

## Supplementary references


1. **Hansen HH**, Feigh M, Veidal SS, Rigbolt KT, Vrang N, Fosgerau K. Mouse models of nonalcoholic steatohepatitis in preclinical drug development. Drug Discov Today 2017;22:1707-1718.
2. **Berthou F**, Sobolewski C, Abegg D, Fournier M, Maeder C, Dolicka D, Correia de Sousa M, et al. Hepatic PTEN Signaling Regulates Systemic Metabolic Homeostasis through Hepatokines-Mediated Liver-to-Peripheral Organs Crosstalk. Int J Mol Sci 2022;23.
3. **Sobolewski C**, Abegg D, Berthou F, Dolicka D, Calo N, Sempoux C, Fournier M, et al. S100A11/ANXA2 belongs to a tumour suppressor/oncogene network deregulated early with steatosis and involved in inflammation and hepatocellular carcinoma development. Gut 2020;69:1841-1854.
4. **0uchi R**, Togo S, Kimura M, Shinozawa T, Koido M, Koike H, Thompson W, et al. Modeling Steatohepatitis in Humans with Pluripotent Stem Cell-Derived Organoids. Cell Metab 2019;30:374-384 e376.
5. **Li Q**, Dong Z, Lian W, Cui J, Wang J, Shen H, Liu W, et al. Ochratoxin A causes mitochondrial dysfunction, apoptotic and autophagic cell death and also induces mitochondrial biogenesis in human gastric epithelium cells. Arch Toxicol 2019;93:1141-1155.
6. **Calo N**, Ramadori P, Sobolewski C, Romero Y, Maeder C, Fournier M, Rantakari P, et al. Stress-activated miR-21/miR-21* in hepatocytes promotes lipid and glucose metabolic disorders associated with high-fat diet consumption. Gut 2016;65:1871-1881.
7. **Mithieux G**, Guignot L, Bordet JC, Wiernsperger N. Intrahepatic mechanisms underlying the effect of metformin in decreasing basal glucose production in rats fed a high-fat diet. Diabetes 2002;51:139-143.
